# Field evaluation of spring wheat genotypes reveals differential resistance to *Zymoseptoria tritici* in Ethiopia

**DOI:** 10.1371/journal.pone.0353375

**Published:** 2026-07-10

**Authors:** Habtewold Kifelew, Habtamu Terefe, Bekele Kasa, Tilahun Mekonen, Zelalem Bekeko, Bulti Tesso

**Affiliations:** 1 Haramaya University, College of Agriculture and Environmental Sciences, Dire Dawa, Ethiopia; 2 Holetta Agricultural Research Center, Holeta, Ethiopia; 3 Addis Ababa University, Institute of Biotechnology, Addis Ababa, Ethiopia; INRAE: Institut National de Recherche pour l’Agriculture l’Alimentation et l’Environnement, FRANCE

## Abstract

*Septoria tritici* blotch (STB), caused by *Zymoseptoria tritici*, is a major disease of spring wheat in Ethiopia and worldwide. This study evaluated 45 spring wheat genotypes for adult plant resistance under natural infection at Holetta Agricultural Research Center during the 2022 and 2023 seasons. Disease was assessed using two methods: (i) visual estimation of disease severity (DS) as the percentage of leaf area with necrotic lesions bearing pycnidia; and (ii) pycnidial density within lesions scored on a 0–5 scale to classify resistance levels. Combined analysis of variance across years showed significant effects (P ≤ 0.01) of year, genotype, and genotype × year interaction. Genotype accounted for 42.81% of the total variance in mean disease severity (from early grain filling to 70% flag leaf infection with pycnidia-bearing necrosis) and 42.3% of the variance in AUDPC. Mean severity ranged from 46.46% to 73.49% in 2022 and 8.73% to 71.71% in 2023, while pycnidial density ranged from 2.23% to 35.7% and 1.21% to 45.47% in 2022 and 2023, respectively. Four reaction classes were identified: resistant (24.4% and 25.2%), moderately resistant (30.4% and 24.4%), moderately susceptible (22.2% and 20.7%), and susceptible (22.96% and 29.63%) in 2022 and 2023, respectively. ‘Catbird’ was the most susceptible cultivar, whereas ‘Gondo’ was the most resistant. Cluster analysis grouped the cultivars into eight clusters based on STB severity and AUDPC, including two clusters (six genotypes) with resistant responses. These clusters did not include the differential lines used to characterize *Z. tritici* races, suggesting that the underlying resistance genes require further molecular characterization. The resistant genotypes (Blouk #1, 6B662, Coulter, Erik, Gondo, and ETW17–115) were effective against pathotypes virulent to major *Stb* genes present in Veranopolis (*Stb2*, *Stb6*), Shafir (*Stb6*), and Estanzuela Federal (*Stb7*). Disease severity and AUDPC were negatively correlated with plant height, number of tillers per plant, spike length, number of seeds per spike, thousand kernel weights, and grain yield. Correspondence and principal component analyses identified three major groups among the 45 genotypes: resistant, high-yield potential, and susceptible. The resistant genotypes identified here provide valuable material for breeding programs targeting improved resistance to *Z. tritici*.

## Introduction

Spring wheat is the primary cereal crop in Ethiopia, producing 4.93 million metric tons with an average productivity of 2.73 tons per hectare. It is cultivated by 4.2 million smallholder farmers across 1.8 million hectares [1]. Recently, due to the expansion of irrigated wheat production initiatives, Ethiopia has become one of the fastest-growing wheat producers in Africa. Ethiopian wheat production is expected to reach 6.5 million metric tons by the year 2025/26 [2]. However, Ethiopian wheat productivity remains low compared to Egypt (6.4 t ha⁻¹) [3]. The low productivity is linked to various biotic and abiotic factors, as well as limited adoption of modern agricultural technologies [4]. In terms of diseases, stem and leaf rust are prevalent in low and mid-altitude regions, while stripe rust (yellow rust) and Septoria are more common in high-altitude wheat-growing areas of Ethiopia [5–8]

Particularly in the highlands, *Zymoseptoria tritici,* the pathogen causing Septoria tritici blotch (STB) disease, is among the most devastating diseases of wheat in Ethiopia, ranking second only to stripe rust. This pathogen is responsible for yield losses that range from 41% [9] to 82% [5,6]. In addition to lowering productivity, *Z. tritici* infection degrades grain quality [10]. Due to frequent STB outbreaks and a lack of effective disease management strategies, wheat production in the area has recently become challenging.

Evaluating spring wheat for resistance to STB is essential in worldwide wheat breeding because of the pathogen evolutionary ability and the rising concern of fungicide resistance [11,12]. STB can lead to considerable yield reductions, making genetic resistance the most sustainable and eco-friendly control method. Screening initiatives mainly concentrate on detecting two types of resistance, qualitative and quantitative (associated with Quantitative Trait Loci, QTL). Qualitative resistance results from major *Stb* genes which confer strong, race-specific resistance, typically following a gene-for-gene interaction model. Though successful against non-virulent pathogen strains, they are susceptible to being outpaced by the pathogen’s swift development of virulence. To date, 24 resistance (R) genes against *Z. tritici* have been identified and documented [13]. Among them, 23 *Stb* genes have been genetically mapped in wheat [14]. These genes were found in wheat through field trials and studies on interactions between genotypes and isolates. Resistance to STB is primarily quantitative under field conditions [15,16]. Quantitative resistance offers a less complete yet long-lasting defense, governed by numerous genes (QTLs) with smaller, cumulative impacts. Contemporary breeding increasingly seeks a combination, or pyramiding, of both significant *Stb* genes and QTLs to enhance protection that is more resilient and durable. Currently, the wheat genome contains a large number of QTLs linked to resistance [17–19]. Since 2015, there have been findings indicating that 89 chromosomal regions harbor QTLs associated with resistance to STB [15]. Alternative origins, such as synthetic hexaploid wheat, are being explored to integrate newly effective resistance genes such as Stb16q [20]. A recent study using QTL analysis discovered that genes involved in both quantitative and qualitative STB-wheat interactions are similar. The study also demonstrated that genes underlying pathogenicity QTL can be analogous to *Avr* genes [21].

Studies in Ethiopia have revealed variability in resistance among bread wheat, durum wheat, and triticale [22]. Eyal and his team [23,24] investigated the virulence traits of 97 *Z. tritici* isolates obtained from 22 countries, including Ethiopia, by evaluating them on 35 varieties of wheat and triticale. Their findings confirmed isolate-cultivar specificity, indicating diverse virulence genes in the pathogen population. Molecular analyses by Tilahun [25] further demonstrated substantial genetic variability in resistance among 180 Ethiopian bread wheat genotypes. Genome-Wide Association Studies (GWAS) conducted on Ethiopian wheat have effectively pinpointed important genomic areas and new germplasm sources that provide consistent resistance to STB. These findings highlight the complex, polygenic basis of resistance and underline the importance of addressing genotype-by-environment interactions [26,27]. Screening programs in Tigray [28], Gondar [29], and Holetta [30] have also contributed to identifying resistant genotypes.

The purpose of this study was to assess phenotypic variation in spring wheat germplasm to identify resistance to STB. To achieve this, 45 spring wheat genotypes, including 10 differential lines for STB, were assessed under natural infection over two consecutive years at an open field site in the central highlands of Ethiopia. Agronomic traits were also measured on the same genotypes, enabling an analysis of the relationship between resistance and agronomic characteristics in Ethiopian spring wheat.

## Materials and methods

### Description of the study area

The genotypes were evaluated at the Holetta Agricultural Research Center in Ethiopia, under field conditions during the 2022 and 2023 cropping seasons. The location is expected to represent the major and potential spring wheat production areas in the central highlands of Ethiopia, the location is also recognized as a hotspot for STB epidemics. The center is 2,400 meters above sea level and located at latitude 9°00’ N and longitude 38°30’ E. The site has minimum and maximum temperatures of 6°C and 22°C, respectively; it receives 1,144 mm of rainfall annually (Table 1). The soil type, classified as a nitosols, has a pH of 6.0 [31].

**Table 1:** Weather condition of Holetta during spring wheat growing season 2022, 2023.

Weather variables*	Year 2022					Year 2023				
	June	July	August	September	Mean	June	July	August	September	Mean
T min (^O^C).	9	10.2	10.5	9.8	9.875	9.8	10.5	10.2	10	10.13
T max (^O^C).	26.5	25.7	25.9	24.5	25.65	22.3	20.9	21.2	21.4	21.45
Rainfall(mm)	200.7	390.8	331.7	218.7	285.48	112.4	202.3	271.8	166.4	188.23
RH (%)	77	84	81	78	80	81	84	84	80	82.25

Source:-Holetta agricultural research center, weather station

*T^O^ min = minimum temperature, T^O^ max = maximum temperature, Rainfall = mean monthly average rainfall amount, RH = Relative humidity percentage

### Plant materials

A total of 45 bread wheat genotypes were evaluated for their reaction to the *Z. tritici* field population at Holetta during the 2022 and 2023 cropping seasons. Most of these genotypes were recently introduced to the country, and their disease resistance status is not yet known. Differential lines—genotypes carrying known major resistance genes to STB were included so as to identify the specific races against which the genotype shows resistance, which can help in gene postulation. The susceptible variety “Pavon-76,” the moderately susceptible variety “Kingbird,” the moderately resistant variety “Alidoro,” and the resistant variety “Hedasie” were included as controls (Table 2).

**Table 2:** Spring wheat genotypes included in the study at Holetta, during 2022 and 2023 Cropping season.

Genotype short name	Genotype	Pedigree	Origin	Reason for including in this study and *Stb* genes	Reference
Biq	BIQA	ETBW 6095	EIAR	*Has Stb* (2, 3,5,7,8,9,14,18)	[25]
Mer	Meraro	*M/4/HAR 1709/ 3/M//24/E*	EIAR	*Has Stb* (5,6,7,8,11,13,16,17)	[25]
Ali	Alidoro	*HK-14-R251*	EIAR	*Has Stb* (2,3,4,5,6,7, 8,9, 11, 14,16,17,18)	[25]
Mad	Madda walabu	TI/3/Fn/Th/Nar59 *2/4/Bol’S’	EIAR	*Has Stb* (2,3,4,5,6, 8,9,13, 14,16,17,18)	[25]
Hul	Hulluka	*UTQUE96/3/PYN/BAU//MILAN*	OARI	*Has Stb* (3,4,5,7,8,13)	[25]
Kin	Kingbird	TAM200/TUI/6/PVN//CAR422/ANA/5/BOW/CROW//BUC/PVN/3/YR/4/TRAP#1	EIAR	*Has Stb* (1,2,3,4,5,7, 8,11,13,17)	[25]
Dan	Danda’a	KIRITATI//2*PBW65/2*SERI.1B	EIAR	*Has Stb* (2,3,4,7,9,13,14,18)	[25]
Et	ET-13A2	*UQ 105 SEL. X ENKOY*	EIAR	*Has Stb* (3,4,5,6,9,13,14)	[25]
Pavon	Pavon-76	*CMl399-D-4M-3Y-lM-l Y-lM-QY*	Mexico	*Has Stb* (3,5,6,8,9,11,12, 13, 14,17,18)	[25]
Sof	Sofumar	*LIRA ‘S’/TAN"S”*	OARI	*Has Stb* (2,3,7,8,9,13,14,17)	[25]
Ki6295	KI 6295-4A	*Romany X GB-GAMENYA*	EIAR	*Has Stb* (1,2,3,5,8,9,11,13, 14,16,17,18)	[25]
K6	K 6290 Bulk	*(AF.MAYOXGEM)XROMANY*	EIAR	*Has Stb* (2, 3,5,7,8,9,14,18)	[25]
Hog	Hoggana	*PYN/BAU//MILAN*	EIAR	*Has Stb* (5,6,7,8,11, 13,16,17)	[25]
Sal	Salamouni	NA	Canada	Stb13 + Stb14*	[32]
6B6	6B662	NA	Brazil	Differential line tan spot	[33]
Cou	Coulter	DT-188/Dt-224//DT-182	Israel	**	[34]
Eri	Erik	Kitt/2/Waldron/Era	USA/ HRSW-KS	**	[34]
Gle	Glenlea	Pemina*2/Bage/2/CB100	CIMMYT	Differential line for tan spot	[33]
Nd	ND-495	Justin*2/3/ND259/Conley//ND112	Uruguay	**	[34]
Ver	Veranopolis	K-44430; PI-214401; PI-297008; AFRC-5645	Ammiad, Israel	Has Stb2 + Stb6*	[35,36]
Isr	Israel 493	NA	Iran	Has the *Stb3* and Stb6*	[35]
Tad	Tadina	PI-494096; AUS-24432; AUS-23036	USA		[37,38]
Sha	Shafir	K-62169	CIMMYT	Stb6*	[39]
Est	Estanzuela Federal	NA	Brazil	Stb7*	[40]
Kk4	KK4500	NA	CIMMYT	Has the *Stb* (6,7,10,12)*	[38]
Km7	KM7	NA	CIMMYT	Stb16*	[41]
Gon	Gondo/CBRD	NA	USA	**	[34]
Br18	BR 18	NA	CIMMYT	**	[34]
Cat	Catbird	NA	CIMMYT	Used as source of resistance by CIMMYT	[42]
Br34	BR 34	ALZ110 / 2* IAS54 /6/ TP /4/ TZPP /SON64	CIMMYT	**	[34]
Fro	Frontana	NA	CIMMYT	Veranopolis and KK4500 are derived from Frontana	[43]
Mut	Mutus	NA	Brazil	**	[34]
Mur	Murga	NA	CIMMYT	Stb16*	[34]
Blo	Blouk #1	NA	CIMMYT	**	[34]
Chi	MP 4106	Chiba//PRLII/cM65531/3/SKAUZ/BAV92	CIMMYT	**	[34]
Cia	CIANO T79	CM31678-R-4Y-2M-21-0M	Mexico	**	[34]
Sok	Line	SOKOLL//W15.92/WBLL1	CIMMYT	**	[34]
Wax	Line	WAXWING*2/CIRCUS	ICSISA SB6737	**	[34]
Hid	Hidasie	ANAC/3/PRL/SARA//TSI/VEE#5/4/CROC-1/AE.SQUAROSA(224)//OPATTA	CIMMYT	Has good resistance to STB	
Etw86	Line	ETW17–86	EIAR	**	
Etw221	Line	ETW17–221	EIAR	**	
Etw246	Line	ETW17–246	EIAR	**	
Etw85	Line	ETW17–85	EIAR	**	
Etw115	Line	ETW17–115	EIAR	**	
Zer	Line	ZERBA-6/FLAG-6/3/TAM200/PASTOR//TOBA97	EIAR	**	

EIAR: Ethiopian Institute of Agricultural Research; CIMMYT: International Maize and Wheat Improvement Center; NA: Not available; *: STB differential line with known resistance major gene; **: the specific *Stb* genes were not clearly identified.

### Sowing and experimental design

Both experiments were established according to a randomized complete block design, with three replications. The experimental area underwent thorough tillage, being plowed three times prior to sowing, and planting rows were established with hand-pulled row maker. Seeds were sown using the hand drilling technique at a depth of approximately five cm. Spring wheat genotypes were planted on 28 June, in accordance with the recommended sowing period for spring wheat at Holetta [44]. Each plot consisted of four rows, each measuring one meter in length and spaced 20 cm apart, with a seeding rate of 150 kg per hectare [44]. Inorganic fertilizers were uniformly applied across all plots at a rate of 100 kg per hectare of di-ammonium phosphate (DAP) and 150 kg per hectare of urea, following the recommendations for spring wheat cultivation. The entire amount of DAP and half of the urea were applied at the time of planting, while the remaining half of the urea was added during the mid-tillering phase. Hand weeding was performed to manage weed growth in the experimental field [44].

### Disease assessment and computation of disease variables

Susceptibility and resistance levels were assessed based on the ability of the pathogen to infect host tissue and produce pycnidia. Disease assessments started at the early grain filling stage of wheat development [45], which is generally when the disease begins under Holetta conditions. Disease was assessed weekly for six weeks, ending when flag leaf infection reached 70% in susceptible genotypes. In each plot, five plants were randomly selected from each of the four corners and the center (X-shaped pattern), totaling 25 plants per plot for estimation of disease. Disease was assessed according to two criteria: disease severity (DS) and pycnidial density (PC).

Disease severity was estimated visually as the percentage of leaf area covered by necrotic lesions containing pycnidia. This assessment employed a modified version of the double-digit (00–99) scoring system developed by Saari and Prescott, which is based on a severity scale for evaluating foliar diseases in wheat [46,47]. The scoring system disease severity (1–9) is as follows: 10% coverage corresponds to a score of 1, 20% coverage to 2, and so on until 90% coverage corresponding to a score of 9. These measurements were combined to calculate the overall disease severity using the following formula:


$$\begin{array}{c} \text{Disease severity }(\%)=\left(\left(\frac{\text{D1}}{\text{Y1}}\right)*\left(\frac{\text{D2}}{\text{Y2}}\right)\right)*\text{100} \end{array}$$

Where D1 represents disease vertical progression relative to plant height. The greater the disease reaches upper leaves, especially when it affects the flag leaf (which plays a key role in yield), the more serious the damage. D2 reflects the diseased leaf area, and corresponds to the (1–9) score-scale described above. This provides the most straightforward indication of tissue damage and reduced ability for photosynthesis. Y1 is the maximum scale value for D1, which is the maximum possible score for the vertical progression of the disease, Y2 is the maximum scale value for D2, that is, the maximum possible score for the intensity of the diseased leaf area [46,47]. The mean of disease severity over the six assessments was then computed and used for further analyses, and is referred later-on as “disease severity.”

The area under the disease progress curve (AUDPC) of disease severity [25,48] was calculated using the following formula:


$$\begin{array}{c} AUDPC\%.day=\sum_{i=\text{1}}^{n-\text{1}}\frac{y_{i}+y_{i+\text{1}}}{\text{2}}\times (t_{i+\text{1}}-t_{i}) \end{array}$$

Where, y_i_ denotes disease severity at the i^th^ observation; t_i_ is the time, expressed in days, associated with the i^th^ observation; and n” is the total number of assessments.

The level of sporulation of lesions was estimated as pycnidial density within necrotic lesions using a 0–5 scale, where 0 = no sporulation; 1 = occasional pycnidia in a few lesions; 2 = low density of pycnidia in many or most lesions, usually unevenly distributed; 3 = even distribution of pycnidia at moderate density over most lesions; 4 = high density of pycnidia distributed over most lesions; and 5 = maximum pycnidial density [49,50] (Table 3).

**Table 3:** Pycnidial density (P) within necrotic lesions: scoring scale, range of infection percentage, mid-point percentage used to calculate means across replicates, and corresponding genotype reaction (infection type) classification [49].

Pycnidial density Score	Range of pycnidial density within a lesion (%)	Mid-Point	Infection type
0	0%	0%	Immune (I)
1	0.1–5%	2.5%	Resistant (R)
2	6–15%	10.5%	Mod. Resistant (MR)
3	16–30%	23.0%	Mod. Susceptible (MS)
4	31–40%	35.5%	Susceptible (S)
5	41–50%	45.5%	Susceptible (S)

The resistance levels of wheat genotypes were classified based on scores from genotype reaction (IT) obtained from the mean percentage of pycnidial density, calculated from six disease assessments. Genotype reactions (Infection types) were estimated and grouped using a 0–5 scale as described by Adhikari *et al*. and Louriki *et al*. [50,51]. Genotypes with pycnidiakl density scores ranging from 0 to 1 were classified as resistant (R), a score of 2 as moderately resistant (MR), a score of 3 as moderately susceptible (MS), and scores of 4–5 as susceptible (S) [51].

All essential agronomic data were collected from the middle two rows within each plot. The data collection was conducted on both a plant and plot basis. For the analysis of plant-based data, a random selection of 10 plants from each plot was made to evaluate a range of characteristics. These characteristics included the number of tillers per plant, the number of kernels per spike, the spikelet count per spike, as well as measurements of plant height (cm), and spike length (cm). Additionally, further data were gathered on the density of productive tillers per square meter, the weight of a thousand kernels (grams), and the grain yield (t/ha) on a plot-specific basis.

### Statistical analyses

In a first stage, the effects of genotype, year, and their interaction on disease severity (mean over six assessments), AUDPC of disease severity, and the agro-morphological variables were assessed with analyses of variance (ANOVA). The R statistical software (version 4.5.2) was used, following the standard procedures outlined by Gomez and Gomez [52]. Severity percentage data were transformed using arcsine transformation prior to analyses [53]. The validity of further statistical analyses, such as ANOVA, was ensured by using Bartlett’s test to determine whether the error variances for distinct genotypes across different attributes were comparable. ANOVA and other parametric statistical tests require the assumption of homoscedasticity, which was examined prior to analysis. When ANOVA indicated significant differences among genotypes, mean comparisons were performed using the least significant difference (LSD) test at a 5% level of significance.

In a second stage, associations between disease severity (mean over six assessments), AUDPC of disease severity, and agronomic traits were assessed with multivariate analyses. Diversity in disease resistance among genotypes was assessed using cluster analysis, correspondence analysis, and principal component analysis (PCA) based on agro-morphological and disease-related traits.

Continuous variables were converted into categorical ones because correspondence analysis is based on contingency tables [54, 55]. Quartiles provide a common and robust discretization method, dividing data into four equal-frequency groups and accommodating skewed distributions [56,57]. Continuous variables (disease and agronomic traits) were discretized into categorical variables, each one including four frequency-based groups (Q1–Q4). Quartile values for each variable are displayed in Table 4.

**Table 4:** Quartile values from wheat genotype categorization based on disease, yield, and agronomic traits, used to classify variables into High, Medium, and Low groups with corresponding genotypes.

Trait	Q1(Low)	Medium (Q2/Q3)	Q3 (high)
Genotype	–	–	–
Severity	0.7779	0.8514	0.9125
AUDPC	2027.7	2290.2	2577.2
Pycnidal density (P)	2.5	10.5	23
Plant height (PH)	83.5	88.0	97.2
Number of productive tiller per plant (NTPP)	2.507	2.601	2.692
Spike length (SL)	6.53	7.27	7.73
Number of seed per spike (NSPS)	6.40	6.48	6.69
Thousand kernel weight (TKW)	30.0	33.0	36.3
Yield (t/ha)	2.08	2.31	2.83

Cluster analysis was performed using Euclidean distance as the dissimilarity measure and Ward’s minimum variance method as the clustering criterion, based on the two-year mean values of AUDPC, severity, and pycnidia density. Correspondence analysis and PCA were used to explore associations among genotypes, disease variables, and agronomic traits. All analyses were conducted using R statistical software (version 4.5.2).

**ETHICS STATEMENT:** The study was approved by review and Ethics Committee, Haramaya University, Agriculture and Environmental Sciences School of Plant Sciences in February, 2022

## Results

### Analysis of Variance (ANOVA)

The combined analysis of variance (ANOVA) reveals a significant (P < 0.01) effect of the genotypes on disease severity and AUDPC (Table 5). The high significance of the year effect indicates that environmental conditions in 2022 and 2023 differed in their conduciveness to STB development. Furthermore, a significant Genotype × Year (G x Y) interaction suggests that specific genotypes responded differently to these seasonal variations. The ANOVA results further indicate that genotype is the main factor contributing to variation in resistance to STB within the collection of 45 spring wheat genotypes, accounting for 42.81% of the total variance in disease severity and 42.3% in AUDPC (Table 5). All genotypes showed high broad-sense heritability values (0.7094 for severity and 0.7132 for AUDPC; Table 5) for these traits, suggesting that resistance to STB can be enhanced through breeding.

**Table 5:** Analysis of variance for the effect of spring wheat genotype on *Septoria tritici* at Holetta, Ethiopia, during the 2022 and 2023 cropping seasons.

Trait	Factor	SS	Mean Square	Pr > F	%SS	h_2_^2^%
Severity*	Genotype	3.233	0.074	<.0001	42.81	0.7094
	year	0.562	0.562	<.0001	7.4	
	Genotype*year	2.994	0.07	<.0001	39.6	
	Residual	0.763	0.0043		10.1	
AUDPC	Genotype	56508295.41	1284279.44	<.0001	42.3	0.7132
	year	8214937.84	8214937.84	<.0001	6.15	
	Genotype*year	54402880.83	1236429.11	<.0001	40.7	
	Residual	14508570	81508.8		10.86	

SS- a sum of squares; MS- mean squares; h_b_^2^- broad-sense heritability index.

*Severity value refers to the average of severity over the 6 assessments

### Genotype-by-year interaction effects

The combined analysis of variance (ANOVA) across the 2022 and 2023 growing seasons revealed highly significant differences (P < 0.01) among genotypes, years, and their interactions for nearly all investigated traits (Table 6). The highly significant genotype effect (MSG) for STB severity, AUDPC, and grain yield confirms the presence of substantial genetic diversity within the 45 spring wheat genotypes, providing a robust basis for identifying resistant and high-yielding lines. While the significant year effect (MSY) and genotype-by-year interaction (MSGY) for disease variables and most agronomic traits highlight the strong influence of seasonal environmental fluctuations on STB development and plant growth, the non-significant MSGY for grain yield (0.015^NS^) suggests that the relative yield performance of the genotypes remained stable across environments. Furthermore, high coefficients of determination (R^2^) ranging from 0.89 to 0.95 for the primary disease and growth metrics, coupled with manageable coefficients of variation, underline the reliability of the experimental conditions at Holetta for differentiating the phenotypic responses of the wheat population (Table 6).

**Table 6:** Combined analysis of variance over the year for STB Severity, grain yield, and yield-related characters of spring wheat genotype grown at Holetta during the 2022 and 2023 growing seasons.

Trait	MSG (44)	MSY (1)	MSGY (44)	MSB	MSE	Mean	Cv.	R^2^	LSD
Severity	0.0735^**^	0.5618^**^	0.068^**^	0.0299^**^	0.00428	0.83	7.86	0.90	0.0746
AUDPC	1284279.44^**^	8214937.84^**^	1236429.11^**^	468196.27^**^	81508.8	2317.52	12.32	0.89	325.28
Plant height	1041.048^**^	31948.03^**^	602.541^**^	167.315^**^	30.2661	92.063	5.98	0.95	6.268
Number of tillers per plant	0.0819^**^	12.752^**^	0.0704^**^	0.2326^**^	0.0677	2.590	10.05	0.62	0.2965
spike length	4.3226^**^	428.65^**^	4.2447^**^	0.2028^**^	0.3393	7.253	8.03	0.93	0.6636
Number of seeds per spike	0.9343^**^	0.60314^**^	0.8623^**^	0.4579^**^	0.1959	6.481	6.83	0.70	0.5043
Thousand kernel weight	228.58199^**^	11774.4^**^	249.1234^**^	126.693^**^	163.824	33.515	38.19	0.53	14.583
Yield tons/hectare	1.999^**^	0.641^**^	0.015^NS^	0.01^NS^	0.05	2.43	9.03	0.91	0.2503

*, ** Significant at p ≤ 0.05, and p ≤ 0.01 probability level respectively, () parenthesis indicate degrees of freedom.

MSG = Mean Squares of Genotype, MSY = Mean squares of year, MSGY = Mean square of genotype x year interaction, MSE = Mean squares of error, MSB = Mean squares of block, CV = Coefficient of variation.

### Phenotypic diversity for STB resistance

The evaluation of 45 spring bread wheat genotypes at Holetta revealed wide variation in response to STB, allowing classification into four infection types (R, MR, MS, S) (S1 Table). Resistant genotypes such as 6B662, Erik, Coulter, and Gondo consistently showed low disease severity and pycnidial density (0.25–7.11), indicating stable resistance across years. In contrast, susceptible genotypes including Catbird, Tadina, and Veranopolis exhibited high severity (>65%) and pycnidial density (up to 45.47), reflecting rapid disease development and high inoculum production. A strong positive association was observed between severity and pycnidial density.

Mean separation analysis showed significant differences (P < 0.05) among genotypes for severity and AUDPC, grouping them into distinct statistical classes. Resistant genotypes formed the lowest mean groups, while susceptible ones ranked highest. Overall, substantial phenotypic diversity was observed, with genotypes distributed across all resistance categories, indicating strong variation in STB resistance.

### Disease progression and intensity (severity & AUDPC)

#### Analysis of STB severity.

**Fig 1:**
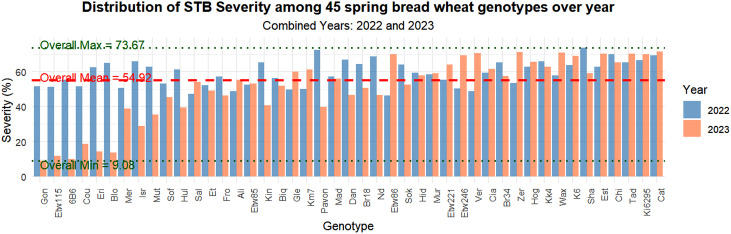
Distribution of *Septoria Tritici* Blotch (STB) severity (%) among 45 spring bread wheat genotypes evaluated across two cropping seasons (2022 and 2023). The horizontal red dashed line represents the overall mean severity (54.92%), while the upper and lower green dotted lines indicate the overall maximum (73.67%) and minimum (9.08%) values, respectively.

The performance of the 45 spring bread wheat genotypes regarding STB severity across both experimental years is illustrated in Fig 1. Panel A (2022) shows a relatively high baseline of infection for most genotypes. Panel B (2023) highlights a clear divergence: the resistant subset (e.g., 6B6, Etw115) shows a sharp decrease in severity compared to 2022, while susceptible lines (e.g., Catbird, Tadina) maintain or exceed 2022 levels, reaching the overall maximum of 73.67%.

The analysis revealed high disease levels across the population, with a grand mean severity of 54.92%. Genotypic responses exhibited a wide range of variation, spanning from 9.08% to 73.67%. Specifically, genotypes 6B662, ETW17–115, Coulter, Erik, and Blouk#1 demonstrated superior resistance, maintaining severity levels significantly below the population mean, a trend most pronounced during the 2023 cropping season.

Conversely, KM7, Shafir, Estanzuela Federal, and Catbird were identified as highly susceptible, with severity ratings frequently exceeding 60–70%. A significant genotype × year (G × Y) interaction was observed; while resistant genotypes maintained or improved their performance in 2023, several susceptible entries notably— ZERBAA-6/FLAG-6/3/TAM200/PASTOR//TOBA97, WAXWING*2/CIRCUS, and ETW17–246—exhibited markedly increased disease intensity during the same period.

#### STB diseases progress analysis using AUDPC.

**Fig 2:**
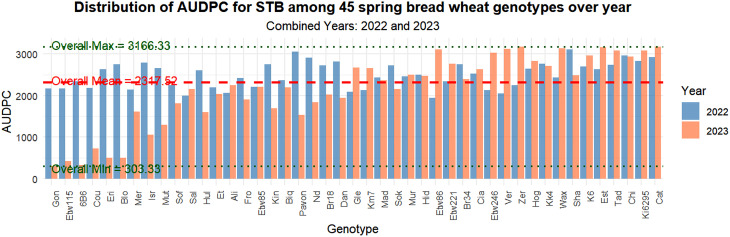
Distribution of Area Under Disease Progress Curve (AUDPC) values for *Septoria Tritici* Blotch (STB) among 45 spring bread wheat genotypes. Values represent the cumulative disease progress over the growing period, with lower values indicating higher levels of quantitative resistance. The mean value (2317.5) serves as a benchmark for identifying superior genotypes relative to the population average.

The performance of the 45 spring bread wheat genotypes regarding the Area Under the Disease Progress Curve (AUDPC) for *Septoria Tritici* Blotch (STB) is illustrated in Fig 2. While the AUDPC values mirrored the severity trends, they provided a more comprehensive, cumulative assessment of the epidemic progression. Panels A (2022) and B (2023) demonstrate that although the grand mean remained high (2317.52), genotypes categorized as “Low AUDPC” (positioned on the left of the x-axis) consistently restricted disease expansion across both seasons.

Statistical analysis of AUDPC revealed substantial phenotypic variation among the genotypes. Responses spanned a broad spectrum, ranging from highly resistant to highly susceptible. A distinct cohort, including ETW17–115, 6B662, Coulter, Erik, and Blouk#1, exhibited the slowest disease progression, with AUDPC values falling significantly below the population mean—reaching a minimum of 303.33 in 2023.

Conversely, genotypes such as Pavon-76, ETW17–86, WAXWING*2/CIRCUS, and Catbird consistently approached the maximum AUDPC value of 3166.33, confirming their high susceptibility. The notable fluctuations in AUDPC values for several genotypes between the two years further underscore a significant genotype-by-year (G × Y) interaction, suggesting that seasonal environmental variations played a critical role in STB epidemic development within the screening field (Fig 2).

#### Comparative analysis of pycnidia density across spring wheat genotypes and resistance levels (2022–2023).

**Fig 3:**
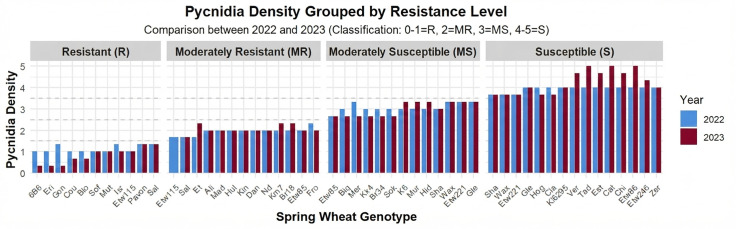
Pycnidia density scores of 45 spring bread wheat genotypes grouped by resistance levels (R, MR, MS, and S) during the 2022 and 2023 cropping seasons. Density was rated on a 0–5 scale, where 0–1(0% − 2.5%) indicates resistance and 4–5 (35.5% − 45.5%) indicates high susceptibility. The faceted arrangement highlights the differential capacity of genotypes to suppress fungal sporulation across varying levels of host resistance.

When genotypes were grouped by phenotypic resistance classification, a clear relationship emerged between visible foliar damage and pathogen reproduction (Fig 3). The evaluation of pycnidia density across the four resistance classes revealed a stepwise increase in fungal colonization corresponding to host susceptibility.

Genotypes in the Resistant (R) group notably 6B662, Erik**,** and Gondo/CBRD effectively suppressed fungal reproduction, maintaining density scores between 0% and 2.5%. This suppression was further enhanced during the 2023 season, where many R-group genotypes scored below 0.5. In contrast, the Moderately Resistant (MR) and Moderately Susceptible (MS) groups exhibited high stability, with density scores consistently clustering around 2 (10.5%) and 3 (23.0%), respectively, across both experimental years.

The Susceptible (S) group demonstrated the highest reproductive potential. While the S group consistently scored between 3 (23.0%) and 4(35.5) in 2022, a further intensification was observed in 2023, with several genotypes including Veranopolis, Tadina, Estanzuela Federal, and Catbird attaining the maximum score of 5 (45.5).

These findings indicate a strong positive correlation between disease severity and the density of *Z. tritici* pycnidia. The results suggest that the most resistant genotypes provide a dual benefit: they not only limit necrotic leaf tissue damage but also significantly restrict the secondary inoculum potential within the field environment.

### Multivariate analysis of agronomic and disease traits

#### Cluster analysis of genotypic responses.

**Fig 4:**
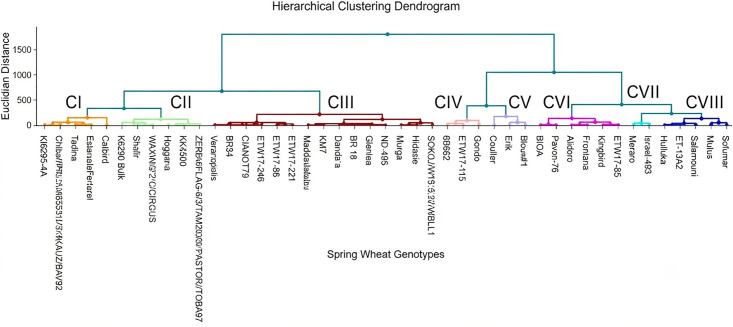
Hierarchical clustering dendrogram (UPGMA) of 45 spring bread wheat genotypes based on *Septoria Tritici* Blotch (STB) epidemiological variables (Severity, AUDPC, and Pycnidia density) under natural infection conditions in Holetta, Ethiopia (2022–2023). **CI-CVIII = Roman numbers showing cluster; Metric variables are:- AUDPC and Severity.

Cluster analysis based on mean disease severity, AUDPC, and pycnidia density grouped the spring wheat genotypes into eight distinct clusters at a similarity threshold of 1500 (Euclidean distance) using the UPGMA method (Fig 4 and S2 Table). These clusters were further organized into two major groups: Group A (Clusters I–III) and Group B (Clusters IV–VIII), clearly separating susceptible and resistant responses. Clusters I and II exhibited the highest disease intensity, with Cluster I (N = 5) showing maximum severity (68.16%), AUDPC (2948.87), and pycnidia density (45.5%), followed by Cluster II (N = 6), which also displayed high susceptibility (severity = 64.75%, AUDPC = 2762.67). Cluster I included genotypes such as Estanzuela Federal (Stb7, Stb12), which showed susceptibility under Holetta conditions, while Cluster II contained KK4500 (*Stb6, Stb7, Stb10, Stb12*) and Shafir (*Stb6*), both exhibiting moderately susceptible reactions. Cluster III (N = 15), the largest group, represented moderate-to-high disease response (severity = 58.42%, AUDPC = 2471.47), and included genotypes such as Veranopolis (*Stb2, Stb6*), KM7 (*Stb16)*, and Murga (*Stb16*), with reactions ranging from susceptible to moderately resistant. Clusters VI, VII, and VIII showed intermediate disease responses, although Cluster VII (N = 2) exhibited high variability in pycnidia density, indicating heterogeneity among its genotypes.

In contrast, Group B (Clusters IV–VIII) was dominated by relatively resistant genotypes. Cluster IV (N = 3) recorded the lowest disease severity (28.95%) and AUDPC (1289.17), indicating strong resistance, while Cluster V (N = 3) showed slightly higher disease levels but the lowest pycnidia density (0.78), suggesting limited pathogen reproduction. Clusters IV, V, and VI collectively comprised genotypes with resistant to moderately resistant reactions and lacked differential lines. Cluster VII included Israel-493 (*Stb3, Stb6*), which exhibited resistance, and Meraro (*Stb6*), which was moderately susceptible, emphasizing the potential contribution of *Stb3* to resistance. Cluster VIII (N = 5), including Salamouni (*Stb13, Stb14*), displayed consistent resistant reactions with low variability. Notably, resistant genotypes identified in this study—Blouk#1, 6B662, Coulter, Erik, Gondo, and ETW17–115—demonstrated effectiveness against pathotypes virulent to major *Stb* genes found in susceptible genotypes such as Veranopolis (*Stb2, Stb6*) and Estanzuela Federal (*Stb7*). Overall, the clustering pattern effectively distinguished highly susceptible genotypes (Clusters I–II) from resistant ones (Clusters IV–V and VIII), with intermediate clusters bridging the two extremes, providing valuable insights for resistance breeding.

### Correlation analysis of epidemiological and agronomic traits

**Fig 5:**
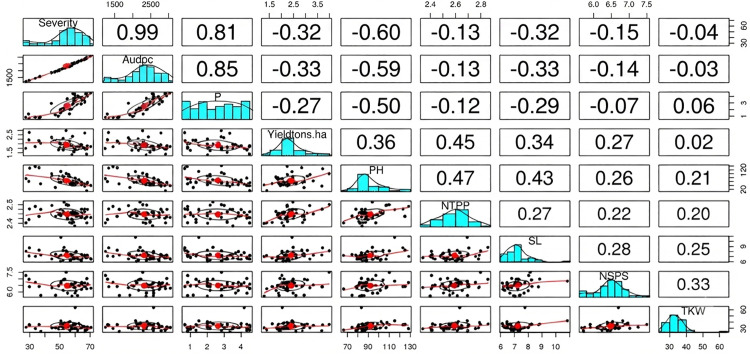
Scatter plot matrix showing mean severity scored from pycnidia bearing necrosis starting from early grain filling stage to flag leaf infection reach 70% with pycnidia bearing necrosis and AUDPC relation with yield and yield related trait on 45 spring wheat genotypes evaluated during 2022 and 2023 cropping season.

The Pearson correlation analysis revealed a near-perfect positive association between STB severity and AUDPC (r = 0.99), indicating that single-point severity assessments were highly representative of cumulative disease progression throughout the season (Fig 5; S1 Fig). Similarly, pycnidia density (P) showed strong positive correlations with both severity (r = 0.81) and AUDPC (r = 0.85), confirming that genotypes with higher levels of leaf necrosis also supported significantly greater fungal reproduction organs.

Regarding agronomic impact, both severity and AUDPC exerted significant negative effect on grain yield (r = −0.32 and r = −0.33, respectively) and plant height (r = −0.60 and r = −0.59, respectively). The strong negative correlation with plant height suggests that shorter genotypes in this population may be more vulnerable to vertical disease progression, or alternatively, that severe infection early in the season led to significant growth stunting. Interestingly, while the disease significantly reduced the number of productive tillers per plant (NTPP; r = −0.13) and spike length (SL; r = −0.33), the correlation with thousand kernel weight (TKW) was near zero (r = −0.04 for severity; r = −0.03 for AUDPC). This lack of a linear relationship implies that STB-induced yield losses in these 45 genotypes were primarily driven by a reduction in reproductive sinks—such as tiller survival and seeds per spike rather than by a decline in the efficiency of individual grain filling.

### Multiple correspondence analysis (MCA) of epidemiological and agronomic traits

**Fig 6:**
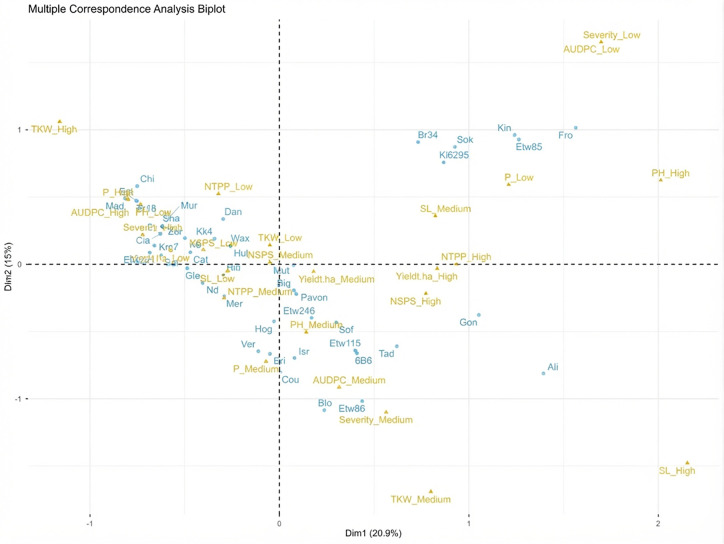
Ordination of nine variables by correspondence analysis. Active variables included Severity, AUDPC, PH (plant height), TKW (thousand kernel weight), SL (spike length), NTPP (number of productive tillers per plant), P (pycnidia density), grain yield, and 45 spring wheat genotypes.

The Multiple Correspondence Analysis (MCA) biplot (Fig 6) integrated the 45 spring wheat genotypes with their categorized disease and agronomic traits, explaining 35.9% of the total phenotypic variation along a well-defined resistance–susceptibility gradient. Dimension 1 (20.9%) functioned as the primary axis of health and productivity, contrasting high-disease indicators specifically (Severity_High, AUDPC_High, and P_High) with superior yield components. Genotypes clustered on the positive side of this dimension, such as Frontana, Kingbird, and Gondo, were strongly associated with low disease levels and high values for grain yield and reproductive sinks, including the number of productive tillers (NTPP) and seeds per spike (NSPS). In contrast, susceptible genotypes like Madda Walabu and Estanzuela Federal aligned with maximum disease intensity and reduced agronomic performance. Dimension 2 (15.0%) further differentiated genotypes based on grain weight and architecture, revealing that high thousand kernel weight (TKW_High) was somewhat decoupled from overall resistance and total grain yield. These findings effectively synchronize the results of the cluster and principal component analyses, identifying a distinct group of elite genotypes as primary candidates for STB resistance breeding programs.

### Principal component analysis (PCA) of epidemiological and agronomic traits

Principal Component Analysis (PCA) showed that the first nine components explained 100% of the total variation among the 45 spring wheat genotypes for agronomic and *Z. tritici*-related traits (S3Table). Based on the Kaiser criterion (eigenvalues > 1), only the first three components were considered significant, jointly explaining 73% of the total variance. PC1, PC2, and PC3 contributed 42%, 19%, and 11% of the variation, respectively, with PC1 (eigenvalue = 3.817) representing the primary source of morphological and pathological diversity. The sharp decline in standard deviation from PC1 (1.95) to subsequent components indicates that most of the variability is captured within the first few dimensions, supporting their use for multivariate analysis and genotype classification.

**Fig 7:**
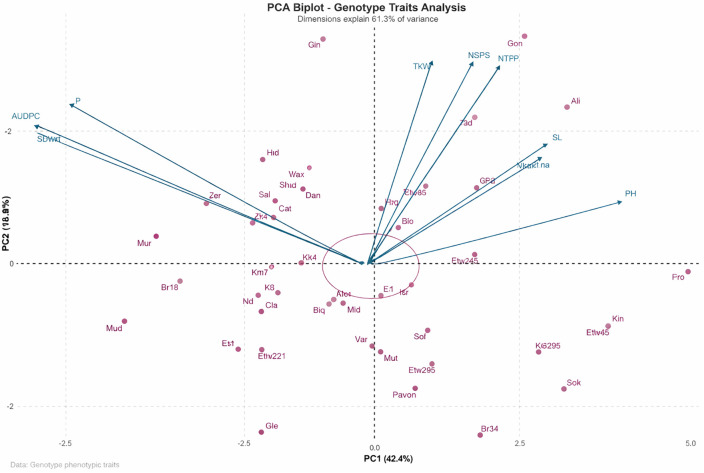
Result of principal component analysis considering disease variables, yield and yield related variables of forty five different spring wheat genotypes included in the study, Holetta, Ethiopia.

The PCA biplot further illustrated the relationships between genotypes and traits, with the first two components explaining 61.3% of the total variance (Fig 7). PC1 (42.4%) represented a strong yield–disease gradient, showing a clear negative association between disease traits (severity, AUDPC, and pycnidia density) and yield-related traits such as grain yield, spike length, and number of seeds per spike. Resistant genotypes, including Frontana, Kingbird, Gondo, and Alidoro, were associated with higher yield performance, whereas susceptible genotypes such as Madda Walabu, Estanzuela Federal, and Catbird clustered with high disease levels and reduced agronomic performance. PC2 (18.9%) further distinguished genotypes based on yield components, particularly thousand kernel weight and seeds per spike, reinforcing the identification of high-performing, STB-resistant genotypes.

## Discussion

The present study provides a comprehensive phenotypic evaluation of 45 spring wheat genotypes for resistance to *Zymoseptoria tritici*, the causal agent of Septoria tritici blotch (STB), under field conditions in Ethiopia across two growing seasons. STB remains one of the most destructive foliar diseases of wheat in the Ethiopian highlands and similar agro-ecological zones, where cool temperatures and prolonged moisture during the main cropping season create highly favorable conditions for epidemic development [58]. By integrating standard disease assessments, pathogen reproductive indicators, and agronomic traits through univariate and multivariate analyses, this study provides insights into the relationships among host resistance, pathogen development, and yield performance under the conditions of the present study. The findings provide useful information for identifying resistance sources and refining selection strategies in wheat breeding programs aimed at improving STB resistance and yield stability.

The ANOVA results revealed substantial genetic variability for STB resistance among the evaluated germplasm. Such genotypic differentiation is a prerequisite for effective phenotypic selection and agrees with previous studies reporting wide variation in STB responses among diverse wheat collections [43,59]. The existence of broad variability is particularly important for Ethiopian breeding programs, which frequently rely on the incorporation of resistance from exotic and historically utilized germplasm to broaden the genetic base of locally adapted cultivars [60]. The identification of resistant and moderately resistant genotypes therefore provides valuable material for future crossing programs.

Despite the strong genotypic effect, the highly significant year effect demonstrated the major influence of environmental conditions on STB epidemic development. Infection, lesion expansion, and pycnidial sporulation of *Z. tritici* are favored by prolonged periods of leaf wetness and moderate temperatures ranging from approximately 15–20°C [61,62]. Variations in rainfall distribution and duration of wet periods between the 2022 and 2023 growing seasons likely altered infection efficiency and disease progress. Similar inter-annual fluctuations have frequently been reported in STB epidemiology and highlight the importance of evaluating germplasm across multiple seasons to capture environmentally driven variation in resistance expression [63].

The significant Genotype × Year (G × Y) interaction further indicated that resistance expression varied between seasons. Consequently, the relative ranking of some genotypes changed depending on environmental conditions, complicating selection decisions [64]. Comparable interactions have been documented previously in the wheat–*Z. tritici* pathosystem, particularly for quantitative resistance mechanisms that are influenced by temperature, humidity, and inoculum pressure [65]. Quantitative resistance, which is typically governed by multiple genes of minor effect, often exhibits greater environmental sensitivity than race-specific major gene resistance [66]. Nevertheless, such resistance is generally considered more durable because it imposes lower selection pressure on pathogen populations. These findings emphasize the need for multi-environment testing to identify resistance sources with both high effectiveness and stable performance.

Although environmental effects were substantial, genotype accounted for a considerable proportion of the phenotypic variation observed for disease severity (42.81%) and AUDPC (42.3%). The relatively high broad-sense heritability estimates for disease severity (H² = 0.7094) and AUDPC (H² = 0.7132) further indicate that much of the observed variation was genetically controlled. These values are comparable to previous estimates reported for the wheat–STB pathosystem [38,67] and suggest that phenotypic selection based on these traits should be effective, even in the presence of significant G × Y interactions [68]. The moderate-to-high heritability estimates also support the potential utility of these traits in breeding pipelines where direct field selection remains an important strategy.

Beyond visual disease symptoms, the assessment of pycnidia density provided additional insights into pathogen reproductive success. The positive association observed between foliar damage and pycnidia density indicates that host susceptibility influences both tissue colonization and pathogen multiplication. Previous studies have shown that resistance to *Z. tritici* may involve two partially independent components: restriction of host damage, expressed as reduced necrosis and chlorosis, and limitation of pathogen reproduction, reflected by lower pycnidia production [22,69,70]. Incorporating both components into resistance evaluations therefore offers a more complete characterization of host responses than reliance on visual severity alone.

Resistant genotypes such as 6B662, Erik, and Gondo/CBRD consistently exhibited low pycnidia density scores, suggesting reduced pathogen reproduction under the experimental conditions. Conversely, susceptible genotypes including Veranopolis, Tadina, Estanzuela Federal, and Catbird displayed greater pycnidia production and therefore a higher potential for inoculum generation. However, because inoculum dynamics were not directly quantified, these observations should be interpreted cautiously.

The positive correlation between disease severity and pycnidia density has potential epidemiological implications. As STB is a polycyclic disease, secondary inoculum production plays a major role in epidemic acceleration. Genotypes combining reduced disease severity with limited pycnidia production could potentially slow disease spread within cropping systems by restricting pathogen multiplication and reducing inoculum pressure on neighboring susceptible plants [71]. Such “buffering effects” may contribute to epidemic suppression in varietal mixtures or regional deployment strategies. Nonetheless, validation of these effects under field and landscape conditions will require dedicated epidemiological investigations [63,72].

Multivariate cluster analysis grouped genotypes into distinct response categories and suggested differences in the effectiveness of previously characterized resistance genes. Genotypes reported to carry *Stb6*, *Stb7*, *Stb10*, *Stb12*, and *Stb16* were predominantly classified within susceptible to moderately susceptible groups under the conditions of this study. For example, Estanzuela Federal (*Stb7* and *Stb12*), KK4500 (carrying multiple *Stb* genes), and Shafir (*Stb6*) exhibited relatively high disease levels. *Stb6*, the first cloned STB resistance gene, has been widely deployed worldwide but has lost effectiveness in several regions due to the emergence of virulent *Z. tritici* populations [73]. The observed susceptibility of these genotypes may therefore indicate reduced effectiveness of these major resistance genes against Ethiopian pathogen populations, consistent with previous reports of pathogen adaptation to race-specific resistance [74,75]. However, because virulence characterization of pathogen isolates was beyond the scope of this study, definitive conclusions regarding virulence evolution cannot be made.

In contrast, genotypes such as Blouk#1, 6B662, and Salamouni displayed comparatively high levels of resistance. Salamouni, in particular, is internationally recognized as a source of durable adult plant resistance to multiple foliar diseases, including STB and leaf rust [76]. Their favorable performance likely reflects the presence of effective polygenic resistance factors that remain functional under Ethiopian conditions. This observation reinforces the widely accepted view that quantitative resistance often provides greater durability than single major genes when deployed against genetically diverse pathogen populations.

Correlation analyses further illustrated the agronomic consequences of STB infection. Disease severity and AUDPC were negatively associated with grain yield (r ≈ −0.32) and plant height (r ≈ −0.60), supporting evidence that increasing disease pressure adversely affects crop productivity. The stronger association with plant height may reflect genotype-specific canopy architecture effects influencing within-canopy humidity and disease development, or alternatively, the inhibitory effects of severe disease on plant growth [77–79]. Disease severity was also negatively associated with productive tiller number and spike length, whereas thousand-kernel weight (TKW) showed only a weak relationship with disease severity (r ≈ −0.04). Similar findings have been reported previously and are often attributed to the timing of infection: early disease development reduces tiller survival and reproductive development, while grain filling may be partially compensated through remobilization of assimilates from healthy tissues [80].

Multivariate analyses provided an integrated assessment of genotype performance. The PCA biplot explained 61.3% of the total variation and revealed a clear contrast between disease-related variables and productivity traits. PC1 (42.4%) separated genotypes exhibiting lower disease levels and superior agronomic performance from those characterized by greater disease susceptibility. Genotypes such as Frontana, Kingbird, Gondo, and Alidoro occupied the favorable region of the biplot associated with reduced disease and improved productivity. Similarly, MCA identified relationships between lower disease levels and favorable reproductive characteristics, including productive tiller number and seeds per spike. In contrast, TKW appeared relatively independent of the principal disease–yield gradient. These findings indicate that reliance on a single trait, particularly visual disease severity, may not adequately capture overall genotype performance or potential yield penalties under STB pressure [81].

The consistent performance of Frontana, a widely recognized source of partial resistance, supports its continued value as a benchmark genotype in STB resistance studies [77,82]. Originally identified in Brazil, Frontana has maintained effectiveness across diverse environments, likely due to its complex genetic architecture involving multiple minor-effect quantitative trait loci [83]. Furthermore, the favorable combination of comparatively low disease levels and acceptable agronomic performance observed in Gondo, Kingbird, Erik, Blouk#1, and 6B662 suggests that these genotypes represent promising sources of resistance for breeding programs aimed at enhancing STB management and yield stability in Ethiopia.

Overall, this study demonstrates that substantial exploitable genetic variation for STB resistance exists within the evaluated spring wheat germplasm. The integration of disease severity, pathogen reproductive traits, and agronomic performance enabled the identification of genotypes expressing both effective resistance and desirable productivity attributes. The results support the strategic deployment of quantitative resistance and highlight the importance of multi-environment evaluations for identifying durable resistance sources. Future studies integrating phenotypic evaluations with pathogen virulence characterization and molecular analyses of resistance loci will further strengthen breeding efforts directed toward the development of wheat cultivars with stable resistance to STB under Ethiopian production conditions.

## Conclusion

This study demonstrated substantial variation in resistance to *Septoria tritici* blotch (STB) among 45 bread wheat genotypes evaluated under Ethiopian field conditions. Genotypes such as Blouk #1, 6B662, Coulter, Erik, Gondo, and ETW17–115 exhibited strong resistance, whereas Catbird was highly susceptible. Pathotype analysis indicated widespread virulence against major *Stb* genes (*Stb2*, *Stb6*, and *Stb7*), highlighting the declining effectiveness of commonly deployed resistance genes, while avirulence was observed for *Stb3*, *Stb13*, *Stb14*, and *Stb16*. High heritability estimates and significant genotypic effects confirmed that STB resistance is largely genetically controlled, despite notable environmental influence. The strong association between disease severity and pycnidia density emphasizes the importance of selecting genotypes that limit both foliar damage and pathogen reproduction. Multivariate analyses identified Blouk #1, Gondo, Erik, 6B662, and Kingbird as elite genotypes combining low disease severity, restricted pathogen reproduction, and stable yield components. These genotypes represent valuable resources for breeding programs aiming to develop high-yielding, durably resistant wheat varieties adapted to Ethiopia and similar environments.

## Supporting information

S1 Table Leaf blotch severity (LBS), infection type (IT), and pycnidia density (P) of bread wheat genotypes in response to Septoria tritici blotch (STB) at Holetta, Ethiopia, during the 2022 and 2023 main cropping seasons. (DOCX)

S2 Table Cluster characteristics based on mean, standard deviation, and standard error of the mean for agronomic and STB-related traits in 45 spring wheat genotypes. (DOCX)

S3 Table Eigenvalues, proportion of variance, and cumulative variance explained by the first nine principal components for agronomic and STB-related traits in 45 spring wheat genotypes. (DOCX)

S1 Fig Correlation between disease severity (%) and area under the disease progress curve (AUDPC) for Septoria tritici blotch (STB) in 45 spring wheat genotypes. A strong positive linear relationship was observed (r = 0.994, R^2^ = 0.988). Data points are color-coded by resistance group, showing distinct clustering of highly resistant genotypes in the lower-left and highly susceptible genotypes in the upper-right of the plot.
(DOCX)
