# Correction: Field evaluation of spring wheat genotypes reveals differential resistance to *Zymoseptoria tritici* in Ethiopia

**DOI:** 10.1371/journal.pone.0355642

**Published:** 2026-08-06

**Authors:** Habtewold Kifelew, Habtamu Terefe, Bekele Kasa, Tilahun Mekonnen, Zelalem Bekeko, Bulti Tesso

The fourth author’s name is spelled incorrectly. The correct name is: Tilahun Mekonnen. The correct citation is: Kifelew H, Terefe H, Kasa B, Mekonnen T, Bekeko Z, Tesso B (2026) Field evaluation of spring wheat genotypes reveals differential resistance to Zymoseptoria tritici in Ethiopia. PLoS One 21(7): e0353375. https://doi.org/10.1371/journal.pone.0353375.
