# Supplementary material for: Field evaluation of spring wheat genotypes reveals differential resistance to Zymoseptoria tritici in Ethiopia
Source: PLoS One. 2026 Jul 10;21(7):e0353375. doi: 10.1371/journal.pone.0353375 (PMC13353992; doi:10.1371/journal.pone.0353375)
Supplement: S1 Table — (DOCX) [file pone.0353375.s001.docx]

**Table S1.**

| Spring wheat Genotypes | 2022 | | | 2023 | | |
| --- | --- | --- | --- | --- | --- | --- |
|  | %LBS | P | IT | %LBS | P | IT |
| Etw17-246 | 50.46^MNO^ | 35.66^A^ | S | 68.97^ABCD^ | 38.56^AB^ | S |
| Tadina | 65.22^ABCDEFG^ | 35.66^A^ | S | 69.89^ABC^ | 45.47^A^ | S |
| Etw17-86 | 46.46^NO^ | 35.66^A^ | S | 69.89^ABC^ | 45.47^A^ | S |
| Ki6295-4A | 66.16^ABCDE^ | 35.66^A^ | S | 69.89^ABC^ | 34.71^ABC^ | S |
| Glenlea | 49.46^MNO^ | 35.66^A^ | S | 60.38^DEFGHIJ^ | 26.43^CDE^ | MS |
| Chiba//PRLII/cM65531/3/SKAUZ/BAV92 | 69.89^ABC^ | 35.66^A^ | S | 65.22^ABCDEF^ | 42.69^A^ | S |
| Catbird | 68.97^ABCD^ | 35.66^A^ | S | 71.71^A^ | 45.47^A^ | S |
| Hoggana | 63.29^BCDEFGHIJ^ | 35.66^A^ | S | 66.16^ABCDE^ | 30.95^BCD^ | S |
| CIANO T79 | 59.40^DEFGHIJKL^ | 35.66^A^ | S | 61.36^BCDEFGHI^ | 30.95^BCD^ | S |
| Veranopolis | 48.46^MNO^ | 35.66^A^ | S | 69.89^AB^ | 42.49^A^ | S |
| Estanzuela Federal | 63.29^BCDEFGHIJ^ | 35.66^A^ | S | 69.89^ABC^ | 42.49^A^ | S |
| ZERBA-6/FLAG-6/3/TAM200/PASTOR//TOBA97 | 53.46^HIJKLMNO^ | 35.66^A^ | S | 71.71^A^ | 35.66^ABC^ | S |
| Shafir | 73.49^A^ | 30.95^A^ | S | 59.40^EFGHIJK^ | 22.98^DE^ | MS |
| Etw17-221 | 55.45^GHIJKLMNO^ | 27.32^AB^ | MS | 64.26^ABCDEFG^ | 30.95^BCD^ | S |
| WAXWING*2/CIRCUS | 57.43^EFGHIJKLM^ | 27.32^BC^ | MS | 69.89^AB^ | 30.95^BCD^ | S |
| Meraro | 50.46^LMNO^ | 27.32^BC^ | MS | 38.56^OP^ | 18.14^EF^ | MS |
| BR 34 | 65.22^ABCDEFG^ | 22.98^C^ | MS | 57.43^EFGHIJK^ | 18.14^EF^ | MS |
| Murga | 59.40^DEFGHIJKLM^ | 22.98^C^ | MS | 59.40^EFGHIJK^ | 27.32^CDE^ | MS |
| Kk4500 | 66.16^ABCDEFG^ | 22.98^C^ | MS | 62.33^ABCDEFGH^ | 18.14^EF^ | MS |
| Biqa | 56.44^EFGHIJKLM^ | 22.98^C^ | MS | 51.46^IJKLM^ | 18.14^EF^ | MS |
| Hidasie | 61.36^CDEFGHIJKL^ | 22.98^C^ | MS | 57.43^EFGHIJK^ | 26.43^CDE^ | MS |
| SOKOLL//W15.92/WBLL1 | 64.26^ABCDEFG^ | 22.98^C^ | MS | 52.46^IJKLM^ | 18.14^EF^ | MS |
| K6290 Bulk | 63.29^ABCDEFGH^ | 22.98^C^ | MS | 68.97^ABCD^ | 26.43^CDE^ | MS |
| Frontana | 57.43^EFGHIJKLM^ | 14.45^D^ | MR | 46.46^LMNO^ | 10.50^GH^ | MR |
| Etw17-85 | 52.46^IJKLMNO^ | 10.50^DE^ | MR | 53.46^HIJKLM^ | 18.14^EF^ | MS |
| Alidoro | 48.46^MNO^ | 10.50^DE^ | MR | 55.45^GHIJKLM^ | 10.50^GH^ | MR |
| Nd-495 | 68.97^ABCD^ | 10.50^DE^ | MR | 46.46^LMNO^ | 10.50^GH^ | MR |
| Madda walabu | 57.43^EFGHIJKLM^ | 10.50^DE^ | MR | 55.45^FGHIJKL^ | 10.50^GH^ | MR |
| BR 18 | 64.26^ABCDEFG^ | 10.50^DE^ | MR | 50.46^JKLM^ | 14.45^FG^ | MR |
| KM7 | 50.46^MNO^ | 10.50^DE^ | MR | 60.38^CDEFGHI^ | 14.45^FG^ | MR |
| Hulluka | 61.36^CDEFGHIJKL^ | 10.50^DE^ | MR | 39.54^NOP^ | 10.50^GH^ | MR |
| Danda’a | 67.11^ABCDE^ | 10.50^DE^ | MR | 46.46^LMNO^ | 10.50^GH^ | MR |
| Kingbird | 65.22^ABCDEFG^ | 10.50^DE^ | MR | 40.52^NOP^ | 10.50^GH^ | MR |
| ET-13A2 | 52.46^JKLMNO^ | 7.11^EF^ | MR | 49.46^KLMN^ | 14.45^FG^ | S |
| Etw17-115 | 50.46^LMNO^ | 7.11^EF^ | MR | 11.76 ^SR^ | 2.54^JK^ | R |
| Gondo | 51.46^KLMNO^ | 4.35^FG^ | R | 8.73 ^S^ | 0.25^K^ | R |
| Israel-493 | 66.16^ABCDEF^ | 4.35^FG^ | R | 28.22 ^Q^ | 2.54^JK^ | R |
| Salamouni | 47.46^NO^ | 4.35^FG^ | R | 54.45^HIJKLM^ | 7.11^HI^ | MR |
| Pavon-76 | 72.61^AB^ | 4.35^FG^ | R | 39.54 ^NOP^ | 4.76^IJ^ | MR |
| Blouk#1 | 65.22^ABCDEFG^ | 2.23^G^ | R | 13.08 ^SR^ | 1.21^JK^ | R |
| Sofumar | 53.46^HIJKLMNO^ | 2.23^G^ | R | 45.47 ^MNO^ | 2.54^JK^ | R |
| Coulter | 51.46^KLMNO^ | 2.23^G^ | R | 18.14 ^R^ | 2.54^JK^ | R |
| Mutus | 63.39^BCDEFGHI^ | 2.23^G^ | R | 35.66^PQ^ | 2.54^JK^ | R |
| Erik | 62.33^BCDEFGHIJK^ | 2.23^G^ | R | 13.76 ^SR^ | 1.21^JK^ | R |
| 6B662 | 55.45^FGHIJKLMN^ | 2.23^G^ | R | 9.99 ^S^ | 1.21 ^JK^ | R |
| R^2^ | 0.68 | 0.96 |  | 0.942163 | 0.93 |  |
| CV | 7.69 | 10.69 |  | 7.85 | 16.02 |  |
| Mean | 0.88 | 0.42 |  | 0.79 | 0.43 |  |
| LSD | 0.1096 | 0.07 |  | 0.10 | 0.11 |  |
