# Supplementary material for: Field evaluation of spring wheat genotypes reveals differential resistance to Zymoseptoria tritici in Ethiopia
Source: PLoS One. 2026 Jul 10;21(7):e0353375. doi: 10.1371/journal.pone.0353375 (PMC13353992; doi:10.1371/journal.pone.0353375)
Supplement: S2 Table — (DOCX) [file pone.0353375.s002.docx]

**Table.S2.**

| Number of cluster | Variable | Mean | Std.Dve | SEM |
| --- | --- | --- | --- | --- |
| cluster I (N=5) | Severity * | 68.16 | 1.398 | 0.625 |
|  | AUDPC | 2948.87 | 58.697 | 26.25 |
|  | Pycnidia | 4.33 | 0.204 | 0.091 |
| cluster II (N=6) | Severity | 64.75 | 1.491 | 0.609 |
|  | AUDPC | 2762.67 | 43.97 | 17.95 |
|  | Pycnidia | 3.44 | 0.43 | 0.176 |
| cluster III (N=15) | Severity | 58.42 | 1.86 | 0.480 |
|  | AUDPC | 2471.47 | 86.79 | 22.41 |
|  | Pycnidia | 3.09 | 0.89 | 0.23 |
| cluster IV (=3) | Severity | 28.95 | 1.28 | 0.737 |
|  | AUDPC | 1289.17 | 48.93 | 28.25 |
|  | Pycnidia | 0.94 | 0.35 | 0.20 |
| cluster V (N=3) | Severity | 35.72 | 1.81 | 1.04 |
|  | AUDPC | 1547.39 | 89.11 | 51.45 |
|  | Pycnidia | 0.78 | 0.096 | 0.06 |
| cluster VI (N=6) | Severity | 53.46 | 1.699 | 0.694 |
|  | AUDPC | 2216.67 | 57.34 | 23.41 |
|  | Pycnidia | 2.111 | 0.49 | 0.20 |
| cluster VII (N=2) | Severity | 45.88 | 1.745 | 1.234 |
|  | AUDPC | 1898.17 | 28.05 | 19.83 |
|  | Pycnidia | 2.083 | 1.295 | 0.92 |
| cluster VIII (N=5) | Severity | 50.013 | 0.748 | 0.33 |
|  | AUDPC | 2058 | 55.97 | 25.03 |
|  | Pycnidia | 1.5 | 0.5 | 0.224 |

*= Severity result scored from pycnidia bearing necrosis**Mean= cluster centroid, Std.Dve.=standard deviation, SEM= Standard Error of the Mean, Roman numbers from I to VIII = cluster numbers, N=number of genotype in that cluster,
