# Supplementary material for: Field evaluation of spring wheat genotypes reveals differential resistance to Zymoseptoria tritici in Ethiopia
Source: PLoS One. 2026 Jul 10;21(7):e0353375. doi: 10.1371/journal.pone.0353375 (PMC13353992; doi:10.1371/journal.pone.0353375)
Supplement: S3 Table — (DOCX) [file pone.0353375.s003.docx]

**Table S3.**

| Components | PC1 | PC2 | PC3 | PC4 | PC5 | PC6 | PC7 | PC8 | PC9 |
| --- | --- | --- | --- | --- | --- | --- | --- | --- | --- |
| eigenvalue | 3.817 | 1.702 | 1.024 | 0.743 | 0.669 | 0.499 | 0.341 | 0.201 | 0.003 |
| Standard deviation | 1.95 | 1.30 | 1.01 | 0.86 | 0.82 | 0.71 | 0.58 | 0.45 | 0.06 |
| Proportion of Variance | 0.42 | 0.19 | 0.11 | 0.08 | 0.07 | 0.06 | 0.04 | 0.02 | 0.00 |
| Cumulative Proportion | 0.42 | 0.61 | 0.73 | 0.81 | 0.88 | 0.94 | 0.98 | 0.99 | 1.00 |
