# Supplementary figures and images for: Field evaluation of spring wheat genotypes reveals differential resistance to Zymoseptoria tritici in Ethiopia
Source: PLoS One. 2026 Jul 10;21(7):e0353375. doi: 10.1371/journal.pone.0353375 (PMC13353992; doi:10.1371/journal.pone.0353375)

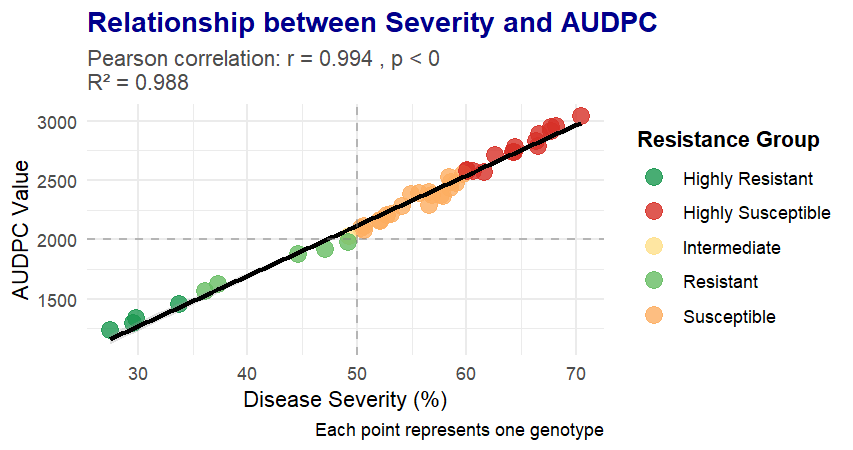


**Fig.S1.**

Supplement: S1 Fig — A strong positive linear relationship was observed (r = 0.994, R2 = 0.988). Data points are color-coded by resistance group, showing distinct clustering of highly resistant genotypes in the lower-left and highly susceptible genotypes in the upper-right of the plot. (DOCX) [file pone.0353375.s004.docx]
