# Supplementary material for: Field evaluation of spring wheat genotypes reveals differential resistance to Zymoseptoria tritici in Ethiopia
Source: PLoS One. 2026 Jul 10;21(7):e0353375. doi: 10.1371/journal.pone.0353375 (PMC13353992; doi:10.1371/journal.pone.0353375)
Supplement: Attachment — Submitted filename: 2023 IndianPhytopathol disease-score.pdf [file pone.0353375.s005.pdf]

Indian Phytopathology
https://doi.org/10.1007/s42360-023-00663-4

SHORT COMMUNICATION

Revisiting the use of disease index and of disease scores in plant pathology

L. Willocquet[1,2] · S. Savary[2,3] · K. P. Singh[3]

Received: 6 April 2023 / Revised: 16 July 2023 / Accepted: 23 July 2023
© Indian Phytopathological Society 2023

Abstract
Plant disease assessment often involves the use of rating scores e.g., "0, 1, 2, … 6", to rapidly record disease severity. Scores are ordinal, but qualitative, variables. The analysis of scores with parametric statistical methods will unavoidably lead to confusion and error. By the same token, using disease scores to calculate sum, mean, or derived indices such as a disease index, is incorrect. The proper analysis of disease data recorded as scores depend on the disease assessment key used to define the disease scores. If disease scores are categorical variables describing symptom attributes, they should be analysed using non-parametric methods for qualitative variables. If disease scores represent disease severity as the fraction of the diseased area, there are two ways for analysis. First, if disease scores correspond to a pre-set number of disease severity values, scores should be back-transformed to these severity values. But if disease scores correspond to successive severity intervals, scores should be converted into severities at the mid-range of these intervals. In both latter cases, the new variables derived from these transformations are continuous, quantitative variables. They may therefore be analysed with parametric methods. Numerical examples are provided to illustrate the outcomes of different ways to process disease rating score data.

Keywords Data analysis · Disease assessment · Disease index · Disease rating · Disease severity · Resistance screening

Introduction

Disease assessment is a necessary step in any study addressing plant disease. Surprisingly, the importance of this step is sometimes overlooked and incorrectly implemented, perhaps because it is perceived as a routine, and is remote from the main focus of the analysis undertaken. Disease assessment is conducted in laboratory, greenhouse, or field experiments as well as in surveys in farmers' fields (or orchards or any other plant populations) for a range of studies, including epidemiological, crop loss, and disease management (Bock et al. 2010; Campbell and Madden 1990). Disease assessment can also be used for strategic decisions in public and private institutions, for example in disease surveillance programmes and in breeding resistant varieties (Chaube and Singh 1991).

Two seminal publications by Large (1966) and James (1971) established the principles and methodology of plant disease assessment. The methodology has been continuously described and expanded in plant pathology-related text books, for example in Zadoks and Schein (1979), Campbell and Madden (1990), Chaube and Singh (1991), Teng and James (2002), Madden et al. (2007), and Munk et al. (2020), as well as in numerous articles, including Nutter et al (2006) and Bock et al (2010). The processing of disease assessment information depends on the nature of the information collected (Campbell and Madden 1990; Madden et al. 2007; Schabenberger and Pierce 2002). Figure 1 provides an overview of the main steps involved in disease assessment and data analyses.

Disease assessment often aims to estimate disease severity. Disease severity is the proportion of diseased tissues (e.g., Campbell and Madden 1990). Disease severity assessment can be applied to any plant tissue, whether root, stem, tuber, or fruit. However, most studies have concerned foliar tissues. On plant foliage, disease severity refers to the fraction of leaf area showing symptoms of

✉ K. P. Singh
kpsingh.gbpuat@gmail.com

[1] Département Santé des Plantes et Environnement, INRAE, Toulouse, France

[2] University of California-Davis, Davis, USA

[3] Department of Plant Pathology, College of Agriculture, G. B. Pant University of Agriculture and Technology, Pantnagar, India

Published online: 21 August 2023

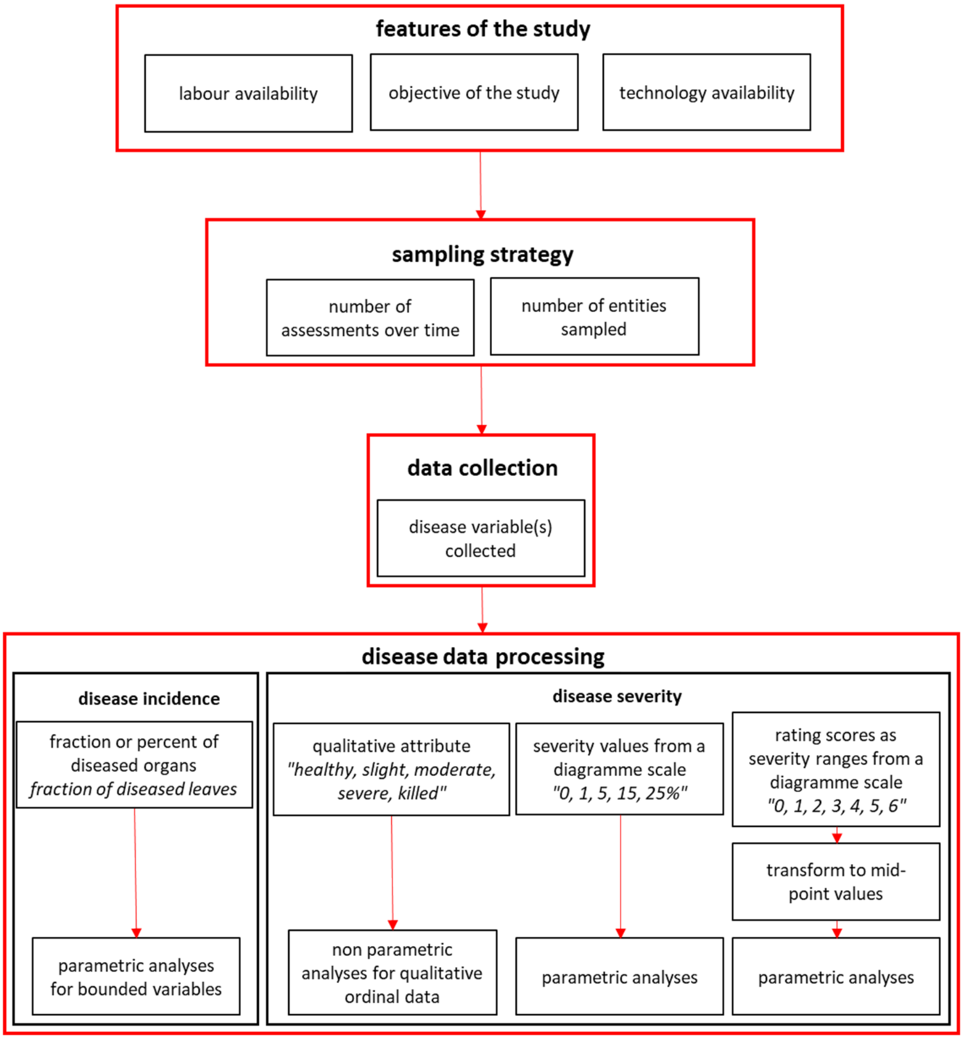

Fig. 1 Overview of steps involved in disease assessment and data analysis. Note that the disease index is not included in the overview, because of issues associated with its use (see text for details). Text in italic represents examples provided for illustration

diseases caused by fungi, oomycetes, or bacteria. "Disease severity" has also been used to express the intensity of symptoms at the plant scale in the case of systemic diseases such as viral diseases (Campbell and Madden 1990). Disease severity is usually assessed along a pre-set, limited number of classes which are described in assessment keys (Large 1966). Such keys may be expressed in a number of forms (Zadoks and Schein 1979), the most familiar one being diagrammatic keys or standard diagrams (James 1971). Standard diagrams can be developed as drawings representing each class, allowing one to assign the observed disease severity to a given class matching best the observation. Disease severity classes are usually recorded as scores, or grades, such as (0, 1, 2, …, 6), in order to save time when recording and encoding data. These scores therefore must be seen as short-hand summaries of the severity levels observed on each plant tissue sample. Figure 2 (Savary 1986) provides an example of a standard diagram with classes from 0 to 6, developed to assess groundnut rust severity on leaflets.

Processing of such data requires attention and is sometimes incorrectly conducted (Bock et al. 2010; Campbell and Madden 1990; Chiang et al. 2017; Madden et al. 2007; Schabenberger and Pierce 2002; Shah and Madden 2004).

The objective of this article is to discuss the methodological aspects pertaining to the processing of disease severity data prior to analyses. The case of disease rating scores and the derived disease index is discussed in detail and illustrated with a numerical example.

Disease severity as a qualitative ordinal variable

This type of variable is often generated from assessments of systemic diseases such as viral diseases, where the disease is assessed according to assessment keys reflecting symptoms at the whole plant scale with ordered (ranked, increasing) intensity, such as “healthy, slight, moderate, severe, killed”. Assessment keys used to assess the level of host plant resistance in field trials very often generate similar qualitative ordinal variables such as “highly susceptible, susceptible,

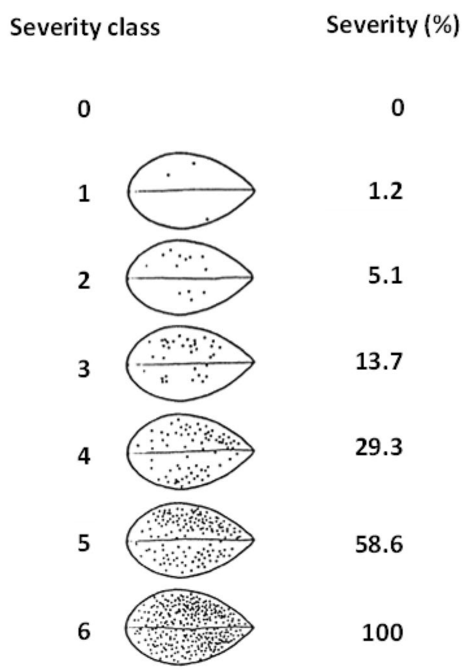

Fig. 2 Standard area diagram for groundnut rust severity assessment, from Savary (1986). 100% severity is the maximum disease which can be sustained by a leaflet, and corresponds to 30% of area covered by pustules

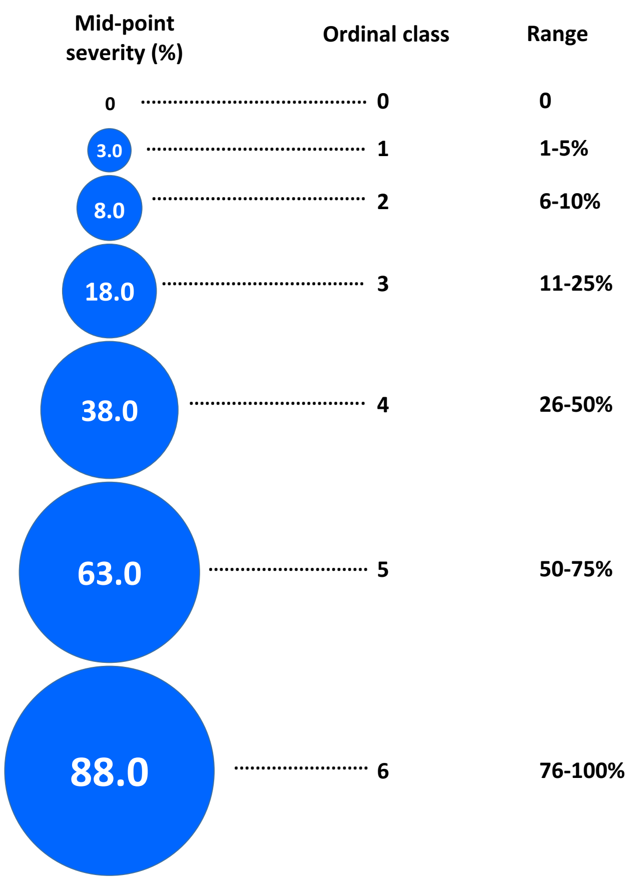

Fig. 3 Example of a seven-point quantitative ordinal disease scale (0–6) with associated mid-point percentage disease severity (0; 3.0; …; 88.0%) and disease severity ranges. Areas of circles are proportional to the mid-point severity

moderately susceptible … highly resistant" (Zadoks and Schein 1979).

Integer values are often attached to these qualitative classes (e.g., 0, 1 … 4) in order to save time for data recording. The resulting variable, despite being expressed as (integer) numbers, cannot be analysed as if it were quantitative. As stated by Shah and Madden (2004) "*Parametric methods of analysis using statistics based on means, or differences between means (such as ANOVA), are thus, strictly speaking, inappropriate for analysing data on an ordinal scale, though they are used quite often in many disciplines*". Such qualitative, categorical variables should be analysed as such with non-parametric statistical methods (see, e.g., Agresti 2002; McCool et al. 1986; Shah and Madden 2007), including multivariate analyses (Legendre and Legendre 2012; Savary et al. 1995).

Disease severity as fraction of diseased area

Disease assessment scales based on classes of increasing severity (e.g., James 1971) are widely used to rapidly assess disease severity as the fraction of diseased area through visual observation. The scales in general assign scores (for example 0, 1, …, x) to classes of increasing severity. A given disease severity score may correspond to two types of information: a central severity value (as, e.g., in Fig. 2), or a severity interval within boundaries. Both cases are examined below.

- In the case of scores representing central severity values, classes are defined according to standard diagrams and to a set of values (e.g., 1, 5, 15, 25%), which are central-class values. Such keys, as illustrated in Fig. 2, have been developed for example by James (1971) for diseases of monocots and dicots, and by Peterson et al. (1948) for cereal rusts. Central disease severity values are discrete, but quantitative values. In this case, therefore, disease scores (e.g., 0, 1, …, x) need to be back-transformed into their central disease severity values, in order to convert scores in severity. The resulting severity data may then be subjected to parametric analyses such as means and analyses of variance.
- In the case of scores representing severity intervals, we use the example of a 0—6 scale (with 7 rating scores) corresponding to severity ranges of: 0; [1–5]; [6–10]; [11–25]; [26–50]; [51–75]; and [76–100%] (Fig. 3). As recommended in some instructions to Authors (e.g.,

information for Authors of the APS journals; https://apsjournals.apsnet.org/page/authorinformation#statistics), as discussed in several articles or textbooks (e.g., Bock et al. 2010), and as quantitatively documented by Chiang et al. (2017), each score should be converted to the mid-point (central-class) of the corresponding disease severity range before using parametric analyses.

Rating scores (i.e., 0, 1, …, x) are sometimes directly used to compute means and other statistics over replicates, or to conduct parametric analyses such as analyses of variance. This approach is incorrect and leads to wrong results. As stated in Campbell and Madden (1990, pp. 117–118), "*When class values are recorded for assessments of disease severity and percentage disease is the desired variable, it is inappropriate to average the class values. Biased results would occur*". Computing the mean of rating scores would be correct only in the case when all severity classes have exactly the same range (e.g., 0–10; 11–20 … 91–100%), which is nearly never the case.

Table 1 numerically illustrates the relationships between mean disease scores and means over disease severity mid-points (central-class values) in three examples, using the 7-class scale of Fig. 3. In each example, disease records for a sample of 10 entities (e.g., leaves) are shown. The examples are chosen to exemplify three cases with the same average disease score, that is, 2. In the first example, disease occurs uniformly with a score of 2. In the second example, disease occurs at varying levels over the 10 samples, with scores ranging from 0 to 4. In the third example, disease is highly aggregated, with many samples healthy (score 0), and few heavily diseased (score 6). The average score value in all examples is 2, corresponding to a mid-point severity of 8%. If scores are expressed as they should, i.e., as mid-point severities, the resulting mean severities widely differ among the three examples: 8, 13, and 27%, respectively (last row of Table 1). Computing a mean over disease scores thus leads to under-estimated disease severity, and this under-estimation increases with disease aggregation.

Table 1 Three examples of disease severity assessment on ten samples according to a (0–6) ordinal disease scale, derived estimates of disease severity

Sample	Example 1	Example 2	Example 3
1	2	1	0
2	2	2	0
3	2	1	0
4	2	0	6
5	2	3	0
6	2	3	0
7	2	4	6
8	2	0	0
9	2	4	2
10	2	2	6
Mean over classes	2	2	2
Severity of mean over classes	8%	8%	8%
Disease index	33%	33%	33%
Mean over mid-point severities	8%	13%	27%

Areas of circles are proportional to the mid-point severity

Disease severity expressed as a disease index

The concept of disease index has been widely used to produce a synthetic, quantitative estimate of disease intensity. The disease index was also termed "infection rating" (IR) by McKinney (1923) for underground tissues of wheat plants inoculated with *Cochliobolus sativus*. IR was computed as:

$$IR = 100 \times \frac{\text{sum of all numerical ratings}}{\text{total number of observations} \times \text{maximum rating}} \quad (1)$$

where values of 0, 0.75, 1, 2, and 3, were assigned to "degrees of infection" of underground parts described as "none", "very slight", "slight", "moderate", and "abundant", respectively.

IR as expressed in Eq. 1 above, or with slight modifications, has been referred to as "disease index" (or "severity index", or "disease severity index") in several textbooks (e.g., Wheeler 1969; Chaube and Singh 1991), and is still often used to process disease data. The disease index (DI)

has been derived from scores defined according to two types of assessment keys: from qualitative assessments based on symptoms ranked along scales (as described above for disease severity as a qualitative ordinal variable), or from keys based on successive severity intervals (as described above for severity as a fraction of diseased area).

DI is generally expressed as a weighted average of the form (Chester 1950):

$$DI = 100 \times \frac{\text{sum of (number of entities assessed as disease score i} \times \text{disease score i)}}{\text{total number of observations} \times \text{maximum disease score}} \quad (2)$$

$$DI = 100 \times \sum_{i=1}^{K} (n_i \times S_i)/(N \times maxS) \quad (3)$$

where i is the ith disease score ($i = 1$ to 7 in the above example); K is the number of classes in the disease scale (7 in the example above); n_i is the number of entities scored as S_i; S_i is the score value (typically integers from 0 to x: 0 to 6 in our example); N is the total number of plant entities assessed (e.g., leaves; $N = 10$ in our example); and $maxS$ is the maximum disease score (6 in our example).

DI can also be written as:

$$DI = \frac{100}{maxS} \times \sum_{j=1}^{N} (S_j)/N \quad (4)$$

where $j = 1$ to N is the jth entity assessed; S_j is the score of entity j assessed; and N and $maxS$ as above.

In other words:

$DI = 100 \times$ mean of disease scores/maximum disease score (see Eqs. 1 and 4).

DI is, therefore, the mean of scores over replicates, expressed as a per cent of the maximum score.

If disease grades are derived from qualitative assessment keys, as in the example from McKinney (1923), it is incorrect to compute disease indices, because, as stated above, grades derived from qualitative variables cannot be summed or averaged.

If disease grades are derived from assessment keys representing successive severity intervals, it again is incorrect to derive a disease index: as stated above, calculating a mean from grades derived from severity intervals is not correct.

We provide below a numerical illustration using the examples displayed in Table 1.

DI can be computed for examples 1, 2, and 3 using Eqs. 2 and 3 (Table 1):

Example 1: DI = 100 × (10 × 2)/ (10 × 6) = 33.3%

Example 2: DI = 100 × [(2 × 0) + (2 × 1) + (2 × 2) + (2 × 3)] + (2 × 4)]/(10 × 6) = 33.3%

Example 3: DI = 100 × [(6 × 0) + (1 × 2) + (3 × 6)]/ (10 × 6) = 33.3%

DI can also be computed for examples 1, 2, and 3 using Eqs. 1 and 4 (Table 1):

Example 1: DI = (100/6) × [(2 + 2 + 2 + 2 + 2 + 2 + 2 + 2 + 2 + 2)/10] = 33.3%

Example 2: DI = (100/6) × [(1 + 2 + 1 + 0 + 3 + 3 + 4 + 0 + 4 + 2)/10] = 33.3%

Example 3: DI = (100/6) × [(0 + 0 + 0 + 6 + 0 + 0 + 6 + 0 + 2 + 6)/10] = 33.3%

The examples above and in Table 1 numerically illustrate the fact that disease indices, if reported as weighed ratios (Eqs. 2 and 3), are actually averages of disease scores, expressed as per cent of a maximum score (Eqs. 1 and 4). DI is 33.3% for all three examples, which is the average mean score (that is, 2), corrected by the maximum score (that is, divided by 6), and represented as per cent (that is, multiplied by 100).

As this example shows, the disease index most often generates an over-estimate of the true severity mean: DI is 33% in all cases, whereas the mean of mid-point severity ranges from 8 to 27%. Furthermore, DI under-estimates disease severity all the more as the samples include higher grades: DI is the same for all three examples, whereas disease severity increases from example 1 (with only scores equalling 2) to example 3 (including scores equalling 6). Such biases have been analysed in detail by Chiang et al. (2017). Considering the inaccuracy of estimates generated by the use of disease index, and the inappropriateness of the approach consisting in calculating the mean of class values, the use of disease index for ordinal scale data is therefore an incorrect approach to report disease severity.

Conclusion

Visual disease assessment using disease score scales remain the standard procedure for disease assessment. A solid, scientific, and published background exists to guide sound methods for assessment and further analysis of the disease information collected.

Disease severity scores cannot be summed, averaged, or converted into disease indices: this is incorrect and leads to incorrect results. The proper analysis of disease severity data depends on the disease assessment key used (Fig. 1):

- Disease severity scores derived from categorical ordinal scales should be analysed using methods available to analyse categorical variables.

- Disease severity scores based on pre-set severity values should be back- transformed to the initial disease severity values, which can in turn be analysed with parametric methods adapted to bounded continuous variables.
- Disease severity scores based on intervals for which severity ranges are not equal should be converted to the mid-point values of the corresponding disease severity range. The computed values can then be analysed with parametric methods adapted to boundary continuous variables.

Author contributions All authors have equally contributed to the data collection and preparation of final manuscript.

Data availability statement Not applicable.

Declarations

Conflict of interest Authors declare that there is no conflict interest.

Ethical approval This article does not contain any studies with human participants or animals performed by the author.

Consent for publication All authors have given their consent for publication of the article in *Indian Phytopathology*.

References

Agresti A (2002) Categorial data analysis, 2nd edn. Wiley, Hoboken

Bock CH, Poole GH, Parker PE, Gottwald TR (2010) Plant disease severity estimated visually, by digital photography and Image analysis, and by hyperspectral imaging. Crit Rev Plant Sci 29:59–107

Campbell LC, Madden LV (1990) Introduction to plant disease epidemiology. Wiley, New York

Chaube HS, Singh US (1991) Plant disease management: principles and practices. CRC Press, Boca Raton

Chester KS (1950) Plant disease losses: their appraisal and interpretation. Plant Dis Rep Suppl 193:190–362

Chiang KS, Liu HI, Bock CH (2017) A discussion on disease severity index values. Part I: warning on inherent errors and suggestions to maximise accuracy. Ann Appl Biol 171:139–154

James WC (1971) An illustrated series of assessment keys for plant diseases, their preparation and usage. Can Plant Dis Surv 51:39–65

Large EC (1966) Measuring plant disease. Annu Rev Phytopathol 4:9–28

Legendre P, Legendre L (2012) Numerical ecology, 3rd English ed. Elsevier, Amsterdam

Madden LV, Hughes G, van den Bosch F (2007) The study of plant disease epidemics. APS Press, St Paul

McCool PM, Younglove T, Musselman RC, Teso RR (1986) Plant injury analysis: contingency tables as an alternative to analyses of variance. Plant Dis 70:357–360

McKinney HH (1923) Influence of soil temperature and moisture on infection of wheat seedlings by *Helminthosporium sativum*. J Agric Res 26:195–218

McRoberts N, Hughes G, Madden LV (2003) The theoretical basis and practical application of relationships between different disease intensity measurements in plants. Ann Appl Biol 142:191–211

Munk L, Djurle A, Yuen J (2020) From disease assessment to decision support systems. In: Tronsmo AM, Collinge DB, Djurle A, Munk L, Yuen J, Tronsmo A (eds) Plant pathology and plant diseases. CAB International, Wallingford, pp 330–356

Nutter FW, Esker PD, Netto RAC (2006) Disease assessment concepts and the advancements made in improving the accuracy and precision of plant disease data. Eur J Plant Pathol 115:95–103

Peterson RF, Campbell AB, Hannah AE (1948) A diagrammatic scale for estimating rust intensity on leaves and stems of cereals. Can J Res (c) 26:496–500

Savary S, Madden LV, Zadoks JC, Klein-Gebbinck HW (1995) Use of categorical information and correspondence analysis in plant disease epidemiology. Adv Bot Res 21:213–240

Savary S (1986) Epidemiological Studies on Groundnut Rust in Côte d'Ivoire- Etudes épidémiologiques sur la rouille de l'arachide en Côte d'Ivoire. PhD thesis. Landbouwuniversiteit te Wageningen, The Netherlands

Schabenberger O, Pierce FJ (2002) Contemporary statistical models for the plant and soil sciences. Taylor & Francis, London

Seem RC (1984) Plant disease incidence and severity relationships. Annu Rev Phytopathol 22:137–150

Shah DA, Madden LV (2004) Nonparametric analysis of ordinal data in designed factorial experiments. Phytopathology 94:33–43

Teng PS, James WC (2002) Disease and yield loss assessment. In: Waller JM, Lenné JM, Waller SJ (eds) Plant pathologist's pocketbook. CAB International, Wallingford, pp 25–38

Wheeler BEJ (1969) An introduction to plant diseases. An introduction to plant diseases. Wiley, London

Zadoks JC, Schein RD (1979) Epidemiology and plant disease management. Oxford University Press, New York

Publisher's Note Springer Nature remains neutral with regard to jurisdictional claims in published maps and institutional affiliations.
