# Supplementary material for: Field evaluation of spring wheat genotypes reveals differential resistance to Zymoseptoria tritici in Ethiopia
Source: PLoS One. 2026 Jul 10;21(7):e0353375. doi: 10.1371/journal.pone.0353375 (PMC13353992; doi:10.1371/journal.pone.0353375)
Supplement: Attachment — Submitted filename: PONE-D-25-41622. rev.pdf [file pone.0353375.s006.pdf]

PLOS ONE

Field Evaluation of Bread Wheat (Triticum aestivum L.) Genotypes Reveals Differential Resistance to Zymoseptoria tritici Blotch at Holetta, Ethiopia

--Manuscript Draft--

**Manuscript Number:**	PONE-D-25-41622
**Article Type:**	Research Article
**Full Title:**	Field Evaluation of Bread Wheat (Triticum aestivum L.) Genotypes Reveals Differential Resistance to Zymoseptoria tritici Blotch at Holetta, Ethiopia
**Short Title:**	Field Evaluation of Bread Wheat (Triticum aestivum L.) Reveals Differential Resistance to Zymoseptoria tritici
**Corresponding Author:**	Habtewold Kifelew Abebe, MS.C<br>Ethiopian Institute of Agricultural Research<br>Addis Ababa, Addis ababa ETHIOPIA
**Keywords:**	Cluster, Differentials, gene postulation, Ethiopia
**Abstract:**	Zymoseptoria tritici blotch is a significant disease affecting wheat (Triticum aestivum L.) in Ethiopia and various other countries worldwide. This study evaluated 45 bread wheat genotypes for their resistance to Z. tritici during the adult plant stage under natural infection conditions. The field trial was conducted at the Holeta Agricultural Research Center in Ethiopia over two consecutive years, 2022 and 2023, utilizing a randomized complete block design with three replications. Disease assessments were carried out using a double-digit scale ranging from 00 to 99. A comprehensive analysis of variance performed over the two years indicated statistically significant differences ($P \leq 0.01$) among the years, genotypes, and the interaction between genotype and year. The severity of the disease varied from 29.52 to 70.44, while pycnidia coverage ranged from 1 to 4.5. Four distinct disease reactions were identified: 24.4% and 25.2% of the genotypes were classified as resistant, 30.4% and 24.4% as moderately resistant, 22.2% and 20.7% as moderately susceptible, and 22.96% and 29.63% as susceptible during 2022 and 2023, respectively. The cultivar Catbird exhibited the highest level of susceptibility, while the cultivar ETW17-115 demonstrated the strongest resistance. A detailed analysis of the dendrogram revealed four clusters, with Cluster VI comprising six genotypes that displayed a resistant response. Notably, this cluster did not include any differential lines for Z. tritici resistance, indicating that the resistance genes present in these genotypes require molecular analysis and further investigation. Principal component analysis revealed that the cultivars "Gondo, 6B662, ETW17-115, Coulter, Erik, and Blouk#1" exhibited significant negative coefficients, suggesting that this component primarily evaluates resistance levels. In contrast, the cultivars “Tadina, Chiba//PRLII/cM65531/3/SKAUZ/BAV92, and WAXWING*2/CIRCUS” exhibited notably positive coefficients, indicating that this component primarily reflects genotype vulnerability to the disease. These findings provide valuable insights for breeding programs aimed at enhancing wheat resistance to Z. tritici.
**Order of Authors:**	Habtewold Kifelew Abebe, MS.C
	Habtamu Terefe terefe, Ph.D.
	Bekele Kasa, Ph.D.
	Tilahun Mekonen, Ph.D.
	Zelalem Bekeko, Ph.D.
	Bulti Tesso, Ph.D.
**Additional Information:**	
**Question**	**Response**
**Financial Disclosure**	The author(s) received no specific funding for this work.

Powered by Editorial Manager® and ProduXion Manager® from Aries Systems Corporation

Enter a financial disclosure statement that describes the sources of funding for the work included in this submission. Review the submission guidelines for detailed requirements. View published research articles from *PLOS ONE* for specific examples.<br><br>This statement is required for submission and **will appear in the published article** if the submission is accepted. Please make sure it is accurate.<br><br>**Funded studies**<br>Enter a statement with the following details:<br>• Initials of the authors who received each award<br>• Grant numbers awarded to each author<br>• The full name of each funder<br>• URL of each funder website<br>• Did the sponsors or funders play any role in the study design, data collection and analysis, decision to publish, or preparation of the manuscript?<br><br>Did you receive funding for this work?	
**Competing Interests**<br><br>Use the instructions below to enter a competing interest statement for this submission. On behalf of all authors, disclose any competing interests that could be perceived to bias this work—acknowledging all financial support and any other relevant financial or non-financial competing interests.<br><br>This statement is required for submission and **will appear in the published article** if the submission is accepted. Please make sure it is accurate and that any funding sources listed in your Funding Information later in the submission form are also declared in your Financial Disclosure statement.<br><br>View published research articles from	The authors declare that they have no conflict of interest.

PLOS ONE for specific examples.<br><br>**NO authors have competing interests**<br><br>Enter: *The authors have declared that no competing interests exist.*<br><br>**Authors with competing interests**<br><br>Enter competing interest details beginning with this statement:<br><br>*I have read the journal's policy and the authors of this manuscript have the following competing interests: [insert competing interests here]*<br><br>* typeset	
**Ethics Statement**<br><br>Enter an ethics statement for this submission. This statement is required if the study involved:<br><br>• Human participants<br>• Human specimens or tissue<br>• Vertebrate animals or cephalopods<br>• Vertebrate embryos or tissues<br>• Field research<br><br>Write "N/A" if the submission does not require an ethics statement.<br><br>General guidance is provided below. Consult the submission guidelines for detailed instructions. **Make sure that all information entered here is included in the Methods section of the manuscript.**	The study was approved by review and Ethics Committee, Harumaya University, Agriculture and Environmental Sciences School of Plant Sciences in February, 2023.

Powered by Editorial Manager® and ProduXion Manager® from Aries Systems Corporation

**Format for specific study types**<br><br>**Human Subject Research (involving human participants and/or tissue)**<br>• Give the name of the institutional review board or ethics committee that approved the study<br>• Include the approval number and/or a statement indicating approval of this research<br>• Indicate the form of consent obtained (written/oral) or the reason that consent was not obtained (e.g. the data were analyzed anonymously)<br><br>**Animal Research (involving vertebrate animals, embryos or tissues)**<br>• Provide the name of the Institutional Animal Care and Use Committee (IACUC) or other relevant ethics board that reviewed the study protocol, and indicate whether they approved this research or granted a formal waiver of ethical approval<br>• Include an approval number if one was obtained<br>• If the study involved *non-human primates*, add *additional details* about animal welfare and steps taken to ameliorate suffering<br>• If anesthesia, euthanasia, or any kind of animal sacrifice is part of the study, include briefly which substances and/or methods were applied<br><br>**Field Research**<br><br>Include the following details if this study involves the collection of plant, animal, or other materials from a natural setting:<br>• Field permit number<br>• Name of the institution or relevant body that granted permission	
**Data Availability**<br><br>Authors are required to make all data underlying the findings described fully available, without restriction, and from the time of publication. PLOS allows rare exceptions to address legal and ethical concerns. See the PLOS Data Policy and FAQ for detailed information.	Yes - all data are fully available without restriction

A Data Availability Statement describing where the data can be found is required at submission. Your answers to this question constitute the Data Availability Statement and **will be published in the article**, if accepted.<br><br>**Important:** Stating 'data available on request from the author' is not sufficient. If your data are only available upon request, select 'No' for the first question and explain your exceptional situation in the text box.<br><br>Do the authors confirm that all data underlying the findings described in their manuscript are fully available without restriction?	
**Describe where the data may be found in full sentences. If you are copying our sample text, replace any instances of XXX with the appropriate details.**<br><br>• If the data are **held or will be held in a public repository**, include URLs, accession numbers or DOIs. If this information will only be available after acceptance, indicate this by ticking the box below. For example: *All XXX files are available from the XXX database (accession number(s) XXX, XXX.).*<br>• If the data are all contained **within the manuscript and/or Supporting Information files**, enter the following: *All relevant data are within the manuscript and its Supporting Information files.*<br>• If neither of these applies but you are able to provide **details of access elsewhere**, with or without limitations, please do so. For example:<br><br>*Data cannot be shared publicly because of [XXX]. Data are available from the XXX Institutional Data Access / Ethics Committee (contact via XXX) for researchers who meet the criteria for access to confidential data.*<br><br>*The data underlying the results presented in the study are available from (include the name of the third party*	The data is available at the coorosponding author and deliver upon request

Powered by Editorial Manager® and ProduXion Manager® from Aries Systems Corporation

and contact information or URL).<br>• This text is appropriate if the data are owned by a third party and authors do not have permission to share the data.<br><br>* typeset	
Additional data availability information:	

Powered by Editorial Manager® and ProduXion Manager® from Aries Systems Corporation

Manuscript

Click here to access/download;Manuscript;Habtewold article on wheat screening for plos one.docx

Field Evaluation of Bread Wheat *(Triticum aestivum L.)* Genotypes Reveals Differential Resistance to *Zymoseptoria tritici* Blotch at Holetta, Ethiopia

Habtewold Kifelew[1], Habtamu Terefe[2], Bekele Kasa [1], Tilahun Mekonen [3], Zelalem Bekeko [2] and Bulti Teso [2]

[1] Holeta Agricultural Research Center, P.O.Box 31/2003, Holeta, Ethiopia
[2] Haramaya University, College of Agriculture and Environmental Sciences, P.O. Box 138, Dire Dawa, Ethiopia,
[3] Addis Ababa University, Institute of Biotechnology, P.O. Box 1176, Addis Ababa, Ethiopia

Corresponding author: Habtewold Kifelew, (kifelew@yahoo.com) Telephone: +251913312650

Abstract

*Zymoseptoria tritici blotch is a significant disease affecting wheat (Triticum aestivum L.) in Ethiopia and various other countries worldwide. This study evaluated 45 bread wheat genotypes for their resistance to Z. tritici during the adult plant stage under natural infection conditions. The field trial was conducted at the Holeta Agricultural Research Center in Ethiopia over two consecutive years, 2022 and 2023, utilizing a randomized complete block design with three replications. Disease assessments were carried out using a double-digit scale ranging from 00 to 99. A comprehensive analysis of variance performed over the two years indicated statistically significant differences (P ≤ 0.01) among the years, genotypes, and the interaction between genotype and year. The severity of the disease varied from 29.52 to 70.44, while pycnidia coverage ranged from 1 to 4.5. Four distinct disease reactions were identified: 24.4% and 25.2% of the genotypes were classified as resistant, 30.4% and 24.4% as moderately resistant, 22.2% and 20.7% as moderately susceptible, and 22.96% and 29.63% as susceptible during 2022 and 2023, respectively. The cultivar Catbird exhibited the highest level of susceptibility, while the cultivar ETW17-115 demonstrated the strongest resistance. A detailed analysis of the dendrogram revealed four clusters, with Cluster VI comprising six genotypes that displayed a resistant response. Notably, this cluster did not include any differential lines for Z. tritici resistance, indicating that the resistance genes present in these genotypes require molecular analysis and further investigation. Principal component analysis revealed that the cultivars "Gondo, 6B662, ETW17-115, Coulter, Erik, and Blouk#1" exhibited significant negative coefficients, suggesting that this component primarily evaluates resistance levels. In contrast, the cultivars "Tadina, Chiba//PRLII/cM65531/3/SKAUZ/BAV92, and WAXWING*2/CIRCUS" exhibited notably positive coefficients, indicating that this component primarily reflects genotype vulnerability to the disease. These findings provide valuable insights for breeding programs aimed at enhancing wheat resistance to Z. tritici.*

Key word: Cluster, Differentials, gene postulation, Ethiopia

Introduction

Wheat is the primary cereal crop in Ethiopia, yielding 4,925,000 metric tons with an average productivity of 2.73 tons per hectare, cultivated by 4,212,518 smallholder farmers across 1.8 million hectares (CSA, 2021). The country aims to reduce wheat imports by boosting domestic production, targeting an increase in yield from 2.7 tons per hectare in 2019 to 4 tons per hectare by 2023 (Getachew, 2020). However, Ethiopia's wheat production and productivity remain low compared to Egypt 6.4 tons/ha (USDA, 2023). The low productivity is linked to various biotic and abiotic challenges, as well as limited adoption of modern agricultural technologies (Tesfaye *et al*., 2001). In terms of biotic stress, stem and leaf rust are prevalent in low and mid-altitude regions, while stripe rust (Yellow rust) and Septoria are more common in high-altitude wheat-growing areas of Ethiopia (Mengistu *et al*., 1991; Endale and Getaneh, 2015; Yitagesu *et al*., 2019; Meyer *et al*., 2021).

Particularly in the highlands, *Zymoseptoria tritici* is among the most devastating diseases of wheat in Ethiopia, ranking second only to stripe rust. This pathogen is responsible for yield losses that range from 41% (Alemar & Temam, 2016) to 82% (Mengistu *et al*., 1991; Endale & Getaneh, 2015). In addition to lowering productivity, *Z. tritici* infection degrades grain quality (Bockus *et al*., 2010). Due to frequent *Z. tritici* outbreaks and a lack of effective disease management strategies, wheat production in the area has recently become challenging.

Resistance to *Z. tritici* is primarily quantitative under field conditions (Brown *et al*., 2015; Dreisigacker *et al*., 2015). However, 18 qualitative resistance genes (*Stb1–Stb18*) have been identified through field trials and genotype-isolate interaction studies (Tabib *et al*., 2011, 2012;

Raman & Milgate, 2012). Furthermore, the wheat genome contains a large number of quantitative trait loci (QTL) linked to resistance (Eriksen *et al*., 2003; Chartrain *et al*., 2004; Simon *et al*., 2004). Since 2015, there have been findings indicating that 89 chromosomal regions harbor QTL associated with resistance to *Z. tritici* (Brown *et al*., 2015). Studies in Ethiopia have revealed significant variability in resistance among bread wheat, durum wheat, and triticale (Kema *et al*., 1996). Eyal and his team (1973, 1985) investigated the virulence traits of 97 *Z. tritici* isolates obtained from 22 countries, including Ethiopia, by evaluating them on 35 varieties of wheat and triticale. Their findings confirmed isolate-cultivar specificity, indicating diverse virulence genes in the pathogen population. Molecular analyses by Tilahun *et al*. (2020) further demonstrated substantial genetic variability in resistance among 180 Ethiopian bread wheat genotypes. Screening programs in Tigray (Teklay *et al*., 2015), Gondar (Yohannes *et al*., 2020), and Holeta (Bekele & Yitagesu, 2019) have also contributed to identifying resistant genotypes.

As of now, the evaluation of wheat genotypes for their resistance to *Z. tritici* has been conducted under field conditions, using a susceptible check as a control. However, it is essential to evaluate genotypes that include differential lines to identify the specific race against which the genotype exhibits resistance and to potentially facilitate gene postulation. Assessing the response of wheat genotypes to *Z. tritici* and speculating on potential genes present in the wheat genotypes were the goals of this study.

Materials and Methods

Description of the Study Areas

The genotypes were evaluated at the Holeta Agricultural Research Center in Ethiopia, under field conditions during the 2022 and 2023 cropping seasons. The location was expected to represent the major and potential bread wheat production area in central highlands of Ethiopia, the location also recognized hot-spot for *Z. tritici* epidemic. The center is 2,400 meters above sea level and located at latitude 9°00' N and longitude 38°30' E. With lowest and maximum temperatures of 6 °C and 22 °C, respectively, it receives 1,144 mm of rainfall annually. The soil type known as nitosols has a pH of 6.0 (Tamene *et al.*, 2015).

Plant Materials

A total of 45 bread wheat genotypes were evaluated for their reaction to the *Z. tritici* field population at Holeta during the 2022 and 2023 cropping seasons. The susceptible variety "Paven- 76," the moderately susceptible variety "Kingbird," the moderately resistant variety "Alidoro," and the resistant variety "Hedasie" were included as controls (Table 1).

Table.1. Wheat varieties and lines included in the study at Holeta, during 2022 and 2023 Cropping season

**S/N**	**Genotype**	**Pedigree**	**Origin**
**1**	BIQA	ETBW 6095	EIAR
**2**	Meraro	*M/4/HAR 1709/ 3/M//24/E*	EIAR
**3**	Alidoro	*HK-14-R251*	EIAR
**4**	Madda walabu	*TI/3/Fn/Th/Nar59 *2/4/Bol'S'*	EIAR
**5**	Hulluka	*UTQUE96/3/PYN/BAU//MILAN*	OARI
**6**	Kingbird	TAM200/TUI/6/PVN//CAR422/ANA/5/BOW/CROW//BUC/PVN/3/YR/4/TRAP#1	EIAR
**7**	Danda'a	*KIRITATI//2*PBW65/2*SERI.1B*	EIAR
**8**	ET-13A2	*UQ 105 SEL. X ENKOY*	EIAR
**9**	Pavon-76	*VCM//CNO"S"/7C/3/KAL/BB*	EIAR
**10**	Sofumar	*LIRA 'S'/TAN"S"*	OARI
**11**	KI 6295-4A	*Romany X GB-GAMENYA*	EIAR

Table.1. continued

S/N	Genotype	Pedigree	Origin
**12**	K 6290 Bulk	*(AF.MAYOXGEM)XROMANY*	EIAR
**13**	Hoggana	*PYN/BAU//MILAN*	EIAR
**14**	Salamouni	*Differential line*	Canada
**15**	6B662	*Differential line*	Brazil
**16**	Coulter	DT-188/Dt-224//DT-182	Israel
**17**	Erik	*Differential line*	USA
**18**	Glenlea	*Differential line*	Israel
**19**	ND-495	*Differential line*	Uruguay
**20**	Veranopolis	*Differential line*	Iran
**21**	Israel 493	*Differential line*	Iran
**22**	Tadina	*Differential line*	CIMMYT
**23**	Shafir	*Differential line*	CIMMYT
**24**	Estanzuela Federal	*Differential line*	Canada
**25**	KK4500	*Differential line*	CIMMYT
**26**	KM7	*Differential line*	CIMMYT
**27**	Gondo	*Differential line*	USA
**28**	BR 18	*Differential line*	CIMMYT
**29**	Catbird (CBRD)	*CBRD*	CIMMYT
**30**	BR 34	*Differential line*	CIMMYT
**31**	Frontana	*Differential line*	CIMMYT
**32**	Mutus	*Differential line*	Brazil
**33**	Murga	*Differential line*	CIMMYT
**34**	Blouk #1	*Differential line*	CIMMYT
**35**	Chiba//PRLII/cM65531/3/S KAUZ/BAV92	ESWYT 141	CIMMYT
**36**	CIANO T79	*Differential line*	CIMMYT
**37**	SOKOLL//W15.92/WBLL1	*Differential line*	CIMMYT
**38**	WAXWING*2/CIRCUS	*Differential line*	
**39**	Hidasie	ANAC/3/PRL/SARA//TSI/VEE#5/4/CROC-1/AE.SQUAROSA(224)//OPATTA	EIAR
**40**	Line	ETW17-86	EIAR
**41**	Line	ETW17-221	EIAR
**42**	Line	ETW17-246	EIAR
**43**	Line	ETW17-85	EIAR
**44**	Line	ETW17-115	EIAR
**45**	Line	ZERBA-6/FLAG-6/3/TAM200/PASTOR//TOBA97	EIAR
**36**	CIANO T79	*Differential line*	CIMMYT

Sowing and Experimental Design

The research was conducted using a randomized complete block design, which included three replications. The experimental area underwent thorough tillage, being plowed three times prior to

sowing, and planting rows were established with hand-pulled row maker. Seeds were sown using the hand drilling technique at a depth of approximately five cm. Bread wheat genotypes were planted in 28 of June, in accordance with the recommended sowing period for wheat in Holeta (Tufa *et al*., 2019). Each plot consisted of two rows, each measuring one meter in length and spaced 20 cm apart, with a seeding rate of 150 kg per hectare (Tufa *et al*., 2019). Inorganic fertilizers were uniformly applied across all plots at a rate of 100 kg per hectare of di-ammonium phosphate (DAP) and 150 kg per hectare of urea, following the recommendations for bread wheat cultivation. The entire amount of DAP and half of the urea were applied at the time of planting, while the remaining half of the urea was added during the mid-tillering phase. Hand weeding was performed to manage weed growth in the experimental field (Tufa *et al*., 2019).

Data Collection

Disease parameters such as severity and pycnidia coverage were collected starting from the early grain filling stage of wheat growth (Zadoks *et al*., 1974), which is typically when disease onset occurs under Holeta conditions. This evaluation utilized an adapted version of the double-digit scoring system (00-99) developed by Saari and Prescott, which is based on a severity scale designed for assessing foliar diseases in wheat (Saari and Prescott, 1975; Eyal *et al*., 1987). Visual evaluations of the disease were quantified by measuring the percentage of leaf area exhibiting necrosis (N) and the coverage of pycnidia (PC). The combination of these metrics was used to estimate the overall disease severity, and was calculated using the specified formula:

$$\text{Disease severity (\%)} = \left(\left(\frac{\mathrm{D1}}{\mathrm{Y1}}\right) * \left(\frac{\mathrm{D2}}{\mathrm{Y2}}\right)\right) * 100$$

Where *D1* represents disease progression relative to plant height, *D2* reflects the diseased leaf area, and *Y1* and *Y2* are the maximum scale values (Saari and Prescott, 1975; Eyal *et al*., 1987).

To assess disease progression over time, we record severity data weekly for six weeks, beginning at the onset of the disease and ends when the flag leaf infection reached 70% on susceptible genotype. The area under the disease progress curve (AUDPC) serves as a quantitative measure of disease intensity over a specified period. This is accomplished by segmenting the time variable (in days) and calculating the average disease intensity for each interval between consecutive time points (Madden *et al.*, 2007; Tilahun *et al.*, 2020). The AUDPC is calculated using the following formula:

$$AUDPC\ \%/day = \sum_{i=1}^{n-1} \frac{y_i + y_{i+1}}{2} \times (t_{i+1} - t_i)$$

Where, "Y_i" denotes the assessment of a disease at the ith observation, "t_i" signifies the time, expressed in days, associated with the ith observation, and "n" represents the overall count of observations.

All essential agronomic data were collected from two rows within each plot. The data collection was conducted on both a plant and plot basis. For the analysis of plant-based data, a random selection of ten plants from each plot was made to evaluate a range of characteristics. These characteristics included the number of tillers per plant, the number of kernels per spike, the spikelet count per spike, as well as measurements of plant height (cm), peduncle length (cm), and spike length (cm). Additionally, further data were gathered on the number of days required to reach heading (75%), the duration until maturity (90%), the grain filling period, the density of productive tillers per square meter, the weight of a thousand kernels (grams), and the grain yield (t/ha) on a plot-specific basis. Finally, the resistance levels of the genotypes were classified based on the mean percentage of pycnidia coverage obtained from six score made starting from diseases on set to 70% of the flag leaf infection recorded on susceptible genotype, which was

estimated and grouped using a 0–5 scale as described by Adhikari *et al*. (2003). Where; 1 = represents pycnidia in a lesions (≤5%); 2 = denotes a density of pycnidia in many or most lesions, usually unevenly distributed (6–15%); 3 = signifies even distribution of pycnidia at moderate density over most of the lesion (16–25%); 4= indicates a high concentration of pycnidia present across the majority of lesions (exceeding 25%), while a score of 5 represents the highest density of pycnidia (surpassing 40%). The Disease ranging from 0 to 1 categorize the genotype as resistant (R), a score of 2 denotes moderately resistant (MR), a score of 3 signifies moderately susceptible (MS), and scores of 4 to 5 are classified as susceptible (S).

Data Analysis

The agro-morphological data, including disease parameters, were subjected to variance analysis using SAS software version 9.2 (SAS, 2008), following the standard procedures outlined by Gomez and Gomez (1984). Severity percentage data were transformed using arcsine transformation (Sokal and Rohlf, 1995). The validity of further statistical analyses, such as ANOVA, was ensured by using Bartlett's test to determine whether the error variances for distinct genotypes across different attributes were comparable. ANOVA and many other statistical tests need the assumption of homoscedasticity, which is checked by this test. The differences between treatment means were compared using the least significant difference (LSD) test at a 5% level of significance when the ANOVA indicated a significant difference among genotypes. Diversity in disease resistance among the genotypes was assessed through cluster and Principal Component analysis based on the disease parameters, utilizing the computer software SPSS.

Results

Analysis of variance

The comprehensive analysis of variance conducted over two years revealed statistically significant differences ($P \leq 0.01$) among the years, genotypes, and the interaction between genotype and year. The significance of these traits indicates a substantial level of genetic variability concerning disease resistance and yield characteristics. The interaction between genotype and year demonstrated notable values for severity, Area Under the Disease Progress Curve (AUDPC), plant height, number of tillers, internode length, number of seeds per spike, thousand-kernel weight, and yield across the two years and among the various genotypes (Table 3). This finding suggests that the performance of these traits was inconsistent across the years.

Table.3. Combined analysis of variance over the year for *Z.tritici* blotch Severity, grain yield, and yield-related characters of bread wheat genotype grown at Holeta during the 2022 and 2023 growing seasons

**Trait**	**MSG (44)**	**MSY (1)**	**MSGY (44)**	**MSB**	**MSE**	**Mean**	**Cv.**	**R^2**	**LSD**
**Severity**	0.0735**	0.5618**	0.068**	0.0299**	0.00428	0.83	7.86	0.90	0.0746
**AUDPC**	1284279.44**	8214937.84**	1236429.11**	468196.27**	81508.8	2317.52	12.32	0.89	325.28
**Plant height**	1041.048**	31948.03**	602.541**	167.315**	30.2661	92.063	5.98	0.95	6.268
**Number of tillers per plant**	0.0819**	12.752**	0.0704**	0.2326**	0.0677	2.590	10.05	0.62	0.2965
**Internode length**	4.3226**	428.65**	4.2447**	0.2028**	0.3393	7.253	8.03	0.93	0.6636
**Number of seeds per spick**	0.9343**	0.60314**	0.8623**	0.4579**	0.1959	6.481	6.83	0.70	0.5043
**Thousand kernel weight**	228.58199**	11774.4**	249.1234**	126.693**	163.824	33.515	38.19	0.53	14.583
**Yield ton/hectare**	567.399**	567.399**	567.399**	567.399**	71.617	34.922	24.23	0.87	9.6418

*, ** Significant at $p \leq 0.05$, and $p \leq 0.01$ probability level respectively and ns non-significant, () parenthesis indicate degrees of freedom. MSG= Mean Squares of Genotype, MSY= Mean squares of year, MSGY = Mean square of genotype x year interaction, MSE = Mean squares of error, CV = Coefficient of variation.

Bread wheat Genotype reaction to *Z. tritici* blotch

The results indicated that the genotype response was 24.4%, with 25.2% classified as resistant. Additionally, 30.4% and 24.4% were categorized as moderately resistant, while 22.2% and 20.7% were deemed moderately susceptible. Finally, 22.96% and 29.63% of the genotypes were found to be susceptible during the 2022 and 2023 cropping seasons, respectively (Fig. 1).

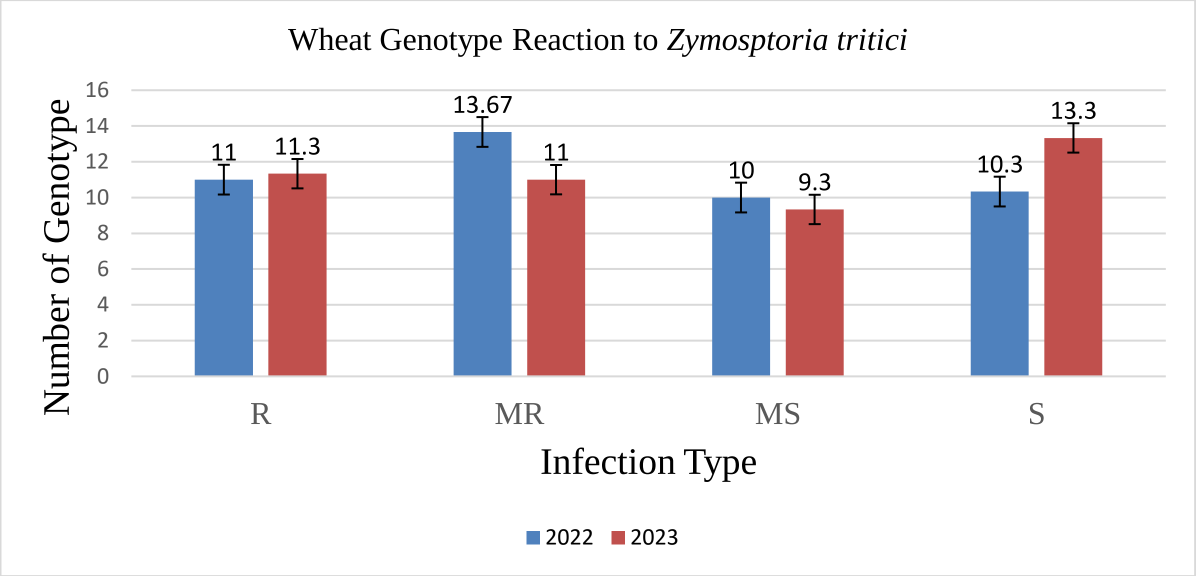

Fig 1. Number of varieties representing different reactions of *Z.tritici*

Among the 45 genotypes assessed over a two-year period, the cultivar "Catbird" exhibited the highest susceptibility, recording a leaf blotch severity score of 70.44% and a pycnidia coverage score of 4.5. In contrast, the cultivar "ETW17-115" demonstrated the greatest resistance, with a leaf blotch severity score of 29.52% and a pycnidia coverage score of 1.3 (Table 2). The data indicate various infection types across different wheat varieties and growing seasons. Among the evaluated genotypes, 11 and 11.3 exhibited a resistant response in both seasons, receiving infection type ratings of 0-1. Additionally, genotypes 13.7 and 11 displayed moderate resistance, with infection type ratings of 1-2 during both seasons. The remaining genotypes showed

moderately susceptible and susceptible responses, with infection type ratings of 3 and 4, respectively (Table 2). It is crucial to identify genotypes that consistently exhibit resistance throughout the year for the purposes of resistance breeding and to serve as sources of resistance.

Table.2. Leaf blotch severity (LB) and infection types (ITs) and Pycnidia coverage (P), of the bread wheat genotypes to *Z.tritici* blotch during (2022 and 2023) at Holeta, Ethiopia.

**S/N**	**Variety**	**2022**		**2023**		**Overall**		
		P	IT	P	IT	LB%	P	IT
**1**	BIQA (ETBW 6095)	3	MS	2.667	MS	54.09	2.833	MS
**2**	Meraro	3.33	MS	2.667	MS	44.65	2.998	MS
**3**	Alidoro (HK-14-R251)	2	MR	2	MR	52.07	2	MR
**4**	Madda walabu	2	MR	2	MR	56.59	2	MR
**5**	Hulluka	2	MR	2	MR	50.36	2	MR
**6**	Kingbird	2	MR	2	MR	53.09	2	MR
**7**	Danda’a	2	MR	2	MR	56.90	2	MR
**8**	ET-13A2	1.667	MR	2.333	MS	50.67	2	MR
**9**	Pavon-76	1.333	R	1.333	MS	56.58	1.333	R
**10**	Sofumar	1	R	1	R	49.25	1	R
**11**	KI 6295-4A	4	S	4	S	68.23	4	S
**12**	K 6290 Bulk	3	MS	3.33	MS	66.37	3.167	MS
**13**	Hoggana	4	S	3.667	S	64.31	3.833	S
**14**	Salamouni	1.333	R	1.667	MR	50.63	1.5	MR
**15**	6B662	1	R	0.333	R	29.84	0.667	R
**16**	Coulter	1	R	0.667	R	33.75	0.833	R
**17**	Erik	1	R	0.333	R	36.14	0.667	R
**18**	Glenlea	4	S	3.333	MS	54.96	3.667	S
**19**	ND-495	2	MR	2	MR	57.91	2	MR
**20**	Veranopolis	4	S	4.667	S	60.05	4.333	S
**21**	Israel 493	1.333	R	1	R	47.11	1.167	R
**22**	Tadina	4	S	5	S	67.76	4.5	S
**23**	Shafir	3.667	MS	3	MS	66.54	3.333	MS
**24**	Estanzuela Federal	4	S	4.667	S	66.66	4.333	S
**25**	KK4500	3	MS	2.667	MS	64.26	2.833	MS
**26**	KM7	2	MR	2.333	MR	55.66	2.167	MR
**27**	Gondo	1.333	R	0.333	R	27.48	0.833	R
**28**	BR 18	2	MR	2.333	MR	57.69	2.167	MR
**29**	Catbird (CBRD)	4	S	5	S	70.44	4.5	S
**30**	BR 34	3	MS	2.667	MS	61.64	2.833	MS
**31**	Frontana	2.333	MR	2	MR	52.14	2.167	MR
**32**	Mutus	1	R	1	R	49.16	1	R
**33**	Murga	3	MS	3.333	MS	59.02	3.167	MS

Table 2 continued….

**S/N**	Variety	2022		2023		Overall		
		P	IT	P	IT	LB%	P	IT
**34**	Blouk #1	1	R	0.667	R	37.29	0.833	R
**35**	Chiba//PRLII/cM65531/3/SKAUZ/BAV92	4	S	4.667	S	67.72	4.333	S
**36**	CIANO T79	4	S	3.667	S	60.62	3.833	S
**37**	SOKOLL//W15.92/WBLL1	3	MS	2.667	MS	58.52	2.833	MS
**38**	WAXWING*2/CIRCUS	3.333	MS	3.667	S	64.45	3.5	S
**39**	Hidasie	3	MS	3.333	MS	58.56	3.167	MS
**40**	ETW17-86	4	S	5	S	58.38	4.5	S
**41**	ETW17-221	3.333	MS	3.667	S	59.68	3.499	S
**42**	ETW17-246	4	S	4.333	S	60.05	4.167	S
**43**	ETW17-85	2	MR	2.667	MS	52.76	2.333	MR
**44**	ETW17-115	1.667	MR	1	R	29.52	1.333	R
**45**	ZERBA-6/FLAG-6/3/TAM200/PASTOR//TOBA97	4	S	4	S	62.57	4	S

Cluster Analysis

The spatial relationships among the various cluster centroids of wheat genotypes are illustrated in Figure 2. A comprehensive analysis of the dendrogram revealed the presence of four clusters, each demonstrating a minimum similarity level of 0.2 based on Euclidean distance within the UPGMA clustering framework. These clusters were categorized into two distinct groups: Group A and Group B. Group A consisted of three clusters—Cluster I, Cluster II, and Cluster III—while Group B was represented by a single cluster, Cluster VI (Fig. 2).

Cluster I comprised ten genotypes, accounting for 22.2% of the total genotypes, among which four are classified as differential lines. The differential lines include “Tadiana” (Stb4 & Stb6), “Shafir” (Stb6), “Estanzuela Federal” (Stb7 & Stb12), and “Kavkaz-K4500” (Stb10 + Stb12 + Stb6 + Stb7), each of which possesses resistance genes. Additionally, these genotypes exhibit reactions that range from susceptible to moderately susceptible. It is also possible that the

resistance genes found in the differential lines may be present in other non-differential genotypes within this cluster.

Cluster II consists of fifteen genotypes, representing 33.3% of all the genotypes. Among these, two are differential lines: the differential line “Veranopolis” carries the resistance genes (Stb10, Stb12, Stb6, and Stb7), and the differential line “Murga” carries resistance gene (Stb16). This cluster demonstrates a moderately susceptible response. It is likely that the cultivars included in this group may also carry resistance genes similar to those found in the differential genotypes.

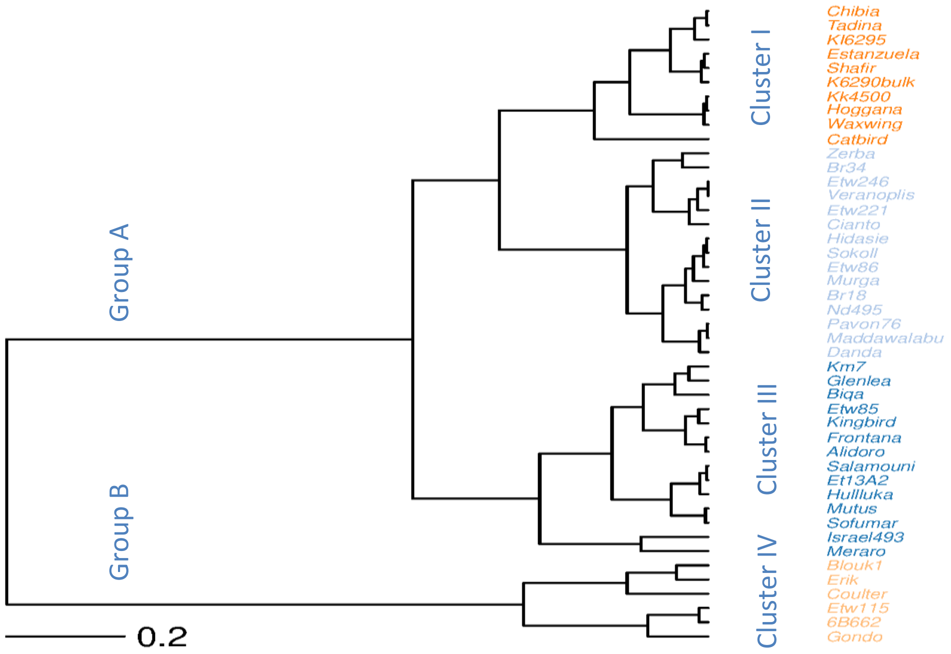

Fig 2. UPGMA clustering of 45 bread wheat genotypes reaction to *Z. tritici* under natural infection conditions, Holeta, Ethiopia (2022/23)

Cluster III includes fourteen genotypes, accounting for 31.1% of the total genotypes. Among these, three are differential lines, including "Israel-493," which possesses the resistance genes Stb3 and Stb6 that provide a resistant response to *Z. tritici* blotch. Another differential line, "Salamouni," is noted for its Stb13 and Stb14 resistance genes. The differential line "KM7" contains the Stb16 gene, recognized for its broad-spectrum resistance. The genotypes within this cluster may carry these resistance genes, and their overall response is characterized by moderate resistance.

Cluster VI consists of six genotypes, each demonstrating a resistance response. Notably, this group lacks any differential lines for *Z. tritici* resistance, suggesting that the R gene found in these genotypes requires molecular analysis and warrants further exploration as a potential source of resistance.

UPGMA (un-weighted pair group method with arithmetic mean) analysis revealed that the genotypes were classified into eight distinct clusters. The resistant and susceptible genotypes were organized into separate groups. Notably, the genotypes found in cluster VIII demonstrated resistance to *Z. tritici* blotch, whereas the other members of the clusters exhibited varying levels of susceptibility, ranging from susceptible to moderately resistant. With the identification of promising genotypes through the *Z. tritici* resistance screening experiment conducted on wheat, further research can be pursued by selecting the most beneficial genotypes. These chosen genotypes may significantly contribute to the development of varieties that are resistant to *Z. tritici* blotch.

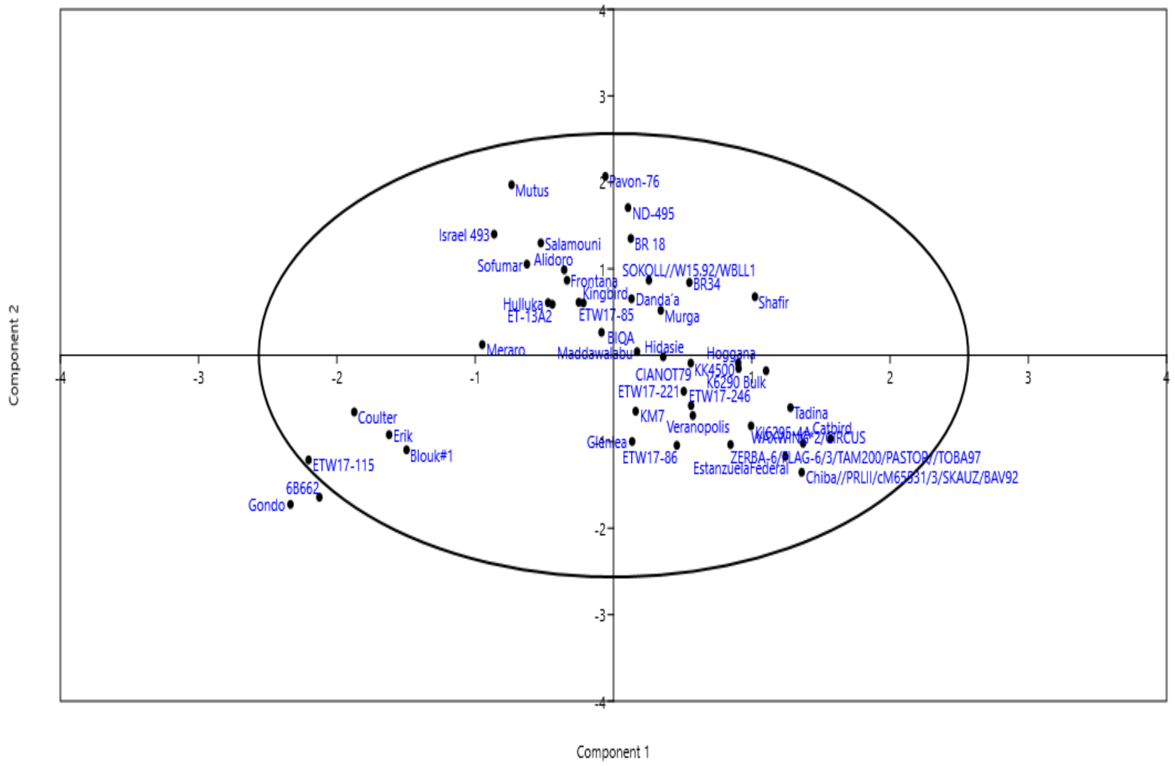

Fig 3. Result of principal component analysis executed on over year mean severity and AUDPC of *Z. tritici* blotch of forty five different wheat genotypes included in the study, Holeta, Ethiopia.

In the presented loading plot, cultivar “Gondo”, “6B662”, “ETW17-115”, “Coulter”, “Erik”, and “Blouk#1” exhibit considerable negative coefficients on component 2. This suggests that this component primarily evaluates the genotype's diminished severity in response to Z. tritici blotch and assesses resistance levels. In contrast, cultivar “Tadina”, “Chiba//PRLII/cM65531/3/SKAUZ/BAV92”, and “WAXWING*2/CIRCUS” display significant positive coefficients on component 1, indicating that this component mainly represents genotype susceptibility to the disease. The remaining genotypes are distributed across both positive and negative coefficients, demonstrating reactions that are moderately resistant and moderately susceptible.

Discussions

To date, the assessment of wheat genotypes for their resistance to *Z. tritici* blotch has been conducted in both protected and open field conditions, using susceptible checks as controls. However, including differential lines in the set of wheat genotypes to be screened is crucial for identifying which *Stb* gene is effective, determining the resistance of specific wheat lines to particular pathotypes, and understanding the infection types. This information enables researchers to postulate the presence of genes in unknown wheat lines (Tabib *et al*., 2012).

Disease severity in plant-pathosystems can be assessed either once or multiple times at specified intervals, starting from the onset of the disease until the conclusion of the epidemic. The initial assessment method serves to estimate various parameters, including the area under the disease progress curves (AUDPC), which numerous pathologists employ to analyze data concerning resistance to *Z. tritici* (Kema *et al*., 1996; Chartrain *et al*., 2004; Mojerlou *et al*., 2009; Ferjaoui et al., 2015). In this study, we evaluated AUDPC, pycnidial coverage (PC), and the necrotic area (N) under natural infection conditions to examine how different wheat genotypes resist *Z. tritici* blotch over two growing seasons. During this period, the pathogen seemed to have adapted to particular wheat species (Chedli *et al*., 2020).

The extensive analysis of variance conducted over a two years period revealed statistically significant differences ($P \leq 0.01$) across years, genotypes, and the interaction between genotype and year. Furthermore, it suggests that a variety selected for *Z. tritici* resistance traits in one year may not exhibit the same relative performance in subsequent years. If genotypes do not demonstrate consistent performance for these traits over time, breeders may need to conduct extensive varietal evaluations through appropriate testing years and adapt their breeding programs accordingly. To accurately assess varietal performance in terms of significant disease

resistance, it is crucial to consider multiple years and examine a broader range of location interactions, as well as to identify trait performance in relation to the effects of different years. These findings are consistent with those of Alemu (2016), who reported a highly significant interaction among variety, location, and year for most traits examined in bread wheat. Furthermore, disease severity showed a highly significant difference among genotypes and across the years, suggesting a shift in race over time and indicating that wheat genotypes respond variably to the prevailing race.

Wheat genotypes exhibit varying levels of resistance to *Z. tritici* blotch. The findings indicated that 24.4% and 25.2% of the genotypes were classified as resistant, while 30.4% and 24.4% were categorized as moderately resistant. Additionally, 22.2% and 20.7% were deemed moderately susceptible, and 22.96% and 29.63% were identified as susceptible during the 2022 and 2023 cropping seasons. These results are consistent with research conducted by Ahmet (2023), who noted a spectrum of reactions among wheat genotypes, ranging from resistant to susceptible to *Z. tritici*. Similarly, Odilbekov *et al*. (2019) reported that immune, highly resistant, and very susceptible reactions were not observed. Furthermore, Omrani *et al*. (2023) tested 53 elite wheat lines against *Z. tritici* at the adult plant stage under natural infection conditions during the 2020 and 2021 growing seasons, finding that the materials exhibited moderately resistant, moderately susceptible, and susceptible reactions. Their studies also revealed high virulence in the resistance genes Stb3, Stb4, Stb7, Stb8, and Stb9, while avirulence was noted in Stb11 and Stb18 across both years. Additionally, Muche (2022) highlighted significant variation in responses to *Z. tritici* among commercially available bread wheat varieties and advanced lines, ranging from resistant to susceptible. This observation is corroborated by findings from Teklay *et al*. (2015), which

documented diverse responses among bread wheat varieties to *Z. tritici* disease in Tigray, Ethiopia.

All genotypes were categorized based on severity as variables, leading to the creation of a dendrogram. The UPGMA analysis indicated that these genotypes were organized into four distinct clusters. It is important to mention that resistant and susceptible genotypes were grouped into different clusters. Cluster VI included genotypes that exhibited resistance to *Z. tritici* blotch, while cluster one comprised the most susceptible genotypes. Among the 45 genotypes evaluated over a two-year period, the cultivar “Catbird” displayed the highest level of susceptibility, with a leaf blotch severity score of 70.44% and a pycnidia coverage score of 4.5. Conversely, the cultivar “ETW17-115” demonstrated the highest resistance, recording a leaf blotch severity score of 29.52% and a pycnidia coverage score of 1.3. Four separate groups were identified by the evaluation of infection types: sensitive, moderately susceptible, resistant, and moderately resistant. The genotypes classified within the superior cluster may prove to be highly beneficial for the development of *Z. tritici* blotch-tolerant or resistant varieties, as well as for other breeding initiatives.

The distribution of these genotypes across the clusters revealed significant genetic variability concerning the resistance traits to *Z. tritici* that were examined. The relationships among the members of these clusters indicated that clusters I, and II, exhibited the highest mean severity values, whereas cluster VI demonstrated the lowest. The findings confirmed that all genotypes within cluster VI, under the trial conditions, displayed the least mean values for *Z. tritici* blotch severity. Following evaluations across different annual conditions, all six genotypes in cluster VI have potential for use as sources of *Z. tritici* resistance. Additionally, these exceptional

genotypes could be incorporated into hybridization programs aimed at developing varieties that are both resistant to *Z. tritici* and high-yielding.

Principal component analysis revealed the presence of four distinct components. The first two components explain the majority of the variation in the dataset. A score plot was created to evaluate the data's structure and to detect possible clusters, outliers, and trends. The positioning of data points on this plot may indicate the presence of two or more separate distributions within the dataset. Typically, coefficients can range from -1 to 1, with values nearing -1 or 1 indicating a substantial influence of the variable on the component. In contrast, coefficients close to 0 suggest a minimal effect of the variable on the component. A comprehensive analysis of the coefficients can provide additional insight into the connection of each component with the relevant variables. The analysis showed that components 1 and 2 combined explain about 80% of the variation. Specifically, the eigenvalues for PC1 and PC2, both exceeding 1, correspond to individual variance contributions of 71.53% and 8.54%, respectively, in relation to the cumulative variation of *Z. tritici* severity. The first two principal components were plotted along the PC axes 1 and 2, demonstrating significant variability among the existing wheat lines and controls (Fig. 3). Severity values exhibiting the highest absolute magnitudes approaching unity within PC1 exert a more significant impact on clustering compared to those with lesser absolute values that are closer to zero (Chahal and Gosal, 2002). Consequently, in this study, the differentiation of advanced lines into distinct clusters was attributed to the cumulative effects of severity and the Area under the Disease Progress Curve (AUDPC).

CONCLUSION

This research emphasizes the considerable differences in resistance to *Zymoseptoria tritici* among 45 bread wheat genotypes assessed in Ethiopia, pinpointing notable cultivars like ETW17-115, which exhibits strong resistance, and Catbird, which shows high susceptibility. The study also revealed that incorporating differential lines into the wheat screening program for *Z. tritici* helps identify the specific pathotypes to which the lines are resistant, aiding in gene postulation. These results highlight the necessity of integrating resistant genotypes into breeding programs to improve wheat production and reduce the effects of this harmful disease.

CONFLICT OF INTEREST STATEMENT: The authors declare that they have no conflict of interest.

ACKNOWLEDGEMENTS

For providing the experimental material, and research facility, we thank Holetta agricultural research center Breeding and Pathology program. Many thanks to plant pathology research stuff for unreserved support during the research period. We thank Ethiopian Institute of Agricultural research for the fund.

DATA AVAILABILITY STATEMENT: Data are available upon request from the corresponding author.

ETHICS S TATEMENT: The study was approved by review and Ethics Committee, Harumaya University, Agriculture and Environmental Sciences School of Plant Sciences in February, 2023.

Reference

Adhikari, T.B., Anderson, J.M. and Goodwin, S.B. Identification and Molecular Mapping of a Gene in Wheat Conferring Resistance to *Mycosphaerella graminicola*. *Phytopathology,* 2003; 93(9), 1158-1164. doi: 10.1094/PHYTO.2003.93.9.1158.

Ahmet ÇAT. Evaluation of Some Bread Wheat (*Triticum aestivum* L.) Varieties Reaction to *Septoria Tritici* Blotch Disease, *Turk J Agric Res* 2023; 11(1): 18-25. doi: 10.19159/tutad.1373924

Alemar Said and Temam Hussein. Epidemics of *Septoria tritici* Blotch and its Development over Time on Bread Wheat in Haddiya Kambata Area of Southern Ethiopia. *Journal of Biology, Agriculture and Healthcare,* 2016; 6(1), 47-57.

Alemu D B. Genetic Variability and Association Among Grain Yield and Yield Related Traits of Bread Wheat (*Triticum aestivum* L.) Genotypes. MSc Thesis Postgraduate Program Directorate, 2016; Haramaya University, Ethiopia.

Bekle Kassa and Yitagesu Kuma . Phenotyping of Bread and Durum Wheat Accessions, lines and Varieties for *Septoria* leaf blotch (SLB) and *Fusarium* head blight at Holetta , Ethiopia. Eshetu Derso, Bedada Girma (eds) in *Proceedings of the Delivering Genetic Gain in Wheat (DGGW) Project Closing Workshop*. Addis Ababa, Ethiopia, 2020; pp.120.

Bockus,W.W., Bowden R.L, Hunger R.M, Morrill W.L., Murray T.D., and Smiley R.W. "Compendium of Wheat Diseases and Pests: Third Edition." *American Phytopathological Society,* 2010; St. Paul, MN

Brown, J.K.M., Chartrain, L., Lasserre-Zuber, P. and Saintenac, C. Genetics of Resistance to *Zymoseptoria tritici* and Applications to Wheat Breeding. *Fungal Genetics and Biology,* 2015; 79, 33-41. https://doi.org/10.1016/j.fgb.2015.04.017

Chahal G. S., Gosal S. S. Principles and Procedures of Plant Breeding: Biotechnological and Conventional Approaches, CRC Press, 2002, Alpha Science International Ltd. Harrow, U.K.

Chartrain, L., Brading, P.A., Makepeace, J.C., Brown, J.K.M. Sources of Resistance to *Septoria tritici* Blotch and Implications for Wheat Breeding." *PlantPathol*. 2004; 53(4),454–460. https://doi.org/10.1111/j.1365-3059.2004.01052.x

Chedli, Rim Bel Hadj, Sarrah Ben M'Barek, Amir Souissi, Amor Yahyaoui, Salah Rezgui, and Hanène Chaabane. Screening for Resistance of Tunisian, Moroccan and Algerian Wheat Cultivars to *Zymoseptoria Tritici* in Northern Tunisia. *Journal of Plant Pathology* , 2020;102(4): 1085–95. https://www.jstor.org/stable/48741582.

CSA (Central Statistical Authority). Agricultural Sample Survey 2020/21 (2013 E.C). Report on Livestock and Livestock Characteristics. Volume II, Statistical Bulletin 589, Central Statistical Authority, 2021; Addis Ababa.

Dreisigacker S, Wang X, Martinez Cisneros BA, Jing R, Singh PK. Adult-plant Resistance to *Septoria tritici* Blotch in Hexaploid Spring Wheat. *Theor Appl Genet,* 2015;128:2317–2329. https://doi.org/10.1007/s00122-015-2587-9

Endale Hailu and Getaneh Woldeab. Survey of Rust and Septoria Leaf Blotch Diseases of Wheat in Central Ethiopia and Virulence Diversity of Stem Rust *Puccinia graminis*f.

sp.*tritici*." *Advances in Crop Science and Technology*, 2015; 3(2), 1-5.

Eriksen L., Borum F., Jahoor A. Inheritance and localization of resistance to *Mycosphaerella graminicola* causing *Septoria tritici* blotch and Plant Height in the Wheat (*Triticum aestivum* L.) Genome with DNA Markers, *Theor Appl Genet*; 2003;107(3):515-27. doi: 10.1007/s00122-003-1276-2.

Eyal, Z., Amiri Z., and Wahl I. Physiologic Specialization of *Septaria tritici. Phytopathology*, 1973; 63:1087

Eyal, Z., Scharen, A. L., Huffman, M. D., and Prescott, J. M. Global Insights in to Virulence Frequencies of *Mycosphaerella graminicola. Phytopathology*, 1985; 75:1456-1462.

Eyal, Z., and Levy, E. Variations in Pathogenicity Patterns of *Mycosphaerella graminicola* within *Triticum* spp in Israel." *Euphytica*, 1987; 36, 237–250. https://doi.org/10.1007/BF00730670

Ferjaoui S., Sbei A., Aouadi N., Hamza S. Monogenic Inheritance of Resistance to *Septoria tritici* blotch in durum wheat 'Agili'. *Int. J. Plant Breed*, 2011; 5:17–20.

Getachew Diriba. Agricultural and Rural Transformation in Ethiopia: Obstacles, Triggers and Reform Considerations Policy Working Paper. Agricultural and Rural Transformation in Ethiopia, Addis Ababa, Ethiopia. 2020; ISBN 978-99944-54-72-3

Kema GHJ, Verstappen ECP, Todorova M, Waalwijk C. Successful Crosses and Molecular Tetrad and Progeny Analyses Demonstrate Heterothallism in *Mycosphaerella graminicola. Curr Genet*, 1996; 30: 251-258.

Madden LV, Hughes G, Van den Bosch F. The Study of Plant Disease Epidemics. 2007; St. Paul, MN, USA: APS Press.

Mengistu, H., Getaneh, W., Yeshi, A., Rbka, D., Ayele, B. Wheat Pathology Research in Ethiopia. In:Hailu,G.,Tanner,D.G.,Mengistu,H.(Eds.),Wheat Research in Ethiopia: a Historical Perspective. IAR/CIMMYT, Addis Ababa, 1991; pp.173-217.

Meyer M, Bacha N, Tesfaye T, Alemayehu Y, Abera E, Hundie B. Wheat rust epidemics damage Ethiopian wheat production: A decade of field disease surveillance reveals national-scale trends in past outbreaks. PLoS ONE, 2021; 16(2): e0245697. https://doi.org/10.1371/journal.pone.0245697

Mojerlou Sh., Safaie N., Alizadeh A. Khelghatibana F. Study of Latent Period and Interactions Between Different *Septoria tritici* Genotypes and Different Wheat Cultivars and Lines in Greenhouse, *Trakia Journal of Sciences*, 2009;7(4), pp 7-17, ISSN 1313-3551

Muche Abebele Getnet. Assessment of Ethiopian Advanced Bread Wheat Genotypes and Varieties to *Septoria tritici* leaf blotch, *RJOAS*, 2022; 6(126), 181, UDC 633; DOI 10.18551/rjoas.2022-06.21

Odilbekov F, Armoniené R, Koc A, Svensson J and Chawade A. GWAS-Assisted Genomic Prediction to Predict Resistance to *Septoria Tritici* Blotch in Nordic Winter Wheat at Seedling Stage. *Front. Genet*. 2019; 10:1224. doi: 10.3389/fgene.2019.01224

Omrani, A., Roohparvar, R., Shahbazi, K. Reaction of Promising Wheat Lines to Septoria Leaf Blotch Disease at the Seedling and Adult Plant Stages. *Applied Entomology and Phytopathology*, 2023; 90(2): 195-208.

Raman H. and Milgate A. 2012. Molecular Breeding for *Septoria tritici* Blotch Resistance in Wheat, *Cereal Research Communications* 40(4), pp. 451–466, DOI: 10.1556/CRC.40.2012.4.1

Saari EE and JM Prescott. A Scale for Appraising the Foliar Intensity of Wheat Diseases. *Plant Disease Reporter*, 1975; 59 (5): 377–380.

SAS. 2008. Statistical Analysis Systems Institute. *Version 9.1*, SAS Institute Inc., Cary, North Carolina, USA.

Simon, M.R., Ayala, F.M., Corda, C.A., Order, M.S., Boerner, A. Molecular Mapping of Quantitative Trait Loci Determining Resistance to *Septoria tritici* Blotch Caused by *Mycosphaerella graminicola*. *Euphytica,* 2004; 138:41-48.

Sokal, R. R., and Rohlf, J. F. Biometry *(3rd ed.)*. 1995; New York: Freeman.

Tabib Ghaffary S.M., Faris J.D., Friesen T.L., Visser R.G.F., van der Lee T.A.J., Robert O., Kema G.H.J. New Broad-spectrum Resistance to *Septoria tritici* Blotch Derived from Synthetic Hexaploid Wheat. *Theor. Appl. Genet*. 2012;124:125–142. doi: 10.1007/s00122-011-1692-7.

Tamene L; Amede T; Kihara J; Tibebe D; Schulz S. (eds.). A review of soil fertility management and crop response to fertilizer application in Ethiopia: towards development of site- and context-specific fertilizer recommendation. CIAT Publication No. 443. International Center for Tropical Agriculture (CIAT), Addis Ababa, Ethiopia. 2017; 86 p. Available at:http://hdl.handle.net/10568/82996

Teklay Abebe Teferi and Zeray Siyoum Gebreslassie. Occurrence and Intensity of Wheat *Septoria tritici* Blotch and Host Response in Tigray, Ethiopia. *Crop Protection,* 2015; 68: 67-71

Tesfaye Zegaye, Girma Taye, D. Tanner, H. Verkuijl, Aklilu Agidie and W. Mwangi. Adoption of Improved Bread Wheat Varieties and Inorganic Fertilizer by Small Scale Farmers in Yelma Dansa and Farta Districts of Northwestern Ethiopia. Mexico, D.F. 2001; Ethiopian Agricultural Research Organization (EARO) and International Maize and Wheat Improvement Center (CIMMYT).

Tilahun Mekonnen, Teklehaimanot Haileselassie, Bekele G. Abayo & Kassahun Tesfaye. Virulence Variability of Ethiopian *Zymoseptoria tritici* Isolates and Efficacy of Wheat Genotypes and *Stb* Resistance Genes Against the Isolates. *Eur J Plant Pathol.,* 2020; 158(4):895-910 https://doi.org/10.1007/s10658-020-02125-3

Tufa Tolcha, Haji Kumbi, and Tolera Abera. Influence of Seed Rate and Row Spacing on Growth and Yield of Bread Wheat in Chelia District of West Showa Zone, Ethiopia,Journal of Natural Sciences Research ISSN 2224-3186 (Paper) ISSN 2225-0921 (Online) 2019; Vol.9, No.5, DOI: 10.7176/JNSR

USDA. World agricultural production. Circular series. WAP 11–22. 2023. https://apps.fas.usda.gov/PSDOnline/Circulars/2023/08/production.pdf.

Yohannes Azene, Birhanu Fentahun and Yeshambel Adugna. Field Response and Identification of Bread Wheat Genotypes to *Septoria tritici* Blotch in the Highlands of North Gondar. *Journal of Current Research in Food Science*, 2020; *1*(2), 09-17.

Zadocks, J. C., Chang, T. T., and Konzak, C. F. A Decimal Code for the Growth Stages of Cereals. *Weeds Res*, 1974; 14:415-421
