# Supplementary material for: Field evaluation of spring wheat genotypes reveals differential resistance to Zymoseptoria tritici in Ethiopia
Source: PLoS One. 2026 Jul 10;21(7):e0353375. doi: 10.1371/journal.pone.0353375 (PMC13353992; doi:10.1371/journal.pone.0353375)
Supplement: Attachment — Submitted filename: A rebuttal letter.docx [file pone.0353375.s009.docx]

**A rebuttal letter**

**Dear Sir/manadam**

Thank you to all the reviewers for their important time and expert feedback. Their comments and suggestions have been acknowledged and are now included in the revised manuscript. For a detailed response, refer to the table below

**Regards**

**1. Abstract and Introduction**

| **💬 Reviewer Comment** | **✔ Agreement Response** |
| --- | --- |
| - *“*The literature related to the topic needs to be included in the Introduction”. | I appreciate this thoughtful remark. I concur with and acknowledge all the remarks and recommendations provided in this section. I enhance the introduction by elaborating on the background section to offer a clearer insight into the problem domain and its significance. I additionally incorporate the recommended article “Kassie et al 2024 Plant Breeding and Kassie et al 2025 PONE.” And additional pertinent recent literature and data |

**3. Methodology**

| **💬 Reviewer Comment** | **✔ Agreement Response** |
| --- | --- |
| - *“*Plant materials: what is a differentia line? what is the rationale for selecting these genotypes? - N and PC are not well defined - Scoring and data collection not unambiguously described. Many data mentioned here is not used in the analysis and not presented in results. - why the researcher choose RCBD | Thank you for this important observation. I agree that additional procedural details will help others replicate the study. I also include the missing information in the plant material table, including the rationale whey we include those material, genotypes pedigree and they differential for  I accept the comment and I already done accordingly, I describe in detail the scoring and data collection, I remove those variable not use in the analysis  the 45 genotype laid in one block size 58meter, the plot size 2meter by 1meter size, the land we use were relatively has low variability within block that is way we use RCBD |
| “Explain how the sample size was determined.” | Thank you for pointing this out. I have now explained how the sample size was determined, based on the standard suggested by Eyal, 20 plants per plot are enough, but we record from 25 plant. It represent the population more than 20% |
| *“*Describe the sampling strategy*.”* | I appreciate your input. I concur that the sampling method required further explanation. We randomly select five plants from each of the four corners and one from the center in an X pattern, resulting in a total of 25 plants sampled from each plot for data recording |

**4. Results**

| **💬 Reviewer Comment** | **✔ Agreement Response** |
| --- | --- |
| - *“*new results need to be produced from the analysis using back-transformed severity data; Fig 1 is not appropriate and has to be removed.*.”* - the clusters need to be characterized with a figure displaying mean and SEM of disease variables for each cluster - the % variance explained by axis 1 and axis 2 on the PCA need to be provided. - The analysis is not clearly described. What was the response variable or variables. What traits are speaking about. - Figure 3, quality is unacceptable - I recommend correlation analysis between traits and importance of agronomic traits regarding to septoria resistance  • Speculating on potential genes present in the wheat genotypes were the goals of your study. did your study achieve the objective? • did you identify the pathotypes in your study | Thank you for noting this. I agree that and I already done the back transformation and put the result in table 4. I already remove Fig 1  I incorporated the table which composed of Characteristics of Cluster Based on Mean, Standard Deviation and Standards Error of Mean  In the PCA I included the table contain variance and Eigen values , the graph also improved  We use the variable Severity, AUDPC, and pycnidia coverage to determine the resistance nature of the genotypes  I already improved the Fig.3 and I also include correspondence analysis graph  I included the correlation analysis  The resistance genotype selected for the pathotype which affect Stb gene in *Veranopolis (Stb2 and Stb6) and Estanzuela Federal (Stb7)* |

**5. Discussion**

| **💬 Reviewer Comment** | **✔ Agreement Response** |
| --- | --- |
| *“*the results need to be compared to results reported in the literature. This includes Kassie et al 2024 Plant Breeding and Kassie et al 2025 PONE*.”* | Thank you for this important comment. I have expanded the discussion to include a more robust comparison of my findings with those from recent and foundational studies. I included suggested article information and other important recent literature |
| Several sentences repeat methods or results. including AUDPC | Thank you for this helpful comment. I removed those sentences from the discussion part. |
