# Supplementary material for: Field evaluation of spring wheat genotypes reveals differential resistance to Zymoseptoria tritici in Ethiopia
Source: PLoS One. 2026 Jul 10;21(7):e0353375. doi: 10.1371/journal.pone.0353375 (PMC13353992; doi:10.1371/journal.pone.0353375)
Supplement: Attachment — Submitted filename: PONE-D-25-41622_R1-AE.docx [file pone.0353375.s008.docx]

**Field Evaluation of Spring Wheat *(Triticum aestivum L.)* Genotypes Reveals Differential Resistance to *Zymoseptoria tritici* Blotch at Holetta, Ethiopia**

Habtewold Kifelew^1^, Habtamu Terefe^2^, Bekele Kasa ^1^, Tilahun Mekonen ^3^, Zelalem Bekeko ^2^ and Bulti Teso ^2^

^1^ Holetta Agricultural Research Center, P.O.Box 31/2003, Holeta, Ethiopia

^2^ Haramaya University, College of Agriculture and Environmental Sciences, P.O. Box 138, Dire Dawa, Ethiopia,

^3^ Addis Ababa University, Institute of Biotechnology, P.O. Box 1176, Addis Ababa, Ethiopia

Corresponding author: Habtewold Kifelew, ([kifelew@yahoo.com](mailto:kifelew@yahoo.com)) Telephone: +251913312650

**Abstract**

Septoria tritici leaf blotch disease, caused by *Zymoseptoria tritici*, and is a major disease of spring wheat impacting wheat production in Ethiopia as well as in many other countries around the world. This study evaluated 45 spring wheat genotypes for their resistance to *Z. tritici* during the adult plant stage under natural infection conditions. The field trial was conducted at the Holetta Agricultural Research Center in Ethiopia over two consecutive years, 2022 and 2023, utilizing a randomized complete block design with three replications. Disease assessments were carried out using a double-digit scale ranging from 00 to 99. A comprehensive analysis of variance performed over the two years indicated statistically significant differences (P ≤ 0.01) among the years, genotypes, and the interaction between genotype and year. Genotype is the main factor contributing to variation in resistance to *Z. tritici* within the 45 spring wheat collection, accounting for 70.94% of the total variance in disease Severity and 71.32% in AUDPC of disease severity. Disease severity varied from 46.46% to 73.49% and 8.73% to 71.71% during 2022 and 2023 respectively, while pycnidia coverage ranging from 2.23% to 35.7% and 1,.21% to 45.47% during 2022 and 2023 respectively. Four distinct disease reactions were identified: 24.4% and 25.2% of the genotypes were classified as resistant, 30.4% and 24.4% as moderately resistant, 22.2% and 20.7% as moderately susceptible, and 22.96% and 29.63% as susceptible during 2022 and 2023, respectively. The cultivar Catbird exhibited the highest level of susceptibility, while the cultivar Gondo demonstrated the strongest resistance. The cultivars could be grouped in eight clusters according to xxx. , with two clusters comprising six genotypes that displayed a resistant response. Notably, this cluster did not include any differential lines for *Z. tritici* resistance, indicating that the resistance genes present in these genotypes require molecular analysis and further investigation. The resistance genotypes identified in the study (Blouk #1, 6B662, Coulter, Erik, Gondo, and ETW17-115) exhibit resistance to the pathotypes that affect the major Stb genes found in Veranopolis (Stb2 and Stb6), Shafir (Stb6) and Estanzuela Federal (Stb7). The relationship between disease and yield-related traits indicates that severity and AUDPC have are negatively correlated with plant height, number of tillers per plant, spike length, number of seeds per spike, thousand kernel weight, and grain yield. Correspondence and principal component analyses revealed that there are three distinct trait groups among the 45 wheat genotype (resistant, high-yield potential and susceptible). Resistance genotypes selected in this study provide valuable insights for breeding programs aimed at enhancing wheat resistance to *Z. tritici*.

**Key word**: wheat, septoria tritici blotch, Zymoseptoria tritici, host plant resistance, breeding

**Introduction**

Spring wheat is the primary cereal crop in Ethiopia, producing 4.93 million metric tons with an average productivity of 2.73 tons per hectare. It is cultivated by 4,212,518 smallholder farmers across 1.8 million hectares [1]. Recently, due to irrigated wheat production initiative, the country has become one of the fastest-growing wheat producers in Africa. The report predicts that Ethiopia's wheat production will reach 6.5 million metric tons by the year 2025/26 [2]. However, Ethiopia's wheat production and productivity remain low compared to Egypt 6.4 tons/ha [3]. The low productivity is linked to various biotic and abiotic challenges, as well as limited adoption of modern agricultural technologies [4]). In terms of biotic stress, stem and leaf rust are prevalent in low and mid-altitude regions, while stripe rust (Yellow rust) and Septoria are more common in high-altitude wheat-growing areas of Ethiopia [5, 6, 7, 8]

Particularly in the highlands, *Zymoseptoria tritici,* the pathogen causing Septoria tritici blotch (STB) diseases, is among the most devastating diseases of wheat in Ethiopia, ranking second only to stripe rust. This pathogen is responsible for yield losses that range from 41% [9] to 82% [5, 6]. In addition to lowering productivity, *Z. tritici* infection degrades grain quality [10]. Due to frequent STB outbreaks and a lack of effective disease management strategies, wheat production in the area has recently become challenging.

Evaluating spring wheat for resistance to STB, is essential in worldwide wheat breeding because of the pathogen's significant evolutionary ability and the rising concern of fungicide resistance [11, 12]. STB can lead to considerable yield reductions, making genetic resistance the most sustainable and eco-friendly control method. Screening initiatives mainly concentrate on detecting two types of resistance, qualitative and quantitative (QTL). Qualitative resistance results of major *Stb* Genes, These genes provide a robust, specific resistance, typically adhering to a gene-for-gene model. Though successful against non-virulent pathogen strains, they are susceptible to being outpaced by the pathogen's swift development of virulence. To date, twenty-four resistance (R) genes against *Z. tritici* have been identified and documented [13]. From them 23 *Stb* genes have been genetically mapped in wheat [14]. These genes were found in wheat through field trials and studies on interactions between genotypes and isolates. Resistance to STB is primarily quantitative under field conditions [15, 16]. Quantitative Resistance (QTLs) offers a less complete yet long-lasting defense, governed by numerous genes with smaller, cumulative impacts. Contemporary breeding increasingly seeks a combination, or pyramiding, of both significant *Stb* genes and quantitative trait loci (QTLs) to enhance protection that is more resilient and durable. Currently the wheat genome contains a large number of quantitative trait loci (QTL) linked to resistance [17, 18, 19]. Since 2015, there have been findings indicating that 89 chromosomal regions harbor QTL associated with resistance to STB [15]. Alternative origins, such as synthetic hexaploid wheat, are being explored to integrate newly effective resistance genes such as Stb16q [20]. A recent study using QTL analysis discovered that genes involved in both quantitative and qualitative STB-wheat interactions are similar. The study also demonstrated that genes underlying pathogenicity QTL can be analogous to *Avr* genes [21].

Studies in Ethiopia have revealed significant variability in resistance among bread wheat, durum wheat, and triticale [22]. Eyal and his team [23, 24] investigated the virulence traits of 97 *Z. tritici* isolates obtained from 22 countries, including Ethiopia, by evaluating them on 35 varieties of wheat and triticale. Their findings confirmed isolate-cultivar specificity, indicating diverse virulence genes in the pathogen population. Molecular analyses by Tilahun [25] further demonstrated substantial genetic variability in resistance among 180 Ethiopian bread wheat genotypes. Genome-Wide Association Studies (GWAS) conducted on Ethiopian wheat have effectively pinpointed important genomic areas and new germplasm sources that provide consistent resistance to STB. These findings highlight the complex, polygenic basis of resistance and underscore the importance of addressing Genotype-by-Environment interactions [26, 27]. Screening programs in Tigray [28], Gondar [29], and Holetta [30] have also contributed to identifying resistant genotypes.

The purpose of this study was to utilize the phenotypic variation in spring wheat germplasm to identify resistance to STB. To achieve this, 45 spring wheat genotypes, including 10 differential lines for STB, were assessed under natural infection over two consecutive years at an open field site in the central highlands of Ethiopia. Various responses were observed, with some genotypes showing resistance to STB. Agronomic traits were also measured on the same genotypes, enabling an analysis of the relationship between resistance and agronomic characteristics in Ethiopian spring wheat. These resistance genotypes could be valuable for breeding programs aimed at enhancing the resilience of Ethiopian and global spring wheat against STB.

**Materials and methods**

**Description of the study area**

The genotypes were evaluated at the Holetta Agricultural Research Center in Ethiopia, under field conditions during the 2022 and 2023 cropping seasons. The location is expected to represent the major and potential spring wheat production area in central highlands of Ethiopia, the location is also recognized hot-spot for STB epidemic. The center is 2,400 meters above sea level and located at latitude 9°00' N and longitude 38°30' E. With lowest and maximum temperatures of 6 °C and 22 °C, respectively, it receives 1,144 mm of rainfall annually (Table 1). The soil type known as nitosols has a pH of 6.0 [31].

Table.1. Weather condition of Holetta during spring wheat growing season 2022, 2023

| Weather variables | Year 2022 | | | | | Year 2023 | | | | |
| --- | --- | --- | --- | --- | --- | --- | --- | --- | --- | --- |
|  | June | July | August | September | Mean | June | July | August | September | Mean |
| T^O^ min. | 9 | 10.2 | 10.5 | 9.8 | 9.875 | 9.8 | 10.5 | 10.2 | 10 | 10.125 |
| T^O^ max. | 26.5 | 25.7 | 25.9 | 24.5 | 25.65 | 22.3 | 20.9 | 21.2 | 21.4 | 21.45 |
| Rainfall | 200.7 | 390.8 | 331.7 | 218.7 | 285.475 | 112.4 | 202.3 | 271.8 | 166.4 | 188.225 |
| RH | 77 | 84 | 81 | 78 | 80 | 81 | 84 | 84 | 80 | 82.25 |

Source:-Holetta agricultural research center, weather station

**Plant materials**

A total of 45 bread wheat genotypes were evaluated for their reaction to the *Z. tritici* field population at Holetta during the 2022 and 2023 cropping seasons. Most of these genotypes were recently introduced to the country, and their disease resistance status is not yet known. Differential lines were included because we need to identify the specific races against which the genotype shows resistance, which can help in gene postulation. The susceptible variety “Paven- 76,” the moderately susceptible variety “Kingbird,” the moderately resistant variety “Alidoro,” and the resistant variety “Hedasie” were included as controls (Table 2).

Table.2. Spring wheat genotypes included in the study at Holetta, during 2022 and 2023 Cropping season

| Genotype | Pedigree | Origin | Reason for including in this study and *Stb* genes | Reference |
| --- | --- | --- | --- | --- |
| BIQA | ETBW 6095 | EIAR | *Has Stb* (2, 3,5,7,8,9,14,18) | [25] |
| Meraro | *M/4/HAR 1709/ 3/M//24/E* | EIAR | *Has Stb* (5,6,7,8,11,13,16,17) | [25] |
| Alidoro | *HK-14-R251* | EIAR | *Has Stb* (2,3,4,5,6,7, 8,9, 11, 14,16,17,18) | [25] |
| Madda walabu | *TI/3/Fn/Th/Nar59 *2/4/Bol'S'* | EIAR | *Has Stb* (2,3,4,5,6, 8,9,13, 14,16,17,18) | [25] |
| Hulluka | *UTQUE96/3/PYN/BAU//MILAN* | OARI | *Has Stb* (3,4,5,7,8,13) | [25] |
| Kingbird | TAM200/TUI/6/PVN//CAR422/ANA/5/BOW/CROW//BUC/PVN/3/YR/4/TRAP#1 | EIAR | *Has Stb* (1,2,3,4,5,7, 8,11,13 ,17) | [25] |
| Danda’a | *KIRITATI//2*PBW65/2*SERI.1B* | EIAR | *Has Stb* (2,3,4,7,9,13,14,18) | [25] |
| ET-13A2 | *UQ 105 SEL. X ENKOY* | EIAR | *Has Stb* (3,4,5,6,9,13,14,) | [25] |
| Pavon-76 | *CMl399-D-4M-3Y-lM-l Y-lM-QY* | Mexico | *Has Stb* (3,5,6,8,9,11,12, 13, 14,17,18) | [25] |
| Sofumar | *LIRA 'S'/TAN"S"* | OARI | *Has Stb* (2,3,7,8,9,13,14,17) | [25] |
| KI 6295-4A | *Romany X GB-GAMENYA* | EIAR | *Has Stb* (1,2,3,5,8,9,11,13, 14,16,17,18) | [25] |
| K 6290 Bulk | *(AF.MAYOXGEM)XROMANY* | EIAR | *Has Stb* (2, 3,5,7,8,9,14,18) | [25] |
| Hoggana | *PYN/BAU//MILAN* | EIAR | *Has Stb* (5,6,7,8,11, 13,16,17) | [25] |
| Salamouni | NA | Canada | Stb13 + Stb14 | [32] |
| 6B662 | NA | Brazil | Differential line tan spot | [33] |
| Coulter | DT-188/Dt-224//DT-182 | Israel | ** | [34] |
| Erik | Kitt/2/Waldron/Era | USA/ HRSW-KS | ** | [34] |
| Glenlea | Pemina*2/Bage/2/CB100 | CIMMYT | Differential line for tan spot | [33] |
| ND-495 | Justin*2/3/ND259/Conley//ND112 | Uruguay | ** | [34] |
| Veranopolis | K-44430; PI-214401; PI-297008; AFRC-5645 | Ammiad, Israel | Has *Stb2 + Stb6* | [35, 36] |
| Israel 493 | NA | Iran | Has the *Stb3* and *Stb6* genes | [[35]](https://bsppjournals.onlinelibrary.wiley.com/doi/10.1111/j.1365-3059.2004.01052.x#b16) |
| Tadina | PI-494096; AUS-24432; AUS-23036 | USA |  | [37, 38] |
| Shafir | K-62169 | CIMMYT | *Stb6* | [[39]](https://bsppjournals.onlinelibrary.wiley.com/doi/10.1111/j.1365-3059.2004.01052.x#b26) |
| Estanzuela Federal | NA | Brazil | *Stb7* | [40] |
| KK4500 | NA | CIMMYT | Has the *Stb* (6,7,10,12) | [38] |
| KM7 | NA | CIMMYT | *Stb16* | [41] |
| Gondo/CBRD | NA | USA | ** | [34] |
| BR 18 | NA | CIMMYT | ** | [34] |
| Catbird | NA | CIMMYT | Used as source of resistance by CIMMYT | [42] |
| BR 34 | ALZ110 / 2* IAS54 /6/ TP /4/ TZPP /SON64 | CIMMYT | ** | [34] |
| Frontana | NA | CIMMYT | Veranopolis and KK4500 are derived from Frontana | [**[43]**](https://bsppjournals.onlinelibrary.wiley.com/doi/10.1111/j.1365-3059.2004.01052.x#b7) |
| Mutus | NA | Brazil | ** | [34] |
| Murga | NA | CIMMYT | Stb16 | [34] |
| Blouk #1 | NA | CIMMYT | ** | [34] |
| MP 4106 | Chiba//PRLII/cM65531/3/SKAUZ/BAV92 | CIMMYT | ** | [34] |
| CIANO T79 | CM31678-R-4Y-2M-21-0M | Mexico | ** | [34] |
| Line | SOKOLL//W15.92/WBLL1 | CIMMYT | ** | [34] |
| Line | WAXWING*2/CIRCUS | ICSISA SB6737 | ** | [34] |
| Hidasie | ANAC/3/PRL/SARA//TSI/VEE#5/4/CROC-1/AE.SQUAROSA(224)//OPATTA | CIMMYT | Has good resistance to STB |  |
| Line | ETW17-86 | EIAR | ** |  |
| Line | ETW17-221 | EIAR | ** |  |
| Line | ETW17-246 | EIAR | ** |  |
| Line | ETW17-85 | EIAR | ** |  |
| Line | ETW17-115 | EIAR | ** |  |
| Line | ZERBA-6/FLAG-6/3/TAM200/PASTOR//TOBA97 | EIAR | ** |  |

**EIAR:-Ethiopian Institute of Agricultural Research,** CIMMYT:- International Maize and Wheat Improvement Center, NA= Not available; **= the specific *Stb* genes were not clearly defined

**Sowing and experimental design**

The research was conducted using a randomized complete block design, which included three replications. The experimental area underwent thorough tillage, being plowed three times prior to sowing, and planting rows were established with hand-pulled row maker. Seeds were sown using the hand drilling technique at a depth of approximately five cm. Spring wheat genotypes were planted in 28 of June, in accordance with the recommended sowing period for spring wheat at Holetta [44]. Each plot consisted of four rows, each measuring one meter in length and spaced 20 cm apart, with a seeding rate of 150 kg per hectare [44]. Inorganic fertilizers were uniformly applied across all plots at a rate of 100 kg per hectare of di-ammonium phosphate (DAP) and 150 kg per hectare of urea, following the recommendations for spring wheat cultivation. The entire amount of DAP and half of the urea were applied at the time of planting, while the remaining half of the urea was added during the mid-tillering phase. Hand weeding was performed to manage weed growth in the experimental field [44].

**Data collection**

Disease variable, including severity and pycnidia coverage, were recorded starting from the early grain filling stage of wheat development [45], which is generally when the disease begins under Holetta conditions. This assessment employed a modified version of the double-digit scoring system (00-99) created by Saari and Prescott, which is based on a severity scale for evaluating foliar diseases in wheat [46, 47]. Visual assessments of the disease were quantified by determining the percentage of leaf area showing necrosis (N) a visible symptom of significant tissue injury and the extent of pycnidia coverage (PC) which measures the percentage of dead and/or affected leaf area covered by pycnidia, the asexual reproductive structures of *Z. tritici*. From each plot five plants were randomly selected from each of the four corners and the center in an X-shaped pattern, totaling 25 plants for estimation of diseases severity. The scoring system for severity is as follows: 10% coverage corresponds to a score of 1, 20% coverage to 2, 30% coverage to 3, 40% coverage to 4, 50% coverage to 5, 60% coverage to 6, 70% coverage to 7, 80% coverage to 8, and 90% coverage to 9. These measurements were combined to calculate the overall disease severity using the following formula:

$$Disease severity \left( \% \right)=\left( \left( \frac{D1}{Y1} \right)*\left( \frac{D2}{Y2} \right) \right)*100$$

Where D_1_:- represents disease progression relative to plant height, this metric usually evaluates how far up the plant the disease has spread, often shown as a percentage of the plant's total height. The greater the disease reaches especially when it affects the flag leaf, which plays a key role in yield the more serious the damage. D1 measures this important vertical progression.  D_2_:- reflects the diseased leaf area, assesses the proportion of leaf surface affected by lesions and/or dead tissue. This provides the most straightforward indication of tissue damage and reduced ability for photosynthesis. Y_1_:- Maximum Scale Value for D_1,_ which is the maximum possible score for the vertical progression of the disease, Y_2_:-Maximum Scale Value for D_2_, this is the maximum possible score for the intensity of the diseased leaf area [46, 47].

To assess disease progression over time, we record severity data weekly for six weeks, beginning at the onset of the disease and ends when the flag leaf infection reached 70% on susceptible genotype. The area under the disease progress curve (AUDPC) serves as a quantitative measure of disease intensity over a specified period. This is accomplished by segmenting the time variable (in days) and calculating the average disease intensity for each interval between consecutive time points [48, 25]. The AUDPC is calculated using the following formula:

$$AUDPC \%/.day=\sum_{i=1}^{n-1} \frac{y_{i}+y_{i+1}}{2}\times(t_{i+1}-t_{i})$$

Where, “Y_i_” denotes the assessment of a disease at the i^th^ observation, “t_i_” signifies the time, expressed in days, associated with the i^th^ observation, and “n” represents the overall count of observations.

All essential agronomic data were collected from the middle two rows within each plot. The data collection was conducted on both a plant and plot basis. For the analysis of plant-based data, a random selection of ten plants from each plot was made to evaluate a range of characteristics. These characteristics included the number of tillers per plant, the number of kernels per spike, the spikelet count per spike, as well as measurements of plant height (cm), and spike length (cm). Additionally, further data were gathered on the density of productive tillers per square meter, the weight of a thousand kernels (grams), and the grain yield (t/ha) on a plot-specific basis.

The resistance levels of the genotypes were classified based on the mean percentage of pycnidia coverage obtained from six score made starting from disease on set to 70% of the flag leaf infection recorded on susceptible genotype, which was estimated and grouped using a 0–5 scale as described by Eyal, and Brown [49] and Adhikari *et al*. [50]. Where; 0 =Immune, no pycnidial formation, no symptoms or occasional hypersensitive fleck. 1 = represents average pycnidia density in a lesions (0-5%); 2 = denotes average density of pycnidia in many or most lesions, usually unevenly distributed (6–15%); 3 = signifies even distribution of pycnidia at moderate density over most of the lesion (16–30%); 4= indicates a high concentration of pycnidia present across the majority of lesions (31-40%), while a score of 5 represents the highest average density of pycnidia (41-50%). The Disease ranging from 0 to 1 categorize the genotype as resistant (R), a score of 2 denotes moderately resistant (MR), a score of 3 signifies moderately susceptible (MS), and scores of 4 to 5 are classified as susceptible (S).

**Statistical analysis**

The agro-morphological data, including disease severity and AUDPC, were subjected to variance analysis using SAS software version 9.2 [51], following the standard procedures outlined by Gomez and Gomez [52]. Severity percentage data were transformed using arcsine transformation [53]. The validity of further statistical analyses, such as ANOVA, was ensured by using Bartlett's test to determine whether the error variances for distinct genotypes across different attributes were comparable. ANOVA and many other statistical tests need the assumption of homoscedasticity, which is checked by this test. The differences between treatment means were compared using the least significant difference (LSD) test at a 5% level of significance when the ANOVA indicated a significant difference among genotypes. Diversity in disease resistance among the genotypes was assessed through cluster, correspondence and principal component analysis based on the agronomic trait, disease severity and AUDPC, utilizing R statistical software. The relationship between severity and AUDPC with agronomic traits was analyzed using SPSS statistical software. The principal components with eigenvalues above 1 were selected and used to interpret the results

**ETHICS S TATEMENT:** The study was approved by review and Ethics Committee, Harumaya University, Agriculture and Environmental Sciences School of Plant Sciences in February, 2022

**Results**

**Reaction of spring wheat genotype to *Zymoseptoria tritici***

The comprehensive analysis of variance conducted over two years revealed statistically significant differences (P ≤ .0001) among the years, genotypes, and the interaction between genotype and year. The significance of severity, AUDPC, Plant height, Number of productive tillers per plant, spick length, Number of seeds per spick, thousand kernel weights, and grain yield indicates a substantial level of genetic variability concerning disease resistance and yield characteristics. The interaction between genotype and year demonstrated notable values for severity, Area Under the Disease Progress Curve (AUDPC), plant height, number of productive tillers, spick length, number of seeds per spike, and thousand-kernel weight across the two years and among the various genotypes (Table 3 and 4). This finding suggests that the performance of these traits was inconsistent across the years.

Table.3. Analysis of variance of the effect of spring wheat genotype on resistance to STB at Holetta, Ethiopia, during 2022 and 2023 cropping season

| Trait | Factor | SS | Mean Square | Pr > F | %SS | h_2_^2^ % |
| --- | --- | --- | --- | --- | --- | --- |
| Severity | Genotype | 3.233 | 0.074 | <.0001 | 70.94 | 0.7094 |
|  | year | 0.562 | 0.562 | <.0001 | 12.33 |  |
|  | Residual | 0.763 | 0.0043 |  | 16.74 |  |
| AUDPC | Genotype | 56508295.41 | 1284279.44 | <.0001 | 71.32 | 0.7132 |
|  | year | 8214937.84 | 8214937.84 | <.0001 | 10.37 |  |
|  | Residual | 14508570 | 81508.8 |  | 18.31 |  |

Notes: SS- a sum of squares; MS- mean squares; h_b_^2^- broad-sense heritability index.

The ANOVA findings indicate that genotype is the main factor contributing to variation in resistance to STB within the 45 spring wheat collection, accounting for 70.94% of the total variance in Severity and 71.32% in AUDPC. All genotypes showed high broad-sense heritability values (0.7094 for severity and 0.7132 for AUDPC) for these traits, suggesting that resistance to STB can be enhanced through breeding.

**Genotype by year interaction**

The average disease severity was higher in 2022 (0.88) than in 2023 (0.79). In 2022, the weather at Holetta was cool and wet, ideal for fungal disease growth, while 2023 was relatively hot and dry. This affected both the spring wheat and the pathogen. The spring wheat genotype “Catbird” remained consistently susceptible over the years; however, the genotypes “Shafir” shifts from susceptible to moderately susceptible whereas, “Salamouni” shifted from resistant to moderately resistant in 2022 and 2023, respectively. Since “Shafir” and “Salamouni” are differential lines for STB and possess major resistance genes (Salamouni: *Stb13* & *Stb14*; Shafir: *Stb6*), these *Stb* genes provide qualitative (major gene) resistance to STB. Nevertheless, their effectiveness is strongly influenced by genotype-by-environment interactions, making the resistance often specific to certain pathogen races and dependent on environmental conditions in the field. The observed changes in ranking for these differential lines may be attributed to shifts in the dominant pathogen races during those years.

Since each year showed considerable variation and an interaction between genotype and year, the following data were evaluated separately: severity, AUDPC, plant height, number of productive tillers, spick length, number of seeds per spick, and thousand kernel weight (Table 4). During 2022, the spring wheat genotypes showed a significant variation (P < 00.0001) in Severity, AUDPC, plant height, number of productive tillers, spick length, number of seeds per spick, and thousand kernel weights.

Table.4. Combined analysis of variance over the year for STB Severity, grain yield, and yield-related characters of spring wheat genotype grown at Holetta during the 2022 and 2023 growing seasons

| Trait | MSG (44) | MSY (1) | MSGY (44) | MSB | MSE | Mean | Cv. | R^2^ | LSD |
| --- | --- | --- | --- | --- | --- | --- | --- | --- | --- |
| Severity | 0.0735^**^ | 0.5618^**^ | 0.068^**^ | 0.0299^**^ | 0.00428 | 0.83 | 7.86 | 0.90 | 0.0746 |
| AUDPC | 1284279.44^**^ | 8214937.84^**^ | 1236429.11^**^ | 468196.27^**^ | 81508.8 | 2317.52 | 12.32 | 0.89 | 325.28 |
| Plant height | 1041.048^**^ | 31948.03^**^ | 602.541^**^ | 167.315^**^ | 30.2661 | 92.063 | 5.98 | 0.95 | 6.268 |
| Number of tillers per plant | 0.0819^**^ | 12.752^**^ | 0.0704^**^ | 0.2326^**^ | 0.0677 | 2.590 | 10.05 | 0.62 | 0.2965 |
| spick length | 4.3226^**^ | 428.65^**^ | 4.2447^**^ | 0.2028^**^ | 0.3393 | 7.253 | 8.03 | 0.93 | 0.6636 |
| Number of seeds per spick | 0.9343^**^ | 0.60314^**^ | 0.8623^**^ | 0.4579^**^ | 0.1959 | 6.481 | 6.83 | 0.70 | 0.5043 |
| Thousand kernel weight | 228.58199^**^ | 11774.4^**^ | 249.1234^**^ | 126.693^**^ | 163.824 | 33.515 | 38.19 | 0.53 | 14.583 |
| Yield tons/hectare | 1.999^**^ | 0.641^**^ | 0.015^NS^ | 0.01^NS^ | 0.05 | 2.43 | 9.03 | 0.91 | 0.2503 |

*, ** Significant at p ≤ 0.05, and p ≤ 0.01 probability level respectively, () parenthesis indicate degrees of freedom.

MSG= Mean Squares of Genotype, MSY= Mean squares of year, MSGY = Mean square of genotype x year interaction, MSE = Mean squares of error, MSB= Mean squares of block, CV = Coefficient of variation.

Eleven genotypes showed a susceptible reaction and significantly high severity and AUDPC, while eleven genotypes showed a resistance reaction and significantly low severity and AUDPC. The most resistant genotype was Gondo, Israel-493, Salamouni, Pavon-76, Blouk#1, Sofumar, Coulter, Mutus, Erik and 6B662, while the most susceptible was “Etw17-246, Tadina, Etw17-86, Ki6295-4A, Glenlea, and Chiba //PRLII/cM65531/3/SKAUZ/BAV92, Catbird, Hoggana, CIANO T79, Veranopolis, Estanzuela Federal, ZERBA-6/FLAG-6/3/TAM200/PASTOR//TOBA97 and Shafir.

In 2023, there was a highly significant difference (P < 00.0001) in the spring wheat genotypes' severity, AUDPC, plant height, number of productive tillers, spick length, number of seeds per spick, and thousand kernel weights (Table 4). A resistance reaction was shown by eleven genotypes with considerably low severity and AUDPC, while a susceptible reaction was shown by fourteen genotypes with significantly high. The most resistant genotypes were “Etw17-115, Gondo, Israel-493, Blouk#1, Sofumar, Coulter, Mutus, Erik, and 6B662," while the most susceptible genotypes were " Etw17-246, Tadina, Etw17-86, Ki6295-4A, Glenlea." The results indicated that the spring wheat genotypes response was 22.2%, with 20% classified as resistant. Additionally, 26.7% and 24.4% were categorized as moderately resistant, while 22.2% and 24.4% were deemed moderately susceptible and finally, 28.8% and 31.1% of the genotypes were found to be susceptible during the 2022 and 2023 cropping seasons, respectively (Table. 5).

Of the 45 genotypes evaluated over two years, in 2022, Shafir showed the highest susceptibility, recording a leaf blotch score of 73.49% and pycnidia coverage of 30.95%, while Salamouni demonstrated the greatest resistance, with a leaf blotch severity of 47.46% and pycnidia coverage of 4.35%. In 2023, “Catbird” and “ZERBA-6/FLAG-6/3/TAM200/PASTOR//TOBA97” were the most vulnerable, showing leaf blotch severity (71.71%) and pycnidia coverage (45.47% and 35.36%), respectively. Meanwhile, “6B662” demonstrated the greatest resistance, achieving a leaf blotch severity score of 9.99% and pycnidia coverage score of 1.21% (Table 5). The data show different types of infections in various spring wheat genotypes and during different growing seasons. Identifying genotypes that reliably display resistance year-round is essential for resistance breeding and as sources of resistance

Table.5. Leaf blotch severity (LBS) and infection types (ITs) and Pycnidia coverage (P), of the bread wheat genotypes to STB (2022 and 2023) at Holetta, Ethiopia.

| Spring wheat Genotypes | 2022 | | | 2023 | | |
| --- | --- | --- | --- | --- | --- | --- |
|  | %LBS | P | IT | %LBS | P | IT |
| Etw17-246 | 50.46^MNO^ | 35.66^A^ | S | 68.97^ABCD^ | 38.56^AB^ | S |
| Tadina | 65.22^ABCDEFG^ | 35.66^A^ | S | 69.89^ABC^ | 45.47^A^ | S |
| Etw17-86 | 46.46^NO^ | 35.66^A^ | S | 69.89^ABC^ | 45.47^A^ | S |
| Ki6295-4A | 66.16^ABCDE^ | 35.66^A^ | S | 69.89^ABC^ | 34.71^ABC^ | S |
| Glenlea | 49.46^MNO^ | 35.66^A^ | S | 60.38^DEFGHIJ^ | 26.43^CDE^ | MS |
| Chiba//PRLII/cM65531/3/SKAUZ/BAV92 | 69.89^ABC^ | 35.66^A^ | S | 65.22^ABCDEF^ | 42.69^A^ | S |
| Catbird | 68.97^ABCD^ | 35.66^A^ | S | 71.71^A^ | 45.47^A^ | S |
| Hoggana | 63.29^BCDEFGHIJ^ | 35.66^A^ | S | 66.16^ABCDE^ | 30.95^BCD^ | S |
| CIANO T79 | 59.40^DEFGHIJKL^ | 35.66^A^ | S | 61.36^BCDEFGHI^ | 30.95^BCD^ | S |
| Veranopolis | 48.46^MNO^ | 35.66^A^ | S | 69.89^AB^ | 42.49^A^ | S |
| Estanzuela Federal | 63.29^BCDEFGHIJ^ | 35.66^A^ | S | 69.89^ABC^ | 42.49^A^ | S |
| ZERBA-6/FLAG-6/3/TAM200/PASTOR//TOBA97 | 53.46^HIJKLMNO^ | 35.66^A^ | S | 71.71^A^ | 35.66^ABC^ | S |
| Shafir | 73.49^A^ | 30.95^A^ | S | 59.40^EFGHIJK^ | 22.98^DE^ | MS |
| Etw17-221 | 55.45^GHIJKLMNO^ | 27.32^AB^ | MS | 64.26^ABCDEFG^ | 30.95^BCD^ | S |
| WAXWING*2/CIRCUS | 57.43^EFGHIJKLM^ | 27.32^BC^ | MS | 69.89^AB^ | 30.95^BCD^ | S |
| Meraro | 50.46^LMNO^ | 27.32^BC^ | MS | 38.56^OP^ | 18.14^EF^ | MS |
| BR 34 | 65.22^ABCDEFG^ | 22.98^C^ | MS | 57.43^EFGHIJK^ | 18.14^EF^ | MS |
| Murga | 59.40^DEFGHIJKLM^ | 22.98^C^ | MS | 59.40^EFGHIJK^ | 27.32^CDE^ | MS |
| Kk4500 | 66.16^ABCDEFG^ | 22.98^C^ | MS | 62.33^ABCDEFGH^ | 18.14^EF^ | MS |
| Biqa | 56.44^EFGHIJKLM^ | 22.98^C^ | MS | 51.46^IJKLM^ | 18.14^EF^ | MS |
| Hidasie | 61.36^CDEFGHIJKL^ | 22.98^C^ | MS | 57.43^EFGHIJK^ | 26.43^CDE^ | MS |
| SOKOLL//W15.92/WBLL1 | 64.26^ABCDEFG^ | 22.98^C^ | MS | 52.46^IJKLM^ | 18.14^EF^ | MS |
| K6290 Bulk | 63.29^ABCDEFGH^ | 22.98^C^ | MS | 68.97^ABCD^ | 26.43^CDE^ | MS |
| Frontana | 57.43^EFGHIJKLM^ | 14.45^D^ | MR | 46.46^LMNO^ | 10.50^GH^ | MR |
| Etw17-85 | 52.46^IJKLMNO^ | 10.50^DE^ | MR | 53.46^HIJKLM^ | 18.14^EF^ | MS |
| Alidoro | 48.46^MNO^ | 10.50^DE^ | MR | 55.45^GHIJKLM^ | 10.50^GH^ | MR |
| Nd-495 | 68.97^ABCD^ | 10.50^DE^ | MR | 46.46^LMNO^ | 10.50^GH^ | MR |
| Madda walabu | 57.43^EFGHIJKLM^ | 10.50^DE^ | MR | 55.45^FGHIJKL^ | 10.50^GH^ | MR |
| BR 18 | 64.26^ABCDEFG^ | 10.50^DE^ | MR | 50.46^JKLM^ | 14.45^FG^ | MR |
| KM7 | 50.46^MNO^ | 10.50^DE^ | MR | 60.38^CDEFGHI^ | 14.45^FG^ | MR |
| Hulluka | 61.36^CDEFGHIJKL^ | 10.50^DE^ | MR | 39.54^NOP^ | 10.50^GH^ | MR |
| Danda’a | 67.11^ABCDE^ | 10.50^DE^ | MR | 46.46^LMNO^ | 10.50^GH^ | MR |
| Kingbird | 65.22^ABCDEFG^ | 10.50^DE^ | MR | 40.52^NOP^ | 10.50^GH^ | MR |
| ET-13A2 | 52.46^JKLMNO^ | 7.11^EF^ | MR | 49.46^KLMN^ | 14.45^FG^ | S |
| Etw17-115 | 50.46^LMNO^ | 7.11^EF^ | MR | 11.76 ^SR^ | 2.54^JK^ | R |
| Gondo | 51.46^KLMNO^ | 4.35^FG^ | R | 8.73 ^S^ | 0.25^K^ | R |
| Israel-493 | 66.16^ABCDEF^ | 4.35^FG^ | R | 28.22 ^Q^ | 2.54^JK^ | R |
| Salamouni | 47.46^NO^ | 4.35^FG^ | R | 54.45^HIJKLM^ | 7.11^HI^ | MR |
| Pavon-76 | 72.61^AB^ | 4.35^FG^ | R | 39.54 ^NOP^ | 4.76^IJ^ | MR |
| Blouk#1 | 65.22^ABCDEFG^ | 2.23^G^ | R | 13.08 ^SR^ | 1.21^JK^ | R |
| Sofumar | 53.46^HIJKLMNO^ | 2.23^G^ | R | 45.47 ^MNO^ | 2.54^JK^ | R |
| Coulter | 51.46^KLMNO^ | 2.23^G^ | R | 18.14 ^R^ | 2.54^JK^ | R |
| Mutus | 63.39^BCDEFGHI^ | 2.23^G^ | R | 35.66^PQ^ | 2.54^JK^ | R |
| Erik | 62.33^BCDEFGHIJK^ | 2.23^G^ | R | 13.76 ^SR^ | 1.21^JK^ | R |
| 6B662 | 55.45^FGHIJKLMN^ | 2.23^G^ | R | 9.99 ^S^ | 1.21 ^JK^ | R |
| R^2^ | 0.68 | 0.96 |  | 0.942163 | 0.93 |  |
| CV | 7.69 | 10.69 |  | 7.85 | 16.02 |  |
| Mean | 0.88 | 0.42 |  | 0.79 | 0.43 |  |
| LSD | 0.1096 | 0.07 |  | 0.10 | 0.11 |  |

**Cluster analysis**

The spatial relationships among the various cluster centroids of spring wheat genotypes are illustrated in Fig. 1. A comprehensive analysis of the dendrogram revealed the presence of eight clusters, each demonstrating a minimum similarity level of 1500 based on Euclidean distance within the UPGMA clustering framework using severity and AUDPC. These clusters were categorized into two distinct groups: Group A and Group B. Group A consisted of three clusters—Cluster I, Cluster II, and Cluster III— while Group B was represented by Five clusters, Cluster IV, V, VI, VII, VIII (Fig. 1).

Cluster I included five genotypes, making up 12.11% of the total. This group contains the cultivar "Estanzuela Federal," which is used as a differential line for STB and carries the resistance genes *Stb7* and *Stb12*. However, under the conditions in Holetta, Ethiopia, it has shown susceptibility. Therefore, the genotypes in this cluster likely possess these resistance genes but display a susceptible response. Cluster II comprises six genotypes, accounting for 13.33% of the total. Among them are two differential lines: “KK4500” (carrying *Stb6*, *Stb7*, *Stb10* and *Stb12*) and “Shafir” (carrying *Stb6*). Both show a moderately susceptible reaction, suggesting that other cultivars in this cluster may also carry these *Stb* genes. Cluster III contains fifteen genotypes, representing 33.33% of the total, including three known differential lines: Veranopolis (*Stb2* and *Stb6*), KM7 (*Stb16*), and Murga (*Stb16*), all of which carry resistance genes. These genotypes exhibit responses ranging from susceptible to moderately resistant, indicating that other genotypes in this cluster might also have resistance genes. Clusters IV, V, and VI together include twelve genotypes, or 26.7% of the total, with no differential lines present. The genotypes in these clusters show resistant to moderately resistant reactions. Cluster VII has two genotypes, one being the differential line Israel- 493 (*Stb3* and *Stb6*), which displays resistance. The other genotype, Meraro, also carries *Stb6* but shows a moderately susceptible reaction. This suggests that the resistance in Israel- 493 is likely due to the *Stb3* gene. Finally, Cluster VIII consists of five genotypes, including the differential line Salamouni (*Stb13* and *Stb14*). Both Salamouni and the other cultivars in these group exhibit resistant reactions, implying that these genotypes may carry *Stb* genes similar to those found in Salamouni (Fig.1) The resistance genotypes identified in the study (Blouk #1, 6B662, Coulter, Erik, Gondo, and ETW17-115) exhibit resistance to the pathotypes that affect the major *Stb* genes found in Veranopolis (*Stb2* and *Stb6*) and Estanzuela Federal (*Stb7*) (Fig 1).

Table.6. Characteristics of Cluster Based on Mean, Standard Deviation and Standards Error of Mean

| Number of cluster | Variable | Mean | Std.Dve | SEM |
| --- | --- | --- | --- | --- |
| cluster I (N=5) | Severity | 68.16 | 1.398 | 0.625 |
|  | AUDPC | 2948.87 | 58.697 | 26.25 |
|  | Pycnidia | 4.33 | 0.204 | 0.091 |
| cluster II (N=6) | Severity | 64.75 | 1.491 | 0.609 |
|  | AUDPC | 2762.67 | 43.97 | 17.95 |
|  | Pycnidia | 3.44 | 0.43 | 0.176 |
| cluster III (N=15) | Severity | 58.42 | 1.86 | 0.480 |
|  | AUDPC | 2471.47 | 86.79 | 22.41 |
|  | Pycnidia | 3.09 | 0.89 | 0.23 |
| cluster IV (=3) | Severity | 28.95 | 1.28 | 0.737 |
|  | AUDPC | 1289.17 | 48.93 | 28.25 |
|  | Pycnidia | 0.94 | 0.35 | 0.20 |
| cluster V (N=3) | Severity | 35.72 | 1.81 | 1.04 |
|  | AUDPC | 1547.39 | 89.11 | 51.45 |
|  | Pycnidia | 0.78 | 0.096 | 0.06 |
| cluster VI (N=6) | Severity | 53.46 | 1.699 | 0.694 |
|  | AUDPC | 2216.67 | 57.34 | 23.41 |
|  | Pycnidia | 2.111 | 0.49 | 0.20 |
| cluster VII (N=2) | Severity | 45.88 | 1.745 | 1.234 |
|  | AUDPC | 1898.17 | 28.05 | 19.83 |
|  | Pycnidia | 2.083 | 1.295 | 0.92 |
| cluster VIII (N=5) | Severity | 50.013 | 0.748 | 0.33 |
|  | AUDPC | 2058 | 55.97 | 25.03 |
|  | Pycnidia | 1.5 | 0.5 | 0.224 |

**Mean= cluster centroid, Std.Dve.=standard deviation, SEM= Standard Error of the Mean, Roman numbers from I to VIII = cluster numbers, N=number of genotype in that cluster,

Cluster (I) has high mean values for severity, AUDPC, and Pycnidia, indicating it contains the most susceptible spring wheat genotypes. Conversely, Cluster (IV) has the lowest means for these variables, suggesting that the spring wheat in this group is highly resistant.

Cluster (VIII) has the lowest standard deviation for severity (0.75), Cluster V has the lowest standard deviation for Pycnidia (0.096), and Cluster (VII) has the lowest standard deviation for AUDPC (28.05). Whereas, the highest standard deviations are found in Cluster (VIII) for severity (1.86), Cluster (VII) for Pycnidia (1.295), and Cluster (V) for AUDPC (89.11). This suggests that spring wheat genotypes in Cluster (VIII) for severity, Cluster (V) for Pycnidia, and Cluster (VII) for AUDPC are tightly grouped with data points close to the cluster mean (high internal similarity). On the other hand, genotypes in Cluster (VIII) for severity, Cluster (VII) for Pycnidia, and Cluster (V) for AUDPC have the highest standard deviations, indicating their data points are widely spread, making these clusters less compact and more diverse internally (Table 6). According to the result, most clusters have standard error of the mean (SEM) values close to zero for severity and Pycnidia, showing that their means are estimated with high reliability.

B

A

Fig.1. UPGMA clustering of 45 bread wheat genotypes reaction to STB under natural infection conditions, Holetta, Ethiopia (2022/23)

**CI-CVIII = Roman numbers showing cluster; Metric variables are:- AUDPC and Severity

UPGMA (un-weighted pair group method with arithmetic mean) analysis revealed that the genotypes were classified into eight distinct clusters. The resistant and susceptible genotypes were organized into separate groups. Notably, the genotypes found in cluster Clusters IV, V, and VI demonstrated resistance to STB, whereas the other members of the clusters exhibited varying levels of susceptibility, ranging from susceptible to moderately resistant. With the identification of promising genotypes through the STB resistance screening experiment conducted on spring wheat, further research can be pursued by selecting the most beneficial genotypes. These chosen genotypes may significantly contribute to the development of varieties that are resistant to STB.

**Correlation and scatter plot matrix**

The epidemiological variables, Severity and AUDPC, were found to have a significant negative correlation with plant height, number of productive tillers per plant, spike length, thousand kernel weight, and grain yield. As the disease severity percentage increased, AUDPC values also raised. This demonstrates the detrimental impact that the prevailing STB had on the traits examined in the spring wheat genotypes evaluated (Table 7).

Table.7. Pearson Correlation result of diseases, yield and yield related trait on 45 spring wheat genotypes evaluated during 2022 and 2023 cropping season

|  | Severity | AUDPC | PH | NTPP | SL | NSPS | TKW | Grain Yield |
| --- | --- | --- | --- | --- | --- | --- | --- | --- |
| Severity | 1 | .998^**^ | -.424^**^ | -.045 | -.194^*^ | -.138 | -.321^**^ | -.063 |
| AUDPC | .998^**^ | 1 | -.424^**^ | -.047 | -.192^*^ | -.142 | -.320^**^ | -.062 |
| PH | -.424^**^ | -.424^**^ | 1 | .176^*^ | .156 | .183^*^ | .427^**^ | .077 |
| NTPP | -.045 | -.047 | .176^*^ | 1 | -.114 | -.021 | .101 | .176^*^ |
| SL | -.194^*^ | -.192^*^ | .156 | -.114 | 1 | .251^**^ | .169^*^ | .052 |
| NSPS | -.138 | -.142 | .183^*^ | -.021 | .251^**^ | 1 | .398^**^ | .074 |
| TKW | -.321^**^ | -.320^**^ | .427^**^ | .101 | .169^*^ | .398^**^ | 1 | .067 |
| Grain Yield | -.063 | -.062 | .077 | .176^*^ | .052 | .074 | .067 | 1 |

**. Correlation is significant at the 0.01 level, *. Correlation is significant at the 0.05 level, AUDPC=-Area under diseases progress curve, PH=- plant height, NTPP=- number of productive tiller per plant, SL=- Spick length, NSPS=- number of seed per spick, TKW=- thousand kernel weight,

The relationship between disease and yield-related traits indicates that severity and AUDPC have a clear negative correlation with plant height, number of tillers per plant, spike length, number of seeds per spike, thousand kernel weight, and grain yield (Fig 2). However, the correlation between disease and thousand kernel weight (TKW) is very weak, with correlation coefficients of -0.04 for severity and -0.03 for AUDPC. This means there is essentially no linear relationship between disease severity or AUDPC and TKW. This suggests that while the disease significantly reduces overall yield by decreasing the number of grains, spikes, and tillers, it does not have a significant impact on the average weight of individual kernels produced (Fig. 2).

Fig.2. Scatter plot matrix showing severity and AUDPC relation with yield and yield related trait on 45 spring wheat genotypes evaluated during 2022 and 2023 cropping season

**Correspondence analysis**

The Correspondence Analysis (CA) bi-plot illustrates the relationships between the evaluated wheat genotypes and various traits. Dimension 1 account for 90.2% of the variation, while Dimension 2 explains 6.5%, together capturing 96.5% of the total variation. The correspondence analysis graph reveals three distinct trait groups: resistant genotypes, high-yield potential genotypes, and susceptible genotypes. Resistant or low-disease genotypes include Blouk #1, Erik, Coulter, ETW17-115, 6B662, and Gondo. These genotypes are positioned opposite the Severity, AUDPC and Pycnidia coverage traits, indicating that they exhibit the highest resistance, with low disease severity and slow disease progression during the trial. High-yield potential genotypes, such as 6B662, Coulter, Eric, Gondo and ETW17-115 are closely associated with grain yield and number of seed per spick (NSPS), number of productive tiller per plant (NTPP), Thousand Kernel Weight (TKW), and spick length (SL) suggesting they are among the highest-yielding genotypes. Susceptible genotypes such as Catbird, BR 18, Chiba//PRLII/cM65531/3/SKAUZ/BAV92, KI6295-4A, Hoggona, Danda’a, CIANOT79, WAXWING*2/CIRCUS, Chibia, and Tadina are located near the Severity, AUDPC and Pycnidia traits, indicating they are the most vulnerable to STB.

Yield versus Disease Trade-off: Traits linked to high yield—such as yield per hectare, thousand kernel weight, and number of seeds per spike are positioned in the upper-left quadrant (negative on Dimension 1). In contrast, disease-related traits like severity and AUDPC cluster near the center on the positive side of Dimension 1. This pattern suggests that the lines with the highest yield potential (Blouk #1, Erik, Coulter, ETW17-115, 6B662, and Gondo) tend to be both highly resistant and high yielding, indicating a possible positive correlation between disease resistance and yield traits within the genotype studied. Plant Architecture: - Plant height (PH) and spike length (SL) are closely associated with the high-performing genotype 6B662 and are found in the upper-left quadrant. This implies that the tallest plants with the longest spikes consistently rank among the top performers in terms of both resistance and yield (Fig 3).

Fig.3. Ordination of nine variables by correspondence analysis. The active variables in the columns are: Severity, AUDPC, PH=plant height, TKW=thousand kernel weight, SL=spick length, NTPP=number of productive tiller per plant, P=pycnidia coverage and grain yield, the rows stands for spring wheat genotypes.

**Principal component analysis**

PC1 is the dominant component and shows the clearest separation of genotypes (Table 8). Negative PC1 (Left Side): High Yield and Resistance. This side is strongly associated with desirable traits such as yield (tons/ha), spike length (SL), plant height (PH), number of tillers per plant (NTRP), and thousand kernel weight (TKW). Genotypes associated with the above trait include 6B662, Coulter, Gondo, ETW17-115, Erik, Blouk #1, and Meraro. These genotypes demonstrate a combination of high yield and likely disease resistance. Positive PC1 (Right Side): High Susceptibility and Severity. This side is strongly associated with disease variables such as Severity, AUDPC, and P. Genotypes linked to high susceptibility include KI6295-4A, WAXWING*2/CIRCUS, ETW17-86, Chiba//PRLII/cM65531/3/SKAUZ/BAV92, Catbird, Hoggona, ZERBA-6/FLAG-6/3/TAM200/PASTOR//TOBA97, Tadina, and Estanzuela Federal. These genotypes are likely to have the lowest yield due to severe disease impact (Fig 4).

Table.8. Importance of components

| Components | PC1 | PC2 | PC3 | PC4 | PC5 | PC6 | PC7 | PC8 | PC9 |
| --- | --- | --- | --- | --- | --- | --- | --- | --- | --- |
| Eigen values | 3.82 | 1.70 | 1.02 | 0.74 | 0.67 | 0.499 | 0.341 | 0.201 | 0.0031 |
| Standard deviation | 1.95 | 1.31 | 1.01 | 0.86 | 0.82 | 0.706 | 0.584 | 0.448 | 0.0560 |
| Proportion of Variance | 0.42 | 0.19 | 0.11 | 0.08 | 0.07 | 0.055 | 0.038 | 0.022 | 0.0004 |
| Cumulative Proportion | 0.42 | 0.61 | 0.73 | 0.81 | 0.88 | 0.939 | 0.977 | 0.999 | 1.000 |

Fig.4. Result of principal component analysis executed on over year diseases, yield and yield related variables of forty five different spring wheat genotypes included in the study, Holetta, Ethiopia.

**Discussion**

In this study, we observed significant differences in the main effects of genotype (G), year (Y), and the G × Y interaction on STB severity, area under the disease progress curve (AUDPC), plant height, yield, and yield-related traits. The interaction between genotype and year is influenced by environmental factors and the genotype response to the prevailing *Z. tritici* races. The notable interaction between genotype and year shows that the wheat lines performed inconsistently because of environmental variations across different years. In particular, years with favorable conditions such as abundant rainfall and extended leaf wetness, which promote STB development likely led to high disease severity in susceptible genotypes, causing their rankings to change compared to years with less favorable conditions. Conversely, dry and hot years tend to reduce disease severity, therefore decreasing differences in disease between susceptible and resistant genotypes. Due to variations in moisture, temperature, and pathogen populations, STB responses vary from year to year [54, 55], which was also observed in our study where STB infection was the highest in 2022 than in the 2023.

The environment influences qualitative and quantitative resistance genes through two primary, interconnected pathways: by modulating the host resistance expression and by affecting the pathogen virulence and dispersal [21]. The field environment directly impacts the wheat plant, altering the expression or effectiveness of the *Stb* genes. Resistance conferred by some *Stb* genes, such as those in “Shafir” and “Salamouni,” can be temperature-dependent. Cool temperature, which is ideal for the fungus, *Stb* gene might provide strong, near-immune resistance but become ineffective resulting in resistance breakdown or only partially effective at higher temperatures [56]. This instability is a major component of the genotype-by-environment variance observed in field trials. Environmental factors, such as water or heat stress, influence the host's growth stage and canopy density. Since *Stb* resistance often functions by prolonging the latent period (the time from infection to visible pycnidia), any factor that prematurely shortens the plant's life span (such as drought) can lead to disease escape, causing a susceptible genotype to appear resistant and thereby masking the true effect of the *Stb* gene [56]. The environment influences the severity and population structure of the pathogen, which directly challenges the *Stb* gene set present in the host spring wheat [15]. Wet, cool conditions (high moisture, low temperature) are optimal for *Z. tritici* spore germination, leaf infection, and spread [57, 58]. In dry, hot years with low disease pressure, even susceptible genotypes may exhibit minimal symptoms, rendering the resistance gene's effect negligible or difficult to measure. Conversely, in wet years with high disease pressure, susceptible genotypes fail drastically, fully testing the effectiveness of the *Stb* gene and resulting in the greatest genotype-by-environment separation (crossover interaction). The environment also controls the pathogen's generation time and mutation rate [59]. The widespread deployment of a single *Stb* gene exerts strong selective pressure, enabling fungal races that have evolved virulence (the ability to overcome the gene) to multiply rapidly [57, 15]. Once a new race overcomes the *Stb* gene, resistance breaks down entirely, and the gene becomes ineffective regardless of temperature or moisture conditions. For breeders, a high genotype-by-environment interaction for traits controlled by qualitative (*Stb*) genes and quantitative trait loci (QTL) necessitates selection for stability or the stacking of multiple genes (gene pyramiding). Gene stacking provides broader resistance, making it much more difficult for the pathogen to overcome the entire set and reducing drastic performance fluctuations caused by single-gene breakdown or environmental variation.

To date, the assessment of wheat genotypes for their resistance to STB has been conducted in both protected and open field conditions, using susceptible checks as controls. However, including differential lines in the set of spring wheat genotypes to be screened is crucial for identifying which *Stb* gene is effective, determining the resistance of specific wheat lines to particular pathotypes, and understanding the infection types. This information enables researchers to postulate the presence of genes in unknown wheat lines [60]. Our study reviled resistance genotypes (Blouk #1, 6B662, Coulter, Erik, Gondo, and ETW17-115) exhibit resistance to the pathotypes that affect the major *Stb* genes found in Veranopolis (*Stb2* and *Stb6*), Shafir (*Stb6*) and Estanzuela Federal (*Stb7*) differential lines. This approach supported by research conducted by Tabib *et al* [60].

Wheat genotypes show different degrees of resistance to STB. The results showed that 24.4% and 25.2% of the genotypes were identified as resistant, whereas 30.4% and 24.4% were deemed moderately resistant. Furthermore, 22.2% and 20.7% were classified as moderately vulnerable, while 22.96% and 29.63% were recognized as vulnerable in the 2022 and 2023 growing seasons. Research in Ethiopia has shown considerable variation in resistance levels among bread wheat, durum wheat, and triticale [22]. Molecular analyses conducted by Tilahun *et al*. [25] revealed significant genetic diversity in resistance among 180 genotypes of Ethiopian bread wheat. Genome-Wide Association Studies (GWAS) performed on Ethiopian wheat have successfully identified key genomic regions and novel germplasm sources that offer reliable resistance to STB. These results emphasize the intricate, polygenic foundation of resistance and stress the significance of considering Genotype-by-Environment interactions [26, 27]

Ahmet [61] observed a range of responses among wheat genotypes, varying from resistant to vulnerable to STB. In the same way, Odilbekov *et al*. [62] noted that immune reactions, highly resistant responses, and very susceptible reactions were not detected. Additionally, Omrani *et al*. [63] evaluated 53 elite wheat lines against STB at the adult plant stage under natural infection circumstances during the 2020 and 2021 growing seasons, discovering that the materials showed moderately resistant, moderately susceptible, and susceptible responses. Their research also demonstrated strong virulence in the resistance genes Stb3, Stb4, Stb7, Stb8, and Stb9, whereas avirulence was observed in Stb11 and Stb18 during both years. Furthermore, Muche [64] pointed out considerable differences in reactions to STB among commercially available bread wheat varieties and advanced lines, spanning from resistant to susceptible. This observation is supported by results from Teklay *et al*. [28], which recorded varied reactions among bread wheat varieties to STB diseases in Tigray, Ethiopia

All genotypes were categorized based on severity and AUDPC as variables, leading to the creation of a dendrogram. The UPGMA analysis indicated that these genotypes were organized into eight distinct clusters. It is important to mention that resistant and susceptible genotypes were grouped into different clusters. Cluster IV, V and VI included genotypes that exhibited resistance to STB, while cluster one comprised the most susceptible genotypes. Among the 45 genotypes evaluated over a two-year period, the cultivar “Catbird” displayed the highest level of susceptibility, with a leaf blotch severity score of 70.44% and a pycnidia coverage score of 4.5. Conversely, the cultivar “ETW17-115” demonstrated the highest resistance, recording a leaf blotch severity score of 29.52% and a pycnidia coverage score of 1.3. Four separate groups were identified by the evaluation of infection types: sensitive, moderately susceptible, resistant, and moderately resistant. The genotypes classified within the superior cluster may prove to be highly beneficial for the development of STB-tolerant or resistant varieties, as well as for other breeding initiatives.

The distribution of these genotypes across the clusters revealed significant genetic variability concerning the resistance traits to STB that were examined. The relationships among the members of these clusters indicated that clusters I, and II, exhibited the highest mean severity and AUDPC values, whereas cluster IV, V, and VII demonstrated the lowest. The findings confirmed that all genotypes within cluster IV, under the trial conditions, displayed the least mean values for STB severity and AUDPC. Following evaluations across different annual conditions, all three genotypes in cluster IV have potential for use as sources of STB resistance. Additionally, these exceptional genotypes could be incorporated into hybridization programs aimed at developing varieties that are both resistant to STB and high-yielding.

Characteristics of Cluster Based on Mean, Standard Deviation and Standards Error of Mean reviled that, the mean (or cluster centroid) is the most important and represents the center of the cluster within the feature space [65]. By comparing the means of a variable across different clusters, we can better describe and label the clusters. Cluster I has high mean values for severity, AUDPC, and Pycnidia, indicating it contains the most susceptible spring wheat genotypes. Conversely, Cluster IV has the lowest means for these variables, suggesting that the spring wheat in this group is highly resistant.

The standard deviation (which reflects the cluster’s spread) measures how much the data points within a cluster vary for a particular variable [66]. It shows how homogeneous or tightly grouped the cluster is. A low standard deviation means the data points are close to the cluster mean, indicating high internal similarity. In contrast, a high standard deviation means the data points are more dispersed, implying the cluster is less compact and more internally diverse. The lowest standard deviation were found in Cluster VIII for severity (0.75), Cluster V for Pycnidia (0.096), and Cluster VII for AUDPC (28.05). whereas, the highest standard deviations are found in Cluster VIII for severity (1.86), Cluster VII for Pycnidia (1.295), and Cluster V for AUDPC (89.11). This suggests that spring wheat genotypes in Cluster VIII for severity, Cluster V for Pycnidia, and Cluster VII for AUDPC are tightly grouped with data points close to the cluster mean (high internal similarity). On the other hand, genotypes in Cluster VIII for severity, Cluster VII for Pycnidia, and Cluster V for AUDPC have the highest standard deviations, indicating their data points are widely spread, making these clusters less compact and more diverse internally. In descriptive clustering, standard error of the mean (SEM) mainly reflects how precisely the cluster mean is estimated. A smaller SEM indicates a more reliable estimate of the cluster mean. According to our result, most clusters have SEM values close to zero for severity and Pycnidia, showing that their means are estimated with high reliability.

The Correspondence Analysis (CA) bi-plot illustrates the relationships between the evaluated wheat genotypes and various traits. Dimension 1 account for 90.2% of the variation, while Dimension 2 explains 6.5%, together capturing 96.5% of the total variation. The correspondence analysis graph reveals three distinct trait groups: resistant genotypes, high-yield potential genotypes, and susceptible genotypes. The resistance genotype was found opposite to Severity, AUDPC and Pycnidia coverage. According to STB resistance genotype has low severity, AUDPC and Pycnidia coverage [19, 67] .

Principal component analysis revealed the presence of eight distinct components. The first two components explain the majority of the variation in the dataset. A score plot was created to evaluate the data's structure and to detect possible clusters, outliers, and trends. The positioning of data points on this plot may indicate the presence of two or more separate distributions within the dataset. Typically, coefficients can range from -1 to 1, with values nearing -1 or 1 indicating a substantial influence of the variable on the component. In contrast, coefficients close to 0 suggest a minimal effect of the variable on the component. A comprehensive analysis of the coefficients can provide additional insight into the connection of each component with the relevant variables. The analysis showed that components 1 and 2 combined explain about 61% of the variation. Specifically, the eigenvalues for PC1 and PC2, both exceeding 1, correspond to individual variance contributions of 42% and 19%, respectively, in relation to the cumulative variation of STB severity. The first two principal components were plotted along the PC axes 1 and 2, demonstrating significant variability among the existing spring wheat genotype and controls. Severity values exhibiting the highest absolute magnitudes approaching unity within PC1 exert a more significant impact on clustering compared to those with lesser absolute values that are closer to zero [68]. Consequently, in this study, the differentiation of advanced genotype into distinct clusters was attributed to the cumulative effects of severity and the Area under the Disease Progress Curve (AUDPC).

**Conclusion**

This study highlights the significant variations in resistance to STB among 45 bread wheat genotypes evaluated in Ethiopia, identifying remarkable cultivars such as Blouk #1, 6B662, Coulter, Erik, Gondo, and ETW17-115, which demonstrates robust resistance, and Catbird, which presents high susceptibility. The research indicated that adding differential lines to the wheat screening program for STB resistance assists in pinpointing the exact pathotypes that the lines resist, facilitating gene postulation. In this study, we identified genotypes (Blouk #1, 6B662, Coulter, Erik, Gondo, and ETW17-115) that show resistance to the pathotypes impacting the primary *Stb* genes present in Veranopolis (Stb2 and Stb6), Shafir (*Stb6*) and Estanzuela Federal (Stb7). Their research also demonstrated strong virulence in the resistance genes *Stb2, Stb6* and *Stb7*, whereas, avirulence where observed on *Stb3, Stb13* and *Stb14*, and *Stb16*. These findings underscore the importance of incorporating resistant genotypes into breeding initiatives to enhance wheat yields and mitigate the impact of this detrimental disease

**CONFLICT OF INTEREST STATEMENT: The authors declare that they have no conflict of interest.**

**ACKNOWLEDGEMENTS**

For providing the experimental material, and research facility, we thank Holetta agricultural research center Breeding and Pathology program. Many thanks to plant pathology research stuff for unreserved support during the research period. We thank Ethiopian Institute of Agricultural research for the fund.

**DATA AVAILABILITY STATEMENT:** The data uploaded as minimal data set of the study used in statistical analysis.

**Reference**

1. CSA (Central Statistical Authority). Agricultural Sample Survey 2020/21 (2013 E.C). Report on Livestock and Livestock Characteristics. Volume II, Statistical Bulletin 589, Central Statistical Authority, 2021; Addis Ababa.
2. USDA. World agricultural production. Circular series. WAP 11–22. 2023. <https://apps.fas.usda.gov/PSDOnline/Circulars/2023/08/production.pdf>.
3. USDA. World agricultural production. Circular series. Report Number: ET2025-0011. [Ethiopia: Grain and Feed Annual | USDA Foreign Agricultural Service](https://www.fas.usda.gov/data/ethiopia-grain-and-feed-annual-8)
4. Tesfaye Z, Girma T, Tanner D, Verkuijl H, Aklilu A and Mwangi W. Adoption of Improved Bread Wheat Varieties and Inorganic Fertilizer by Small Scale Farmers in Yelma Dansa and Farta Districts of Northwestern Ethiopia. Mexico, D.F. 2001; Ethiopian Agricultural Research Organization (EARO) and International Maize and Wheat Improvement Center (CIMMYT).
5. Mengistu H, Getaneh W, Yeshi A, Rbka D, Ayele B. Wheat Pathology Research in Ethiopia. In: Hailu,G.,Tanner,D.G.,Mengistu,H.(Eds.),Wheat Research in Ethiopia: a Historical Perspective. IAR/CIMMYT, Addis Ababa, 1991; pp.173-217.
6. Endale H and Getaneh W. Survey of Rust and Septoria Leaf Blotch Diseases of Wheat in Central Ethiopia and Virulence Diversity of Stem Rust *Puccinia graminis*f. sp.*tritici*.” *Advances in Crop Science and Technology,* 2015; 3(2), 1-5.
7. Yitagesu T, Alemayehu C, Bekele K. Survey of *Septoria tritici* blotch of bread wheat (*Triticum aestivum* L.) in the central highlands of Ethiopia. *American Journal of Bioscience and Bioengineering.* 2018; 6(5): 36-41
8. Meyer M, Bacha N, Tesfaye T, Alemayehu Y, Abera E, Hundie B. Wheat rust epidemics damage Ethiopian wheat production: A decade of field disease surveillance reveals national-scale trends in past outbreaks. *PLoS ONE*. 2021; 16(2): e0245697. https://doi.org/10.1371/journal.pone.0245697
9. Alemar S and Temam H. Epidemics of *Septoria tritici* Blotch and its Development over Time on Bread Wheat in Haddiya Kambata Area of Southern Ethiopia. *Journal of Biology, Agriculture and Healthcare,* 2016; 6(1), 47-57.
10. Bockus WW, Bowden RL, Hunger RM, Morrill WL, Murray TD, and Smiley RW. Compendium of Wheat Diseases and Pests: Third Edition. *American Phytopathological Society*, 2010; St. Paul, MN
11. Heick TM, Justesen AF, Jorgensen LN. Resistance of wheat pathogen Zymoseptoria tritici to DMI and QoI fungicides in the Nordic-Baltic region - a status. *Eur J Plant Pathol*. 2017;149(3):669–82.
12. Cools HJ, Fraaije BA. Update on mechanisms of azole resistance in *Mycosphaerella graminicola* and implications for future control. *Pest Manag Sci*. 2013;69(2):150–5.
13. Yang N, Ovenden B, Baxter B, McDonald MC, Solomon PS and Milgate A. Multi-stage resistance to *Zymoseptoria tritici* revealed by GWAS in an Australian bread wheat diversity panel. *Front. Plant Sci*. 2022; 13:990915. doi: 10.3389/fpls.2022.990915
14. Suffert F, Le Prieur S, Gelisse S, Dzialo E, Saintenac C, Marcel TC. Estimating the frequency of Virulence against an *Stb* gene in *Zymoseptoria tritici* populations by bulk phenotyping on checkerboard microcanopies of wheat NILs. *plant pathology*. 2024;73(6):1573-1585, <https://doi.org/10.1111/ppa.13894>
15. Brown JKM, Chartrain L, Lasserre-Zuber P, and Saintenac C. Genetics of Resistance to *Zymoseptoria tritici* and Applications to Wheat Breeding. *Fungal Genetics and Biology,* 2015; 79(1), 33-41. <https://doi.org/10.1016/j.fgb.2015.04.017>
16. Dreisigacker S, Wang X, Martinez Cisneros BA, Jing R, Singh PK. Adult-plant Resistance to *Septoria tritici* Blotch in Hexaploid Spring Wheat. *Theor Appl Genet,* 2015;128:2317–2329. <https://doi.org/10.1007/s00122-015-2587-9>
17. [Eriksen](https://pubmed.ncbi.nlm.nih.gov/?term=Eriksen+L&cauthor_id=12783168)  L, [Borum](https://pubmed.ncbi.nlm.nih.gov/?term=Borum+F&cauthor_id=12783168) F, [Jahoor](https://pubmed.ncbi.nlm.nih.gov/?term=Jahoor+A&cauthor_id=12783168) A. Inheritance and localization of resistance to *Mycosphaerella graminicola* causing *Septoria tritici* blotch and Plant Height in the Wheat (*Triticum aestivum* L.) Genome with DNA Markers, *Theor Appl Genet*. 2003;107(3):515-27. doi: 10.1007/s00122-003-1276-2.
18. Chartrain L, Brading PA, Makepeace JC, Brown JKM. Sources of Resistance to *Septoria tritici* Blotch and Implications for Wheat Breeding. *PlantPathol*. 2004; 53(4),454–460. <https://doi.org/10.1111/j.1365-3059.2004.01052.x>
19. Simon MR, Ayala FM, Corda CA, Order MS, Boerner A. Molecular Mapping of Quantitative Trait Loci Determining Resistance to *Septoria tritici* Blotch Caused by *Mycosphaerella graminicola*. *Euphytica*. 2004; 138:41-48.
20. Tabib Ghaffary SM, Faris JD, Friesen TL, Visser RG, van der Lee TA, Robert O, Kema GH. New broad-spectrum resistance to septoria tritici blotch derived from synthetic hexaploid wheat. *Theor Appl Genet*. 2012;124(1):125-42. doi: 10.1007/s00122-011-1692-7. Epub 2011 Sep 13. PMID: 21912855; PMCID: PMC3249545.
21. Langlands-Perry C, Pitarch A, Lapalu N, Cuenin M, Bergez C, Noly A, et al. Quantitative and qualitative plant-pathogen interactions call upon similar pathogenicity genes with a spectrum of effects. *Front Plant Sci*. 2023;14:1128546. doi: 10.3389/fpls.2023.1128546.
22. Kema GHJ, Verstappen ECP, Todorova M, Waalwijk C. Successful Crosses and Molecular Tetrad and Progeny Analyses Demonstrate Heterothallism in *Mycosphaerella graminicola. Curr Genet.* 1996; 30: 251-258.
23. Eyal Z, Amiri Z, and Wahl I. Physiologic Specialization of *Septaria tritici.* *Phytopathology.* 1973; 63(1):1087
24. Eyal Z, Scharen A L, Huffman M D, and Prescott J M. Global Insights in to Virulence Frequencies of *Mycosphaerella graminicola. Phytopathology.* 1985; 75:1456-1462.
25. Tilahun M, Teklehaimanot H, Bekele G A, and Kassahun T. Virulence Variability of Ethiopian *Zymoseptoria tritici* Isolates and Efficacy of Wheat Genotypes and *Stb* Resistance Genes Against the Isolates. *Eur J Plant Pathol.,* 2020; 158(4):895-910 <https://doi.org/10.1007/s10658-020-02125-3>
26. Kassie MM, Abebe TD, Desta EA, Tadesse W. Genome-wide association analysis of *Septoria tritici* blotch for adult plant resistance in elite bread wheat (*Triticum aestivum* L) genotypes. *PLoS ONE*. 2025; 20(3): e0317603. https://doi.org/10.1371/journal.pone.0317603
27. Yosef G., Kidane B N, Hailemariam DK, Mengistu, CF, Mario E P, Matteo D. Genome-Wide Association Study of *Septoria tritici* Blotch Resistance in Ethiopian Durum Wheat Landraces, *Front. Plant Sci*. 2017; (8) | https://doi.org/10.3389/fpls.2017.01586
28. Teklay AT and Zeray S G. Occurrence and Intensity of Wheat *Septoria tritici* Blotch and Host Response in Tigray, Ethiopia. *Crop Protection.* 2015; 68: 67-71
29. Yohannes A, Birhanu F and Yeshambel A. Field Response and Identification of Bread Wheat Genotypes to *Septoria tritici* Blotch in the Highlands of North Gondar. *Journal of Current Research in Food Science*, 2020; *1*(2), 09-17.
30. Bekle K and Yitagesu K . Phenotyping of Bread and Durum Wheat Accessions, lines and Varieties for *Septoria* leaf blotch (SLB) and *Fusarium* head blight at Holetta , Ethiopia. Eshetu Derso, Bedada Girma (eds) in *Proceedings of the Delivering Genetic Gain in Wheat (DGGW) Project Closing Workshop*. Addis Ababa, Ethiopia, 2020; pp.120.
31. Tamene L, Amede T, Kihara J, Tibebe D, Schulz S. (eds.). A review of soil fertility management and crop response to fertilizer application in Ethiopia: towards development of site- and context-specific fertilizer recommendation. CIAT Publication No. 443. International Center for Tropical Agriculture (CIAT), Addis Ababa, Ethiopia. 2017; 86 p. Available at:http://hdl.handle.net/10568/82996
32. McIntosh R A, Devos K M., and Dubcovsky J V. Catalogue of gene symbols for wheat supplement retrieved from http://wheatpwusdagov/ggpages/wgc/upd html.2007.
33. Singh P K, Singh RP, Duveiller E, Mergoum M, Adhikari TB, Elias EM. Genetics of wheat–*Pyrenophora tritici-repentis* interactions, *Euphytica*. 2010; 171:1–13 DOI 10.1007/s10681-009-0074-6
34. Pawan Singh , Scientist and Head of the Wheat Pathology, Global Wheat Program at International Maize and Wheat Improvement Center (CIMMYT), Mexico
35. Wilson RE. Inheritance of resistance to *Septoria tritici* in wheat. In: Scharen AL (ed) Septoria of Cereals; Proceedings of the Workshop. Bozeman, Montana State University. 1985. pp 33–35.
36. Brown JKM, Kema GHJ, Forrer HR, Verstappen ECP, Arraiano LS, Brading PA, Foster EM, Fried PM, Jenny E. Resistance of wheat cultivars and breeding lines to *Septoria tritici* blotch caused by isolates of *Mycosphaerella graminicola* in field trials. *Plant Pathol.* 2001; 50(1):325–338.
37. Adhikari TB, Yang X, Cavaletto JR, Hu X, Buechley G, Ohm HW, Shaner G. Molecular mapping of *Stb1*, a potentially durable gene for resistance to *Septoria tritici* blotch in wheat. *Theor Appl Genet*. 2004; 109(1):944–953.
38. Chartrain L, Joaquim P, Berry ST, Arraiano LS, Azanza F, Brown JKM. Genetics of resistance to *Septoria tritici* blotch in the Portuguese wheat breeding line TE 9111. *Theor Appl Genet*. 2005; 110(1):1138–1144.
39. Kema GHJ, Verstappen ECP, Waalwijk C. Avirulence in the wheat *Septoria tritici* leaf blotch fungus *Mycosphaerella graminicola* is controlled by a single locus. *Mol Plant Microbe Interac*t. 2000; 13:1375–1379.
40. McCartney CA, Bruˆ le´-Babel AL, Lamari L, Somers DJ. Chromosomal location of a race-specific resistance gene to *Mycosphaerella graminicola* in the spring wheat ST6. *Theor Appl Genet*.2003; 107:1181–1186.
41. Dalvand M, Zafar D, Pari S M J, Roohparvar R, & Ghafari S M T. Studying genetic diversity in *Zymoseptoria tritici*, causal agent of *Septoria Tritici* blotch, by using ISSR and SSR markers. *Journal of Agricultural Science and Technology*. 2018; 20(6), 1307–1316.
42. Dubin HI and Rajaram S. Breeding disease-resistance wheat for tropical highlands and lowlands. *Annu. Rev. Phytopathol*. 1996; 34:503-526.
43. Eyal Z. The *Septoria tritici* and *Stagonospora nodorum* blotch diseases of wheat. *Eur J Plant Pathol*. 1999; 105:629–641.
44. Tufa T, Haji K, and Tolera A. Influence of Seed Rate and Row Spacing on Growth and Yield of Bread Wheat in Chelia District of West Showa Zone, Ethiopia, *Journal of Natural Sciences Research*. 2019;9(5): ISSN 2224-3186 (Paper) ISSN 2225-0921 (Online), DOI: 10.7176/JNSR
45. Zadocks J C, Chang T T, and Konzak C F A. Decimal Code for the Growth Stages of Cereals. *Weeds Res*, 1974; 14:415-421
46. Saari EE and JM Prescott.  A Scale for Appraising the Foliar Intensity of Wheat Diseases. *Plant Disease Reporter.* 1975; 59 (5): 377–380.
47. Eyal Z, and Levy E. Variations in Pathogenicity Patterns of *Mycosphaerella graminicola* within *Triticum* spp in Israel.” *Euphytica*. 1987; 36: 237–250. <https://doi.org/10.1007/BF00730670>
48. Madden LV, Hughes G, Van den Bosch F.  The Study of Plant Disease Epidemics. 2007; St. Paul, MN, USA: APS Press.
49. Eyal Z, and Brown MB. A quantitative method for estimating density of *Septaria tritici* pycnidia on wheat leaves. *Phytopathology*. 1976; 66:11-14.
50. Adhikari T B, Anderson JM, and Goodwin SB,. Identification and Molecular Mapping of a Gene in Wheat Conferring Resistance to *Mycosphaerella graminicola*. *Phytopathology,* 2003; 93(9), 1158-1164. doi: 10.1094/PHYTO.2003.93.9.1158.
51. SAS. 2008. Statistical Analysis Systems Institute*. Version 9.1*, SAS Institute Inc., Cary, North Carolina, USA.
52. Gomez KA, and Gomez AA. Statistical Procedures for Agricultural Research. 2^nd^ Edition, John Wiley and Sons, New York. 1984. 680 p.
53. Sokal, R. R., and Rohlf, J. F. Biometry *(3^rd^ ed.)*. 1995; New York: Freeman.
54. Riaz A, Kockappelgren P, Hehir J G, Kang J, Meade F, Cockram J, *et al*.  Genetic analysis using a multi-parent wheat population identifies novel sources of Septoria tritici resistance.  *Genes*. 2020; 11(8), 887. blotch  <https://doi.org/10.3390/genes11080887>
55. Prahl KC, Klink H, Hasler M, Verreet J-A, Birr T. Will Climate Change Affect the Disease Progression of *Septoria Tritici* Blotch in Northern Europe? Agronomy. 2023; 13(4):1005. https://doi.org/10.3390/agronomy13041005
56. Chungu C, Gilbert J, and Townley-Smith F. *Septoria tritici* blotch development as affected by temperature, duration of leaf wetness, inoculum concentration, and host. *Plant Dis*. 2001;85(1):430-435.
57. Tiley Anna MM, Karki Sujit J, and Feechan Angela. Zymoseptoria tritici. In: eLS. John Wiley & Sons, Ltd, Chichester. 2018. DOI: 10.1002/9780470015902.a0027948
58. Porras R, Miguel-Rojas C, Lorite IJ, Pérez-de-Luque A, Sillero JC. Characterization of Durum Wheat Resistance against *Septoria Tritici* Blotch under Climate Change Conditions of Increasing Temperature and CO_2_ Concentration. *Agronomy*. 2023; 13(10):2638. <https://doi.org/10.3390/agronomy13102638>
59. Suffert F, Ravigné V, Sache I. Seasonal changes drive short-term selection for fitness traits in the wheat pathogen *Zymoseptoria tritici*. *Appl Environ Microbiol*. 2015; 81:6367–6379. doi:10.1128/AEM.00529-15.
60. Tabib Ghaffary M S, van der Lee TAJ, Verstappen ECP, and Kema GH J.  Screening for resistance to septoria tritici blotch, the major wheat disease in Western Europe. Poster session presented at 7th International *Mycosphaerella* and *Stagonospora* Symposium, Ascona, Switzerland. 2008.  <https://edepot.wur.nl/189351>
61. Ahmet ÇAT. Evaluation of Some Bread Wheat (*Triticum aestivum* L.) Varieties Reaction to *Septoria Tritici* Blotch Disease, *Turk J Agric Res.* 2023; 11(1): 18-25. doi: 10.19159/tutad.1373924
62. Odilbekov F, Armoniené R, Koc A, Svensson J and Chawade A. GWAS-Assisted Genomic Prediction to Predict Resistance to *Septoria Tritici* Blotch in Nordic Winter Wheat at Seedling Stage. *Front. Genet*. 2019; 10:1224. doi: 10.3389/fgene.2019.01224
63. Omrani A, Roohparvar R, Shahbazi K. Reaction of Promising Wheat Lines to Septoria Leaf Blotch Disease at the Seedling and Adult Plant Stages. *Applied Entomology and Phytopathology*. 2023; 90(2): 195-208.
64. Muche A G. Assessment of Ethiopian Advanced Bread Wheat Genotypes and Varieties to *Septoria tritici* leaf blotch, *RJOAS*. 2022; 6(126), 181, UDC 633; DOI 10.18551/rjoas.2022-06.21
65. Amelio A, Tagarelli A. [Encyclopedia of Bioinformatics and Computational Biology](https://www.sciencedirect.com/referencework/9780128114322/encyclopedia-of-bioinformatics-and-computational-biology) , 2018 pp 437-448
66. Vasileios C. Pezoulas, Themis P. Exarchos, Dimitrios I. Fotiadis [Medical Data Sharing, Harmonization and Analytics](https://www.sciencedirect.com/book/9780128165072/medical-data-sharing-harmonization-and-analytics) , 2020 pp 227-309
67. Lakachew B, Hassen S, Wuletaw T. Association mapping of *Septoria tritici* blotch resistance in bread wheat in Bale and Arsi highlands, Ethiopia, Heliyon. 2024; 10(11): 2405-8440, e32265,ISSN, <https://doi.org/10.1016/j.heliyon.2024.e32265>.
68. [Chahal](https://www.google.com.et/search?tbo=p&tbm=bks&q=inauthor:%22G.+S.+Chahal%22) G S, [Gosal](https://www.google.com.et/search?tbo=p&tbm=bks&q=inauthor:%22S.+S.+Gosal%22) S S. Principles and Procedures of Plant Breeding: Biotechnological and Conventional Approaches, CRC Press, 2002, Alpha Science International Ltd. Harrow, U.K.
