# Supplementary material for: Field evaluation of spring wheat genotypes reveals differential resistance to Zymoseptoria tritici in Ethiopia
Source: PLoS One. 2026 Jul 10;21(7):e0353375. doi: 10.1371/journal.pone.0353375 (PMC13353992; doi:10.1371/journal.pone.0353375)
Supplement: Attachment — Submitted filename: Response to Reviewers.docx [file pone.0353375.s011.docx]

**Response to Reviewers**

Dear Reviewer,

We sincerely thank you for your careful evaluation of our manuscript and for the constructive comments and suggestions provided. We have carefully revised the manuscript to address all concerns raised. Our detailed responses are presented below.

**1. Definition of differential lines**

We have clarified in the revised manuscript that “differential lines” refer to wheat genotypes carrying known major resistance genes (*Stb* genes) to Septoria tritici blotch (STB). These lines are commonly used to identify pathogen virulence patterns and to postulate the presence of specific resistance genes based on their reaction profiles.

**2. Clarification of N and associated symptoms**

We have specified whether “N” (necrosis) includes pycnidia coverage (PC). In the revised version, necrosis (N) refers strictly to chlorotic and pycnidia bearing necrotic leaf area, while pycnidia density (PC) represents the proportion of pycnidia density within necrotic tissue. The symptoms associated with N—chlorotic lesions progressing to necrotic blotches—are now clearly described.

**3. Disease scale discrepancies (Eyal vs. Adhikari scales)**

We acknowledge the inconsistency in the description of disease scales. The revised manuscript now clearly states which scale was adopted as follows. Infected plants were evaluated using two disease assessment methods. (i) Disease severity (DS) was estimated visually as the percentage of leaf area covered by necrotic lesions containing pycnidia. This assessment employed a modified version of the double-digit (00–99) scoring system developed by Saari and Prescott, which is based on a severity scale for evaluating foliar diseases in wheat. (ii) The level of sporulation was estimated as pycnidial density within necrotic lesions using a 0–5 scale based on Adhikari and Louriki, A detailed description of the scoring criteria has been added in materials and method section.

**4. Description of the scale used**

We have clarified that the scoring scale corresponds to the average percentage of leaf area covered by pycnidia-bearing necrosis per leaf, assessed visually. It does not refer solely to pycnidia density within lesions but to the overall proportion of infected leaf tissue per assessed leaf.

**5. Meaning of numbers in parentheses**

The numbers previously shown in parentheses represented the range of percentage score of pycnidia density within necrotic lesion. This has now been explicitly explained in the table in materials and method section to avoid ambiguity.

**6. Data transformation and computation of means**

We have revised the statistical description to clarify that when categorical disease classes corresponded to percentage ranges, midpoint values were used for quantitative analysis (e.g., calculation of means across plants and replicates). The methodology for back-transformation and reclassification into resistance levels is now clearly described.

**7. Qualitative vs. quantitative analysis**

We have clarified the classification of variables as either quantitative (e.g., percentage severity, AUDPC) or qualitative (e.g., resistance categories). Where applicable, categorical analyses—such as the proportion of genotypes exhibiting low, medium, and high severity—are now explicitly described.

**8. Variables and criteria used in cluster analysis**

The revised manuscript now specifies the variables included in the clustering procedure (e.g., mean severity, AUDPC, pycnidia density), the distance metric applied (Euclidean distance), and the clustering method used (hierarchical clustering with Ward’s method). These details have been added to the Materials and Methods section.

**9. Addition of bar chart**

A new figure (bar chart) has been included presenting all genotypes with at least one assessment of disease severity and corresponding AUDPC values, as requested.

**10. Computation of infection types**

We have clarified how infection types were derived. Infection types were computed based on the mean percentage of pycnidia-density within a lesion across six sequential disease assessments and subsequently categorized according to predefined resistance thresholds. This procedure is now fully described in Materials and Methods.

**11. Definition of severity**

We have explicitly defined “severity” as the percentage of leaf area covered by necrotic lesions bearing pycnidia, assessed visually at each scoring date.

**12. Graph readability**

For improved clarity, genotypes name shorted, and labeled selectively to enhance legibility, as suggested.

**13. Correspondence analysis categories**

We have clarified that correspondence analysis was conducted using categorical variables. Continuous variables such as AUDPC and severity were first discretized into quartile-based categories (low, medium and high). The definition of each category is now clearly described in materials and method section.

**14. Revision of the Discussion section**

The Discussion has been substantially shortened and revised. Repetitions from the Introduction, Materials and Methods, and Results sections have been removed. The revised Discussion now focuses on synthesizing key findings, interpreting their biological relevance, and highlighting implications for resistance breeding and STB management.

We greatly appreciate the reviewer’s insightful comments, which have significantly improved the clarity, rigor, and overall quality of our manuscript.

Sincerely,
Habtewold Kifelew
