# Supplementary material for: Field evaluation of spring wheat genotypes reveals differential resistance to Zymoseptoria tritici in Ethiopia
Source: PLoS One. 2026 Jul 10;21(7):e0353375. doi: 10.1371/journal.pone.0353375 (PMC13353992; doi:10.1371/journal.pone.0353375)
Supplement: Attachment — Submitted filename: Response_to_Reviewers_auresp_3.docx [file pone.0353375.s015.docx]

**Response to Reviewers**

**Manuscript ID:** PONE-D-25-41622R2
**Title:** Field Evaluation of Spring Wheat (*Triticum aestivum* L.) Genotypes Reveals Differential Resistance to *Zymoseptoria tritici* at Holetta, Ethiopia

Dear Dr. Willocquet,

We sincerely thank you for your careful evaluation of our revised manuscript and for the constructive comments provided. We appreciate the opportunity to further improve our work. We have carefully addressed each of the issues raised, and the revisions have been incorporated into the manuscript accordingly. Below we provide a point-by-point response to your comments.

**Comment 1: Clarification of disease severity definition**

*“It is not clear in the text if ‘disease severity’ refers to the average over the 6 assessments, or to a specific assessment. This needs to be specified in Materials and Methods as well as in all parts of the Results…”*

**Response:**
Thank you for this important clarification. In the revised manuscript, we have explicitly defined disease severity in the Materials and Methods section. Disease severity now refers to the mean of six successive field assessments conducted at regular intervals during the epidemic period.

We have also revised all relevant sections in the Results to consistently indicate that severity values represent the average across the six assessments, and this clarification is now consistently applied throughout the manuscript (including figure captions and table notes where applicable).

**Comment 2: Organization of the Results section and reduction of tables/figures**

*“The results section needs to be reorganized, and the number of tables and figures need to be reduced…”*

**Response:**
We appreciate this suggestion and have substantially reorganized the Results section to improve clarity, logical flow, and conciseness. Specifically:

- We reviewed all tables and figures and **removed redundant or overlapping visuals**.
- Remaining figures and tables were reorganized to follow a clearer logical sequence:
  1. ANOVA results are presented first.
  2. Disease severity and AUDPC results are presented next using **separate, streamlined figures**, each structured by year.
  3. Pycnidial density results are presented separately, with genotypes grouped according to resistance classes.
  4. Multivariate analysis results (e.g., PCA/cluster analysis) are presented last.

Additionally, descriptive text in the Results section has been revised to be more concise, focusing strictly on reporting findings, while interpretative statements and implications have been moved to the Discussion section in accordance with your recommendation.

**Comment 3: Discrepancy in genotype classification criteria**

*“On the one hand, classification of R, MR, S is based on pycnidial density. On the other hand, severity or AUDPC are also used…”*

**Response:**
We agree that the previous version lacked clarity regarding classification criteria. In the revised manuscript, we have:

- Clearly stated that pycnidial density is the primary criterion used for categorizing genotypes into resistant (R), moderately resistant (MR), and susceptible (S) groups.
- Clarified that AUDPC and disease severity are used as complementary quantitative indicators to describe epidemic intensity, but not as primary classification criteria.
- Removed any ambiguity by ensuring that genotype classification is consistently based on pycnidial density, while severity and AUDPC are presented as supporting epidemiological measures.

We have also revised the relevant sections in both the Results and Methods to explicitly distinguish between:

- Classification criteria (pycnidial density), and
- Analytical variables (severity and AUDPC).

**Comment 4: Clarification of the purpose of classification and analysis**

*“Is the purpose of the work to classify genotypes according to a pre-set criterion, or to assess and identify the best criterion of classification?”*

**Response:**
Thank you for raising this important conceptual point. We have clarified the objective of the study in the Introduction and reinforced it in the Methods and Discussion sections.

The primary objective of the study is to:

- Evaluate and characterize the resistance of spring wheat genotypes to *Zymoseptoria tritici* under field conditions, using a pre-defined classification criterion (pycnidial density) commonly used in plant pathology.

The study does not aim to identify a new classification criterion, but rather to:

- Assess genotype performance using established epidemiological and pathological parameters (severity, AUDPC, and pycnidial density), and
- Use these complementary metrics to provide a comprehensive assessment of resistance.

This clarification has been explicitly stated in the revised manuscript to avoid ambiguity.

**Comment 5: Clarification of variables used in cluster analysis**

*“The variables involved in the cluster analysis are still not described clearly…”*

**Response:**
We appreciate this observation and have now clarified the cluster analysis methodology in the revised manuscript.

Specifically:

- The variables included in the cluster analysis are now explicitly listed in the Methods section.
- The analysis includes **disease-related variables only**, namely:
  - Disease severity (averaged over the six assessments),
  - AUDPC,
  - Pycnidial density.

Agronomic traits were **not included** in the cluster analysis; this has been clearly stated.

We have also clarified that:

- For disease severity and pycnidial density, **summary statistics (mean over the six assessments)** were used rather than individual time-point measurements.

These clarifications have been incorporated into the Methods section to ensure transparency and reproducibility.

We thank the Academic Editor for the insightful and constructive comments, which have significantly improved the clarity, structure, and scientific rigor of the manuscript. We believe that the revised version now addresses all concerns and meets the expectations for publication in *PLOS ONE*.

Sincerely,
Habtewold Kifelew / Corresponding Author
