# Supplementary material for: Field evaluation of spring wheat genotypes reveals differential resistance to Zymoseptoria tritici in Ethiopia
Source: PLoS One. 2026 Jul 10;21(7):e0353375. doi: 10.1371/journal.pone.0353375 (PMC13353992; doi:10.1371/journal.pone.0353375)
Supplement: Attachment — Submitted filename: PONE-D-25-41622_R3 - AE.docx [file pone.0353375.s012.docx]

**Field Evaluation of Spring Wheat Genotypes Reveals Differential Resistance to Zymoseptoria tritici in Ethiopia**

Habtewold Kifelew^2^, Habtamu Terefe^2^, Bekele Kasa ^1^, Tilahun Mekonen ^3^, Zelalem Bekeko ^2^ and Bulti Tesso ^2^

^1^ Holetta Agricultural Research Center, P.O.Box 31/2003, Holeta, Ethiopia

^2^ Haramaya University, College of Agriculture and Environmental Sciences, P.O. Box 138, Dire Dawa, Ethiopia,

^3^ Addis Ababa University, Institute of Biotechnology, P.O. Box 1176, Addis Ababa, Ethiopia

Corresponding author: Habtewold Kifelew, ([kifelew@yahoo.com](mailto:kifelew@yahoo.com)) Telephone: +251913312650

**Abstract**

*Septoria tritici* blotch (STB), caused by *Zymoseptoria tritici*, is a major disease of spring wheat in Ethiopia and worldwide. This study evaluated 45 spring wheat genotypes for adult plant resistance under natural infection at Holetta Agricultural Research Center during the 2022 and 2023 seasons. Disease was assessed using two methods: (i) visual estimation of disease severity (DS) as the percentage of leaf area with necrotic lesions bearing pycnidia; and (ii) pycnidial density within lesions scored on a 0–5 scale to classify resistance levels. Combined analysis of variance across years showed significant effects (P ≤ 0.01) of year, genotype, and genotype × year interaction. Genotype accounted for 42.81% of the total variance in mean disease severity (from early grain filling to 70% flag leaf infection with pycnidia-bearing necrosis) and 42.3% of the variance in AUDPC. Mean severity ranged from 46.46% to 73.49% in 2022 and 8.73% to 71.71% in 2023, while pycnidial density ranged from 2.23% to 35.7% and 1.21% to 45.47% in 2022 and 2023, respectively. Four reaction classes were identified: resistant (24.4% and 25.2%), moderately resistant (30.4% and 24.4%), moderately susceptible (22.2% and 20.7%), and susceptible (22.96% and 29.63%) in 2022 and 2023, respectively. ‘Catbird’ was the most susceptible cultivar, whereas ‘Gondo’ was the most resistant. Cluster analysis grouped the cultivars into eight clusters based on STB severity and AUDPC, including two clusters (six genotypes) with resistant responses. These clusters did not include the differential lines used to characterize Z. tritici races, suggesting that the underlying resistance genes require further molecular characterization. The resistant genotypes (Blouk #1, 6B662, Coulter, Erik, Gondo, and ETW17-115) were effective against pathotypes virulent to major *Stb* genes present in Veranopolis (*Stb2*, *Stb6*), Shafir (*Stb6*), and Estanzuela Federal (*Stb7*). Disease severity and AUDPC were negatively correlated with plant height, number of tillers per plant, spike length, number of seeds per spike, thousand kernel weights, and grain yield. Correspondence and principal component analyses identified three major groups among the 45 genotypes: resistant, high-yield potential, and susceptible. The resistant genotypes identified here provide valuable material for breeding programs targeting improved resistance to *Z. tritici*.

**Key word**: wheat, *Septoria tritici* blotch, *Zymoseptoria tritici*, host plant resistance, breeding

**Introduction**

Spring wheat is the primary cereal crop in Ethiopia, producing 4.93 million metric tons with an average productivity of 2.73 tons per hectare. It is cultivated by 4,212,518 smallholder farmers across 1.8 million hectares [1]. Recently, due to the expansion of irrigated wheat production initiatives, Ethiopia has become one of the fastest-growing wheat producers in Africa. Ethiopia wheat production is expected to reach 6.5 million metric tons by the year 2025/26 [2]. However, Ethiopia wheat productivity remains low compared to Egypt (6.4 t ha⁻¹) [3]. The low productivity is linked to various biotic and abiotic factors, as well as limited adoption of modern agricultural technologies [4]. In terms of diseases, stem and leaf rust are prevalent in low and mid-altitude regions, while stripe rust (yellow rust) and Septoria are more common in high-altitude wheat-growing areas of Ethiopia [5, 6, 7, 8]

Particularly in the highlands, *Zymoseptoria tritici,* the pathogen causing Septoria tritici blotch (STB) disease, is among the most devastating diseases of wheat in Ethiopia, ranking second only to stripe rust. This pathogen is responsible for yield losses that range from 41% [9] to 82% [5, 6]. In addition to lowering productivity, *Z. tritici* infection degrades grain quality [10]. Due to frequent STB outbreaks and a lack of effective disease management strategies, wheat production in the area has recently become challenging.

Evaluating spring wheat for resistance to STB is essential in worldwide wheat breeding because of the pathogen evolutionary ability and the rising concern of fungicide resistance [11, 12]. STB can lead to considerable yield reductions, making genetic resistance the most sustainable and eco-friendly control method. Screening initiatives mainly concentrate on detecting two types of resistance, qualitative and quantitative (associated with Quantitative Trait Loci, QTL). Qualitative resistance results from major Stb genes which confer strong, race-specific resistance, typically following a gene-for-gene interaction model. Though successful against non-virulent pathogen strains, they are susceptible to being outpaced by the pathogen swift development of virulence. To date, 24 resistance (R) genes against *Z. tritici* have been identified and documented [13]. Among them, 23 *Stb* genes have been genetically mapped in wheat [14]. These genes were found in wheat through field trials and studies on interactions between genotypes and isolates. Resistance to STB is primarily quantitative under field conditions [15, 16]. Quantitative resistance offers a less complete yet long-lasting defense, governed by numerous genes (QTLs) with smaller, cumulative impacts. Contemporary breeding increasingly seeks a combination, or pyramiding, of both significant *Stb* genes and QTLs to enhance protection that is more resilient and durable. Currently the wheat genome contains a large number of QTLs linked to resistance [17, 18, 19]. Since 2015, there have been findings indicating that 89 chromosomal regions harbor QTLs associated with resistance to STB [15]. Alternative origins, such as synthetic hexaploid wheat, are being explored to integrate newly effective resistance genes such as Stb16q [20]. A recent study using QTL analysis discovered that genes involved in both quantitative and qualitative STB-wheat interactions are similar. The study also demonstrated that genes underlying pathogenicity QTL can be analogous to *Avr* genes [21].

Studies in Ethiopia have revealed variability in resistance among bread wheat, durum wheat, and triticale [22]. Eyal and his team [23, 24] investigated the virulence traits of 97 *Z. tritici* isolates obtained from 22 countries, including Ethiopia, by evaluating them on 35 varieties of wheat and triticale. Their findings confirmed isolate-cultivar specificity, indicating diverse virulence genes in the pathogen population. Molecular analyses by Tilahun [25] further demonstrated substantial genetic variability in resistance among 180 Ethiopian bread wheat genotypes. Genome-Wide Association Studies (GWAS) conducted on Ethiopian wheat have effectively pinpointed important genomic areas and new germplasm sources that provide consistent resistance to STB. These findings highlight the complex, polygenic basis of resistance and underscore the importance of addressing genotype-by-environment interactions [26, 27]. Screening programs in Tigray [28], Gondar [29], and Holetta [30] have also contributed to identifying resistant genotypes.

The purpose of this study was to assess phenotypic variation in spring wheat germplasm to identify resistance to STB. To achieve this, 45 spring wheat genotypes, including 10 differential lines for STB, were assessed under natural infection over two consecutive years at an open field site in the central highlands of Ethiopia. Agronomic traits were also measured on the same genotypes, enabling an analysis of the relationship between resistance and agronomic characteristics in Ethiopian spring wheat.

**Materials and methods**

**Description of the study area**

The genotypes were evaluated at the Holetta Agricultural Research Center in Ethiopia, under field conditions during the 2022 and 2023 cropping seasons. The location is expected to represent the major and potential spring wheat production area in central highlands of Ethiopia, the location is also recognized as a hotspot for STB epidemics. The center is 2,400 meters above sea level and located at latitude 9°00' N and longitude 38°30' E. The site has minimum and maximum temperatures of 6°C and 22°C, respectively; it receives 1,144 mm of rainfall annually (Table 1). The soil type known as nitosols has a pH of 6.0 [31].

| Weather variables* | Year 2022 | | | | | Year 2023 | | | | |
| --- | --- | --- | --- | --- | --- | --- | --- | --- | --- | --- |
|  | June | July | August | September | Mean | June | July | August | September | Mean |
| T^O^ min. | 9 | 10.2 | 10.5 | 9.8 | 9.875 | 9.8 | 10.5 | 10.2 | 10 | 10.13 |
| T^O^ max. | 26.5 | 25.7 | 25.9 | 24.5 | 25.65 | 22.3 | 20.9 | 21.2 | 21.4 | 21.45 |
| Rainfall(mm) | 200.7 | 390.8 | 331.7 | 218.7 | 285.48 | 112.4 | 202.3 | 271.8 | 166.4 | 188.23 |
| RH (%) | 77 | 84 | 81 | 78 | 80 | 81 | 84 | 84 | 80 | 82.25 |

**Table.1. Weather condition of Holetta during spring wheat growing season 2022, 2023**

Source:-Holetta agricultural research center, weather station

*T^O^ min=minimum temperature, T^O^ max= maximum temperature, Rainfall=mean monthly average rainfall amount, RH=Relative humidity percentage

**Plant materials**

A total of 45 bread wheat genotypes were evaluated for their reaction to the *Z. tritici* field population at Holetta during the 2022 and 2023 cropping seasons. Most of these genotypes were recently introduced to the country, and their disease resistance status is not yet known. Differential lines—genotypes carrying known major resistance genes to STB were included so as to identify the specific races against which the genotype shows resistance, which can help in gene postulation. The susceptible variety “Paven- 76,” the moderately susceptible variety “Kingbird,” the moderately resistant variety “Alidoro,” and the resistant variety “Hedasie” were included as controls (Table 2).

**Table.2. Spring wheat genotypes included in the study at Holetta, during 2022 and 2023 Cropping season**

| Genotype short name | Genotype | Pedigree | Origin | Reason for including in this study and *Stb* genes | Reference |
| --- | --- | --- | --- | --- | --- |
| Biq | BIQA | ETBW 6095 | EIAR | *Has Stb* (2, 3,5,7,8,9,14,18) | [25] |
| Mer | Meraro | *M/4/HAR 1709/ 3/M//24/E* | EIAR | *Has Stb* (5,6,7,8,11,13,16,17) | [25] |
| Ali | Alidoro | *HK-14-R251* | EIAR | *Has Stb* (2,3,4,5,6,7, 8,9, 11, 14,16,17,18) | [25] |
| Mad | Madda walabu | *TI/3/Fn/Th/Nar59 *2/4/Bol'S'* | EIAR | *Has Stb* (2,3,4,5,6, 8,9,13, 14,16,17,18) | [25] |
| Hul | Hulluka | *UTQUE96/3/PYN/BAU//MILAN* | OARI | *Has Stb* (3,4,5,7,8,13) | [25] |
| Kin | Kingbird | TAM200/TUI/6/PVN//CAR422/ANA/5/BOW/CROW//BUC/PVN/3/YR/4/TRAP#1 | EIAR | *Has Stb* (1,2,3,4,5,7, 8,11,13 ,17) | [25] |
| Dan | Danda’a | *KIRITATI//2*PBW65/2*SERI.1B* | EIAR | *Has Stb* (2,3,4,7,9,13,14,18) | [25] |
| Et | ET-13A2 | *UQ 105 SEL. X ENKOY* | EIAR | *Has Stb* (3,4,5,6,9,13,14,) | [25] |
| Pavon | Pavon-76 | *CMl399-D-4M-3Y-lM-l Y-lM-QY* | Mexico | *Has Stb* (3,5,6,8,9,11,12, 13, 14,17,18) | [25] |
| Sof | Sofumar | *LIRA 'S'/TAN"S"* | OARI | *Has Stb* (2,3,7,8,9,13,14,17) | [25] |
| Ki6295 | KI 6295-4A | *Romany X GB-GAMENYA* | EIAR | *Has Stb* (1,2,3,5,8,9,11,13, 14,16,17,18) | [25] |
| K6 | K 6290 Bulk | *(AF.MAYOXGEM)XROMANY* | EIAR | *Has Stb* (2, 3,5,7,8,9,14,18) | [25] |
| Hog | Hoggana | *PYN/BAU//MILAN* | EIAR | *Has Stb* (5,6,7,8,11, 13,16,17) | [25] |
| Sal | Salamouni | NA | Canada | Stb13 + Stb14* | [32] |
| 6B6 | 6B662 | NA | Brazil | Differential line tan spot | [33] |
| Cou | Coulter | DT-188/Dt-224//DT-182 | Israel | ** | [34] |
| Eri | Erik | Kitt/2/Waldron/Era | USA/ HRSW-KS | ** | [34] |
| Gle | Glenlea | Pemina*2/Bage/2/CB100 | CIMMYT | Differential line for tan spot | [33] |
| Nd | ND-495 | Justin*2/3/ND259/Conley//ND112 | Uruguay | ** | [34] |
| Ver | Veranopolis | K-44430; PI-214401; PI-297008; AFRC-5645 | Ammiad, Israel | Has *Stb2 + Stb6** | [35, 36] |
| Isr | Israel 493 | NA | Iran | Has the *Stb3* and *Stb6** | [[35]](https://bsppjournals.onlinelibrary.wiley.com/doi/10.1111/j.1365-3059.2004.01052.x#b16) |
| Tad | Tadina | PI-494096; AUS-24432; AUS-23036 | USA |  | [37, 38] |
| Sha | Shafir | K-62169 | CIMMYT | *Stb6** | [[39]](https://bsppjournals.onlinelibrary.wiley.com/doi/10.1111/j.1365-3059.2004.01052.x#b26) |
| Est | Estanzuela Federal | NA | Brazil | *Stb7** | [40] |
| Kk4 | KK4500 | NA | CIMMYT | Has the *Stb* (6,7,10,12)* | [38] |
| Km7 | KM7 | NA | CIMMYT | *Stb16** | [41] |
| Gon | Gondo/CBRD | NA | USA | ** | [34] |
| Br18 | BR 18 | NA | CIMMYT | ** | [34] |
| Cat | Catbird | NA | CIMMYT | Used as source of resistance by CIMMYT | [42] |
| Br34 | BR 34 | ALZ110 / 2* IAS54 /6/ TP /4/ TZPP /SON64 | CIMMYT | ** | [34] |
| Fro | Frontana | NA | CIMMYT | Veranopolis and KK4500 are derived from Frontana | [**[43]**](https://bsppjournals.onlinelibrary.wiley.com/doi/10.1111/j.1365-3059.2004.01052.x#b7) |
| Mut | Mutus | NA | Brazil | ** | [34] |
| Mur | Murga | NA | CIMMYT | Stb16* | [34] |
| Blo | Blouk #1 | NA | CIMMYT | ** | [34] |
| Chi | MP 4106 | Chiba//PRLII/cM65531/3/SKAUZ/BAV92 | CIMMYT | ** | [34] |
| Cia | CIANO T79 | CM31678-R-4Y-2M-21-0M | Mexico | ** | [34] |
| Sok | Line | SOKOLL//W15.92/WBLL1 | CIMMYT | ** | [34] |
| Wax | Line | WAXWING*2/CIRCUS | ICSISA SB6737 | ** | [34] |
| Hid | Hidasie | ANAC/3/PRL/SARA//TSI/VEE#5/4/CROC-1/AE.SQUAROSA(224)//OPATTA | CIMMYT | Has good resistance to STB |  |
| Etw86 | Line | ETW17-86 | EIAR | ** |  |
| Etw221 | Line | ETW17-221 | EIAR | ** |  |
| Etw246 | Line | ETW17-246 | EIAR | ** |  |
| Etw85 | Line | ETW17-85 | EIAR | ** |  |
| Etw115 | Line | ETW17-115 | EIAR | ** |  |
| Zer | Line | ZERBA-6/FLAG-6/3/TAM200/PASTOR//TOBA97 | EIAR | ** |  |

EIAR: Ethiopian Institute of Agricultural Research; CIMMYT: International Maize and Wheat Improvement Center; NA: Not available; *: STB differential line with known resistance major gene; **: the specific *Stb* genes were not clearly identified.

**Sowing and experimental design**

Both experiments were established according to a randomized complete block design, with three replications. The experimental area underwent thorough tillage, being plowed three times prior to sowing, and planting rows were established with hand-pulled row maker. Seeds were sown using the hand drilling technique at a depth of approximately five cm. Spring wheat genotypes were planted on 28 June, in accordance with the recommended sowing period for spring wheat at Holetta [44]. Each plot consisted of four rows, each measuring one meter in length and spaced 20 cm apart, with a seeding rate of 150 kg per hectare [44]. Inorganic fertilizers were uniformly applied across all plots at a rate of 100 kg per hectare of di-ammonium phosphate (DAP) and 150 kg per hectare of urea, following the recommendations for spring wheat cultivation. The entire amount of DAP and half of the urea were applied at the time of planting, while the remaining half of the urea was added during the mid-tillering phase. Hand weeding was performed to manage weed growth in the experimental field [44].

**Disease assessment and computation of disease variables**

Susceptibility and resistance levels were assessed based on the ability of the pathogen to infect host tissue and produce pycnidia. Disease assessments started at the early grain filling stage of wheat development [45], which is generally when the disease begins under Holetta conditions. Disease was assessed weekly for six weeks, ending when flag leaf infection reached 70% in susceptible genotypes. In each plot, five plants were randomly selected from each of the four corners and the center (X-shaped pattern), totaling 25 plants per plot for estimation of disease. Disease was assessed according to two criteria: disease severity (DS) and pycnidial density (PC).

Disease severity was estimated visually as the percentage of leaf area covered by necrotic lesions containing pycnidia. This assessment employed a modified version of the double-digit (00–99) scoring system developed by Saari and Prescott, which is based on a severity scale for evaluating foliar diseases in wheat [46, 47]. The scoring system disease severity (1 to 9) is as follows: 10% coverage corresponds to a score of 1, 20% coverage to 2, and so on until 90% coverage corresponding to a score of 9. These measurements were combined to calculate the overall disease severity using the following formula:

$$Disease severity \left( \% \right)=\left( \left( \frac{D1}{Y1} \right)*\left( \frac{D2}{Y2} \right) \right)*100$$

Where D1 represents disease vertical progression relative to plant height. The greater the disease reaches upper leaves, especially when it affects the flag leaf (which plays a key role in yield), the more serious the damage.  D2 reflects the diseased leaf area, and corresponds to the (1-9) score-scale described above. This provides the most straightforward indication of tissue damage and reduced ability for photosynthesis. Y1 is the maximum scale value for D1, which is the maximum possible score for the vertical progression of the disease, Y2 is the maximum scale value for D2, that is, the maximum possible score for the intensity of the diseased leaf area [46, 47]. The mean of disease severity over the six assessments was then computed and used for further analyses, and is referred later-on as "disease severity".

The area under the disease progress curve (AUDPC) of disease severity [48, 25] was calculated using the following formula:

$$AUDPC \%.day=\sum_{i=1}^{n-1} \frac{y_{i}+y_{i+1}}{2}\times(t_{i+1}-t_{i})$$

Where, y_i_ denotes disease severity at the i^th^ observation; t_i_ is the time, expressed in days, associated with the i^th^ observation; and n” is the total number of assessments.

The level of sporulation of lesions was estimated as pycnidial density within necrotic lesions using a 0–5 scale, where 0 = no sporulation; 1 = occasional pycnidia in a few lesions; 2 = low density of pycnidia in many or most lesions, usually unevenly distributed; 3 = even distribution of pycnidia at moderate density over most lesions; 4 = high density of pycnidia distributed over most lesions; and 5 = maximum pycnidial density [50, 51] (Table 3).

**Table 3. Pycnidial density within necrotic lesions: scoring scale, range of infection percentage, mid-point percentage used to calculate means across replicates, and corresponding genotype reaction (infection type) classification [50]**

| Pycnidia density Score | Range of pycnidia density within a lesion (%) | Mid-Point | Infection type |
| --- | --- | --- | --- |
| 0 | 0% | 0% | Immune (I) |
| 1 | 0.1–5% | 2.5% | Resistant (R) |
| 2 | 6–15% | 10.5% | Mod. Resistant (MR) |
| 3 | 16–30% | 23.0% | Mod. Susceptible (MS) |
| 4 | 31–40% | 35.5% | Susceptible (S) |
| 5 | 41–50% | 45.5% | Susceptible (S) |

The resistance levels of wheat genotypes were classified based on scores from genotype reaction (IT) obtained from the mean percentage of pycnidia density, calculated from six disease assessments. Genotype reactions (Infection types) were estimated and grouped using a 0–5 scale as described by Adhikari *et al*. and Louriki *et al*. [50, 51]. Genotypes with pycnidia density scores ranging from 0 to 1 were classified as resistant (R), a score of 2 as moderately resistant (MR), a score of 3 as moderately susceptible (MS), and scores of 4 to 5 as susceptible (S) [51].

All essential agronomic data were collected from the middle two rows within each plot. The data collection was conducted on both a plant and plot basis. For the analysis of plant-based data, a random selection of 10 plants from each plot was made to evaluate a range of characteristics. These characteristics included the number of tillers per plant, the number of kernels per spike, the spikelet count per spike, as well as measurements of plant height (cm), and spike length (cm). Additionally, further data were gathered on the density of productive tillers per square meter, the weight of a thousand kernels (grams), and the grain yield (t/ha) on a plot-specific basis.

**Statistical analyses**

In a first stage, the effects of genotype, year, and their interaction on disease severity (mean over six assessments), AUDPC of disease severity, and the agro-morphological variables were assessed with analyses of variance (ANOVA). The R statistical software (version 4.5.2) was used, following the standard procedures outlined by Gomez and Gomez [57]. Severity percentage data were transformed using arcsine transformation prior to analyses [58]. The validity of further statistical analyses, such as ANOVA, was ensured by using Bartlett's test to determine whether the error variances for distinct genotypes across different attributes were comparable. ANOVA and other parametric statistical tests require the assumption of homoscedasticity, which was examined prior to analysis. When ANOVA indicated significant differences among genotypes, mean comparisons were performed using the least significant difference (LSD) test at a 5% level of significance.

In a second stage, associations between disease severity (mean over six assessments), AUDPC of disease severity, and agronomic traits were assessed with multivariate analyses. Diversity in disease resistance among genotypes was assessed using cluster analysis, correspondence analysis, and principal component analysis (PCA) based on agro-morphological and disease-related traits.

Continuous variables were converted into categorical ones because correspondence analysis is based on contingency tables [52, 53]. Quartiles provide a common and robust discretization method, dividing data into four equal-frequency groups and accommodating skewed distributions [54, 55]. Continuous variables (disease and agronomic traits) were discretized into categorical variables, each one including four frequency-based groups (Q1–Q4). Quartile values for each variable are displayed in Table 4.

**Table 4. Quartile values from wheat genotype categorization based on disease, yield, and agronomic traits, used to classify variables into High, Medium, and Low groups with corresponding genotypes.**

| Trait | Q1(Low) | Medium (Q2/Q3) | Q3 (high) |
| --- | --- | --- | --- |
| Genotype | - | - | - |
| Severity | 0.7779 | 0.8514 | 0.9125 |
| AUDPC | 2027.7 | 2290.2 | 2577.2 |
| Pycnida density (P) | 2.5 | 10.5 | 23 |
| Plant height (PH) | 83.5 | 88.0 | 97.2 |
| Number of productive tiller per plant (NTPP) | 2.507 | 2.601 | 2.692 |
| Spike length (SL) | 6.53 | 7.27 | 7.73 |
| Number of seed per spike (NSPS) | 6.40 | 6.48 | 6.69 |
| Thousand kernel weight (TKW) | 30.0 | 33.0 | 36.3 |
| Yield tons per hectare (Yieldt.ha) | 2.08 | 2.31 | 2.83 |

Cluster analysis was performed using Euclidean distance as the dissimilarity measure and Ward’s minimum variance method as the clustering criterion. Correspondence analysis and PCA were used to explore associations among genotypes, disease variables, and agronomic traits. All analyses were conducted using R statistical software (version 4.5.2).

**ETHICS STATEMENT:** The study was approved by review and Ethics Committee, Harumaya University, Agriculture and Environmental Sciences School of Plant Sciences in February, 2022

**Results**

**Reaction of spring wheat genotype to *Zymoseptoria tritici***

The ANOVA revealed statistically significant effects (P ≤ 0.0001) of year, genotype, and the interaction between genotype and year on disease severity (mean over six assessments), AUDPC of disease severity, plant height, number of productive tillers per plant, spike length, number of seeds per spike, thousand kernel weights, and grain yield (Table 5, Table 6). This finding suggests that both disease level and agronomic traits were inconsistent across the years.

**Table 5. Analysis of variance for the effect of spring wheat genotype on septoria tritici blotch at Holetta, Ethiopia, during the 2022 and 2023 cropping seasons**

| Trait | Factor | SS | Mean Square | Pr > F | %SS | h_2_^2^ % |
| --- | --- | --- | --- | --- | --- | --- |
| Severity | Genotype | 3.233 | 0.074 | <.0001 | 42.81 | 0.7094 |
|  | year | 0.562 | 0.562 | <.0001 | 7.4 |  |
|  | Genotype*year | 2.994 | 0.07 | <.0001 | 39.6 |  |
|  | Residual | 0.763 | 0.0043 |  | 10.1 |  |
| AUDPC | Genotype | 56508295.41 | 1284279.44 | <.0001 | 42.3 | 0.7132 |
|  | year | 8214937.84 | 8214937.84 | <.0001 | 6.15 |  |
|  | Genotype*year | 54402880.83 | 1236429.11 | <.0001 | 40.7 |  |
|  | Residual | 14508570 | 81508.8 |  | 10.86 |  |

SS- a sum of squares; MS- mean squares; h_b_^2^- broad-sense heritability index.

The ANOVA results further indicate that genotype is the main factor contributing to variation in resistance to STB within the 45 spring wheat collection, accounting for 42.81% of the total variance in disease severity and 42.3% in AUDPC (Table 5). All genotypes showed high broad-sense heritability values (0.7094 for severity and 0.7132 for AUDPC; Table 5) for these traits, suggesting that resistance to STB can be enhanced through breeding.

The average disease severity was higher in 2022 (0.88) than in 2023 (0.79). In 2022, the weather at Holetta was cool and wet, ideal for fungal disease growth, while 2023 was relatively hot and dry. This affected both the spring wheat and the pathogen. The spring wheat genotype “Catbird” remained consistently susceptible over the years; however, the genotypes “Shafir” shifts from susceptible to moderately susceptible whereas, “Salamouni” shifted from resistant to moderately resistant in 2022 and 2023, respectively.. Nevertheless, their effectiveness is strongly influenced by genotype-by-environment interactions, making the resistance often specific to certain pathogen races and dependent on environmental conditions in the field. The observed changes in ranking for these differential lines may be attributed to shifts in the dominant pathogen races during those years.

**Table.6. Combined analysis of variance over the year for STB Severity, grain yield, and yield-related characters of spring wheat genotype grown at Holetta during the 2022 and 2023 growing seasons**

| Trait | MSG (44) | MSY (1) | MSGY (44) | MSB | MSE | Mean | Cv. | R^2^ | LSD |
| --- | --- | --- | --- | --- | --- | --- | --- | --- | --- |
| Severity | 0.0735^**^ | 0.5618^**^ | 0.068^**^ | 0.0299^**^ | 0.00428 | 0.83 | 7.86 | 0.90 | 0.0746 |
| AUDPC | 1284279.44^**^ | 8214937.84^**^ | 1236429.11^**^ | 468196.27^**^ | 81508.8 | 2317.52 | 12.32 | 0.89 | 325.28 |
| Plant height | 1041.048^**^ | 31948.03^**^ | 602.541^**^ | 167.315^**^ | 30.2661 | 92.063 | 5.98 | 0.95 | 6.268 |
| Number of tillers per plant | 0.0819^**^ | 12.752^**^ | 0.0704^**^ | 0.2326^**^ | 0.0677 | 2.590 | 10.05 | 0.62 | 0.2965 |
| spike length | 4.3226^**^ | 428.65^**^ | 4.2447^**^ | 0.2028^**^ | 0.3393 | 7.253 | 8.03 | 0.93 | 0.6636 |
| Number of seeds per spike | 0.9343^**^ | 0.60314^**^ | 0.8623^**^ | 0.4579^**^ | 0.1959 | 6.481 | 6.83 | 0.70 | 0.5043 |
| Thousand kernel weight | 228.58199^**^ | 11774.4^**^ | 249.1234^**^ | 126.693^**^ | 163.824 | 33.515 | 38.19 | 0.53 | 14.583 |
| Yield tons/hectare | 1.999^**^ | 0.641^**^ | 0.015^NS^ | 0.01^NS^ | 0.05 | 2.43 | 9.03 | 0.91 | 0.2503 |

*, ** Significant at p ≤ 0.05, and p ≤ 0.01 probability level respectively, () parenthesis indicate degrees of freedom.

MSG= Mean Squares of Genotype, MSY= Mean squares of year, MSGY = Mean square of genotype x year interaction, MSE = Mean squares of error, MSB= Mean squares of block, CV = Coefficient of variation.

Eleven genotypes showed a susceptible reaction and significantly high severity and AUDPC, while eleven genotypes showed a resistance reaction and significantly low severity and AUDPC. The most resistant genotype was Gondo, Israel-493, Salamouni, Pavon-76, Blouk#1, Sofumar, Coulter, Mutus, Erik and 6B662, while the most susceptible was “Etw17-246, Tadina, Etw17-86, Ki6295-4A, Glenlea, and Chiba //PRLII/cM65531/3/SKAUZ/BAV92, Catbird, Hoggana, CIANO T79, Veranopolis, Estanzuela Federal, ZERBA-6/FLAG-A resistance reaction was showed by eleven genotypes with considerably low severity and AUDPC, while a susceptible reaction was showed by fourteen genotypes with significantly high. The most resistant genotypes were “Etw17-115, Gondo, Israel-493, Blouk#1, Sofumar, Coulter, Mutus, Erik, and 6B662," while the most susceptible genotypes were " Etw17-246, Tadina, Etw17-86, Ki6295-4A, Glenlea." The results indicated that the spring wheat genotypes response was 22.2%, with 20% classified as resistant. Additionally, 26.7% and 24.4% were categorized as moderately resistant, while 22.2% and 24.4% were deemed moderately susceptible and finally, 28.8% and 31.1% of the genotypes were found to be susceptible during the 2022 and 2023 cropping seasons, respectively (Table. 5).

Of the 45 genotypes evaluated over two years, in 2022, Shafir showed the highest susceptibility, recording a leaf blotch score of 73.49% and pycnidia density of 30.95%, while Salamouni demonstrated the greatest resistance, with a leaf blotch severity of 47.46% and pycnidia density of 4.35%. In 2023, “Catbird” and “ZERBA-6/FLAG-6/3/TAM200/PASTOR//TOBA97” were the most vulnerable, showing leaf blotch severity (71.71%) and pycnidia density (45.47% and 35.36%), respectively. Meanwhile, “6B662” demonstrated the greatest resistance, achieving a leaf blotch severity score of 9.99% and pycnidia density score of 1.21% (Table 5). The data indicate variable infection responses across genotypes and growing seasons. Identifying genotypes that reliably display resistance year-round is essential for resistance breeding and as sources of resistance

The assessment of 45 wheat genotypes for resistance to STB revealed substantial variation in disease progression, measured by the area under the disease progress curve (AUDPC). Values ranged from 1,237 to 3,044, with a mean of 2,318. Based on these values, genotypes were grouped into four resistance categories: Resistant (R), Moderately Resistant (MR), Moderately Susceptible (MS), and Susceptible (S) (Fig 1)

The distribution was skewed toward higher disease severity, with 20 genotypes classified as susceptible, including ‘Catbird’ (AUDPC = 3,044). In contrast, the resistant category comprised only four genotypes: ‘Gondo/CBRD’, ‘ETW17-115’, ‘6B662’, and ‘Coulter’. ‘Gondo/CBRD’ showed the highest field resistance, with an AUDPC approximately 47% below the mean and 59% below the most susceptible entry. The Moderately Susceptible group formed the largest intermediate cluster, suggesting that although complete crop loss is unlikely, these varieties remain vulnerable under favorable conditions.

**Fig.1.** Distribution of Area Under the Disease Progress Curve (AUDPC) values for Septoria Tritici Blotch (STB) across 45 wheat genotypes. Bars are color-coded by resistance category: Resistant (dark green), Moderately Resistant (light green), Moderately Susceptible (yellow), and Susceptible (red). Horizontal dashed lines indicate threshold values for category classification. The population mean (AUDPC = 2,318) and range (1,237–3,044) are displayed.

The severity of STB among the 45 spring wheat genotypes showed a broad continuous distribution, ranging from 27.48% to 70.44%, with a mean of 54.71%. The population was skewed toward high infection levels. Genotypes were classified into four categories based on necrotic leaf area: Resistant (R), Moderately Resistant (MR), Moderately Susceptible (MS), and Susceptible (S) (Fig 2).

The Susceptible group was the largest, comprising 15 genotypes with more than 60% infected leaf area, including ‘Catbird’ (70.44%). In contrast, the resistant group included four genotypes (‘Gondo/CBRD’, ‘ETW17-115’, ‘6B662’, and ‘Coulter’) with severity below 35%. ‘Gondo/CBRD’ was the most resistant, with 27.48% severity—49.8% lower than the population mean. The Moderately Susceptible category formed a large cluster (n = 18), with values between 50% and 60%, indicating generally high vulnerability within the panel.

**Fig.2.** Percent disease severity of Septoria Tritici Blotch (STB) across 45 wheat genotypes. Bars represent the mean severity percentage for each genotype, color-coded by resistance category: Resistant (dark green), Moderately Resistant (light green), Moderately Susceptible (yellow), and Susceptible (red). Horizontal dashed lines indicate the classification thresholds for each resistance level. The overall population mean (54.71%) and range (27.48%–70.44%) are indicated in the header.

To examine the relationship between epidemic development rate and final disease pressure, STB severity and AUDPC were normalized to a 0–100 scale for direct comparison. A strong positive correlation was observed across the 45 genotypes, although differences emerged by resistance level. In highly susceptible genotypes, such as ‘Catbird’ and ‘KI6295-4A’, normalized AUDPC values exceeded normalized severity, indicating early onset and rapid pathogen spread. In contrast, a distinct slow-rusting phenotype was evident in the resistant group (Fig 3).

In resistant genotypes (‘Gondo/CBRD’, ‘ETW17-115’, ‘6B662’, and ‘Coulter’), normalized AUDPC was markedly lower than severity. For example, although ‘6B662’ and ‘ETW17-115’ reached severities of approximately 25–30%, their normalized AUDPC values were near the lower end of the scale. This pattern suggests effective adult plant resistance or delayed disease development that slows infection despite symptom expression. Overall, the mean severity (54.71%) and mean AUDPC (2,318) indicate high STB pressure, with ‘Gondo/CBRD’ emerging as the most stable resistant genotype across temporal and terminal measures (Fig 3).

**Fig.3.** Normalized comparison of Septoria Tritici Blotch (STB) severity and Area Under the Disease Progress Curve (AUDPC) across 45 wheat genotypes. Both traits were transformed to a 0–100 scale to facilitate direct comparison of disease accumulation over time (AUDPC) versus terminal infection levels (Severity). Genotypes are ranked by descending AUDPC values, with the highest susceptibility observed in ‘Catbird’ and the highest resistance in ‘Gondo/CBRD’.

The relationship between disease severity and the area under the disease progress curve (AUDPC) was evaluated to assess phenotypic consistency across 45 wheat genotypes. Regression analysis showed an exceptionally strong positive linear correlation, with a Pearson coefficient of r = 0.994 (p < 0.01) and R^2^ = 0.988. This near-perfect association indicates that terminal severity reliably predicts cumulative disease pressure during the growing season (Fig 4).

Genotypes clustered along the regression line according to resistance class. Highly resistant genotypes, such as ‘Gondo/CBRD’, occupied the lower-left region, with minimal terminal symptoms and low seasonal disease accumulation. In contrast, highly susceptible genotypes, including ‘Catbird’, clustered in the upper-right region, with high values for both traits.

Normalized analysis (0–100 scale) further clarified infection dynamics. While the traits closely aligned in intermediate genotypes, the most resistant lines showed disproportionately lower normalized AUDPC relative to terminal severity. This divergence suggests a slow-rusting mechanism, where epidemic development is delayed despite eventual necrosis at the end of the evaluation period (Fig 4).

**Fig.4.** Correlation between disease severity (%) and Area Under the Disease Progress Curve (AUDPC) for Septoria Tritici Blotch (STB) in 45 wheat genotypes. A near-perfect positive linear relationship was observed, with a Pearson correlation coefficient of r = 0.994 and a coefficient of determination of R^2^ = 0.988. Data points are color-coded by resistance group, illustrating the distinct clustering of highly resistant genotypes (bottom-left) versus highly susceptible genotypes (top-right).

**Table 5. Leaf blotch severity (LBS), infection type (IT), and pycnidia density (P) of bread wheat genotypes in response to STB at Holetta, Ethiopia (2022–2023)**

| Spring wheat Genotypes | 2022 | | | 2023 | | |
| --- | --- | --- | --- | --- | --- | --- |
|  | %LBS | P | IT | %LBS | P | IT |
| Etw17-246 | 50.46^MNO^ | 35.66^A^ | S | 68.97^ABCD^ | 38.56^AB^ | S |
| Tadina | 65.22^ABCDEFG^ | 35.66^A^ | S | 69.89^ABC^ | 45.47^A^ | S |
| Etw17-86 | 46.46^NO^ | 35.66^A^ | S | 69.89^ABC^ | 45.47^A^ | S |
| Ki6295-4A | 66.16^ABCDE^ | 35.66^A^ | S | 69.89^ABC^ | 34.71^ABC^ | S |
| Glenlea | 49.46^MNO^ | 35.66^A^ | S | 60.38^DEFGHIJ^ | 26.43^CDE^ | MS |
| Chiba//PRLII/cM65531/3/SKAUZ/BAV92 | 69.89^ABC^ | 35.66^A^ | S | 65.22^ABCDEF^ | 42.69^A^ | S |
| Catbird | 68.97^ABCD^ | 35.66^A^ | S | 71.71^A^ | 45.47^A^ | S |
| Hoggana | 63.29^BCDEFGHIJ^ | 35.66^A^ | S | 66.16^ABCDE^ | 30.95^BCD^ | S |
| CIANO T79 | 59.40^DEFGHIJKL^ | 35.66^A^ | S | 61.36^BCDEFGHI^ | 30.95^BCD^ | S |
| Veranopolis | 48.46^MNO^ | 35.66^A^ | S | 69.89^AB^ | 42.49^A^ | S |
| Estanzuela Federal | 63.29^BCDEFGHIJ^ | 35.66^A^ | S | 69.89^ABC^ | 42.49^A^ | S |
| ZERBA-6/FLAG-6/3/TAM200/PASTOR//TOBA97 | 53.46^HIJKLMNO^ | 35.66^A^ | S | 71.71^A^ | 35.66^ABC^ | S |
| Shafir | 73.49^A^ | 30.95^A^ | S | 59.40^EFGHIJK^ | 22.98^DE^ | MS |
| Etw17-221 | 55.45^GHIJKLMNO^ | 27.32^AB^ | MS | 64.26^ABCDEFG^ | 30.95^BCD^ | S |
| WAXWING*2/CIRCUS | 57.43^EFGHIJKLM^ | 27.32^BC^ | MS | 69.89^AB^ | 30.95^BCD^ | S |
| Meraro | 50.46^LMNO^ | 27.32^BC^ | MS | 38.56^OP^ | 18.14^EF^ | MS |
| BR 34 | 65.22^ABCDEFG^ | 22.98^C^ | MS | 57.43^EFGHIJK^ | 18.14^EF^ | MS |
| Murga | 59.40^DEFGHIJKLM^ | 22.98^C^ | MS | 59.40^EFGHIJK^ | 27.32^CDE^ | MS |
| Kk4500 | 66.16^ABCDEFG^ | 22.98^C^ | MS | 62.33^ABCDEFGH^ | 18.14^EF^ | MS |
| Biqa | 56.44^EFGHIJKLM^ | 22.98^C^ | MS | 51.46^IJKLM^ | 18.14^EF^ | MS |
| Hidasie | 61.36^CDEFGHIJKL^ | 22.98^C^ | MS | 57.43^EFGHIJK^ | 26.43^CDE^ | MS |
| SOKOLL//W15.92/WBLL1 | 64.26^ABCDEFG^ | 22.98^C^ | MS | 52.46^IJKLM^ | 18.14^EF^ | MS |
| K6290 Bulk | 63.29^ABCDEFGH^ | 22.98^C^ | MS | 68.97^ABCD^ | 26.43^CDE^ | MS |
| Frontana | 57.43^EFGHIJKLM^ | 14.45^D^ | MR | 46.46^LMNO^ | 10.50^GH^ | MR |
| Etw17-85 | 52.46^IJKLMNO^ | 10.50^DE^ | MR | 53.46^HIJKLM^ | 18.14^EF^ | MS |
| Alidoro | 48.46^MNO^ | 10.50^DE^ | MR | 55.45^GHIJKLM^ | 10.50^GH^ | MR |
| Nd-495 | 68.97^ABCD^ | 10.50^DE^ | MR | 46.46^LMNO^ | 10.50^GH^ | MR |
| Madda walabu | 57.43^EFGHIJKLM^ | 10.50^DE^ | MR | 55.45^FGHIJKL^ | 10.50^GH^ | MR |
| BR 18 | 64.26^ABCDEFG^ | 10.50^DE^ | MR | 50.46^JKLM^ | 14.45^FG^ | MR |
| KM7 | 50.46^MNO^ | 10.50^DE^ | MR | 60.38^CDEFGHI^ | 14.45^FG^ | MR |
| Hulluka | 61.36^CDEFGHIJKL^ | 10.50^DE^ | MR | 39.54^NOP^ | 10.50^GH^ | MR |
| Danda’a | 67.11^ABCDE^ | 10.50^DE^ | MR | 46.46^LMNO^ | 10.50^GH^ | MR |
| Kingbird | 65.22^ABCDEFG^ | 10.50^DE^ | MR | 40.52^NOP^ | 10.50^GH^ | MR |
| ET-13A2 | 52.46^JKLMNO^ | 7.11^EF^ | MR | 49.46^KLMN^ | 14.45^FG^ | S |
| Etw17-115 | 50.46^LMNO^ | 7.11^EF^ | MR | 11.76 ^SR^ | 2.54^JK^ | R |
| Gondo | 51.46^KLMNO^ | 4.35^FG^ | R | 8.73 ^S^ | 0.25^K^ | R |
| Israel-493 | 66.16^ABCDEF^ | 4.35^FG^ | R | 28.22 ^Q^ | 2.54^JK^ | R |
| Salamouni | 47.46^NO^ | 4.35^FG^ | R | 54.45^HIJKLM^ | 7.11^HI^ | MR |
| Pavon-76 | 72.61^AB^ | 4.35^FG^ | R | 39.54 ^NOP^ | 4.76^IJ^ | MR |
| Blouk#1 | 65.22^ABCDEFG^ | 2.23^G^ | R | 13.08 ^SR^ | 1.21^JK^ | R |
| Sofumar | 53.46^HIJKLMNO^ | 2.23^G^ | R | 45.47 ^MNO^ | 2.54^JK^ | R |
| Coulter | 51.46^KLMNO^ | 2.23^G^ | R | 18.14 ^R^ | 2.54^JK^ | R |
| Mutus | 63.39^BCDEFGHI^ | 2.23^G^ | R | 35.66^PQ^ | 2.54^JK^ | R |
| Erik | 62.33^BCDEFGHIJK^ | 2.23^G^ | R | 13.76 ^SR^ | 1.21^JK^ | R |
| 6B662 | 55.45^FGHIJKLMN^ | 2.23^G^ | R | 9.99 ^S^ | 1.21 ^JK^ | R |
| R^2^ | 0.68 | 0.96 |  | 0.942163 | 0.93 |  |
| CV | 7.69 | 10.69 |  | 7.85 | 16.02 |  |
| Mean | 0.88 | 0.42 |  | 0.79 | 0.43 |  |
| LSD | 0.1096 | 0.07 |  | 0.10 | 0.11 |  |

**Clusters of wheat genotypes**

The cluster analysis allowed identifying 8 clusters (Fig. 5), each cluster demonstrating a minimum similarity level of 1500 based on Euclidean distance within the UPGMA clustering framework using severity and AUDPC. These clusters were categorized into two distinct groups: Group A and Group B. Group A consisted of three clusters—Cluster I, Cluster II, and Cluster III— while Group B was represented by Five clusters, Cluster IV, V, VI, VII, VIII (Fig. 5).

Cluster I, included five genotypes, and has high mean values for severity, AUDPC, and Pycnidia, indicating it contains the most susceptible spring wheat genotypes.. This group contains the cultivar "Estanzuela Federal," which is used as a differential line for STB and carries the resistance genes *Stb7* and *Stb12*. However, under the conditions in Holetta, Ethiopia, it has shown susceptibility. Therefore, the genotypes in this cluster likely possess these resistance genes but display a susceptible response. Cluster II comprises six genotypes. Among them are two differential lines: “KK4500” (carrying *Stb6*, *Stb7*, *Stb10* and *Stb12*) and “Shafir” (carrying *Stb6*). Both show a moderately susceptible reaction, suggesting that other cultivars in this cluster may also carry these *Stb* genes. Cluster III contains fifteen genotypes, , including three known differential lines: Veranopolis (*Stb2* and *Stb6*), KM7 (*Stb16*), and Murga (*Stb16*), all of which carry resistance genes. These genotypes exhibit responses ranging from susceptible to moderately resistant, indicating that other genotypes in this cluster might also have resistance genes. Clusters IV, V, and VI together include twelve genotypes, or 26.7% of the total, with no differential lines present. The genotypes in these clusters show resistant to moderately resistant reactions. Cluster VII has two genotypes, one being the differential line Israel- 493 (*Stb3* and *Stb6*), which displays resistance. The other genotype, Meraro, also carries *Stb6* but shows a moderately susceptible reaction. This suggests that the resistance in Israel- 493 is likely due to the *Stb3* gene. Finally, Cluster VIII consists of five genotypes, including the differential line Salamouni (*Stb13* and *Stb14*). Both Salamouni and the other cultivars in these group exhibit resistant reactions, implying that these genotypes may carry *Stb* genes similar to those found in Salamouni (Fig.5) The resistance genotypes identified in the study (Blouk #1, 6B662, Coulter, Erik, Gondo, and ETW17-115) exhibit resistance to the pathotypes that affect the major *Stb* genes found in Veranopolis (*Stb2* and *Stb6*) and Estanzuela Federal (*Stb7*) (Fig 5).

**Table.6. Characteristics of Cluster Based on Mean, Standard Deviation and Standards Error of Mean**

| Number of cluster | Variable | Mean | Std.Dve | SEM |
| --- | --- | --- | --- | --- |
| cluster I (N=5) | Severity * | 68.16 | 1.398 | 0.625 |
|  | AUDPC | 2948.87 | 58.697 | 26.25 |
|  | Pycnidia | 4.33 | 0.204 | 0.091 |
| cluster II (N=6) | Severity | 64.75 | 1.491 | 0.609 |
|  | AUDPC | 2762.67 | 43.97 | 17.95 |
|  | Pycnidia | 3.44 | 0.43 | 0.176 |
| cluster III (N=15) | Severity | 58.42 | 1.86 | 0.480 |
|  | AUDPC | 2471.47 | 86.79 | 22.41 |
|  | Pycnidia | 3.09 | 0.89 | 0.23 |
| cluster IV (=3) | Severity | 28.95 | 1.28 | 0.737 |
|  | AUDPC | 1289.17 | 48.93 | 28.25 |
|  | Pycnidia | 0.94 | 0.35 | 0.20 |
| cluster V (N=3) | Severity | 35.72 | 1.81 | 1.04 |
|  | AUDPC | 1547.39 | 89.11 | 51.45 |
|  | Pycnidia | 0.78 | 0.096 | 0.06 |
| cluster VI (N=6) | Severity | 53.46 | 1.699 | 0.694 |
|  | AUDPC | 2216.67 | 57.34 | 23.41 |
|  | Pycnidia | 2.111 | 0.49 | 0.20 |
| cluster VII (N=2) | Severity | 45.88 | 1.745 | 1.234 |
|  | AUDPC | 1898.17 | 28.05 | 19.83 |
|  | Pycnidia | 2.083 | 1.295 | 0.92 |
| cluster VIII (N=5) | Severity | 50.013 | 0.748 | 0.33 |
|  | AUDPC | 2058 | 55.97 | 25.03 |
|  | Pycnidia | 1.5 | 0.5 | 0.224 |

*= Severity result scored from pycnidia bearing necrosis**Mean= cluster centroid, Std.Dve.=standard deviation, SEM= Standard Error of the Mean, Roman numbers from I to VIII = cluster numbers, N=number of genotype in that cluster,

Cluster (VIII) has the lowest standard deviation for severity (0.75), Cluster V has the lowest standard deviation for Pycnidia (0.096), and Cluster (VII) has the lowest standard deviation for AUDPC (28.05). Whereas the highest standard deviations are found in Cluster (VIII) for severity (1.86), Cluster (VII) for Pycnidia (1.295), and Cluster (V) for AUDPC (89.11). This suggests that spring wheat genotypes in Cluster (VIII) for severity, Cluster (V) for Pycnidia, and Cluster (VII) for AUDPC are tightly grouped with data points close to the cluster mean (high internal similarity). On the other hand, genotypes in Cluster (VIII) for severity, Cluster (VII) for Pycnidia, and Cluster (V) for AUDPC have the highest standard deviations, indicating their data points are widely spread, making these clusters less compact and more diverse internally (Table 6). According to the result, most clusters have standard error of the mean (SEM) values close to zero for severity and Pycnidia, showing that their means are estimated with high reliability.

B

A

Fig.5. UPGMA clustering of 45 bread wheat genotypes reaction to STB under natural infection conditions, Holetta, Ethiopia (2022/23)

**CI-CVIII = Roman numbers showing cluster; Metric variables are:- AUDPC and Severity

**Correlation and principal component analysis**

Disease severity was highly correlated to the AUDPC of disease severity, and both variables were strongly (0.88) correlated to pycnidia density (Fig. 6). Disease severity and AUDPC had a significant negative correlation with plant height, number of productive tillers per plant, spike length, thousand kernel weight, and grain yield (Fig 6). .

). However, the correlation between disease and thousand kernel weight (TKW) is very weak, with correlation coefficients of -0.321 for severity and -0.320 for AUDPC. This means there is essentially no linear relationship between disease severity or AUDPC and TKW. This suggests that while the disease significantly reduces overall yield by decreasing the number of grains, spikes, and tillers, it does not have a significant impact on the average weight of individual kernels produced (Fig. 6).

Fig.6. Scatter plot matrix showing mean severity scored from pycnidia bearing necrosis starting from early grain filling stage to flag leaf infection reach 70% with pycnidia bearing necrosis and AUDPC relation with yield and yield related trait on 45 spring wheat genotypes evaluated during 2022 and 2023 cropping season

**Multiple correspondence analysis**

The multiple correspondence analysis (MCA) biplot (Fig. 7) illustrates associations among 45 wheat genotypes and categorized disease and yield traits under STB epidemic conditions. The first two dimensions explained 35.9% of the total variation (Dim1 = 20.9%; Dim2 = 15.0%). Dimension 1 represented a resistance–susceptibility gradient, contrasting low disease categories (Severity_Low, AUDPC_Low) with high disease categories (Severity_High, AUDPC_High). Genotypes on the positive axis were associated with low disease pressure and superior yield traits (Yield_High, NTPP_High, NSPS_High), whereas those on the negative axis were linked to high disease and reduced yield. Dimension 2 further separated genotypes by grain weight (TKW) and plant height (PH).

High disease indicators (Severity_High, AUDPC_High, P_High) clustered on the left with susceptible genotypes such as Madda walabu, BR18, Shafir, and CIANO T79. The upper-right quadrant contained low disease markers (Severity_Low, AUDPC_Low, P_Low) associated with resistant genotypes including Frontana, Kingbird, and ETW17-85. High yield (Yldt.ha_High) and NSPS_High were positioned in the lower-right quadrant near genotypes combining resistance and yield potential (Gondo/CBRD, Alidoro). PH_High was located on the far right, while TKW_High appeared in the upper-left quadrant, suggesting that high kernel weight does not always align with resistance.

On Dimension 1, the right side was associated with Severity_Low, AUDPC_Low, Yield_High, NTPP_High, NSPS_High, and PH_High (e.g., Frontana, Kingbird, SOKOLL//W15.92/WBLL1, BR34, KI6295-4A, ETW17-85, Gondo/CBRD, Alidoro). The left side corresponded to Severity_High, AUDPC_High, Yield_Low, NSPS_Low, and NTPP_Low (e.g., Madda walabu, Murga, BR18, Shafir, MP4106, Catbird, KK4550, Hulluka, Salamouni). On Dimension 2, the upper quadrant was associated with TKW_High and low disease (Frontana, Kingbird, BR34, SOKOLL//W15.92/WBLL1, KI6295-4A), while the lower quadrant reflected moderate disease and intermediate agronomic performance (Erik, Coulter, 6B662, Tadina, ETW17-115, Sofumar, Veranopolis). Based on MCA, four groups were identified:
Group I (Resistant and high yielding): Frontana, Kingbird, SOKOLL//W15.92/WBLL1, BR34, KI6295-4A, ETW17-85, Gondo/CBRD. Group II (Moderately resistant): Pavon-76, ETW17-115, Sofumar, Veranopolis, Israel 493, Alidoro. Group III (Susceptible): Mutus, Coulter, Meraro, Glenlea, ETW17-86, ETW-221, CIANO T79, KK4500, Hoggana, K6290 Bulk, Shafir, Estanzuela Federal, MP4106. Group IV (Highly susceptible): Hulluka, Salamouni, Madda walabu, ZERBA-6/FLAG-6/3/TAM200/PASTOR//TOBA97, BR18, ND-495, WAXWING*2/CIRCUS, Catbird.

Fig.7. Ordination of nine variables by correspondence analysis. Active variables included Severity, AUDPC, PH (plant height), TKW (thousand kernel weight), SL (spike length), NTPP (number of productive tillers per plant), P (pycnidia density), grain yield, and 45 spring wheat genotypes.

**Principal component analysis**

Principal Component Analysis (PCA) showed that the first nine components explained 100% of the total variation among the 45 spring wheat genotypes for agronomic and *Zymoseptoria tritici*-related traits (suppl Table xxx). Based on the Kaiser criterion (eigenvalues > 1), only the first three components were considered significant, jointly explaining 73% of the total variance. PC1, PC2, and PC3 contributed 42%, 19%, and 11% of the variation, respectively, with PC1 (eigenvalue = 3.817) representing the primary source of morphological and pathological diversity. The sharp decline in standard deviation from PC1 (1.95) to subsequent components indicates that most of the variability is captured within the first few dimensions, supporting their use for multivariate analysis and genotype classification.

**Table 8. Eigenvalues, proportion of variance, and cumulative variance explained by the first nine principal components for agronomic and STB-related traits in 45 spring wheat genotypes.**

| Components | PC1 | PC2 | PC3 | PC4 | PC5 | PC6 | PC7 | PC8 | PC9 |
| --- | --- | --- | --- | --- | --- | --- | --- | --- | --- |
| eigenvalue | 3.817 | 1.702 | 1.024 | 0.743 | 0.669 | 0.499 | 0.341 | 0.201 | 0.003 |
| Standard deviation | 1.95 | 1.30 | 1.01 | 0.86 | 0.82 | 0.71 | 0.58 | 0.45 | 0.06 |
| Proportion of Variance | 0.42 | 0.19 | 0.11 | 0.08 | 0.07 | 0.06 | 0.04 | 0.02 | 0.00 |
| Cumulative Proportion | 0.42 | 0.61 | 0.73 | 0.81 | 0.88 | 0.94 | 0.98 | 0.99 | 1.00 |

The PCA biplot further illustrated the relationships between genotypes and traits, with the first two components explaining 61.3% of the total variance (Fig. 7). PC1 (42.4%) represented a strong yield–disease gradient, showing a clear negative association between disease traits (severity, AUDPC, and pycnidia) and yield-related traits such as grain yield, spike length, and number of seeds per spike. Resistant genotypes, including Frontana, Kingbird, Gondo, and Alidoro, were associated with higher yield performance, whereas susceptible genotypes such as Madda Walabu, Estanzuela Federal, and Catbird clustered with high disease level and reduced agronomic performance. PC2 (18.9%) further distinguished genotypes based on yield components, particularly thousand kernel weight and seeds per spike, reinforcing the identification of high-performing, STB-resistant genotypes.

Fig.8. Result of principal component analysis executed on over year diseases, yield and yield related variables of forty five different spring wheat genotypes included in the study, Holetta, Ethiopia.

**Discussion**

The field evaluation carried out in this study revealed a broad range of responses to STB among the tested spring wheat genotypes, highlighting the existence of substantial genetic variation for resistance. Similar levels of diversity have been documented in large-scale field screenings conducted in Ethiopia, Morocco, and across Europe [26, 51, 59]. The identification of genotypes with consistently low disease scores evidenced by reduced pycnidial density and limited lesion expansion suggests the presence of effective resistance mechanisms comparable to those reported in elite cultivars, landrace collections, and lines harboring effective *Stb* resistance genes. This variation in response is in agreement with numerous earlier studies that have categorized wheat germplasm into distinct resistance and susceptibility groups under both natural and artificially induced infection conditions [26, 59, 74]. The genotypic response was also found to interact significantly with environmental factors.

The interaction between genotype and year is influenced by environmental factors and the genotype response to the prevailing *Z. tritici* races. The notable interaction between genotype and year showed that the wheat lines performed inconsistently because of environmental variations across different years. In particular, years with favorable conditions such as abundant rainfall and extended leaf wetness, which promote STB development likely led to high disease severity in susceptible genotypes, causing their rankings to change compared to years with less favorable conditions. Conversely, dry and hot years tend to reduce disease severity, therefore decreasing differences in disease between susceptible and resistant genotypes. Due to variations in moisture, temperature, and pathogen populations, STB responses vary from year to year [59, 60].

The environment influences qualitative and quantitative resistance genes through two primary, interconnected pathways: by modulating the host resistance expression and by affecting the pathogen virulence and dispersal [21]. The field environment directly impacts the wheat plant, altering the expression or effectiveness of the *Stb* genes. Resistance conferred by some *Stb* genes, such as those in “Shafir” and “Salamouni,” can be temperature-dependent. Under cool temperatures—optimal for fungal development—some Stb genes may confer strong resistance. However, their effectiveness may decline at higher temperatures [61]. This instability is a major component of the genotype-by-environment variance observed in field trials. Environmental factors, such as water or heat stress, influence the host's growth stage and canopy density. Since *Stb* resistance often functions by prolonging the latent period (the time from infection to visible pycnidia), any factor that prematurely shortens the plant's life span (such as drought) can lead to disease escape, causing a susceptible genotype to appear resistant and thereby masking the true effect of the *Stb* gene [61]. The environment influences the severity and population structure of the pathogen, which directly challenges the *Stb* gene set present in the host spring wheat [15]. Wet, cool conditions (high moisture, low temperature) are optimal for *Z. tritici* spore germination, leaf infection, and spread [62, 63]. In dry, hot years with low disease pressure, even susceptible genotypes may exhibit minimal symptoms, rendering the resistance gene's effect negligible or difficult to measure. Conversely, in wet years with high disease pressure, susceptible genotypes fail drastically, fully testing the effectiveness of the *Stb* gene and resulting in the greatest genotype-by-environment separation (crossover interaction). The environment also controls the pathogen's generation time and mutation rate [64]. The widespread deployment of a single *Stb* gene exerts strong selective pressure, enabling fungal races that have evolved virulence (the ability to overcome the gene) to multiply rapidly [62, 15]. Once a new race overcomes the *Stb* gene, resistance breaks down entirely, and the gene becomes ineffective regardless of temperature or moisture conditions. For breeders, a high genotype-by-environment interaction for traits controlled by qualitative (*Stb*) genes and quantitative trait loci (QTL) necessitates selection for stability or the stacking of multiple genes (gene pyramiding). Gene stacking provides broader resistance, making it much more difficult for the pathogen to overcome the entire set and reducing drastic performance fluctuations caused by single-gene breakdown or environmental variation.

To date, the assessment of wheat genotypes for their resistance to STB has been conducted in both protected and open field conditions, using susceptible checks as controls. However, including differential lines in the set of spring wheat genotypes to be screened is crucial for identifying which *Stb* gene is effective, determining the resistance of specific wheat lines to particular pathotypes, and understanding the infection types. This information enables researchers to postulate the presence of genes in unknown wheat lines [65].

Wheat genotypes show different degrees of resistance to STB. Research in Ethiopia has shown considerable variation in resistance levels among bread wheat, durum wheat, and triticale [22]. Molecular analyses conducted by Tilahun *et al*. [25] revealed significant genetic diversity in resistance among 180 genotypes of Ethiopian bread wheat. Genome-Wide Association Studies (GWAS) performed on Ethiopian wheat have successfully identified key genomic regions and novel germplasm sources that offer reliable resistance to STB. These results emphasize the intricate, polygenic foundation of resistance and stress the significance of considering Genotype-by-Environment interactions [26, 27].

Ahmet [61] observed a range of responses among wheat genotypes, varying from resistant to vulnerable to STB. In the same way, Odilbekov *et al*. [67] noted that immune reactions, highly resistant responses, and very susceptible reactions were not detected. Additionally, Omrani *et al*. [68] evaluated 53 elite wheat lines against STB at the adult plant stage under natural infection circumstances during the 2020 and 2021 growing seasons, discovering that the materials showed moderately resistant, moderately susceptible, and susceptible responses. Their research also demonstrated strong virulence in the resistance genes Stb3, Stb4, Stb7, Stb8, and Stb9, whereas avirulence was observed in Stb11 and Stb18 during both years. Furthermore, Muche [69] pointed out considerable differences in reactions to STB among commercially available bread wheat varieties and advanced lines, spanning from resistant to susceptible. This observation is supported by results from Teklay *et al*. [28], which recorded varied reactions among bread wheat varieties to STB diseases in Tigray, Ethiopia

The cluster analyses led to the identification of a group of genotypes (GIVE THE NAMES FOF THE 3 GENOTYPES HERE)which may prove to be highly beneficial for the development of STB- resistant varieties, as well as for other breeding initiatives (cluster IV, figure xxx). .These genotypes could be incorporated into hybridization programs aimed at developing varieties that are both resistant to STB and high-yielding.

**Conclusion**

This study highlights variation in STB resistance among 45 bread wheat genotypes evaluated in Ethiopia, identifying resistant cultivars such as Blouk #1, 6B662, Coulter, Erik, Gondo, and ETW17-115, and the highly susceptible Catbird. Adding differential lines to the screening program helps identify the pathotypes each line resists, facilitating gene postulation. We identified genotypes (Blouk #1, 6B662, Coulter, Erik, Gondo, and ETW17-115) resistant to pathotypes affecting the primary Stb genes in Veranopolis (Stb2 and Stb6), Shafir (Stb6), and Estanzuela Federal (Stb7). The study also showed strong virulence on Stb2, Stb6, and Stb7, and avirulence on Stb3, Stb13, Stb14, and Stb16. These findings support incorporating resistant genotypes into breeding programs to enhance yield and reduce disease impact. Although resistant genotypes were identified, the genetic basis of their resistance remains unknown. Molecular analyses (QTL mapping, genome-wide association studies) are needed to identify the responsible genes or alleles. Further studies should determine the combining ability of these lines and their performance when crossed with high-yielding, susceptible varieties.

**CONFLICT OF INTEREST STATEMENT: The authors declare that they have no conflict of interest.**

**ACKNOWLEDGEMENTS**

For providing the experimental material, and research facility, we thank Holetta agricultural research center Breeding and Pathology program. Many thanks to plant pathology research stuff for unreserved support during the research period. We thank Ethiopian Institute of Agricultural research for the fund.

**DATA AVAILABILITY STATEMENT:** The data uploaded as minimal data set of the study used in statistical analysis.

**Reference**

1. CSA (Central Statistical Authority). Agricultural Sample Survey 2020/21 (2013 E.C). Report on Livestock and Livestock Characteristics. Volume II, Statistical Bulletin 589, Central Statistical Authority, 2021; Addis Ababa.
2. USDA. World agricultural production. Circular series. WAP 11–22. 2023. <https://apps.fas.usda.gov/PSDOnline/Circulars/2023/08/production.pdf>.
3. USDA. World agricultural production. Circular series. Report Number: ET2025-0011. [Ethiopia: Grain and Feed Annual | USDA Foreign Agricultural Service](https://www.fas.usda.gov/data/ethiopia-grain-and-feed-annual-8)
4. Tesfaye Z, Girma T, Tanner D, Verkuijl H, Aklilu A and Mwangi W. Adoption of Improved Bread Wheat Varieties and Inorganic Fertilizer by Small Scale Farmers in Yelma Dansa and Farta Districts of Northwestern Ethiopia. Mexico, D.F. 2001; Ethiopian Agricultural Research Organization (EARO) and International Maize and Wheat Improvement Center (CIMMYT).
5. Mengistu H, Getaneh W, Yeshi A, Rbka D, Ayele B. Wheat Pathology Research in Ethiopia. In: Hailu,G.,Tanner,D.G.,Mengistu,H.(Eds.),Wheat Research in Ethiopia: a Historical Perspective. IAR/CIMMYT, Addis Ababa, 1991; pp.173-217.
6. Endale H and Getaneh W. Survey of Rust and Septoria Leaf Blotch Diseases of Wheat in Central Ethiopia and Virulence Diversity of Stem Rust *Puccinia graminis*f. sp.*tritici*.” *Advances in Crop Science and Technology,* 2015; 3(2), 1-5.
7. Yitagesu T, Alemayehu C, Bekele K. Survey of *Septoria tritici* blotch of bread wheat (*Triticum aestivum* L.) in the central highlands of Ethiopia. *American Journal of Bioscience and Bioengineering.* 2018; 6(5): 36-41
8. Meyer M, Bacha N, Tesfaye T, Alemayehu Y, Abera E, Hundie B. Wheat rust epidemics damage Ethiopian wheat production: A decade of field disease surveillance reveals national-scale trends in past outbreaks. *PLoS ONE*. 2021; 16(2): e0245697. https://doi.org/10.1371/journal.pone.0245697
9. Alemar S and Temam H. Epidemics of *Septoria tritici* Blotch and its Development over Time on Bread Wheat in Haddiya Kambata Area of Southern Ethiopia. *Journal of Biology, Agriculture and Healthcare,* 2016; 6(1), 47-57.
10. Bockus WW, Bowden RL, Hunger RM, Morrill WL, Murray TD, and Smiley RW. Compendium of Wheat Diseases and Pests: Third Edition. *American Phytopathological Society*, 2010; St. Paul, MN
11. Heick TM, Justesen AF, Jorgensen LN. Resistance of wheat pathogen Zymoseptoria tritici to DMI and QoI fungicides in the Nordic-Baltic region - a status. *Eur J Plant Pathol*. 2017;149(3):669–82.
12. Cools HJ, Fraaije BA. Update on mechanisms of azole resistance in *Mycosphaerella graminicola* and implications for future control. *Pest Manag Sci*. 2013;69(2):150–5.
13. Yang N, Ovenden B, Baxter B, McDonald MC, Solomon PS and Milgate A. Multi-stage resistance to *Zymoseptoria tritici* revealed by GWAS in an Australian bread wheat diversity panel. *Front. Plant Sci*. 2022; 13:990915. doi: 10.3389/fpls.2022.990915
14. Suffert F, Le Prieur S, Gelisse S, Dzialo E, Saintenac C, Marcel TC. Estimating the frequency of Virulence against an *Stb* gene in *Zymoseptoria tritici* populations by bulk phenotyping on checkerboard microcanopies of wheat NILs. *plant pathology*. 2024;73(6):1573-1585, <https://doi.org/10.1111/ppa.13894>
15. Brown JKM, Chartrain L, Lasserre-Zuber P, and Saintenac C. Genetics of Resistance to *Zymoseptoria tritici* and Applications to Wheat Breeding. *Fungal Genetics and Biology,* 2015; 79(1), 33-41. <https://doi.org/10.1016/j.fgb.2015.04.017>
16. Dreisigacker S, Wang X, Martinez Cisneros BA, Jing R, Singh PK. Adult-plant Resistance to *Septoria tritici* Blotch in Hexaploid Spring Wheat. *Theor Appl Genet,* 2015;128:2317–2329. <https://doi.org/10.1007/s00122-015-2587-9>
17. [Eriksen](https://pubmed.ncbi.nlm.nih.gov/?term=Eriksen+L&cauthor_id=12783168)  L, [Borum](https://pubmed.ncbi.nlm.nih.gov/?term=Borum+F&cauthor_id=12783168) F, [Jahoor](https://pubmed.ncbi.nlm.nih.gov/?term=Jahoor+A&cauthor_id=12783168) A. Inheritance and localization of resistance to *Mycosphaerella graminicola* causing *Septoria tritici* blotch and Plant Height in the Wheat (*Triticum aestivum* L.) Genome with DNA Markers, *Theor Appl Genet*. 2003;107(3):515-27. doi: 10.1007/s00122-003-1276-2.
18. Chartrain L, Brading PA, Makepeace JC, Brown JKM. Sources of Resistance to *Septoria tritici* Blotch and Implications for Wheat Breeding. *PlantPathol*. 2004; 53(4),454–460. <https://doi.org/10.1111/j.1365-3059.2004.01052.x>
19. Simon MR, Ayala FM, Corda CA, Order MS, Boerner A. Molecular Mapping of Quantitative Trait Loci Determining Resistance to *Septoria tritici* Blotch Caused by *Mycosphaerella graminicola*. *Euphytica*. 2004; 138:41-48.
20. Tabib Ghaffary SM, Faris JD, Friesen TL, Visser RG, van der Lee TA, Robert O, Kema GH. New broad-spectrum resistance to septoria tritici blotch derived from synthetic hexaploid wheat. *Theor Appl Genet*. 2012;124(1):125-42. doi: 10.1007/s00122-011-1692-7. Epub 2011 Sep 13. PMID: 21912855; PMCID: PMC3249545.
21. Langlands-Perry C, Pitarch A, Lapalu N, Cuenin M, Bergez C, Noly A, et al. Quantitative and qualitative plant-pathogen interactions call upon similar pathogenicity genes with a spectrum of effects. *Front Plant Sci*. 2023;14:1128546. doi: 10.3389/fpls.2023.1128546.
22. Kema GHJ, Verstappen ECP, Todorova M, Waalwijk C. Successful Crosses and Molecular Tetrad and Progeny Analyses Demonstrate Heterothallism in *Mycosphaerella graminicola. Curr Genet.* 1996; 30: 251-258.
23. Eyal Z, Amiri Z, and Wahl I. Physiologic Specialization of *Septaria tritici.* *Phytopathology.* 1973; 63(1):1087
24. Eyal Z, Scharen A L, Huffman M D, and Prescott J M. Global Insights in to Virulence Frequencies of *Mycosphaerella graminicola. Phytopathology.* 1985; 75:1456-1462.
25. Tilahun M, Teklehaimanot H, Bekele G A, and Kassahun T. Virulence Variability of Ethiopian *Zymoseptoria tritici* Isolates and Efficacy of Wheat Genotypes and *Stb* Resistance Genes Against the Isolates. *Eur J Plant Pathol.,* 2020; 158(4):895-910 <https://doi.org/10.1007/s10658-020-02125-3>
26. Kassie MM, Abebe TD, Desta EA, Tadesse W. Genome-wide association analysis of *Septoria tritici* blotch for adult plant resistance in elite bread wheat (*Triticum aestivum* L) genotypes. *PLoS ONE*. 2025; 20(3): e0317603. https://doi.org/10.1371/journal.pone.0317603
27. Yosef G., Kidane B N, Hailemariam DK, Mengistu, CF, Mario E P, Matteo D. Genome-Wide Association Study of *Septoria tritici* Blotch Resistance in Ethiopian Durum Wheat Landraces, *Front. Plant Sci*. 2017; (8) | https://doi.org/10.3389/fpls.2017.01586
28. Teklay AT and Zeray S G. Occurrence and Intensity of Wheat *Septoria tritici* Blotch and Host Response in Tigray, Ethiopia. *Crop Protection.* 2015; 68: 67-71
29. Yohannes A, Birhanu F and Yeshambel A. Field Response and Identification of Bread Wheat Genotypes to *Septoria tritici* Blotch in the Highlands of North Gondar. *Journal of Current Research in Food Science*, 2020; *1*(2), 09-17.
30. Bekle K and Yitagesu K . Phenotyping of Bread and Durum Wheat Accessions, lines and Varieties for *Septoria* leaf blotch (SLB) and *Fusarium* head blight at Holetta , Ethiopia. Eshetu Derso, Bedada Girma (eds) in *Proceedings of the Delivering Genetic Gain in Wheat (DGGW) Project Closing Workshop*. Addis Ababa, Ethiopia, 2020; pp.120.
31. Tamene L, Amede T, Kihara J, Tibebe D, Schulz S. (eds.). A review of soil fertility management and crop response to fertilizer application in Ethiopia: towards development of site- and context-specific fertilizer recommendation. CIAT Publication No. 443. International Center for Tropical Agriculture (CIAT), Addis Ababa, Ethiopia. 2017; 86 p. Available at:http://hdl.handle.net/10568/82996
32. McIntosh R A, Devos K M., and Dubcovsky J V. Catalogue of gene symbols for wheat supplement retrieved from http://wheatpwusdagov/ggpages/wgc/upd html.2007.
33. Singh P K, Singh RP, Duveiller E, Mergoum M, Adhikari TB, Elias EM. Genetics of wheat–*Pyrenophora tritici-repentis* interactions, *Euphytica*. 2010; 171:1–13 DOI 10.1007/s10681-009-0074-6
34. Pawan Singh , Scientist and Head of the Wheat Pathology, Global Wheat Program at International Maize and Wheat Improvement Center (CIMMYT), Mexico
35. Wilson RE. Inheritance of resistance to *Septoria tritici* in wheat. In: Scharen AL (ed) Septoria of Cereals; Proceedings of the Workshop. Bozeman, Montana State University. 1985. pp 33–35.
36. Brown JKM, Kema GHJ, Forrer HR, Verstappen ECP, Arraiano LS, Brading PA, Foster EM, Fried PM, Jenny E. Resistance of wheat cultivars and breeding lines to *Septoria tritici* blotch caused by isolates of *Mycosphaerella graminicola* in field trials. *Plant Pathol.* 2001; 50(1):325–338.
37. Adhikari TB, Yang X, Cavaletto JR, Hu X, Buechley G, Ohm HW, Shaner G. Molecular mapping of *Stb1*, a potentially durable gene for resistance to *Septoria tritici* blotch in wheat. *Theor Appl Genet*. 2004; 109(1):944–953.
38. Chartrain L, Joaquim P, Berry ST, Arraiano LS, Azanza F, Brown JKM. Genetics of resistance to *Septoria tritici* blotch in the Portuguese wheat breeding line TE 9111. *Theor Appl Genet*. 2005; 110(1):1138–1144.
39. Kema GHJ, Verstappen ECP, Waalwijk C. Avirulence in the wheat *Septoria tritici* leaf blotch fungus *Mycosphaerella graminicola* is controlled by a single locus. *Mol Plant Microbe Interac*t. 2000; 13:1375–1379.
40. McCartney CA, Bruˆ le´-Babel AL, Lamari L, Somers DJ. Chromosomal location of a race-specific resistance gene to *Mycosphaerella graminicola* in the spring wheat ST6. *Theor Appl Genet*.2003; 107:1181–1186.
41. Dalvand M, Zafar D, Pari S M J, Roohparvar R, & Ghafari S M T. Studying genetic diversity in *Zymoseptoria tritici*, causal agent of *Septoria Tritici* blotch, by using ISSR and SSR markers. *Journal of Agricultural Science and Technology*. 2018; 20(6), 1307–1316.
42. Dubin HI and Rajaram S. Breeding disease-resistance wheat for tropical highlands and lowlands. *Annu. Rev. Phytopathol*. 1996; 34:503-526.
43. Eyal Z. The *Septoria tritici* and *Stagonospora nodorum* blotch diseases of wheat. *Eur J Plant Pathol*. 1999; 105:629–641.
44. Tufa T, Haji K, and Tolera A. Influence of Seed Rate and Row Spacing on Growth and Yield of Bread Wheat in Chelia District of West Showa Zone, Ethiopia, *Journal of Natural Sciences Research*. 2019;9(5): ISSN 2224-3186 (Paper) ISSN 2225-0921 (Online), DOI: 10.7176/JNSR
45. Zadocks J C, Chang T T, and Konzak C F A. Decimal Code for the Growth Stages of Cereals. *Weeds Res*, 1974; 14:415-421
46. Saari EE and JM Prescott.  A Scale for Appraising the Foliar Intensity of Wheat Diseases. *Plant Disease Reporter.* 1975; 59 (5): 377–380.
47. Eyal Z, and Levy E. Variations in Pathogenicity Patterns of *Mycosphaerella graminicola* within *Triticum* spp in Israel.” *Euphytica*. 1987; 36: 237–250. <https://doi.org/10.1007/BF00730670>
48. Madden LV, Hughes G, Van den Bosch F.  The Study of Plant Disease Epidemics. 2007; St. Paul, MN, USA: APS Press.
49. Eyal Z, and Brown MB. A quantitative method for estimating density of *Septaria tritici* pycnidia on wheat leaves. *Phytopathology*. 1976; 66:11-14.
50. Adhikari T B, Anderson JM, and Goodwin SB,. Identification and Molecular Mapping of a Gene in Wheat Conferring Resistance to *Mycosphaerella graminicola*. *Phytopathology,* 2003; 93(9), 1158-1164. doi: 10.1094/PHYTO.2003.93.9.1158.
51. Louriki S, Rehman S, Hanafi S, Bouhouch Y, Al-Jaboobi M, Amri A, Douira A, and Tadesse W. Identification of Resistance Sources and Genome-Wide Association Mapping of Septoria Tritici Blotch Resistance in Spring Bread Wheat Germplasm of ICARDA. Frontiers in Plant Science, 12. 2021.https://doi.org/10.3389/fpls.2021.600176.
52. Lestari K. E., Utami M. R., and Yudhanegara M. R. Sequential exploratory design by performing correspondence analysis to investigate procedural fluency of undergraduate student. *AIP Conference Proceedings*, 2023, *2588*(1), 050004. [https://doi.org/10.1063/5.0111974](https://www.google.com/search?q=https://doi.org/10.1063/5.0111974)
53. Mishra P., Pandey C. M., Singh U., and Gupta, A. (2018). Scales of measurement and presentation of statistical data. *Annals of Cardiac Anaesthesia*, 2018, *21*(4), 419. [https://doi.org/10.4103/aca.aca_131_18](https://www.google.com/search?q=https://doi.org/10.4103/aca.aca_131_18)
54. Canova S., Cortinovis D. L., and Ambrogi F. How to describe univariate data. *Journal of Thoracic Disease*,2017, *9*(6), 1741–1743. <https://doi.org/10.21037/jtd.2017.05.80>
55. Maslove D. M., Podchiyska T., and Lowe H. J. Discretization of continuous features in clinical datasets. *Journal of the American Medical Informatics Association*, 2013, *20*(3), 544–553. <https://doi.org/10.1136/amiajnl-2012-000929>
56. Mabikwa O. V., Greenwood D. C., Baxter P. D., and Fleming S. J. Assessing the reporting of categorised quantitative variables in observational epidemiological studies. *BMC Health Services Research*, 2017, *17*(1). <https://doi.org/10.1186/s12913-017-2137-z>
57. Gomez KA, and Gomez AA. Statistical Procedures for Agricultural Research. 2^nd^ Edition, John Wiley and Sons, New York. 1984. 680 p.
58. Sokal, R. R., and Rohlf, J. F. Biometry *(3^rd^ ed.)*. 1995; New York: Freeman.
59. Riaz A, Kockappelgren P, Hehir J G, Kang J, Meade F, Cockram J, *et al*.  Genetic analysis using a multi-parent wheat population identifies novel sources of Septoria tritici resistance.  *Genes*. 2020; 11(8), 887. blotch  <https://doi.org/10.3390/genes11080887>
60. Prahl KC, Klink H, Hasler M, Verreet J-A, Birr T. Will Climate Change Affect the Disease Progression of *Septoria Tritici* Blotch in Northern Europe? *Agronomy*. 2023; 13(4):1005. https://doi.org/10.3390/agronomy13041005
61. Chungu C, Gilbert J, and Townley-Smith F. *Septoria tritici* blotch development as affected by temperature, duration of leaf wetness, inoculum concentration, and host. *Plant Dis*. 2001;85(1):430-435.
62. Tiley Anna MM, Karki Sujit J, and Feechan Angela. Zymoseptoria tritici. In: eLS. John Wiley & Sons, Ltd, Chichester. 2018. DOI: 10.1002/9780470015902.a0027948
63. Porras R, Miguel-Rojas C, Lorite IJ, Pérez-de-Luque A, Sillero JC. Characterization of Durum Wheat Resistance against *Septoria Tritici* Blotch under Climate Change Conditions of Increasing Temperature and CO_2_ Concentration. *Agronomy*. 2023; 13(10):2638. <https://doi.org/10.3390/agronomy13102638>
64. Suffert F, Ravigné V, Sache I. Seasonal changes drive short-term selection for fitness traits in the wheat pathogen *Zymoseptoria tritici*. *Appl Environ Microbiol*. 2015; 81:6367–6379. doi:10.1128/AEM.00529-15.
65. Tabib Ghaffary M S, van der Lee TAJ, Verstappen ECP, and Kema GH J.  Screening for resistance to septoria tritici blotch, the major wheat disease in Western Europe. Poster session presented at 7th International *Mycosphaerella* and *Stagonospora* Symposium, Ascona, Switzerland. 2008.  <https://edepot.wur.nl/189351>
66. Ahmet ÇAT. Evaluation of Some Bread Wheat (*Triticum aestivum* L.) Varieties Reaction to *Septoria Tritici* Blotch Disease, *Turk J Agric Res.* 2023; 11(1): 18-25. doi: 10.19159/tutad.1373924
67. Odilbekov F, Armoniené R, Koc A, Svensson J and Chawade A. GWAS-Assisted Genomic Prediction to Predict Resistance to *Septoria Tritici* Blotch in Nordic Winter Wheat at Seedling Stage. *Front. Genet*. 2019; 10:1224. doi: 10.3389/fgene.2019.01224
68. Omrani A, Roohparvar R, Shahbazi K. Reaction of Promising Wheat Lines to Septoria Leaf Blotch Disease at the Seedling and Adult Plant Stages. *Applied Entomology and Phytopathology*. 2023; 90(2): 195-208.
69. Muche A G. Assessment of Ethiopian Advanced Bread Wheat Genotypes and Varieties to *Septoria tritici* leaf blotch, *RJOAS*. 2022; 6(126), 181, UDC 633; DOI 10.18551/rjoas.2022-06.21
70. Amelio A, Tagarelli A. [Encyclopedia of Bioinformatics and Computational Biology](https://www.sciencedirect.com/referencework/9780128114322/encyclopedia-of-bioinformatics-and-computational-biology) , 2018 pp 437-448
71. Vasileios C. Pezoulas, Themis P. Exarchos, Dimitrios I. Fotiadis [Medical Data Sharing, Harmonization and Analytics](https://www.sciencedirect.com/book/9780128165072/medical-data-sharing-harmonization-and-analytics) , 2020 pp 227-309
72. Bojan Žlahtič, Peter Kokol, Helena Blažun Vošner and J. Završnik. "The role of correspondence analysis in medical research." *Frontiers in Public Health*, 12.2024. https://doi.org/10.3389/fpubh.2024.1362699..
73. Fellenberg KN, Hauser, B. Brors, A. Neutzner, J. Hoheisel and M. Vingron. "Correspondence analysis applied to microarray data." *Proceedings of the National Academy of Sciences of the United States of America*, 98. 2001, 10781 - 10786. <https://doi.org/10.1073/pnas.181597298>.
74. Lakachew Binalf, H. Shifa and Wuletaw Tadesse. "Association mapping of septoria tritici blotch resistance in bread wheat in Bale and Arsi highlands, Ethiopia." *Heliyon*, 10.2024, <https://doi.org/10.1016/j.heliyon.2024.e32265>.
75. Tilahun Mekonnen, C. Sneller, T. Haileselassie, Cathrine Ziyomo, B. Abeyo, S. B. Goodwin, D. Lule and K. Tesfaye. "Genome-Wide Association Study Reveals Novel Genetic Loci for Quantitative Resistance to Septoria Tritici Blotch in Wheat (Triticum aestivum L.)." *Frontiers in Plant Science*, 12. 2021, https://doi.org/10.3389/fpls.2021.671323.
76. Dominika Piaskowska, U. Piechota, M. Radecka-Janusik and P. Czembor. "QTL Mapping of Seedling and Adult Plant Resistance to Septoria Tritici Blotch in Winter Wheat cv. Mandub (Triticum aestivum L.)." *Agronomy*, 11. 2021, 1108. https://doi.org/10.3390/agronomy11061108.
77. Qalavand F, M. Nasr-Esfahani, J. Vatandoost and D. Amin Azarm. "Transcriptome - based analysis of resistance mechanism to Bipolaris sorokiniana - wheat common root rot disease.." *Plant biology.*2022, https://doi.org/10.1111/plb.13470.
78. Hasan, M., Mia, M., Ahmed, J., Karim, M., Islam, A., & Mohi-Ud-Din, M. Heat stress tolerance in wheat seedling: Clustering genotypes and identifying key traits using multivariate analysis. *Heliyon*, 10.2024, https://doi.org/10.1016/j.heliyon.2024.e38623.
79. [Chahal](https://www.google.com.et/search?tbo=p&tbm=bks&q=inauthor:%22G.+S.+Chahal%22) G S, [Gosal](https://www.google.com.et/search?tbo=p&tbm=bks&q=inauthor:%22S.+S.+Gosal%22) S S. Principles and Procedures of Plant Breeding: Biotechnological and Conventional Approaches, CRC Press, 2002, Alpha Science International Ltd. Harrow, U.K.
