# Supplementary material for: Field evaluation of spring wheat genotypes reveals differential resistance to Zymoseptoria tritici in Ethiopia
Source: PLoS One. 2026 Jul 10;21(7):e0353375. doi: 10.1371/journal.pone.0353375 (PMC13353992; doi:10.1371/journal.pone.0353375)
Supplement: Attachment — Submitted filename: Response_to_Reviewers_auresp_4.docx [file pone.0353375.s017.docx]

**Response to Reviewers**

**Manuscript ID:** PONE-D-25-41622R2
**Title:** Field Evaluation of Spring Wheat (*Triticum aestivum* L.) Genotypes Reveals Differential Resistance to *Zymoseptoria tritici* at Holetta, Ethiopia

Dear Editor and Reviewer,

We sincerely thank the reviewer for the detailed and constructive comments provided throughout the evaluation process. We carefully revised the manuscript entitled Field Evaluation of Spring Wheat Genotypes Reveals Differential Resistance to *Zymoseptoria tritici* in Ethiopia accordingly. All suggested modifications have substantially improved the clarity, structure, and scientific interpretation of the study. Our detailed responses are provided below.

**Response to Reviewer Comments**

**Comment 1**

**“Indicate here the variables used for the cluster analysis. This is necessary, the reader needs to know from which variables the clusters are identified; was it severity, AUDPC, and IT means and SE (or SEM?) over 2 years?”**

**Response**

We appreciate this important comment. In the revised manuscript, the Statistical Analysis section was clarified to explicitly indicate the variables used in cluster analysis. The revised text now states that cluster analysis was performed using:

- two-year mean disease severity,
- AUDPC of disease severity, and
- pycnidia density (infection type indicator).

We also clarified that clustering was based on genotype means rather than standard errors or SEM values. The revised statement now reads:

“Cluster analysis was performed using Euclidean distance as the dissimilarity measure and Ward’s minimum variance method as the clustering criterion, based on the two-year mean values of AUDPC, severity, and pycnidia density.”

This revision improved transparency and reproducibility of the multivariate analysis.

**Comment 2**

**“Severity and AUDPC are redundant; main results should be shown only for one variable.”**

**Response**

We fully agree with the reviewer. After revision:

- Disease severity was retained as the principal epidemiological variable in the main Results section.
- AUDPC results were condensed and simplified.
- Several AUDPC tables and figures were transferred to Supplementary Materials.

The revised manuscript now emphasizes severity-based interpretation while using AUDPC only as supporting epidemiological evidence. Correlation analysis confirmed the near-complete redundancy between severity and AUDPC (r = 0.998).

**Comment 3**

**“Figure 4 is trivial and should be supplementary material.”**

**Response**

We agree and moved the redundant figure illustrating the relationship between severity and AUDPC to the Supplementary Materials. This helped reduce figure redundancy and improve manuscript readability.

**Comment 4**

**“A large part of the text is about how genotypes are ranked according to R, MR, etc., but no figure is shown to illustrate this.”**

**Response**

Following the reviewer’s recommendation and using the uploaded Excel templates, we prepared new graphical presentations illustrating:

- genotype ranking,
- disease severity,
- infection type (IT) classes,
- yearly differences between 2022 and 2023.

The revised figures now clearly present resistant (R), moderately resistant (MR), moderately susceptible (MS), and susceptible (S) genotypes separately for each year.

The corresponding Results section was completely rewritten to concisely describe these new figures.

**Comment 5**

**“Figures 2–4 should be deleted because they are redundant and varieties are categorized according to IT, not severity.”**

**Response**

We fully agree and revised the manuscript accordingly:

- Redundant figures were removed.
- Resistance classification is now consistently based on pycnidia density/infection type (IT), as described in the Materials and Methods.
- Disease severity is now treated separately as an epidemiological measurement.

This correction improved consistency between the Methods and Results sections.

**Comment 6**

**“Results indicate responses differ in both years. Therefore, separate figures are required.”**

**Response**

We appreciate this recommendation. Two separate disease-response figures were developed:

- one for the 2022 season,
- one for the 2023 season.

These revised figures simultaneously present:

- disease severity, and
- infection type classifications.

The new figures clearly demonstrate the genotype × year interaction and seasonal variation in STB pressure.

**Comment 7**

**“The text describing the figures should be rewritten and better articulated.”**

**Response**

The Results section was substantially rewritten to align with the revised graphical presentations. The revised text now:

- concisely describes yearly disease responses,
- explains genotype rankings,
- distinguishes severity from resistance classification,
- emphasizes genotype × environment interactions.

The revised section is more concise and logically organized.

**Comment 8**

**“Some tables should appear as supplementary materials.”**

**Response**

We carefully streamlined the manuscript by transferring several extensive tables to Supplementary Materials, including:

- detailed genotype ranking tables,
- complete AUDPC summaries,
- supplementary PCA and clustering tables.

This improved readability and reduced redundancy.

**Comment 9**

**“Results would be easier to present as graphs rather than tables.”**

**Response**

We agree and redesigned several results presentations using the reviewer-provided Excel templates. The revised graphical figures now better illustrate:

- disease gradients,
- resistance categories,
- yearly variation,
- genotype performance.

Corresponding detailed tables were transferred to Supplementary Materials.

**Comment 10**

**“PCA should be presented after correlation analysis.”**

**Response**

The Results section was reorganized accordingly. Correlation analysis now precedes PCA presentation, improving logical progression and interpretation.

**Comment 11**

**“Too many figures and tables.”**

**Response**

We substantially reduced figure and table redundancy by:

- deleting overlapping figures,
- moving secondary analyses to Supplementary Materials,
- simplifying graphical presentation.

The revised manuscript is now more concise and focused.

**Comment 12**

**“The Discussion is far too long and contains disconnected literature.”**

**Response**

We carefully shortened and refocused the Discussion section. General literature statements not directly linked to the present findings were removed. The revised Discussion now focuses on:

- interpretation of genotype performance,
- environmental influence on resistance expression,
- implications for breeding and disease management.

Several reviewer-identified paragraphs were deleted or condensed.

**Comment 13**

**“Interpretation of environmental effects should be reconsidered.”**

**Response**

We greatly appreciate this insightful recommendation. After re-evaluating the weather data and disease response patterns, the Discussion was revised accordingly.

The revised interpretation now states that:

- 2022 was warmer and wetter than 2023 and therefore more favorable for STB development;
- under highly conducive epidemic conditions, quantitative resistance was less effective and differences among genotypes were reduced;
- during the cooler and drier 2023 season, resistant genotypes expressed stronger disease suppression, whereas susceptible genotypes remained heavily affected.

This revised interpretation is now consistent with the meteorological data presented in Table 1 and with observed genotype responses.

**Comment 14**

**“Some unrelated literature sections should be deleted.”**

**Response**

We carefully removed several generalized literature discussions that were not directly related to interpretation of the present results. The revised Discussion is now substantially more concise, focused, and directly linked to the findings obtained in this study.

**Comment 15**

**“Please use the uploaded Word and Excel files to prepare the revised manuscript and figures.”**

**Response**

The revised manuscript was prepared directly using:

- the uploaded revised Word document,
- the reviewer-provided Excel templates for figure redesign and graphical improvement.

These files were extensively used to:

- redesign disease-response figures,
- improve cluster visualization,
- reorganize the Results section,
- shorten and improve the Discussion section.

**Comment 16**

**“New figures are needed for disease levels and clusters.”**

**Response**

New figures were developed accordingly:

- separate yearly disease-response figures,
- revised cluster analysis figure,
- simplified graphical summaries showing resistance categories and disease intensity.

The revised cluster figure now more clearly separates resistant and susceptible genotype groups and improves interpretation of multivariate disease responses.

We sincerely thank the reviewer for the insightful comments and recommendations. The revisions made in response to these suggestions substantially improved the scientific quality, clarity, interpretation, and organization of the manuscript.

Sincerely,

Habtewold Kifelew
On behalf of all authors
