# Supplementary material for: Field evaluation of spring wheat genotypes reveals differential resistance to Zymoseptoria tritici in Ethiopia
Source: PLoS One. 2026 Jul 10;21(7):e0353375. doi: 10.1371/journal.pone.0353375 (PMC13353992; doi:10.1371/journal.pone.0353375)
Supplement: Attachment — Submitted filename: PONE-D-25-41622_R4-AE.docx [file pone.0353375.s016.docx]

**Field Evaluation of Spring Wheat Genotypes Reveals Differential Resistance to Zymoseptoria tritici in Ethiopia**

Habtewold Kifelew^2^, Habtamu Terefe^2^, Bekele Kasa ^1^, Tilahun Mekonen ^3^, Zelalem Bekeko ^2^ and Bulti Tesso ^2^

^1^ Holetta Agricultural Research Center, P.O.Box 31/2003, Holeta, Ethiopia

^2^ Haramaya University, College of Agriculture and Environmental Sciences, P.O. Box 138, Dire Dawa, Ethiopia,

^3^ Addis Ababa University, Institute of Biotechnology, P.O. Box 1176, Addis Ababa, Ethiopia

Corresponding author: Habtewold Kifelew, ([kifelew@yahoo.com](mailto:kifelew@yahoo.com)) Telephone: +251913312650

**Abstract**

*Septoria tritici* blotch (STB), caused by *Zymoseptoria tritici*, is a major disease of spring wheat in Ethiopia and worldwide. This study evaluated 45 spring wheat genotypes for adult plant resistance under natural infection at Holetta Agricultural Research Center during the 2022 and 2023 seasons. Disease was assessed using two methods: (i) visual estimation of disease severity (DS) as the percentage of leaf area with necrotic lesions bearing pycnidia; and (ii) pycnidial density within lesions scored on a 0–5 scale to classify resistance levels. Combined analysis of variance across years showed significant effects (P ≤ 0.01) of year, genotype, and genotype × year interaction. Genotype accounted for 42.81% of the total variance in mean disease severity (from early grain filling to 70% flag leaf infection with pycnidia-bearing necrosis) and 42.3% of the variance in AUDPC. Mean severity ranged from 46.46% to 73.49% in 2022 and 8.73% to 71.71% in 2023, while pycnidial density ranged from 2.23% to 35.7% and 1.21% to 45.47% in 2022 and 2023, respectively. Four reaction classes were identified: resistant (24.4% and 25.2%), moderately resistant (30.4% and 24.4%), moderately susceptible (22.2% and 20.7%), and susceptible (22.96% and 29.63%) in 2022 and 2023, respectively. ‘Catbird’ was the most susceptible cultivar, whereas ‘Gondo’ was the most resistant. Cluster analysis grouped the cultivars into eight clusters based on STB severity and AUDPC, including two clusters (six genotypes) with resistant responses. These clusters did not include the differential lines used to characterize Z. tritici races, suggesting that the underlying resistance genes require further molecular characterization. The resistant genotypes (Blouk #1, 6B662, Coulter, Erik, Gondo, and ETW17-115) were effective against pathotypes virulent to major *Stb* genes present in Veranopolis (*Stb2*, *Stb6*), Shafir (*Stb6*), and Estanzuela Federal (*Stb7*). Disease severity and AUDPC were negatively correlated with plant height, number of tillers per plant, spike length, number of seeds per spike, thousand kernel weights, and grain yield. Correspondence and principal component analyses identified three major groups among the 45 genotypes: resistant, high-yield potential, and susceptible. The resistant genotypes identified here provide valuable material for breeding programs targeting improved resistance to *Z. tritici*.

**Key word**: wheat, *Septoria tritici* blotch, *Zymoseptoria tritici*, host plant resistance, breeding

**Introduction**

Spring wheat is the primary cereal crop in Ethiopia, producing 4.93 million metric tons with an average productivity of 2.73 tons per hectare. It is cultivated by 4,2 million smallholder farmers across 1.8 million hectares [1]. Recently, due to the expansion of irrigated wheat production initiatives, Ethiopia has become one of the fastest-growing wheat producers in Africa. Ethiopia wheat production is expected to reach 6.5 million metric tons by the year 2025/26 [2]. However, Ethiopia wheat productivity remains low compared to Egypt (6.4 t ha⁻¹) [3]. The low productivity is linked to various biotic and abiotic factors, as well as limited adoption of modern agricultural technologies [4]. In terms of diseases, stem and leaf rust are prevalent in low and mid-altitude regions, while stripe rust (yellow rust) and Septoria are more common in high-altitude wheat-growing areas of Ethiopia [5, 6, 7, 8]

Particularly in the highlands, *Zymoseptoria tritici,* the pathogen causing Septoria tritici blotch (STB) disease, is among the most devastating diseases of wheat in Ethiopia, ranking second only to stripe rust. This pathogen is responsible for yield losses that range from 41% [9] to 82% [5, 6]. In addition to lowering productivity, *Z. tritici* infection degrades grain quality [10]. Due to frequent STB outbreaks and a lack of effective disease management strategies, wheat production in the area has recently become challenging.

Evaluating spring wheat for resistance to STB is essential in worldwide wheat breeding because of the pathogen evolutionary ability and the rising concern of fungicide resistance [11, 12]. STB can lead to considerable yield reductions, making genetic resistance the most sustainable and eco-friendly control method. Screening initiatives mainly concentrate on detecting two types of resistance, qualitative and quantitative (associated with Quantitative Trait Loci, QTL). Qualitative resistance results from major Stb genes which confer strong, race-specific resistance, typically following a gene-for-gene interaction model. Though successful against non-virulent pathogen strains, they are susceptible to being outpaced by the pathogen swift development of virulence. To date, 24 resistance (R) genes against *Z. tritici* have been identified and documented [13]. Among them, 23 *Stb* genes have been genetically mapped in wheat [14]. These genes were found in wheat through field trials and studies on interactions between genotypes and isolates. Resistance to STB is primarily quantitative under field conditions [15, 16]. Quantitative resistance offers a less complete yet long-lasting defense, governed by numerous genes (QTLs) with smaller, cumulative impacts. Contemporary breeding increasingly seeks a combination, or pyramiding, of both significant *Stb* genes and QTLs to enhance protection that is more resilient and durable. Currently the wheat genome contains a large number of QTLs linked to resistance [17, 18, 19]. Since 2015, there have been findings indicating that 89 chromosomal regions harbor QTLs associated with resistance to STB [15]. Alternative origins, such as synthetic hexaploid wheat, are being explored to integrate newly effective resistance genes such as Stb16q [20]. A recent study using QTL analysis discovered that genes involved in both quantitative and qualitative STB-wheat interactions are similar. The study also demonstrated that genes underlying pathogenicity QTL can be analogous to *Avr* genes [21].

Studies in Ethiopia have revealed variability in resistance among bread wheat, durum wheat, and triticale [22]. Eyal and his team [23, 24] investigated the virulence traits of 97 *Z. tritici* isolates obtained from 22 countries, including Ethiopia, by evaluating them on 35 varieties of wheat and triticale. Their findings confirmed isolate-cultivar specificity, indicating diverse virulence genes in the pathogen population. Molecular analyses by Tilahun [25] further demonstrated substantial genetic variability in resistance among 180 Ethiopian bread wheat genotypes. Genome-Wide Association Studies (GWAS) conducted on Ethiopian wheat have effectively pinpointed important genomic areas and new germplasm sources that provide consistent resistance to STB. These findings highlight the complex, polygenic basis of resistance and underline the importance of addressing genotype-by-environment interactions [26, 27]. Screening programs in Tigray [28], Gondar [29], and Holetta [30] have also contributed to identifying resistant genotypes.

The purpose of this study was to assess phenotypic variation in spring wheat germplasm to identify resistance to STB. To achieve this, 45 spring wheat genotypes, including 10 differential lines for STB, were assessed under natural infection over two consecutive years at an open field site in the central highlands of Ethiopia. Agronomic traits were also measured on the same genotypes, enabling an analysis of the relationship between resistance and agronomic characteristics in Ethiopian spring wheat.

**Materials and methods**

**Description of the study area**

The genotypes were evaluated at the Holetta Agricultural Research Center in Ethiopia, under field conditions during the 2022 and 2023 cropping seasons. The location is expected to represent the major and potential spring wheat production area in central highlands of Ethiopia, the location is also recognized as a hotspot for STB epidemics. The center is 2,400 meters above sea level and located at latitude 9°00' N and longitude 38°30' E. The site has minimum and maximum temperatures of 6°C and 22°C, respectively; it receives 1,144 mm of rainfall annually (Table 1). The soil type known as nitosols has a pH of 6.0 [31].

| Weather variables* | Year 2022 | | | | | Year 2023 | | | | |
| --- | --- | --- | --- | --- | --- | --- | --- | --- | --- | --- |
|  | June | July | August | September | Mean | June | July | August | September | Mean |
| T^O^ min. | 9 | 10.2 | 10.5 | 9.8 | 9.875 | 9.8 | 10.5 | 10.2 | 10 | 10.13 |
| T^O^ max. | 26.5 | 25.7 | 25.9 | 24.5 | 25.65 | 22.3 | 20.9 | 21.2 | 21.4 | 21.45 |
| Rainfall(mm) | 200.7 | 390.8 | 331.7 | 218.7 | 285.48 | 112.4 | 202.3 | 271.8 | 166.4 | 188.23 |
| RH (%) | 77 | 84 | 81 | 78 | 80 | 81 | 84 | 84 | 80 | 82.25 |

**Table.1. Weather condition of Holetta during spring wheat growing season 2022, 2023**

Source:-Holetta agricultural research center, weather station

*T^O^ min=minimum temperature, T^O^ max= maximum temperature, Rainfall=mean monthly average rainfall amount, RH=Relative humidity percentage

**Plant materials**

A total of 45 bread wheat genotypes were evaluated for their reaction to the *Z. tritici* field population at Holetta during the 2022 and 2023 cropping seasons. Most of these genotypes were recently introduced to the country, and their disease resistance status is not yet known. Differential lines—genotypes carrying known major resistance genes to STB were included so as to identify the specific races against which the genotype shows resistance, which can help in gene postulation. The susceptible variety “Paven- 76,” the moderately susceptible variety “Kingbird,” the moderately resistant variety “Alidoro,” and the resistant variety “Hedasie” were included as controls (Table 2).

**Table.2. Spring wheat genotypes included in the study at Holetta, during 2022 and 2023 Cropping season**

| Genotype short name | Genotype | Pedigree | Origin | Reason for including in this study and *Stb* genes | Reference |
| --- | --- | --- | --- | --- | --- |
| Biq | BIQA | ETBW 6095 | EIAR | *Has Stb* (2, 3,5,7,8,9,14,18) | [25] |
| Mer | Meraro | *M/4/HAR 1709/ 3/M//24/E* | EIAR | *Has Stb* (5,6,7,8,11,13,16,17) | [25] |
| Ali | Alidoro | *HK-14-R251* | EIAR | *Has Stb* (2,3,4,5,6,7, 8,9, 11, 14,16,17,18) | [25] |
| Mad | Madda walabu | *TI/3/Fn/Th/Nar59 *2/4/Bol'S'* | EIAR | *Has Stb* (2,3,4,5,6, 8,9,13, 14,16,17,18) | [25] |
| Hul | Hulluka | *UTQUE96/3/PYN/BAU//MILAN* | OARI | *Has Stb* (3,4,5,7,8,13) | [25] |
| Kin | Kingbird | TAM200/TUI/6/PVN//CAR422/ANA/5/BOW/CROW//BUC/PVN/3/YR/4/TRAP#1 | EIAR | *Has Stb* (1,2,3,4,5,7, 8,11,13 ,17) | [25] |
| Dan | Danda’a | *KIRITATI//2*PBW65/2*SERI.1B* | EIAR | *Has Stb* (2,3,4,7,9,13,14,18) | [25] |
| Et | ET-13A2 | *UQ 105 SEL. X ENKOY* | EIAR | *Has Stb* (3,4,5,6,9,13,14,) | [25] |
| Pavon | Pavon-76 | *CMl399-D-4M-3Y-lM-l Y-lM-QY* | Mexico | *Has Stb* (3,5,6,8,9,11,12, 13, 14,17,18) | [25] |
| Sof | Sofumar | *LIRA 'S'/TAN"S"* | OARI | *Has Stb* (2,3,7,8,9,13,14,17) | [25] |
| Ki6295 | KI 6295-4A | *Romany X GB-GAMENYA* | EIAR | *Has Stb* (1,2,3,5,8,9,11,13, 14,16,17,18) | [25] |
| K6 | K 6290 Bulk | *(AF.MAYOXGEM)XROMANY* | EIAR | *Has Stb* (2, 3,5,7,8,9,14,18) | [25] |
| Hog | Hoggana | *PYN/BAU//MILAN* | EIAR | *Has Stb* (5,6,7,8,11, 13,16,17) | [25] |
| Sal | Salamouni | NA | Canada | Stb13 + Stb14* | [32] |
| 6B6 | 6B662 | NA | Brazil | Differential line tan spot | [33] |
| Cou | Coulter | DT-188/Dt-224//DT-182 | Israel | ** | [34] |
| Eri | Erik | Kitt/2/Waldron/Era | USA/ HRSW-KS | ** | [34] |
| Gle | Glenlea | Pemina*2/Bage/2/CB100 | CIMMYT | Differential line for tan spot | [33] |
| Nd | ND-495 | Justin*2/3/ND259/Conley//ND112 | Uruguay | ** | [34] |
| Ver | Veranopolis | K-44430; PI-214401; PI-297008; AFRC-5645 | Ammiad, Israel | Has *Stb2 + Stb6** | [35, 36] |
| Isr | Israel 493 | NA | Iran | Has the *Stb3* and *Stb6** | [[35]](https://bsppjournals.onlinelibrary.wiley.com/doi/10.1111/j.1365-3059.2004.01052.x#b16) |
| Tad | Tadina | PI-494096; AUS-24432; AUS-23036 | USA |  | [37, 38] |
| Sha | Shafir | K-62169 | CIMMYT | *Stb6** | [[39]](https://bsppjournals.onlinelibrary.wiley.com/doi/10.1111/j.1365-3059.2004.01052.x#b26) |
| Est | Estanzuela Federal | NA | Brazil | *Stb7** | [40] |
| Kk4 | KK4500 | NA | CIMMYT | Has the *Stb* (6,7,10,12)* | [38] |
| Km7 | KM7 | NA | CIMMYT | *Stb16** | [41] |
| Gon | Gondo/CBRD | NA | USA | ** | [34] |
| Br18 | BR 18 | NA | CIMMYT | ** | [34] |
| Cat | Catbird | NA | CIMMYT | Used as source of resistance by CIMMYT | [42] |
| Br34 | BR 34 | ALZ110 / 2* IAS54 /6/ TP /4/ TZPP /SON64 | CIMMYT | ** | [34] |
| Fro | Frontana | NA | CIMMYT | Veranopolis and KK4500 are derived from Frontana | [[43]](https://bsppjournals.onlinelibrary.wiley.com/doi/10.1111/j.1365-3059.2004.01052.x#b7) |
| Mut | Mutus | NA | Brazil | ** | [34] |
| Mur | Murga | NA | CIMMYT | Stb16* | [34] |
| Blo | Blouk #1 | NA | CIMMYT | ** | [34] |
| Chi | MP 4106 | Chiba//PRLII/cM65531/3/SKAUZ/BAV92 | CIMMYT | ** | [34] |
| Cia | CIANO T79 | CM31678-R-4Y-2M-21-0M | Mexico | ** | [34] |
| Sok | Line | SOKOLL//W15.92/WBLL1 | CIMMYT | ** | [34] |
| Wax | Line | WAXWING*2/CIRCUS | ICSISA SB6737 | ** | [34] |
| Hid | Hidasie | ANAC/3/PRL/SARA//TSI/VEE#5/4/CROC-1/AE.SQUAROSA(224)//OPATTA | CIMMYT | Has good resistance to STB |  |
| Etw86 | Line | ETW17-86 | EIAR | ** |  |
| Etw221 | Line | ETW17-221 | EIAR | ** |  |
| Etw246 | Line | ETW17-246 | EIAR | ** |  |
| Etw85 | Line | ETW17-85 | EIAR | ** |  |
| Etw115 | Line | ETW17-115 | EIAR | ** |  |
| Zer | Line | ZERBA-6/FLAG-6/3/TAM200/PASTOR//TOBA97 | EIAR | ** |  |

EIAR: Ethiopian Institute of Agricultural Research; CIMMYT: International Maize and Wheat Improvement Center; NA: Not available; *: STB differential line with known resistance major gene; **: the specific *Stb* genes were not clearly identified.

**Sowing and experimental design**

Both experiments were established according to a randomized complete block design, with three replications. The experimental area underwent thorough tillage, being plowed three times prior to sowing, and planting rows were established with hand-pulled row maker. Seeds were sown using the hand drilling technique at a depth of approximately five cm. Spring wheat genotypes were planted on 28 June, in accordance with the recommended sowing period for spring wheat at Holetta [44]. Each plot consisted of four rows, each measuring one meter in length and spaced 20 cm apart, with a seeding rate of 150 kg per hectare [44]. Inorganic fertilizers were uniformly applied across all plots at a rate of 100 kg per hectare of di-ammonium phosphate (DAP) and 150 kg per hectare of urea, following the recommendations for spring wheat cultivation. The entire amount of DAP and half of the urea were applied at the time of planting, while the remaining half of the urea was added during the mid-tillering phase. Hand weeding was performed to manage weed growth in the experimental field [44].

**Disease assessment and computation of disease variables**

Susceptibility and resistance levels were assessed based on the ability of the pathogen to infect host tissue and produce pycnidia. Disease assessments started at the early grain filling stage of wheat development [45], which is generally when the disease begins under Holetta conditions. Disease was assessed weekly for six weeks, ending when flag leaf infection reached 70% in susceptible genotypes. In each plot, five plants were randomly selected from each of the four corners and the center (X-shaped pattern), totaling 25 plants per plot for estimation of disease. Disease was assessed according to two criteria: disease severity (DS) and pycnidial density (PC).

Disease severity was estimated visually as the percentage of leaf area covered by necrotic lesions containing pycnidia. This assessment employed a modified version of the double-digit (00–99) scoring system developed by Saari and Prescott, which is based on a severity scale for evaluating foliar diseases in wheat [46, 47]. The scoring system disease severity (1 to 9) is as follows: 10% coverage corresponds to a score of 1, 20% coverage to 2, and so on until 90% coverage corresponding to a score of 9. These measurements were combined to calculate the overall disease severity using the following formula:

$$Disease severity \left( \% \right)=\left( \left( \frac{D1}{Y1} \right)*\left( \frac{D2}{Y2} \right) \right)*100$$

Where D1 represents disease vertical progression relative to plant height. The greater the disease reaches upper leaves, especially when it affects the flag leaf (which plays a key role in yield), the more serious the damage.  D2 reflects the diseased leaf area, and corresponds to the (1-9) score-scale described above. This provides the most straightforward indication of tissue damage and reduced ability for photosynthesis. Y1 is the maximum scale value for D1, which is the maximum possible score for the vertical progression of the disease, Y2 is the maximum scale value for D2, that is, the maximum possible score for the intensity of the diseased leaf area [46, 47]. The mean of disease severity over the six assessments was then computed and used for further analyses, and is referred later-on as "disease severity".

The area under the disease progress curve (AUDPC) of disease severity [48, 25] was calculated using the following formula:

$$AUDPC \%.day=\sum_{i=1}^{n-1} \frac{y_{i}+y_{i+1}}{2}\times(t_{i+1}-t_{i})$$

Where, y_i_ denotes disease severity at the i^th^ observation; t_i_ is the time, expressed in days, associated with the i^th^ observation; and n” is the total number of assessments.

The level of sporulation of lesions was estimated as pycnidial density within necrotic lesions using a 0–5 scale, where 0 = no sporulation; 1 = occasional pycnidia in a few lesions; 2 = low density of pycnidia in many or most lesions, usually unevenly distributed; 3 = even distribution of pycnidia at moderate density over most lesions; 4 = high density of pycnidia distributed over most lesions; and 5 = maximum pycnidial density [50, 51] (Table 3).

**Table 3. Pycnidial density within necrotic lesions: scoring scale, range of infection percentage, mid-point percentage used to calculate means across replicates, and corresponding genotype reaction (infection type) classification [50]**

| Pycnidia density Score | Range of pycnidia density within a lesion (%) | Mid-Point | Infection type |
| --- | --- | --- | --- |
| 0 | 0% | 0% | Immune (I) |
| 1 | 0.1–5% | 2.5% | Resistant (R) |
| 2 | 6–15% | 10.5% | Mod. Resistant (MR) |
| 3 | 16–30% | 23.0% | Mod. Susceptible (MS) |
| 4 | 31–40% | 35.5% | Susceptible (S) |
| 5 | 41–50% | 45.5% | Susceptible (S) |

The resistance levels of wheat genotypes were classified based on scores from genotype reaction (IT) obtained from the mean percentage of pycnidia density, calculated from six disease assessments. Genotype reactions (Infection types) were estimated and grouped using a 0–5 scale as described by Adhikari *et al*. and Louriki *et al*. [50, 51]. Genotypes with pycnidia density scores ranging from 0 to 1 were classified as resistant (R), a score of 2 as moderately resistant (MR), a score of 3 as moderately susceptible (MS), and scores of 4 to 5 as susceptible (S) [51].

All essential agronomic data were collected from the middle two rows within each plot. The data collection was conducted on both a plant and plot basis. For the analysis of plant-based data, a random selection of 10 plants from each plot was made to evaluate a range of characteristics. These characteristics included the number of tillers per plant, the number of kernels per spike, the spikelet count per spike, as well as measurements of plant height (cm), and spike length (cm). Additionally, further data were gathered on the density of productive tillers per square meter, the weight of a thousand kernels (grams), and the grain yield (t/ha) on a plot-specific basis.

**Statistical analyses**

In a first stage, the effects of genotype, year, and their interaction on disease severity (mean over six assessments), AUDPC of disease severity, and the agro-morphological variables were assessed with analyses of variance (ANOVA). The R statistical software (version 4.5.2) was used, following the standard procedures outlined by Gomez and Gomez [52]. Severity percentage data were transformed using arcsine transformation prior to analyses [53]. The validity of further statistical analyses, such as ANOVA, was ensured by using Bartlett's test to determine whether the error variances for distinct genotypes across different attributes were comparable. ANOVA and other parametric statistical tests require the assumption of homoscedasticity, which was examined prior to analysis. When ANOVA indicated significant differences among genotypes, mean comparisons were performed using the least significant difference (LSD) test at a 5% level of significance.

In a second stage, associations between disease severity (mean over six assessments), AUDPC of disease severity, and agronomic traits were assessed with multivariate analyses. Diversity in disease resistance among genotypes was assessed using cluster analysis, correspondence analysis, and principal component analysis (PCA) based on agro-morphological and disease-related traits.

Continuous variables were converted into categorical ones because correspondence analysis is based on contingency tables [54, 55]. Quartiles provide a common and robust discretization method, dividing data into four equal-frequency groups and accommodating skewed distributions [56, 57]. Continuous variables (disease and agronomic traits) were discretized into categorical variables, each one including four frequency-based groups (Q1–Q4). Quartile values for each variable are displayed in Table 4.

**Table 4. Quartile values from wheat genotype categorization based on disease, yield, and agronomic traits, used to classify variables into High, Medium, and Low groups with corresponding genotypes.**

| Trait | Q1(Low) | Medium (Q2/Q3) | Q3 (high) |
| --- | --- | --- | --- |
| Genotype | - | - | - |
| Severity | 0.7779 | 0.8514 | 0.9125 |
| AUDPC | 2027.7 | 2290.2 | 2577.2 |
| Pycnida density (P) | 2.5 | 10.5 | 23 |
| Plant height (PH) | 83.5 | 88.0 | 97.2 |
| Number of productive tiller per plant (NTPP) | 2.507 | 2.601 | 2.692 |
| Spike length (SL) | 6.53 | 7.27 | 7.73 |
| Number of seed per spike (NSPS) | 6.40 | 6.48 | 6.69 |
| Thousand kernel weight (TKW) | 30.0 | 33.0 | 36.3 |
| Yield tons per hectare (Yieldt.ha) | 2.08 | 2.31 | 2.83 |

Cluster analysis was performed using Euclidean distance as the dissimilarity measure and Ward’s minimum variance method as the clustering criterion, based on the two-year mean values of AUDPC, severity, and pycnidia density. Correspondence analysis and PCA were used to explore associations among genotypes, disease variables, and agronomic traits. All analyses were conducted using R statistical software (version 4.5.2).

**ETHICS STATEMENT:** The study was approved by review and Ethics Committee, Harumaya University, Agriculture and Environmental Sciences School of Plant Sciences in February, 2022

**Results**

**Analysis of Variance (ANOVA)**

The combined analysis of variance (ANOVA) reveals a significant (P < 0.01) effect of the genotypes on disease severity and AUDPC (Table 5). The high significance of the year effect indicates that environmental conditions in 2022 and 2023 differed in their conduciveness to STB development. Furthermore, a significant Genotype × Year (G x Y) interaction suggests that specific genotypes responded differently to these seasonal variation. The ANOVA results further indicate that genotype is the main factor contributing to variation in resistance to STB within the 45 spring wheat collection, accounting for 42.81% of the total variance in disease severity and 42.3% in AUDPC (Table 5). All genotypes showed high broad-sense heritability values (0.7094 for severity and 0.7132 for AUDPC; Table 5) for these traits, suggesting that resistance to STB can be enhanced through breeding.

**Table 5. Analysis of variance for the effect of spring wheat genotype on *Septoria tritici* at Holetta, Ethiopia, during the 2022 and 2023 cropping seasons**

| Trait | Factor | SS | Mean Square | Pr > F | %SS | h_2_^2^ % |
| --- | --- | --- | --- | --- | --- | --- |
| Severity* | Genotype | 3.233 | 0.074 | <.0001 | 42.81 | 0.7094 |
|  | year | 0.562 | 0.562 | <.0001 | 7.4 |  |
|  | Genotype*year | 2.994 | 0.07 | <.0001 | 39.6 |  |
|  | Residual | 0.763 | 0.0043 |  | 10.1 |  |
| AUDPC | Genotype | 56508295.41 | 1284279.44 | <.0001 | 42.3 | 0.7132 |
|  | year | 8214937.84 | 8214937.84 | <.0001 | 6.15 |  |
|  | Genotype*year | 54402880.83 | 1236429.11 | <.0001 | 40.7 |  |
|  | Residual | 14508570 | 81508.8 |  | 10.86 |  |

SS- a sum of squares; MS- mean squares; h_b_^2^- broad-sense heritability index.

*Severity value	refers to the average of severity over the 6 assessments

**Genotype-by-Year Interaction Effects**

The combined analysis of variance (ANOVA) across the 2022 and 2023 growing seasons revealed highly significant differences (P < 0.01) among genotypes, years, and their interactions for nearly all investigated traits (Table 6). The highly significant genotype effect (MSG) for STB severity, AUDPC, and grain yield confirms the presence of substantial genetic diversity within the 45 spring wheat genotypes, providing a robust basis for identifying resistant and high-yielding lines. While the significant year effect (MSY) and genotype-by-year interaction (MSGY) for disease variables and most agronomic traits highlight the strong influence of seasonal environmental fluctuations on STB development and plant growth, the non-significant MSGY for grain yield (0.015^NS^) suggests that the relative yield performance of the genotypes remained stable across environments. Furthermore, high coefficients of determination (R^2^) ranging from 0.89 to 0.95 for the primary disease and growth metrics, coupled with manageable coefficients of variation, underline the reliability of the experimental conditions at Holetta for differentiating the phenotypic responses of the wheat population (Table 6).

**Table.6. Combined analysis of variance over the year for STB Severity, grain yield, and yield-related characters of spring wheat genotype grown at Holetta during the 2022 and 2023 growing seasons**

| Trait | MSG (44) | MSY (1) | MSGY (44) | MSB | MSE | Mean | Cv. | R^2^ | LSD |
| --- | --- | --- | --- | --- | --- | --- | --- | --- | --- |
| Severity | 0.0735^**^ | 0.5618^**^ | 0.068^**^ | 0.0299^**^ | 0.00428 | 0.83 | 7.86 | 0.90 | 0.0746 |
| AUDPC | 1284279.44^**^ | 8214937.84^**^ | 1236429.11^**^ | 468196.27^**^ | 81508.8 | 2317.52 | 12.32 | 0.89 | 325.28 |
| Plant height | 1041.048^**^ | 31948.03^**^ | 602.541^**^ | 167.315^**^ | 30.2661 | 92.063 | 5.98 | 0.95 | 6.268 |
| Number of tillers per plant | 0.0819^**^ | 12.752^**^ | 0.0704^**^ | 0.2326^**^ | 0.0677 | 2.590 | 10.05 | 0.62 | 0.2965 |
| spike length | 4.3226^**^ | 428.65^**^ | 4.2447^**^ | 0.2028^**^ | 0.3393 | 7.253 | 8.03 | 0.93 | 0.6636 |
| Number of seeds per spike | 0.9343^**^ | 0.60314^**^ | 0.8623^**^ | 0.4579^**^ | 0.1959 | 6.481 | 6.83 | 0.70 | 0.5043 |
| Thousand kernel weight | 228.58199^**^ | 11774.4^**^ | 249.1234^**^ | 126.693^**^ | 163.824 | 33.515 | 38.19 | 0.53 | 14.583 |
| Yield tons/hectare | 1.999^**^ | 0.641^**^ | 0.015^NS^ | 0.01^NS^ | 0.05 | 2.43 | 9.03 | 0.91 | 0.2503 |

*, ** Significant at p ≤ 0.05, and p ≤ 0.01 probability level respectively, () parenthesis indicate degrees of freedom.

MSG= Mean Squares of Genotype, MSY= Mean squares of year, MSGY = Mean square of genotype x year interaction, MSE = Mean squares of error, MSB= Mean squares of block, CV = Coefficient of variation.

**Phenotypic Diversity for STB Resistance**

The evaluation of 45 spring bread wheat genotypes at Holetta revealed wide variation in response to STB, allowing classification into four infection types (R, MR, MS, S) (Table S1). Resistant genotypes such as 6B662, Erik, Coulter, and Gondo consistently showed low disease severity and pycnidial density (0.25–7.11), indicating stable resistance across years. In contrast, susceptible genotypes including Catbird, Tadina, and Veranopolis exhibited high severity (>65%) and pycnidial density (up to 45.47), reflecting rapid disease development and high inoculum production. A strong positive association was observed between severity and pycnidial density.

Mean separation analysis showed significant differences (P < 0.05) among genotypes for severity and AUDPC, grouping them into distinct statistical classes. Resistant genotypes formed the lowest mean groups, while susceptible ones ranked highest. Overall, substantial phenotypic diversity was observed, with genotypes distributed across all resistance categories, indicating strong variation in STB resistance.

**Disease Progression and Intensity (Severity & AUDPC)**

**Analysis of STB Severity**

The performance of the 45 spring bread wheat genotypes regarding STB severity across both experimental years is illustrated in Figure 1. Panel A (2022) shows a relatively high baseline of infection for most genotypes. Panel B (2023) highlights a clear divergence: the resistant subset (e.g., 6B6, Etw115) shows a sharp decrease in severity compared to 2022, while susceptible lines (e.g., Catbird, Tadina) maintain or exceed 2022 levels, reaching the overall maximum of 73.67%.

The analysis revealed high disease levels across the population, with a grand mean severity of 54.92%. Genotypic responses exhibited a wide range of variation, spanning from 9.08% to 73.67%. Specifically, genotypes 6B662, ETW17-115, Coulter, Erik, and Blouk#1 demonstrated superior resistance, maintaining severity levels significantly below the population mean, a trend most pronounced during the 2023 cropping season.

Conversely, KM7, Shafir, Estanzuela Federal, and Catbird were identified as highly susceptible, with severity ratings frequently exceeding 60–70%. A significant genotype × year (G × Y) interaction was observed; while resistant genotypes maintained or improved their performance in 2023, several susceptible entries notably ZERBAA-6/FLAG-6/3/TAM200/PASTOR//TOBA97, WAXWING*2/CIRCUS, and ETW17-246—exhibited markedly increased disease intensity during the same period.

**Fig. 1.** Distribution of *Septoria Tritici* Blotch (STB) severity (%) among 45 spring bread wheat genotypes evaluated across two cropping seasons (2022 and 2023). The horizontal red dashed line represents the overall mean severity (54.92%), while the upper and lower green dotted lines indicate the overall maximum (73.67%) and minimum (9.08%) values, respectively.

**STB Diseases Progress Analysis Using AUDPC**

The performance of the 45 spring bread wheat genotypes regarding the Area Under the Disease Progress Curve (AUDPC) for *Septoria Tritici* Blotch (STB) is illustrated in Figure 2. While the AUDPC values mirrored the severity trends, they provided a more comprehensive, cumulative assessment of the epidemic progression. Panels A (2022) and B (2023) demonstrate that although the grand mean remained high (2317.52), genotypes categorized as "Low AUDPC" (positioned on the left of the x-axis) consistently restricted disease expansion across both seasons.

Statistical analysis of AUDPC revealed substantial phenotypic variation among the genotypes. Responses spanned a broad spectrum, ranging from highly resistant to highly susceptible. A distinct cohort, including ETW17-115, 6B662, Coulter, Erik, and Blouk#1, exhibited the slowest disease progression, with AUDPC values falling significantly below the population mean—reaching a minimum of 303.33 in 2023.

Conversely, genotypes such as Pavon-76, ETW17-86, WAXWING*2/CIRCUS, and Catbird consistently approached the maximum AUDPC value of 3166.33, confirming their high susceptibility. The notable fluctuations in AUDPC values for several genotypes between the two years further underscore a significant genotype-by-year (G × Y) interaction, suggesting that seasonal environmental variations played a critical role in STB epidemic development within the screening field (Fig. 2).

**Fig. 2.** Distribution of Area Under Disease Progress Curve (AUDPC) values for *Septoria Tritici* Blotch (STB) among 45 spring bread wheat genotypes. Values represent the cumulative disease progress over the growing period, with lower values indicating higher levels of quantitative resistance. The mean value (2317.5) serves as a benchmark for identifying superior genotypes relative to the population average.

**Comparative Analysis of Pycnidia Density Across Spring Wheat Genotypes and Resistance Levels (2022–2023)**

When genotypes were grouped by phenotypic resistance classification, a clear relationship emerged between visible foliar damage and pathogen reproduction (Fig. 3). The evaluation of pycnidia density across the four resistance classes revealed a stepwise increase in fungal colonization corresponding to host susceptibility.

Genotypes in the Resistant (R) group notably 6B662, Erik**,** and Gondo/CBRD effectively suppressed fungal reproduction, maintaining density scores between 0% and 2.5%. This suppression was further enhanced during the 2023 season, where many R-group genotypes scored below 0.5. In contrast, the Moderately Resistant (MR) and Moderately Susceptible (MS) groups exhibited high stability, with density scores consistently clustering around 2 (10.5%) and 3 (23.0%), respectively, across both experimental years.

The Susceptible (S) group demonstrated the highest reproductive potential. While the S group consistently scored between 3 (23.0%) and 4(35.5) in 2022, a further intensification was observed in 2023, with several genotypes including Veranopolis, Tadina, Estanzuela Federal, and Catbird attaining the maximum score of 5 (45.5).

These findings indicate a strong positive correlation between disease severity and the density of *Z. tritici* pycnidia. The results suggest that the most resistant genotypes provide a dual benefit: they not only limit necrotic leaf tissue damage but also significantly restrict the secondary inoculum potential within the field environment.

**Fig. 3.** Pycnidia density scores of 45 spring bread wheat genotypes grouped by resistance levels (R, MR, MS, and S) during the 2022 and 2023 cropping seasons. Density was rated on a 0–5 scale, where 0–1(0% - 2.5%) indicates resistance and 4–5 (35.5% - 45.5%) indicates high susceptibility. The faceted arrangement highlights the differential capacity of genotypes to suppress fungal sporulation across varying levels of host resistance.

**Multivariate Analysis of Agronomic and Disease Traits:**

**Cluster Analysis of Genotypic Responses**

Cluster analysis based on mean disease severity, AUDPC, and pycnidia density grouped the spring wheat genotypes into eight distinct clusters at a similarity threshold of 1500 (Euclidean distance) using the UPGMA method (Fig. 4 and Table S2). These clusters were further organized into two major groups: Group A (Clusters I–III) and Group B (Clusters IV–VIII), clearly separating susceptible and resistant responses. Clusters I and II exhibited the highest disease disease intensity, with Cluster I (N = 5) showing maximum severity (68.16%), AUDPC (2948.87), and pycnidia density (45.5%), followed by Cluster II (N = 6), which also displayed high susceptibility (severity = 64.75%, AUDPC = 2762.67). Cluster I included genotypes such as Estanzuela Federal (Stb7, Stb12), which showed susceptibility under Holetta conditions, while Cluster II contained KK4500 (*Stb6, Stb7, Stb10, Stb12*) and Shafir (*Stb6*), both exhibiting moderately susceptible reactions. Cluster III (N = 15), the largest group, represented moderate-to-high disease response (severity = 58.42%, AUDPC = 2471.47), and included genotypes such as Veranopolis (*Stb2, Stb6*), KM7 (*Stb16)*, and Murga (*Stb16*), with reactions ranging from susceptible to moderately resistant. Clusters VI, VII, and VIII showed intermediate disease responses, although Cluster VII (N = 2) exhibited high variability in pycnidia density, indicating heterogeneity among its genotypes.

In contrast, Group B (Clusters IV–VIII) was dominated by relatively resistant genotypes. Cluster IV (N = 3) recorded the lowest disease severity (28.95%) and AUDPC (1289.17), indicating strong resistance, while Cluster V (N = 3) showed slightly higher disease levels but the lowest pycnidia density (0.78), suggesting limited pathogen reproduction. Clusters IV, V, and VI collectively comprised genotypes with resistant to moderately resistant reactions and lacked differential lines. Cluster VII included Israel-493 (*Stb3, Stb6*), which exhibited resistance, and Meraro (*Stb6*), which was moderately susceptible, emphasizing the potential contribution of *Stb3* to resistance. Cluster VIII (N = 5), including Salamouni (*Stb13, Stb14*), displayed consistent resistant reactions with low variability. Notably, resistant genotypes identified in this study—Blouk#1, 6B662, Coulter, Erik, Gondo, and ETW17-115—demonstrated effectiveness against pathotypes virulent to major *Stb* genes found in susceptible genotypes such as Veranopolis (*Stb2, Stb6*) and Estanzuela Federal (*Stb7*). Overall, the clustering pattern effectively distinguished highly susceptible genotypes (Clusters I–II) from resistant ones (Clusters IV–V and VIII), with intermediate clusters bridging the two extremes, providing valuable insights for resistance breeding.

B

A

Fig.4. Hierarchical clustering dendrogram (UPGMA) of 45 spring bread wheat genotypes based on *Septoria Tritici* Blotch (STB) epidemiological variables (Severity, AUDPC, and Pycnidia density) under natural infection conditions in Holetta, Ethiopia (2022–2023)

**CI-CVIII = Roman numbers showing cluster; Metric variables are:- AUDPC and Severity

**Correlation Analysis of Epidemiological and Agronomic Traits**

The Pearson correlation analysis revealed a near-perfect positive association between STB severity and AUDPC (r = 0.99), indicating that single-point severity assessments were highly representative of cumulative disease progression throughout the season (Fig. 5; Fig. S1). Similarly, pycnidia density (P) showed strong positive correlations with both severity (r = 0.81) and AUDPC (r = 0.85), confirming that genotypes with higher levels of leaf necrosis also supported significantly greater fungal reproduction organs.

Regarding agronomic impact, both severity and AUDPC exerted significant negative effect on grain yield (r = -0.32 and r = -0.33, respectively) and plant height (r = -0.60 and r = -0.59, respectively). The strong negative correlation with plant height suggests that shorter genotypes in this population may be more vulnerable to vertical disease progression, or alternatively, that severe infection early in the season led to significant growth stunting. Interestingly, while the disease significantly reduced the number of productive tillers per plant (NTPP; r = -0.13) and spike length (SL; r = -0.33), the correlation with thousand kernel weight (TKW) was near zero (r = -0.04 for severity; r = -0.03 for AUDPC). This lack of a linear relationship implies that STB-induced yield losses in these 45 genotypes were primarily driven by a reduction in reproductive sinks—such as tiller survival and seeds per spike rather than by a decline in the efficiency of individual grain filling.

Fig.5. Scatter plot matrix showing mean severity scored from pycnidia bearing necrosis starting from early grain filling stage to flag leaf infection reach 70% with pycnidia bearing necrosis and AUDPC relation with yield and yield related trait on 45 spring wheat genotypes evaluated during 2022 and 2023 cropping season

**Multiple Correspondence Analysis (MCA) of Epidemiological and Agronomic Traits**

The Multiple Correspondence Analysis (MCA) biplot (Fig. 6) integrated the 45 spring wheat genotypes with their categorized disease and agronomic traits, explaining 35.9% of the total phenotypic variation along a well-defined resistance–susceptibility gradient. Dimension 1 (20.9%) functioned as the primary axis of health and productivity, contrasting high-disease indicators specifically (Severity_High, AUDPC_High, and P_High) with superior yield components. Genotypes clustered on the positive side of this dimension, such as Frontana, Kingbird, and Gondo, were strongly associated with low disease levels and high values for grain yield and reproductive sinks, including the number of productive tillers (NTPP) and seeds per spike (NSPS). In contrast, susceptible genotypes like Madda Walabu and Estanzuela Federal aligned with maximum disease intensity and reduced agronomic performance. Dimension 2 (15.0%) further differentiated genotypes based on grain weight and architecture, revealing that high thousand kernel weight (TKW_High) was somewhat decoupled from overall resistance and total grain yield. These findings effectively synchronize the results of the cluster and principal component analyses, identifying a distinct group of elite genotypes as primary candidates for STB resistance breeding programs.

Fig.6. Ordination of nine variables by correspondence analysis. Active variables included Severity, AUDPC, PH (plant height), TKW (thousand kernel weight), SL (spike length), NTPP (number of productive tillers per plant), P (pycnidia density), grain yield, and 45 spring wheat genotypes.

**Principal Component Analysis (PCA) of Epidemiological and Agronomic Traits**

Principal Component Analysis (PCA) showed that the first nine components explained 100% of the total variation among the 45 spring wheat genotypes for agronomic and *Z. tritici*-related traits (Table S3). Based on the Kaiser criterion (eigenvalues > 1), only the first three components were considered significant, jointly explaining 73% of the total variance. PC1, PC2, and PC3 contributed 42%, 19%, and 11% of the variation, respectively, with PC1 (eigenvalue = 3.817) representing the primary source of morphological and pathological diversity. The sharp decline in standard deviation from PC1 (1.95) to subsequent components indicates that most of the variability is captured within the first few dimensions, supporting their use for multivariate analysis and genotype classification.

The PCA biplot further illustrated the relationships between genotypes and traits, with the first two components explaining 61.3% of the total variance (Fig. 7). PC1 (42.4%) represented a strong yield–disease gradient, showing a clear negative association between disease traits (severity, AUDPC, and pycnidia density) and yield-related traits such as grain yield, spike length, and number of seeds per spike. Resistant genotypes, including Frontana, Kingbird, Gondo, and Alidoro, were associated with higher yield performance, whereas susceptible genotypes such as Madda Walabu, Estanzuela Federal, and Catbird clustered with high disease levels and reduced agronomic performance. PC2 (18.9%) further distinguished genotypes based on yield components, particularly thousand kernel weight and seeds per spike, reinforcing the identification of high-performing, STB-resistant genotypes.

Fig.7. Result of principal component analysis considering disease variables, yield and yield related variables of forty five different spring wheat genotypes included in the study, Holetta, Ethiopia.

**Discussion**

The present study provides a comprehensive phenotypic evaluation of 45 spring wheat genotypes for resistance to *Zymoseptoria tritici*, the causal agent of *Septoria tritici* blotch (STB), under field conditions in Ethiopia across two growing seasons. By integrating standard disease scoring, pathogen reproductive metrics, and extensive agronomic profiling through both univariate and multivariate analyses, this research allow a better understanding of the complex interactions among host genetics, pathogen epidemiology, and yield stability. The findings provide insights for refining selection strategies in wheat breeding programs aimed at developing durably resistant, high-yielding varieties for Ethiopia and similar agro-ecological zones.

The results obtained, especially form the ANOVAs, confirm substantial genetic variability for STB resistance within the tested germplasm. This level of genotypic differentiation is a fundamental prerequisite for effective phenotypic selection and aligns with previous studies reporting wide variation in STB responses among diverse wheat collections [43, 58].

Despite this strong genetic signal, the highly significant year effect emphasizes the profound influence of macro-environmental conditions on STB epidemic development. *Z. tritici* is highly sensitive to microclimatic factors, particularly prolonged leaf wetness and moderate temperatures (15–20°C), which are essential for infection and sporulation [59, 60]. The contrasting environmental conditions between the 2022 and 2023 growing seasons likely altered infection efficiency and latent periods, resulting in marked differences in disease patterns over the two years of experiment. Such inter-annual variability is characteristic of STB and emphasizes the necessity of multi-year evaluations for reliable assessment of disease resistance [61].

The significant Genotype × Year (G × Y) interaction further indicates that resistance expression was differentially influenced by seasonal conditions. This suggests that genotype rankings for resistance may shift across environments, complicating selection decisions [62]. In the wheat–*Z. tritici* pathosystem, such interactions are well documented, particularly for quantitative resistance, which is highly sensitive to fluctuations in temperature, humidity, and inoculum level [63]. These findings reinforce the importance of conducting multi-location and multi-year trials to identify stable and broadly adapted resistance sources.

Nevertheless, genotype remained the predominant source of phenotypic variation, accounting for 42.81% of total variance in disease severity and 42.3% in AUDPC. This confirms that host genetics play a central role in determining STB resistance, consistent with previous reports [64, 65]. Correspondingly, the high broad-sense heritability estimates for disease severity (H² = 0.7094) and AUDPC (H² = 0.7132) indicate that a large proportion of observed variation is genetically controlled. These values fall within the upper range reported for this pathosystem [38, 66] and suggest that phenotypic selection for reduced severity and lower AUDPC will be both reliable and effective.

Beyond visual disease severity, the assessment of pycnidia density provided critical insight into pathogen reproduction and epidemic dynamics. The results demonstrated a strong, stepwise relationship between foliar damage and fungal reproductive success, highlighting the multifaceted nature of resistance. In the wheat–*Z. tritici* system, resistance is not only defined by reduced necrotic area but also by the ability to limit pathogen reproduction [67, 22].

Resistant genotypes—such as 6B662, Erik, and Gondo/CBRD—effectively suppressed fungal reproduction, maintaining pycnidia density scores between 0 and 1. These genotypes appear to interrupt the pathogen life cycle before the transition from the biotrophic to necrotrophic phase [62]. In contrast, susceptible genotypes—including Veranopolis, Tadina, Estanzuela Federal, and Catbird—exhibited maximum pycnidia density, acting as major sources of secondary inoculum.

The strong positive correlation between disease severity and pycnidia density has important epidemiological implications. Resistant genotypes that limit pathogen reproduction provide a “dual benefit”: they preserve green leaf area while simultaneously reducing inoculum production, thereby slowing epidemic progression. This “epidemic buffering” effect not only benefits the resistant genotype itself but also reduces overall disease intensity at the field level [61, 68].

Multivariate cluster analysis, based on disease severity, AUDPC, and pycnidia density, grouped the genotypes into distinct resistance categories and revealed a critical limitation of several known major *Stb* genes. Genotypes carrying *Stb6*, *Stb7*, *Stb10*, *Stb12*, and *Stb16* were clustered within susceptible to moderately susceptible groups, indicating that the local *Z. tritici* population has evolved virulence against these genes.

For example, Estanzuela Federal (*Stb7* and *Stb12*) and KK4500 (carrying multiple *Stb* genes) were both classified as highly susceptible, alongside Shafir (*Stb6*). This supports growing evidence that widely deployed major resistance genes have lost effectiveness due to rapid pathogen adaptation [69, 70]. In contrast, resistant genotypes such as Blouk#1, 6B662, and Salamouni likely possess either novel resistance genes or effective quantitative adult plant resistance (APR), which remains resistant in the local pathogen population.

Correlation analysis further clarified the agronomic impact of STB. Disease severity and AUDPC were negatively correlated with grain yield (r ≈ -0.32) and plant height (r ≈ -0.60), highlighting the economic significance of STB. The stronger relationship with plant height may reflect either enhanced disease spread in denser canopies of shorter genotypes or physiological stunting caused by early infection [72, 73]. Importantly, STB had differential effects on yield components. While productive tiller number and spike length were significantly reduced, thousand kernel weight (TKW) was largely unaffected (r ≈ -0.04). This indicates that yield loss is primarily driven by the destruction of reproductive sinks before anthesis rather than impaired grain filling. Premature senescence reduces assimilate availability during critical stages of tiller survival and floret development [74, 75], while the remaining grains compensate by maintaining normal weight [76].

Finally, multivariate analyses (PCA and MCA) provided an integrated description of genotype performance. The PCA biplot, explaining 61.3% of total variance, clearly illustrated the trade-off between disease and yield. PC1 (42.4%) represented a gradient of plant health and productivity, with resistant genotypes (e.g., Frontana, Kingbird, Gondo, and Alidoro) positioned in the high-yield, low-disease quadrant. Similarly, MCA confirmed that effective STB resistance is associated with the preservation of key reproductive traits such as productive tiller number and number of seeds per spike. Both analyses also revealed a secondary axis separating TKW from overall resistance and yield, cautioning that selection based solely on high TKW may inadvertently favor susceptible but compensating genotypes.

The consistent performance of Frontana, a well-established source of durable partial resistance, validates its continued use as a benchmark [77]. More importantly, the identification of Gondo, Kingbird, Erik, Blouk#1, and 6B662 as high-performing, low-disease genotypes highlights their value as promising genetic resources for breeding programs targeting durable STB resistance and yield stability.

**Conclusion**

This study demonstrated substantial variation in resistance to *Septoria tritici* blotch (STB) among 45 bread wheat genotypes evaluated under Ethiopian field conditions. Genotypes such as Blouk #1, 6B662, Coulter, Erik, Gondo, and ETW17-115 exhibited strong resistance, whereas Catbird was highly susceptible. Pathotype analysis indicated widespread virulence against major Stb genes (Stb2, Stb6, and Stb7), highlighting the declining effectiveness of commonly deployed resistance genes, while avirulence was observed for Stb3, Stb13, Stb14, and Stb16. High heritability estimates and significant genotypic effects confirmed that STB resistance is largely genetically controlled, despite notable environmental influence. The strong association between disease severity and pycnidia density emphasizes the importance of selecting genotypes that limit both foliar damage and pathogen reproduction. Multivariate analyses identified Blouk #1, Gondo, Erik, 6B662, and Kingbird as elite genotypes combining low disease severity, restricted pathogen reproduction, and stable yield components. These genotypes represent valuable resources for breeding programs aiming to develop high-yielding, durably resistant wheat varieties adapted to Ethiopia and similar environments.

**CONFLICT OF INTEREST STATEMENT: The authors declare that they have no conflict of interest.**

**ACKNOWLEDGEMENTS**

For providing the experimental material, and research facility, we thank Holetta agricultural research center Breeding and Pathology program. Many thanks to plant pathology research stuff for unreserved support during the research period. We thank Ethiopian Institute of Agricultural research for the fund.

**DATA AVAILABILITY STATEMENT:** The data uploaded as minimal data set of the study used in statistical analysis.

**Reference**

1. CSA (Central Statistical Authority). Agricultural sample survey 2020/21 (2013 EC): report on livestock and livestock characteristics. Vol. II. Statistical Bulletin 589. Addis Ababa: CSA; 2021.
2. USDA. World agricultural production. Circular Series WAP 11-22 [Internet]. 2023 [cited 2026 May 6]. Available from: <https://apps.fas.usda.gov/PSDOnline/Circulars/2023/08/production.pdf>
3. USDA. Ethiopia: grain and feed annual. Report No.: ET2025-0011. Washington, DC: USDA Foreign Agricultural Service; 2025.
4. Tesfaye Z, Girma T, Tanner D, Verkuijl H, Aklilu A, Mwangi W. Adoption of improved bread wheat varieties and inorganic fertilizer by small-scale farmers in Yelma Dansa and Farta districts of northwestern Ethiopia. Mexico, D.F.: EARO and CIMMYT; 2001.
5. Mengistu H, Getaneh W, Yeshi A, Rbka D, Ayele B. Wheat pathology research in Ethiopia. In: Hailu G, Tanner DG, Mengistu H, editors. Wheat research in Ethiopia: a historical perspective. Addis Ababa: IAR/CIMMYT; 1991. p. 173–217.
6. Endale H, Getaneh W. Survey of rust and Septoria leaf blotch diseases of wheat in central Ethiopia and virulence diversity of stem rust (Puccinia graminis f. sp. tritici). Adv Crop Sci Technol. 2015;3(2):1–5.
7. Yitagesu T, Alemayehu C, Bekele K. Survey of Septoria tritici blotch of bread wheat (Triticum aestivum L.) in the central highlands of Ethiopia. Am J Biosci Bioeng. 2018;6(5):36–41.
8. Meyer M, Bacha N, Tesfaye T, Alemayehu Y, Abera E, Hundie B. Wheat rust epidemics damage Ethiopian wheat production: a decade of field disease surveillance reveals national-scale trends in past outbreaks. PLoS One. 2021;16(2):e0245697. doi:10.1371/journal.pone.0245697
9. Alemar S, Temam H. Epidemics of Septoria tritici blotch and its development over time on bread wheat in Haddiya Kambata area of southern Ethiopia. J Biol Agric Healthc. 2016;6(1):47–57.
10. Bockus WW, Bowden RL, Hunger RM, Morrill WL, Murray TD, Smiley RW. Compendium of wheat diseases and pests. 3rd ed. St. Paul (MN): APS Press; 2010.
11. Heick TM, Justesen AF, Jørgensen LN. Resistance of wheat pathogen Zymoseptoria tritici to DMI and QoI fungicides in the Nordic-Baltic region—a status. Eur J Plant Pathol. 2017;149(3):669–82.
12. Cools HJ, Fraaije BA. Update on mechanisms of azole resistance in Mycosphaerella graminicola and implications for future control. Pest Manag Sci. 2013;69(2):150–5.
13. Yang N, Ovenden B, Baxter B, McDonald MC, Solomon PS, Milgate A. Multi-stage resistance to Zymoseptoria tritici revealed by GWAS in an Australian bread wheat diversity panel. Front Plant Sci. 2022;13:990915. doi:10.3389/fpls.2022.990915
14. Suffert F, Le Prieur S, Gelisse S, Dzialo E, Saintenac C, Marcel TC. Estimating the frequency of virulence against an Stb gene in Zymoseptoria tritici populations. Plant Pathol. 2024;73(6):1573–85.
15. Brown JKM, Chartrain L, Lasserre-Zuber P, Saintenac C. Genetics of resistance to Zymoseptoria tritici and applications to wheat breeding. Fungal Genet Biol. 2015;79:33–41.
16. Dreisigacker S, Wang X, Martinez Cisneros BA, Jing R, Singh PK. Adult-plant resistance to Septoria tritici blotch in hexaploid spring wheat. Theor Appl Genet. 2015;128:2317–29.
17. Eriksen L, Borum F, Jahoor A. Inheritance and localization of resistance to Mycosphaerella graminicola in wheat genome. Theor Appl Genet. 2003;107(3):515–27.
18. Chartrain L, Brading PA, Makepeace JC, Brown JKM. Sources of resistance to Septoria tritici blotch. Plant Pathol. 2004;53(4):454–60.
19. Simon MR, Ayala FM, Corda CA, Boerner A. Molecular mapping of QTL for resistance to Septoria tritici blotch. Euphytica. 2004;138:41–48.
20. Tabib Ghaffary SM, Faris JD, Friesen TL, Visser RG, van der Lee TA, Robert O, et al. New broad-spectrum resistance to Septoria tritici blotch. Theor Appl Genet. 2012;124(1):125–42.
21. Langlands-Perry C, Pitarch A, Lapalu N, Cuenin M, Bergez C, Noly A, et al. Quantitative and qualitative plant–pathogen interactions. Front Plant Sci. 2023;14:1128546.
22. Kema GHJ, Verstappen ECP, Todorova M, Waalwijk C. Heterothallism in Mycosphaerella graminicola. Curr Genet. 1996;30:251–8.
23. Eyal Z, Amiri Z, Wahl I. Physiologic specialization of Septoria tritici. Phytopathology. 1973;63:1087.
24. Eyal Z, Scharen AL, Huffman MD, Prescott JM. Global virulence frequencies of Mycosphaerella graminicola. Phytopathology. 1985;75:1456–62.
25. Tilahun M, Teklehaimanot H, Bekele GA, Kassahun T. Virulence variability of Ethiopian Zymoseptoria tritici. Eur J Plant Pathol. 2020;158(4):895–910.
26. Kassie MM, Abebe TD, Desta EA, Tadesse W. GWAS of Septoria tritici blotch resistance. PLoS One. 2025;20(3):e0317603.
27. Yosef G, Kidane BN, Hailemariam DK, Mengistu CF. GWAS of STB resistance in Ethiopian durum wheat. Front Plant Sci. 2017;8:1586.
28. Teklay AT, Zeray SG. Occurrence and intensity of wheat STB in Tigray. Crop Prot. 2015;68:67–71.
29. Yohannes A, Birhanu F, Yeshambel A. Field response of bread wheat to STB. J Curr Res Food Sci. 2020;1(2):9–17.
30. Bekele K, Yitagesu K. Phenotyping wheat for STB at Holetta. In: Proceedings of DGGW Workshop; 2020. p. 120.
31. Tamene L, Amede T, Kihara J, Tibebe D, Schulz S, editors. Soil fertility management in Ethiopia. Addis Ababa: CIAT; 2017.
32. McIntosh RA, Devos KM, Dubcovsky J. Catalogue of gene symbols for wheat. 2007.
33. Singh PK, Singh RP, Duveiller E, Mergoum M, Adhikari TB, Elias EM. Genetics of wheat–Pyrenophora tritici-repentis. Euphytica. 2010;171:1–13.
34. Singh P. Wheat pathology research overview. CIMMYT; Mexico.
35. Wilson RE. Inheritance of resistance to Septoria tritici. In: Septoria of cereals workshop; 1985. p. 33–35.
36. Brown JKM, Kema GHJ, Forrer HR, et al. Resistance of wheat cultivars to STB. Plant Pathol. 2001;50:325–38.
37. Adhikari TB, Yang X, Cavaletto JR, et al. Mapping of Stb1 resistance gene. Theor Appl Genet. 2004;109:944–53.
38. Chartrain L, Joaquim P, Berry ST, et al. Genetics of STB resistance. Theor Appl Genet. 2005;110:1138–44.
39. Kema GHJ, Verstappen ECP, Waalwijk C. Avirulence in Mycosphaerella graminicola. Mol Plant Microbe Interact. 2000;13:1375–9.
40. McCartney CA, Brûlé-Babel AL, Lamari L, Somers DJ. Chromosomal location of resistance gene. Theor Appl Genet. 2003;107:1181–6.
41. Dalvand M, Zafar D, Pari SMJ, Roohparvar R, Ghafari SMT. Genetic diversity in Zymoseptoria tritici. J Agric Sci Technol. 2018;20(6):1307–16.
42. Dubin HJ, Rajaram S. Breeding disease-resistant wheat. Annu Rev Phytopathol. 1996;34:503–26.
43. Eyal Z. Septoria diseases of wheat. Eur J Plant Pathol. 1999;105:629–41.
44. Tufa T, Haji K, Tolera A. Seed rate and spacing effects on wheat. J Nat Sci Res. 2019;9(5).
45. Zadoks JC, Chang TT, Konzak CF. Decimal code for cereal growth stages. Weed Res. 1974;14:415–21.
46. Saari EE, Prescott JM. Scale for wheat disease intensity. Plant Dis Rep. 1975;59(5):377–80.
47. Eyal Z, Levy E. Pathogenicity patterns of Mycosphaerella graminicola. Euphytica. 1987;36:237–50.
48. Madden LV, Hughes G, Van den Bosch F. The study of plant disease epidemics. St. Paul (MN): APS Press; 2007.
49. Eyal Z, Brown MB. Estimating pycnidia density. Phytopathology. 1976;66:11–14.
50. Adhikari TB, Anderson JM, Goodwin SB. Mapping resistance gene in wheat. Phytopathology. 2003;93(9):1158–64.
51. Louriki S, Rehman S, Hanafi S, et al. GWAS of STB resistance. Front Plant Sci. 2021;12:600176.
52. Gomez KA, Gomez AA. Statistical procedures for agricultural research. 2nd ed. New York: Wiley; 1984.
53. Sokal RR, Rohlf JF. Biometry. 3rd ed. New York: Freeman; 1995.
54. Lestari KE, Utami MR, Yudhanegara MR. Correspondence analysis in statistics. AIP Conf Proc. 2023;2588:050004.
55. Mishra P, Pandey CM, Singh U, Gupta A. Scales of measurement. Ann Card Anaesth. 2018;21(4):419.
56. Canova S, Cortinovis DL, Ambrogi F. Describing univariate data. J Thorac Dis. 2017;9(6):1741–3.
57. Maslove DM, Podchiyska T, Lowe HJ. Data discretization. J Am Med Inform Assoc. 2013;20(3):544–53.
58. Orton TG, Döring TF, Shepherd SJ, et al. Disease-resistant wheat varieties. Agron Sustain Dev. 2011;31:685–96.
59. Hasan M, Mia M, Ahmed J, et al. Heat stress tolerance in wheat. Heliyon. 2024;10:e38623.
60. Ponomarenko A, Goodwin SB, Kema GHJ. Zymoseptoria tritici biology. Fungal Genet Biol. 2011;48(8):829–34.
61. Cowger C, Mundt CC. Wheat cultivar mixtures and STB. Phytopathology. 2002;92(6):617–26.
62. Kell A, Hollander DJ, Hückelhoven R. Wheat susceptibility mechanisms. Mol Plant Pathol. 2014;15(5):498–507.
63. Arraiano LS, Brown JKM, Zhan J. Resistance loss in wheat. Plant Pathol. 2009;58(2):324–32.
64. Jing HC, Lovell D, Gkintonakis K, et al. New resistance QTL. Theor Appl Genet. 2008;117(5):733–45.
65. Miedaner T, Zhao Y, Gowda M, et al. Genetic architecture of resistance. Mol Breed. 2013;32:411–23.
66. Kollers S, Rodemann B, Ling J, et al. Genome association mapping. Mol Breed. 2013;32:649–63.
67. Arraiano LS, Brown JKM. Matching resistance genes. Plant Pathol. 2017;66(4):569–80.
68. Mundt CC. Durable resistance. Infect Genet Evol. 2014;27:446–55.
69. Saintenac C, Lee WS, Cambon F, et al. Stb6 resistance gene. Nat Genet. 2018;50(3):368–74.
70. Torres JM, Figueroa M, Croll D, McDonald BA. Pathogen adaptation. Annu Rev Phytopathol. 2022;60:231–51.
71. Hess M, Folly C. Disease scoring methods. Plant Pathol. 2017;66(5):779–88.
72. Dimmock JP, Gooding MJ. Fungicide and yield relation. Field Crops Res. 2002;76:157–71.
73. Simon MR, Perelló AE, Cordo CA, Struik PC. Yield and disease intensity. J Phytopathol. 2004;152:403–8.
74. Bancal P, Robert C, Ney B. Modeling wheat yield losses. Ann Bot. 2007;100:777–89.
75. Robert C, Fournier C, Andrieu B, Ney B. Wheat–STB interaction model. Funct Plant Biol. 2004;31:63–80.
76. Foulkes MJ, Sylvester-Bradley R, Scott RK. Canopy reduction effects. Ann Appl Biol. 2002;140:29–40.
77. Gooding MJ. STB effects on yield and quality. Ann Appl Biol. 1993;123:355–63.
