# Supplementary material for: Field evaluation of spring wheat genotypes reveals differential resistance to Zymoseptoria tritici in Ethiopia
Source: PLoS One. 2026 Jul 10;21(7):e0353375. doi: 10.1371/journal.pone.0353375 (PMC13353992; doi:10.1371/journal.pone.0353375)
Supplement: Peer review history

# Peer review: Decision Letter 0

**DOI:** 10.1371/journal.pone.0353375.r001

**Authors:** Laetitia Willocquet

17 Sep 2025

PONE-D-25-41622

Field Evaluation of Bread Wheat (Triticum aestivum L.) Genotypes Reveals Differential Resistance to Zymoseptoria tritici Blotch at Holetta, Ethiopia

PLOS ONE

Dear Dr. Abebe,

Thank you for submitting your manuscript to PLOS ONE. After careful consideration, we feel that it has merit but does not fully meet PLOS ONE’s publication criteria as it currently stands. Therefore, we invite you to submit a revised version of the manuscript that addresses the points raised during the review process.

The manuscript was reviewed by three experts in the field. All three reviewers recognize the potential interest of the work, but also highlight several issues which need to be addressed. Some of these issue may require to conduct new analyses. When preparing your revised manuscript, you need to provide point to point replies to each comment made by the reviewers.

Please submit your revised manuscript by Nov 01 2025 11:59PM. If you will need more time than this to complete your revisions, please reply to this message or contact the journal office at plosone@plos.org. When you're ready to submit your revision, log on to https://www.editorialmanager.com/pone/ and select the 'Submissions Needing Revision' folder to locate your manuscript file.

Please include the following items when submitting your revised manuscript:

- A rebuttal letter that responds to each point raised by the academic editor and reviewer(s). You should upload this letter as a separate file labeled 'Response to Reviewers'.
- A marked-up copy of your manuscript that highlights changes made to the original version. You should upload this as a separate file labeled 'Revised Manuscript with Track Changes'.
- An unmarked version of your revised paper without tracked changes. You should upload this as a separate file labeled 'Manuscript'.

If you would like to make changes to your financial disclosure, please include your updated statement in your cover letter. Guidelines for resubmitting your figure files are available below the reviewer comments at the end of this letter.

If applicable, we recommend that you deposit your laboratory protocols in protocols.io to enhance the reproducibility of your results. Protocols.io assigns your protocol its own identifier (DOI) so that it can be cited independently in the future. For instructions see: https://journals.plos.org/plosone/s/submission-guidelines#loc-laboratory-protocols. Additionally, PLOS ONE offers an option for publishing peer-reviewed Lab Protocol articles, which describe protocols hosted on protocols.io. Read more information on sharing protocols at https://plos.org/protocols?utm_medium=editorial-email&utm_source=authorletters&utm_campaign=protocols.

We look forward to receiving your revised manuscript.

Kind regards,

Laetitia Willocquet, PhD

Academic Editor

PLOS ONE

Journal Requirements:

When submitting your revision, we need you to address these additional requirements.

1. Please ensure that your manuscript meets PLOS ONE's style requirements, including those for file naming. The PLOS ONE style templates can be found at

https://journals.plos.org/plosone/s/file?id=wjVg/PLOSOne_formatting_sample_main_body.pdf and

https://journals.plos.org/plosone/s/file?id=ba62/PLOSOne_formatting_sample_title_authors_affiliations.pdf.

2. In the online submission form, you indicated that [The data is available at the coorosponding author and deliver upon request].

All PLOS journals now require all data underlying the findings described in their manuscript to be freely available to other researchers, either 1. In a public repository, 2. Within the manuscript itself, or 3. Uploaded as supplementary information.

This policy applies to all data except where public deposition would breach compliance with the protocol approved by your research ethics board. If your data cannot be made publicly available for ethical or legal reasons (e.g., public availability would compromise patient privacy), please explain your reasons on resubmission and your exemption request will be escalated for approval.

3. Your ethics statement should only appear in the Methods section of your manuscript. If your ethics statement is written in any section besides the Methods, please move it to the Methods section and delete it from any other section. Please ensure that your ethics statement is included in your manuscript, as the ethics statement entered into the online submission form will not be published alongside your manuscript.

4. If the reviewer comments include a recommendation to cite specific previously published works, please review and evaluate these publications to determine whether they are relevant and should be cited. There is no requirement to cite these works unless the editor has indicated otherwise.

Additional Editor Comments (if provided):

Editor: The manuscript was reviewed by three experts in the field. All three reviewers recognize the potential interest of the work, but also highlight several issues which need to be addressed. Some of these issue may require to conduct new analyses. When preparing your revised manuscript, you need to provide point to point replies to each comment made by the reviewers

[Note: HTML markup is below. Please do not edit.]

Reviewers' comments:

Reviewer's Responses to Questions

**Comments to the Author**

1. Is the manuscript technically sound, and do the data support the conclusions?

The manuscript must describe a technically sound piece of scientific research with data that supports the conclusions. Experiments must have been conducted rigorously, with appropriate controls, replication, and sample sizes. The conclusions must be drawn appropriately based on the data presented.

Reviewer #1: Partly

Reviewer #2: No

Reviewer #3: Yes

**********

2. Has the statistical analysis been performed appropriately and rigorously?

Reviewer #1: No

Reviewer #2: I Don't Know

Reviewer #3: No

**********

3. Have the authors made all data underlying the findings in their manuscript fully available?

The PLOS Data policy requires authors to make all data underlying the findings described in their manuscript fully available without restriction, with rare exception (please refer to the Data Availability Statement in the manuscript PDF file). The data should be provided as part of the manuscript or its supporting information, or deposited to a public repository. For example, in addition to summary statistics, the data points behind means, medians and variance measures should be available. If there are restrictions on publicly sharing data—e.g. participant privacy or use of data from a third party—those must be specified.

Reviewer #1: No

Reviewer #2: No

Reviewer #3: No

**********

4. Is the manuscript presented in an intelligible fashion and written in standard English?

PLOS ONE does not copyedit accepted manuscripts, so the language in submitted articles must be clear, correct, and unambiguous. Any typographical or grammatical errors should be corrected at revision, so please note any specific errors here.

Reviewer #1: No

Reviewer #2: No

Reviewer #3: Yes

**********

5. Review Comments to the Author

Please use the space provided to explain your answers to the questions above. You may also include additional comments for the author, including concerns about dual publication, research ethics, or publication ethics. (Please upload your review as an attachment if it exceeds 20,000 characters)

Reviewer #1: The manuscript presents results from field experiments conducted over two years in order to assess the resistance level against septoria tritici blotch of 45 bread wheat genotypes in Ethiopia. The manuscript presents in my view results which can be useful for the community. There are however several issues to address prior to publication.

The English and style needs to be improved throughout the manuscript.

General comments: indicate in the title and elsewhere that the study addresses spring wheat

Introduction

The literature related to the topic needs to be included in the Introduction. This includes Kassie et al 2024 Plant Breeding and Kassie et al 2025 PONE.

L79-82: this needs to be rewritten. The reader cannot understand the value of the current work from these sentences.

Materials and Methods:

Plant materials: what is a differentia line?

Plant materials: what is the rationale for selecting these genotypes?

L11926: describe the sampling strategy: was dissease assessed from whole plot observation, or where plants or tillers selected to assess disease? If so, how many individuals were sampled, and how?

L152-9: because scores correspond to severity ranges which are not equal (range of score 1 = 5-0 =5; range of score 2 = 15-6=9; range of score 3 = 9; range of score 4 = 40-25=15; range of score 5 needs to be defined from the maximum possible severity of the disease), scores cannot be considered as a continuous variables, and means and other computations or analyses are not appropriate. Scores need to be back transformed to mid-point severity, and then this back-transformed variable can be analysed. See the article uploaded in the system for further guidance.

L161 and elsewhere: disease or agronomic features measured are not "parameters"; they are variables. The term parameter needs to be replaced throughout the manuscript

L170-2: the cluster analysis needs to be described: what were the variables used? What was the criteria used? What was the distance used?

L170-2: the PCA needs to be described: what were the variables used?

Results: new results need to be produced from the analysis using back-transformed severity data

Results: charts need to be shown which display disease intensity for the 45 genotypes. Because the ANOVA indicates that year*genotype is significant, results need to be shown for each year separately (data pooled over 2 years cannot be displayed)

Results: Fig 1 is not appropriate and has to be removed.

Results: the clusters need to be characterized with a figure displaying mean and SEM of disease variables for each cluster.

Results: the % variance explained by axis 1 and axis 2 on the PCA need to be provided.

Discussion: the results need to be compared to results reported in the literature. This includes Kassie et al 2024 Plant Breeding and Kassie et al 2025 PONE.

Reviewer #2: ***Overview***

This manuscript presents a field experiment measuring Z. tritici resistance 45 bread wheat genotypes in Ethiopia over two seasons utilizing natural infection. The work overall has potential to provide interesting results but the description and presentation of the work suffers from incoherrence and inclarity throughout the manuscript. There are some significant shortcomings in the methods and results parts of the manuscript lacking clarity and missing important references. Some issues are elementary. The data may allow for credible reanalysis and representation of the work. Currently the manuscript does not provide any basis for reproducibility of the work. Below we present some of the issues - not in the order of importance.

***Detailed***

Abstract:

L 10: Z. tritici is the pathogen.

L28: no need for quotation marks

Intro:

LL 45--47: This is already in past

L55: Zt is the pathogen, Septoria Tritici Blotch is the disease

LL63--65: There are more resistance genes already in the cited Brown et al 2015. See e.g.

Langlands-Perry C, Pitarch A, Lapalu N, Cuenin M, Bergez C, Noly A, Amezrou R, Gélisse S, Barrachina C, Parrinello H, Suffert F, Valade R and Marcel TC (2023) Quantitative and qualitative plant-pathogen interactions call upon similar pathogenicity genes with a spectrum of effects. Front. Plant Sci. 14:1128546. doi: 10.3389/fpls.2023.1128546

and

Yang N, McDonald MC, Solomon PS, Milgate AW. Genetic mapping of Stb19, a new resistance gene to Zymoseptoria tritici in wheat. Theor Appl Genet. 2018 Dec;131(12):2765-2773. doi: 10.1007/s00122-018-3189-0. Epub 2018 Sep 20. PMID: 30238255.

Materials and Methods

LL 103--: Table 1: Column S/N undefined, Column pedigree contains unclear information, Differential lines not defined (what do they differentiate, some of them Pyrenophora tritici-repentis?), is no 35 genotype and pedigree in correct order?, no 29 Catbord pedigree, no 36 duplicated at the bottom

LL 119: "such as" - whas something else also recorded? Everythin should be described in detail.

LL 121--123: does "adapted" refer to Eyal's adaptation of Saari & Prescott scale or was it adapted here further

LL 124--125: N and PC are not well defined and not used in the later descriptions, hence totally unclear

L 127: D1, D2, Y1 and Y2 are not well defined (0-9 scale probably?) - somehow related to N and PC perhaps? Basis for this conversion to percentages is not solid. If we interpret it correctly, this would give 50% if all leaves below half-height would be fully diseased and above not OR if all leaves at all heights would be 50% diseased, which is much more devastating since the top leaves are more important for yield.

LL 138--139: and several other places. Do not try to use different verb for every occasion. Select one and stick to it, since simple language makes the reading easier and more understandable.

LL 141: the plot was only the two rows, but this sounds like the two rows was a subset of the plot.

LL 141--151: Scoring and data collection not unambiguously described. Many data mentioned here is not used in the analysis and not presented in results.

L 152: 0 not described. Descriptions directly copied from Adhikari et al, but badly reformed with unnecessary verbs and omission of some words so that clarity is lost. The meaning of the percentages in parentehses is not clearly described.

Results

LL 176--: The analysis is not clearly described. What was the response variable or variables. What traits are speaking about.

LL 192--: Table 3 comes before Table 2. Not all traits are included that were mentioned in methods. Use the exact same wording throughout the presentation (main text and tables) to avoid confusion.

LL 199--200: Unclear description. What response? How do you calculate these percents? Which is overall mean and which the yearly values? Please speack in biological terms.

LL 209--212: Don't mix genotype number (from table 1) and number of genotypes (count)

LL 216--: Use the same terminology and abbreviations throughout.

L 253: UPGMA explained only here although used several times earlier.

L 263: Figure 3, quality is unacceptable

Discussion

Several sentences repeat methods or results.

LL 321--: Rest of the discussion seems out of place, lacking references alltogether, describing some basics of methods etc. as if it was written by different person for different purposes and simply pasted here. Some examples are

L 321--322: Seems like a basic description of approach, but unclearly presented.

LL 354--356: description of the basic approach behind Fig 3

L 360: AUDPC explained already for the second time in discussion despite being used from the start of the manuscript (L132).

Reviewer #3: • update your refinance, it is not 18 resistance genes now it more than 23 resistance genes identified

• Why you chose RCBD, as the number of entries increase the variability within a block increase as a result the preseason will decrease, I am not clear why the researcher choose RCBD

• I recommend correlation analysis between traits and importance of agronomic traits regarding to septoria resistance

• Fig 3 did not show PC1 and PC2 value. you have to show the value in the plot or put the eigenvalues for PC in table.

• Speculating on potential genes present in the wheat genotypes were the goals of your study. did your study achieve the objective?

• did you identify the pathotypes in your study?? if yes please indicate in your result part and if no please remove this form your conclusion

**********

6. PLOS authors have the option to publish the peer review history of their article (what does this mean?). If published, this will include your full peer review and any attached files.

If you choose “no”, your identity will remain anonymous but your review may still be made public.

**Do you want your identity to be public for this peer review?**  For information about this choice, including consent withdrawal, please see our Privacy Policy.

Reviewer #1: No

Reviewer #2: **Yes:** Petteri Karisto

Reviewer #3: No

**********

[NOTE: If reviewer comments were submitted as an attachment file, they will be attached to this email and accessible via the submission site. Please log into your account, locate the manuscript record, and check for the action link "View Attachments". If this link does not appear, there are no attachment files.]

While revising your submission, please upload your figure files to the Preflight Analysis and Conversion Engine (PACE) digital diagnostic tool, https://pacev2.apexcovantage.com/. PACE helps ensure that figures meet PLOS requirements. To use PACE, you must first register as a user. Registration is free. Then, login and navigate to the UPLOAD tab, where you will find detailed instructions on how to use the tool. If you encounter any issues or have any questions when using PACE, please email PLOS at figures@plos.org. Please note that Supporting Information files do not need this step.

Attachment Submitted filename: 2023 IndianPhytopathol disease-score.pdf

Attachment Submitted filename: PONE-D-25-41622. rev.pdf

# Author comment: Author response to Decision Letter 1

**DOI:** 10.1371/journal.pone.0353375.r002

22 Nov 2025

A rebuttal letter

Dear Sir/manadam

Thank you to all the reviewers for their important time and expert feedback. Their comments and suggestions have been acknowledged and are now included in the revised manuscript. For a detailed response, refer to the table below

Regards

1. Abstract and Introduction

💬 Reviewer Comment ✔ Agreement Response

• “The literature related to the topic needs to be included in the Introduction”. I appreciate this thoughtful remark. I concur with and acknowledge all the remarks and recommendations provided in this section. I enhance the introduction by elaborating on the background section to offer a clearer insight into the problem domain and its significance. I additionally incorporate the recommended article “Kassie et al 2024 Plant Breeding and Kassie et al 2025 PONE.” And additional pertinent recent literature and data

3. Methodology

💬 Reviewer Comment ✔ Agreement Response

• “Plant materials: what is a differentia line?

what is the rationale for selecting these genotypes?

• N and PC are not well defined

• Scoring and data collection not unambiguously described. Many data mentioned here is not used in the analysis and not presented in results.

• why the researcher choose RCBD

I appreciate this significant observation. I concur that more procedural specifics will assist others in replicating the research. I additionally incorporate the absent details in the plant material table, such as the reasons for including those materials, genotype pedigrees, and their differences.

I acknowledge the comment and have already acted on it; I provide a detailed description of the scoring and data collection and I eliminate the variables not utilized in the analysis.

The 45 genotypes were arranged in a single block measuring 58 meters, with each plot sized 2 meters by 1 meter. The land utilized had relatively low variability within the block, which is why we employed RCBD

“Explain how the sample size was determined.” Thank you for pointing this out. I have now explained how the sample size was determined, based on the standard suggested by Eyal, 20 plants per plot are enough, but we record from 25 plant. It represent the population more than 20%

“Describe the sampling strategy.” I appreciate your input. I concur that the sampling method required further explanation. We randomly select five plants from each plot of the four corners and one from the center in an X pattern, resulting in a total of 25 plants sampled from each plot for data recording

4. Results

💬 Reviewer Comment ✔ Agreement Response

• “new results need to be produced from the analysis using back-transformed severity data; Fig 1 is not appropriate and has to be removed..”

• the clusters need to be characterized with a figure displaying mean and SEM of disease variables for each cluster

• the % variance explained by axis 1 and axis 2 on the PCA need to be provided.

• The analysis is not clearly described. What was the response variable or variables. What traits are speaking about.

• Figure 3, quality is unacceptable

• I recommend correlation analysis between traits and importance of agronomic traits regarding to septoria resistance

• Speculating on potential genes present in the wheat genotypes were the goals of your study. did your study achieve the objective?

• did you identify the pathotypes in your study Thank you for noting this. I agree that and I already done the back transformation and put the result in table 4. I already remove Fig 1

I incorporated the table which composed of Characteristics of Cluster Based on Mean, Standard Deviation and Standards Error of Mean

In the PCA I included the table contain variance and Eigen values , the graph also improved

We use the variable Severity, AUDPC, and pycnidia coverage to determine the resistance nature of the genotypes

I already improved the Fig.3 and I also include correspondence analysis graph

I included the correlation analysis

The resistance genotype selected for the pathotype which affect Stb gene in Veranopolis (Stb2 and Stb6) and Estanzuela Federal (Stb7)

5. Discussion

💬 Reviewer Comment ✔ Agreement Response

“the results need to be compared to results reported in the literature. This includes Kassie et al 2024 Plant Breeding and Kassie et al 2025 PONE.” Thank you for this important comment. I have expanded the discussion to include a more robust comparison of my findings with those from recent and foundational studies. I included suggested article information and other important recent literature

Several sentences repeat methods or results. including AUDPC Thank you for this helpful comment. I removed those sentences from the discussion part.

Attachment Submitted filename: A rebuttal letter.docx

# Editor report: Decision Letter 1

**DOI:** 10.1371/journal.pone.0353375.r003

**Authors:** Laetitia Willocquet, Laetitia Willocquet

28 Jan 2026

PONE-D-25-41622R1

Field Evaluation of Spring Wheat (Triticum aestivum L.) Genotypes Reveals Differential Resistance to Zymoseptoria tritici Blotch at Holetta, Ethiopia

PLOS One

Dear Dr. Abebe,

Thank you for submitting your manuscript to PLOS ONE. After careful consideration, we feel that it has merit but does not fully meet PLOS ONE’s publication criteria as it currently stands. Therefore, we invite you to submit a revised version of the manuscript that addresses the points raised during the review process.

Comments from Academic Editor:

The manuscript still requires extensive revisions. Please use the word file uploaded in the system in order to prepare a revised manuscript. This will entail new analyses. The uploaded word file includes comments to be accounted for when preparing the revised manuscript.

Please submit your revised manuscript by Mar 14 2026 11:59PM. If you will need more time than this to complete your revisions, please reply to this message or contact the journal office at plosone@plos.org. When you're ready to submit your revision, log on to https://www.editorialmanager.com/pone/ and select the 'Submissions Needing Revision' folder to locate your manuscript file.

Please include the following items when submitting your revised manuscript:

- A letter that responds to each point raised by the academic editor and reviewer(s). You should upload this letter as a separate file labeled 'Response to Reviewers'.
- A marked-up copy of your manuscript that highlights changes made to the original version. You should upload this as a separate file labeled 'Revised Manuscript with Track Changes'.
- An unmarked version of your revised paper without tracked changes. You should upload this as a separate file labeled 'Manuscript'.

If you would like to make changes to your financial disclosure, please include your updated statement in your cover letter. Guidelines for resubmitting your figure files are available below the reviewer comments at the end of this letter.

If applicable, we recommend that you deposit your laboratory protocols in protocols.io to enhance the reproducibility of your results. Protocols.io assigns your protocol its own identifier (DOI) so that it can be cited independently in the future. For instructions see: https://journals.plos.org/plosone/s/submission-guidelines#loc-laboratory-protocols. Additionally, PLOS ONE offers an option for publishing peer-reviewed Lab Protocol articles, which describe protocols hosted on protocols.io. Read more information on sharing protocols at https://plos.org/protocols?utm_medium=editorial-email&utm_source=authorletters&utm_campaign=protocols.

We look forward to receiving your revised manuscript.

Kind regards,

Laetitia Willocquet, PhD

Academic Editor

PLOS One

Journal Requirements:

If the reviewer comments include a recommendation to cite specific previously published works, please review and evaluate these publications to determine whether they are relevant and should be cited. There is no requirement to cite these works unless the editor has indicated otherwise.

Additional Editor Comments:

Comments from Academic Editor:

The manuscript still requires extensive revisions. Please use the word file uploaded in the system in order to prepare a revised manuscirpt. This will entail new analyses. The uploaded word file includes comments to be accounted for when preparing the revised manuscirpt.

[Note: HTML markup is below. Please do not edit.]

[NOTE: If reviewer comments were submitted as an attachment file, they will be attached to this email and accessible via the submission site. Please log into your account, locate the manuscript record, and check for the action link "View Attachments". If this link does not appear, there are no attachment files.]

To ensure your figures meet our technical requirements, please review our figure guidelines: https://journals.plos.org/plosone/s/figures

You may also use PLOS’s free figure tool, NAAS, to help you prepare publication quality figures: https://journals.plos.org/plosone/s/figures#loc-tools-for-figure-preparation.

NAAS will assess whether your figures meet our technical requirements by comparing each figure against our figure specifications.

Attachment Submitted filename: PONE-D-25-41622_R1-AE.docx

# Author comment: Author response to Decision Letter 2

**DOI:** 10.1371/journal.pone.0353375.r004

25 Feb 2026

Response to Reviewers

Dear Reviewer,

We sincerely thank you for your careful evaluation of our manuscript and for the constructive comments and suggestions provided. We have carefully revised the manuscript to address all concerns raised. Our detailed responses are presented below.

1. Definition of differential lines

We have clarified in the revised manuscript that “differential lines” refer to wheat genotypes carrying known major resistance genes (Stb genes) to Septoria tritici blotch (STB). These lines are commonly used to identify pathogen virulence patterns and to postulate the presence of specific resistance genes based on their reaction profiles.

2. Clarification of N and associated symptoms

We have specified whether “N” (necrosis) includes pycnidia coverage (PC). In the revised version, necrosis (N) refers strictly to chlorotic and pycnidia bearing necrotic leaf area, while pycnidia density (PC) represents the proportion of pycnidia density within necrotic tissue. The symptoms associated with N—chlorotic lesions progressing to necrotic blotches—are now clearly described.

3. Disease scale discrepancies (Eyal vs. Adhikari scales)

We acknowledge the inconsistency in the description of disease scales. The revised manuscript now clearly states which scale was adopted as follows. Infected plants were evaluated using two disease assessment methods. (i) Disease severity (DS) was estimated visually as the percentage of leaf area covered by necrotic lesions containing pycnidia. This assessment employed a modified version of the double-digit (00–99) scoring system developed by Saari and Prescott, which is based on a severity scale for evaluating foliar diseases in wheat. (ii) The level of sporulation was estimated as pycnidial density within necrotic lesions using a 0–5 scale based on Adhikari and Louriki, A detailed description of the scoring criteria has been added in materials and method section.

4. Description of the scale used

We have clarified that the scoring scale corresponds to the average percentage of leaf area covered by pycnidia-bearing necrosis per leaf, assessed visually. It does not refer solely to pycnidia density within lesions but to the overall proportion of infected leaf tissue per assessed leaf.

5. Meaning of numbers in parentheses

The numbers previously shown in parentheses represented the range of percentage score of pycnidia density within necrotic lesion. This has now been explicitly explained in the table in materials and method section to avoid ambiguity.

6. Data transformation and computation of means

We have revised the statistical description to clarify that when categorical disease classes corresponded to percentage ranges, midpoint values were used for quantitative analysis (e.g., calculation of means across plants and replicates). The methodology for back-transformation and reclassification into resistance levels is now clearly described.

7. Qualitative vs. quantitative analysis

We have clarified the classification of variables as either quantitative (e.g., percentage severity, AUDPC) or qualitative (e.g., resistance categories). Where applicable, categorical analyses—such as the proportion of genotypes exhibiting low, medium, and high severity—are now explicitly described.

8. Variables and criteria used in cluster analysis

The revised manuscript now specifies the variables included in the clustering procedure (e.g., mean severity, AUDPC, pycnidia density), the distance metric applied (Euclidean distance), and the clustering method used (hierarchical clustering with Ward’s method). These details have been added to the Materials and Methods section.

9. Addition of bar chart

A new figure (bar chart) has been included presenting all genotypes with at least one assessment of disease severity and corresponding AUDPC values, as requested.

10. Computation of infection types

We have clarified how infection types were derived. Infection types were computed based on the mean percentage of pycnidia-density within a lesion across six sequential disease assessments and subsequently categorized according to predefined resistance thresholds. This procedure is now fully described in Materials and Methods.

11. Definition of severity

We have explicitly defined “severity” as the percentage of leaf area covered by necrotic lesions bearing pycnidia, assessed visually at each scoring date.

12. Graph readability

For improved clarity, genotypes name shorted, and labeled selectively to enhance legibility, as suggested.

13. Correspondence analysis categories

We have clarified that correspondence analysis was conducted using categorical variables. Continuous variables such as AUDPC and severity were first discretized into quartile-based categories (low, medium and high). The definition of each category is now clearly described in materials and method section.

14. Revision of the Discussion section

The Discussion has been substantially shortened and revised. Repetitions from the Introduction, Materials and Methods, and Results sections have been removed. The revised Discussion now focuses on synthesizing key findings, interpreting their biological relevance, and highlighting implications for resistance breeding and STB management.

We greatly appreciate the reviewer’s insightful comments, which have significantly improved the clarity, rigor, and overall quality of our manuscript.

Sincerely,

Habtewold Kifelew

Attachment Submitted filename: Response to Reviewers.docx

# Editor report: Decision Letter 2

**DOI:** 10.1371/journal.pone.0353375.r005

**Authors:** Laetitia Willocquet, Laetitia Willocquet, Laetitia Willocquet

15 Mar 2026

PONE-D-25-41622R2

Field Evaluation of Spring Wheat (Triticum aestivum L.) Genotypes Reveals Differential Resistance to Zymoseptoria tritici  at Holetta, Ethiopia

PLOS One

Dear Dr. Abebe,

Thank you for submitting your manuscript to PLOS ONE. After careful consideration, we feel that it has merit but does not fully meet PLOS ONE’s publication criteria as it currently stands. Therefore, we invite you to submit a revised version of the manuscript that addresses the points raised during the review process.

Please see comments from Academic Editor at the end of this letter, and download the word file uploaded in the system with further comments.

Please submit your revised manuscript by Apr 29 2026 11:59PM. If you will need more time than this to complete your revisions, please reply to this message or contact the journal office at plosone@plos.org. When you're ready to submit your revision, log on to https://www.editorialmanager.com/pone/ and select the 'Submissions Needing Revision' folder to locate your manuscript file.

Please include the following items when submitting your revised manuscript:

- A letter that responds to each point raised by the academic editor and reviewer(s). You should upload this letter as a separate file labeled 'Response to Reviewers'.
- A marked-up copy of your manuscript that highlights changes made to the original version. You should upload this as a separate file labeled 'Revised Manuscript with Track Changes'.
- An unmarked version of your revised paper without tracked changes. You should upload this as a separate file labeled 'Manuscript'.

If you would like to make changes to your financial disclosure, please include your updated statement in your cover letter. Guidelines for resubmitting your figure files are available below the reviewer comments at the end of this letter.

If applicable, we recommend that you deposit your laboratory protocols in protocols.io to enhance the reproducibility of your results. Protocols.io assigns your protocol its own identifier (DOI) so that it can be cited independently in the future. For instructions see: https://journals.plos.org/plosone/s/submission-guidelines#loc-laboratory-protocols. Additionally, PLOS ONE offers an option for publishing peer-reviewed Lab Protocol articles, which describe protocols hosted on protocols.io. Read more information on sharing protocols at https://plos.org/protocols?utm_medium=editorial-email&utm_source=authorletters&utm_campaign=protocols.

We look forward to receiving your revised manuscript.

Kind regards,

Laetitia Willocquet, PhD

Academic Editor

PLOS One

**Journal Requirements:**

If the reviewer comments include a recommendation to cite specific previously published works, please review and evaluate these publications to determine whether they are relevant and should be cited. There is no requirement to cite these works unless the editor has indicated otherwise.

**Additional Editor Comments:**

The revised manuscript has been improved. There are however issues to address, which are indicated below, as well as in the word file uploaded in the system. Please use that word file to prepare a revised manuscript.

1 – disease severity. It is not clear in the text if "disease severity" refers to the average over the 6 assessments, or to a specific assessment. This needs to be specified in Materials and Methods as well as in all parts of the Results showing results on diseases severity.

2 – The results section needs to be reorganized, and the number of tables and figures need to be reduced. I advise the authors to first determine the figures and tables to be shown in the results section. This should have a logical order. Then the text of results should simply and in a synthetic way describe the results. All text which provide implication of results need to be moved to the Discussion part.

An issue to be addressed in the Results is that, on the one hand, the classification of R, MR, S is based on pycnidial density. On the other hand, in Results, it seems that disease severity or AUDPC are also used to classify the genotypes. This discrepancy neds to be solved

A possible reorganisation is as follows (but see comments after the enumeration):

1 – present ANOVA results

2 – present bar charts with severity (average or at 1 assessment) and AUDPC: 1 figure for severity, one figure for AUDPC. In each figure, 2 panels (2 bar charts: 1 for each year. At this stage, genotypes should not be groups according to levels of resistance.

3 – present bar charts based on pycnidial density: one chart per year. This time, genotypes can be grouped according to levels of resistance.

4 – present results from multivariate analyses

The purpose of the results presented needs to be clarified. For example, if a criterion is stated to classify genotypes (according to pycnidial density), why make a classification on the basis of different variables? Is the purpose of the work to classify genotypes according to a pre-set criterion, or to assess and identify the best criterion of classification? This needs to be clarified.

In the text, the variables involved in the cluster analysis are still not described clearly: is it only severity, AUDPC and pycnidia density, or does it also involve agronomic traits? With respect to disease severity and pycnidial density: are all variables of the 6 assessments used, or are synthetic variables (e.g., average over 6 assessments, or last assessment) used? As of present, the reader cannot understand what was exactly done.

[Note: HTML markup is below. Please do not edit.]

[NOTE: If reviewer comments were submitted as an attachment file, they will be attached to this email and accessible via the submission site. Please log into your account, locate the manuscript record, and check for the action link "View Attachments". If this link does not appear, there are no attachment files.]

To ensure your figures meet our technical requirements, please review our figure guidelines: https://journals.plos.org/plosone/s/figures

You may also use PLOS’s free figure tool, NAAS, to help you prepare publication quality figures: https://journals.plos.org/plosone/s/figures#loc-tools-for-figure-preparation.

NAAS will assess whether your figures meet our technical requirements by comparing each figure against our figure specifications.

Attachment Submitted filename: PONE-D-25-41622_R2-AE.docx

# Author comment: Author response to Decision Letter 3

**DOI:** 10.1371/journal.pone.0353375.r006

6 Apr 2026

Response to Reviewers

Manuscript ID: PONE-D-25-41622R2

Title: Field Evaluation of Spring Wheat (Triticum aestivum L.) Genotypes Reveals Differential Resistance to Zymoseptoria tritici at Holetta, Ethiopia

Dear Dr. Willocquet,

We sincerely thank you for your careful evaluation of our revised manuscript and for the constructive comments provided. We appreciate the opportunity to further improve our work. We have carefully addressed each of the issues raised, and the revisions have been incorporated into the manuscript accordingly. Below we provide a point-by-point response to your comments.

Comment 1: Clarification of disease severity definition

“It is not clear in the text if ‘disease severity’ refers to the average over the 6 assessments, or to a specific assessment. This needs to be specified in Materials and Methods as well as in all parts of the Results…”

Response:

Thank you for this important clarification. In the revised manuscript, we have explicitly defined disease severity in the Materials and Methods section. Disease severity now refers to the mean of six successive field assessments conducted at regular intervals during the epidemic period.

We have also revised all relevant sections in the Results to consistently indicate that severity values represent the average across the six assessments, and this clarification is now consistently applied throughout the manuscript (including figure captions and table notes where applicable).

Comment 2: Organization of the Results section and reduction of tables/figures

“The results section needs to be reorganized, and the number of tables and figures need to be reduced…”

Response:

We appreciate this suggestion and have substantially reorganized the Results section to improve clarity, logical flow, and conciseness. Specifically:

• We reviewed all tables and figures and removed redundant or overlapping visuals.

• Remaining figures and tables were reorganized to follow a clearer logical sequence:

1. ANOVA results are presented first.

2. Disease severity and AUDPC results are presented next using separate, streamlined figures, each structured by year.

3. Pycnidial density results are presented separately, with genotypes grouped according to resistance classes.

4. Multivariate analysis results (e.g., PCA/cluster analysis) are presented last.

Additionally, descriptive text in the Results section has been revised to be more concise, focusing strictly on reporting findings, while interpretative statements and implications have been moved to the Discussion section in accordance with your recommendation.

Comment 3: Discrepancy in genotype classification criteria

“On the one hand, classification of R, MR, S is based on pycnidial density. On the other hand, severity or AUDPC are also used…”

Response:

We agree that the previous version lacked clarity regarding classification criteria. In the revised manuscript, we have:

• Clearly stated that pycnidial density is the primary criterion used for categorizing genotypes into resistant (R), moderately resistant (MR), and susceptible (S) groups.

• Clarified that AUDPC and disease severity are used as complementary quantitative indicators to describe epidemic intensity, but not as primary classification criteria.

• Removed any ambiguity by ensuring that genotype classification is consistently based on pycnidial density, while severity and AUDPC are presented as supporting epidemiological measures.

We have also revised the relevant sections in both the Results and Methods to explicitly distinguish between:

• Classification criteria (pycnidial density), and

• Analytical variables (severity and AUDPC).

Comment 4: Clarification of the purpose of classification and analysis

“Is the purpose of the work to classify genotypes according to a pre-set criterion, or to assess and identify the best criterion of classification?”

Response:

Thank you for raising this important conceptual point. We have clarified the objective of the study in the Introduction and reinforced it in the Methods and Discussion sections.

The primary objective of the study is to:

• Evaluate and characterize the resistance of spring wheat genotypes to Zymoseptoria tritici under field conditions, using a pre-defined classification criterion (pycnidial density) commonly used in plant pathology.

The study does not aim to identify a new classification criterion, but rather to:

• Assess genotype performance using established epidemiological and pathological parameters (severity, AUDPC, and pycnidial density), and

• Use these complementary metrics to provide a comprehensive assessment of resistance.

This clarification has been explicitly stated in the revised manuscript to avoid ambiguity.

Comment 5: Clarification of variables used in cluster analysis

“The variables involved in the cluster analysis are still not described clearly…”

Response:

We appreciate this observation and have now clarified the cluster analysis methodology in the revised manuscript.

Specifically:

• The variables included in the cluster analysis are now explicitly listed in the Methods section.

• The analysis includes disease-related variables only, namely:

o Disease severity (averaged over the six assessments),

o AUDPC,

o Pycnidial density.

Agronomic traits were not included in the cluster analysis; this has been clearly stated.

We have also clarified that:

• For disease severity and pycnidial density, summary statistics (mean over the six assessments) were used rather than individual time-point measurements.

These clarifications have been incorporated into the Methods section to ensure transparency and reproducibility.

We thank the Academic Editor for the insightful and constructive comments, which have significantly improved the clarity, structure, and scientific rigor of the manuscript. We believe that the revised version now addresses all concerns and meets the expectations for publication in PLOS ONE.

Sincerely,

Habtewold Kifelew / Corresponding Author

Attachment Submitted filename: Response_to_Reviewers_auresp_3.docx

# Editor report: Decision Letter 3

**DOI:** 10.1371/journal.pone.0353375.r007

**Authors:** Laetitia Willocquet, Laetitia Willocquet, Laetitia Willocquet, Laetitia Willocquet

30 Apr 2026

PONE-D-25-41622R3

Field Evaluation of Spring Wheat (Triticum aestivum L.) Genotypes Reveals Differential Resistance to Zymoseptoria tritici  at Holetta, Ethiopia

PLOS One

Dear Dr. Abebe,

Thank you for submitting your manuscript to PLOS ONE. After careful consideration, we feel that it has merit but does not fully meet PLOS ONE’s publication criteria as it currently stands. Therefore, we invite you to submit a revised version of the manuscript that addresses the points raised during the review process.

==============================

Please read Additional Editor Comments below for details on revisions required

==============================

Please submit your revised manuscript by Jun 14 2026 11:59PM. If you will need more time than this to complete your revisions, please reply to this message or contact the journal office at plosone@plos.org. When you're ready to submit your revision, log on to https://www.editorialmanager.com/pone/ and select the 'Submissions Needing Revision' folder to locate your manuscript file.

Please include the following items when submitting your revised manuscript:

- A letter that responds to each point raised by the academic editor and reviewer(s). You should upload this letter as a separate file labeled 'Response to Reviewers'.
- A marked-up copy of your manuscript that highlights changes made to the original version. You should upload this as a separate file labeled 'Revised Manuscript with Track Changes'.
- An unmarked version of your revised paper without tracked changes. You should upload this as a separate file labeled 'Manuscript'.

If you would like to make changes to your financial disclosure, please include your updated statement in your cover letter. Guidelines for resubmitting your figure files are available below the reviewer comments at the end of this letter.

If applicable, we recommend that you deposit your laboratory protocols in protocols.io to enhance the reproducibility of your results. Protocols.io assigns your protocol its own identifier (DOI) so that it can be cited independently in the future. For instructions see: https://journals.plos.org/plosone/s/submission-guidelines#loc-laboratory-protocols. Additionally, PLOS ONE offers an option for publishing peer-reviewed Lab Protocol articles, which describe protocols hosted on protocols.io. Read more information on sharing protocols at https://plos.org/protocols?utm_medium=editorial-email&utm_source=authorletters&utm_campaign=protocols.

As the corresponding author, your ORCID iD is verified in the submission system and will appear in the published article. PLOS supports the use of ORCID, and we encourage all coauthors to register for an ORCID iD and use it as well. Please encourage your coauthors to verify their ORCID iD within the submission system before final acceptance, as unverified ORCID iDs will not appear in the published article. *Only*  the individual author can complete the verification step; PLOS staff *cannot*  verify ORCID iDs on behalf of authors.

We look forward to receiving your revised manuscript.

Kind regards,

Laetitia Willocquet, PhD

Academic Editor

PLOS One

Journal Requirements:

If the reviewer comments include a recommendation to cite specific previously published works, please review and evaluate these publications to determine whether they are relevant and should be cited. There is no requirement to cite these works unless the editor has indicated otherwise.

Additional Editor Comments:

The manuscript requires further improvements. These includes mainly:

- new figures for the results describing the level of disease on genotypes (2 figures, one for each year), and describing the clusters. The text of the first part of the results needs to be changed so as to concisely describe the new figures.

- the Discussion needs to be shortened: many parts are not appropriate.

Please use the word file uploaded in the system to prepare a revised manuscript.

Please make use of the two excel files uploaded in the system in order to prepare new figures.

Please upload as main document the revised manuscript with all track changes accepted (clean version), and upload as additional material the manuscript with track changes visible.

[Note: HTML markup is below. Please do not edit.]

[NOTE: If reviewer comments were submitted as an attachment file, they will be attached to this email and accessible via the submission site. Please log into your account, locate the manuscript record, and check for the action link "View Attachments". If this link does not appear, there are no attachment files.]

To ensure your figures meet our technical requirements, please review our figure guidelines: https://journals.plos.org/plosone/s/figures

You may also use PLOS’s free figure tool, NAAS, to help you prepare publication quality figures: https://journals.plos.org/plosone/s/figures#loc-tools-for-figure-preparation.

NAAS will assess whether your figures meet our technical requirements by comparing each figure against our figure specifications.

Attachment Submitted filename: PONE-D-25-41622_R3 - AE.docx

Attachment Submitted filename: PONE-D-25-41622_R3 - 1 -genotype bar chart.xlsx

Attachment Submitted filename: PONE-D-25-41622_R3 - 2 -cluster bar chart.xlsx

# Author comment: Author response to Decision Letter 4

**DOI:** 10.1371/journal.pone.0353375.r008

7 May 2026

Response to Reviewers

Manuscript ID: PONE-D-25-41622R2

Title: Field Evaluation of Spring Wheat (Triticum aestivum L.) Genotypes Reveals Differential Resistance to Zymoseptoria tritici at Holetta, Ethiopia

Dear Editor and Reviewer,

We sincerely thank the reviewer for the detailed and constructive comments provided throughout the evaluation process. We carefully revised the manuscript entitled Field Evaluation of Spring Wheat Genotypes Reveals Differential Resistance to Zymoseptoria tritici in Ethiopia accordingly. All suggested modifications have substantially improved the clarity, structure, and scientific interpretation of the study. Our detailed responses are provided below.

Response to Reviewer Comments

Comment 1

“Indicate here the variables used for the cluster analysis. This is necessary, the reader needs to know from which variables the clusters are identified; was it severity, AUDPC, and IT means and SE (or SEM?) over 2 years?”

Response

We appreciate this important comment. In the revised manuscript, the Statistical Analysis section was clarified to explicitly indicate the variables used in cluster analysis. The revised text now states that cluster analysis was performed using:

• two-year mean disease severity,

• AUDPC of disease severity, and

• pycnidia density (infection type indicator).

We also clarified that clustering was based on genotype means rather than standard errors or SEM values. The revised statement now reads:

“Cluster analysis was performed using Euclidean distance as the dissimilarity measure and Ward’s minimum variance method as the clustering criterion, based on the two-year mean values of AUDPC, severity, and pycnidia density.”

This revision improved transparency and reproducibility of the multivariate analysis.

Comment 2

“Severity and AUDPC are redundant; main results should be shown only for one variable.”

Response

We fully agree with the reviewer. After revision:

• Disease severity was retained as the principal epidemiological variable in the main Results section.

• AUDPC results were condensed and simplified.

• Several AUDPC tables and figures were transferred to Supplementary Materials.

The revised manuscript now emphasizes severity-based interpretation while using AUDPC only as supporting epidemiological evidence. Correlation analysis confirmed the near-complete redundancy between severity and AUDPC (r = 0.998).

Comment 3

“Figure 4 is trivial and should be supplementary material.”

Response

We agree and moved the redundant figure illustrating the relationship between severity and AUDPC to the Supplementary Materials. This helped reduce figure redundancy and improve manuscript readability.

Comment 4

“A large part of the text is about how genotypes are ranked according to R, MR, etc., but no figure is shown to illustrate this.”

Response

Following the reviewer’s recommendation and using the uploaded Excel templates, we prepared new graphical presentations illustrating:

• genotype ranking,

• disease severity,

• infection type (IT) classes,

• yearly differences between 2022 and 2023.

The revised figures now clearly present resistant (R), moderately resistant (MR), moderately susceptible (MS), and susceptible (S) genotypes separately for each year.

The corresponding Results section was completely rewritten to concisely describe these new figures.

Comment 5

“Figures 2–4 should be deleted because they are redundant and varieties are categorized according to IT, not severity.”

Response

We fully agree and revised the manuscript accordingly:

• Redundant figures were removed.

• Resistance classification is now consistently based on pycnidia density/infection type (IT), as described in the Materials and Methods.

• Disease severity is now treated separately as an epidemiological measurement.

This correction improved consistency between the Methods and Results sections.

Comment 6

“Results indicate responses differ in both years. Therefore, separate figures are required.”

Response

We appreciate this recommendation. Two separate disease-response figures were developed:

• one for the 2022 season,

• one for the 2023 season.

These revised figures simultaneously present:

• disease severity, and

• infection type classifications.

The new figures clearly demonstrate the genotype × year interaction and seasonal variation in STB pressure.

Comment 7

“The text describing the figures should be rewritten and better articulated.”

Response

The Results section was substantially rewritten to align with the revised graphical presentations. The revised text now:

• concisely describes yearly disease responses,

• explains genotype rankings,

• distinguishes severity from resistance classification,

• emphasizes genotype × environment interactions.

The revised section is more concise and logically organized.

Comment 8

“Some tables should appear as supplementary materials.”

Response

We carefully streamlined the manuscript by transferring several extensive tables to Supplementary Materials, including:

• detailed genotype ranking tables,

• complete AUDPC summaries,

• supplementary PCA and clustering tables.

This improved readability and reduced redundancy.

Comment 9

“Results would be easier to present as graphs rather than tables.”

Response

We agree and redesigned several results presentations using the reviewer-provided Excel templates. The revised graphical figures now better illustrate:

• disease gradients,

• resistance categories,

• yearly variation,

• genotype performance.

Corresponding detailed tables were transferred to Supplementary Materials.

Comment 10

“PCA should be presented after correlation analysis.”

Response

The Results section was reorganized accordingly. Correlation analysis now precedes PCA presentation, improving logical progression and interpretation.

Comment 11

“Too many figures and tables.”

Response

We substantially reduced figure and table redundancy by:

• deleting overlapping figures,

• moving secondary analyses to Supplementary Materials,

• simplifying graphical presentation.

The revised manuscript is now more concise and focused.

Comment 12

“The Discussion is far too long and contains disconnected literature.”

Response

We carefully shortened and refocused the Discussion section. General literature statements not directly linked to the present findings were removed. The revised Discussion now focuses on:

• interpretation of genotype performance,

• environmental influence on resistance expression,

• implications for breeding and disease management.

Several reviewer-identified paragraphs were deleted or condensed.

Comment 13

“Interpretation of environmental effects should be reconsidered.”

Response

We greatly appreciate this insightful recommendation. After re-evaluating the weather data and disease response patterns, the Discussion was revised accordingly.

The revised interpretation now states that:

• 2022 was warmer and wetter than 2023 and therefore more favorable for STB development;

• under highly conducive epidemic conditions, quantitative resistance was less effective and differences among genotypes were reduced;

• during the cooler and drier 2023 season, resistant genotypes expressed stronger disease suppression, whereas susceptible genotypes remained heavily affected.

This revised interpretation is now consistent with the meteorological data presented in Table 1 and with observed genotype responses.

Comment 14

“Some unrelated literature sections should be deleted.”

Response

We carefully removed several generalized literature discussions that were not directly related to interpretation of the present results. The revised Discussion is now substantially more concise, focused, and directly linked to the findings obtained in this study.

Comment 15

“Please use the uploaded Word and Excel files to prepare the revised manuscript and figures.”

Response

The revised manuscript was prepared directly using:

• the uploaded revised Word document,

• the reviewer-provided Excel templates for figure redesign and graphical improvement.

These files were extensively used to:

• redesign disease-response figures,

• improve cluster visualization,

• reorganize the Results section,

• shorten and improve the Discussion section.

Comment 16

“New figures are needed for disease levels and clusters.”

Response

New figures were developed accordingly:

• separate yearly disease-response figures,

• revised cluster analysis figure,

• simplified graphical summaries showing resistance categories and disease intensity.

The revised cluster figure now more clearly separates resistant and susceptible genotype groups and improves interpretation of multivariate disease responses.

We sincerely thank the reviewer for the insightful comments and recommendations. The revisions made in response to these suggestions substantially improved the scientific quality, clarity, interpretation, and organization of the manuscript.

Sincerely,

Habtewold Kifelew

On behalf of all authors

Attachment Submitted filename: Response_to_Reviewers_auresp_4.docx

# Editor report: Decision Letter 4

**DOI:** 10.1371/journal.pone.0353375.r009

**Authors:** Laetitia Willocquet, Laetitia Willocquet, Laetitia Willocquet, Laetitia Willocquet, Laetitia Willocquet

1 Jun 2026

PONE-D-25-41622R4

Field Evaluation of Spring Wheat Genotypes Reveals Differential Resistance to Zymoseptoria tritici in Ethiopia

PLOS One

Dear Dr. Abebe,

Thank you for submitting your manuscript to PLOS ONE. After careful consideration, we feel that it has merit but does not fully meet PLOS ONE’s publication criteria as it currently stands. Therefore, we invite you to submit a revised version of the manuscript that addresses the points raised during the review process.

Additional minor revisions are still required. Please use the word file uploaded in the system to prepare a revised manuscript. Comments and suggestions of changes are included in this file.

Please submit your revised manuscript by Jul 16 2026 11:59PM. If you will need more time than this to complete your revisions, please reply to this message or contact the journal office at plosone@plos.org. When you're ready to submit your revision, log on to https://www.editorialmanager.com/pone/ and select the 'Submissions Needing Revision' folder to locate your manuscript file.

Please include the following items when submitting your revised manuscript:

- A letter that responds to each point raised by the academic editor and reviewer(s). You should upload this letter as a separate file labeled 'Response to Reviewers'.
- A marked-up copy of your manuscript that highlights changes made to the original version. You should upload this as a separate file labeled 'Revised Manuscript with Track Changes'.
- An unmarked version of your revised paper without tracked changes. You should upload this as a separate file labeled 'Manuscript'.

If you would like to make changes to your financial disclosure, please include your updated statement in your cover letter. Guidelines for resubmitting your figure files are available below the reviewer comments at the end of this letter.

If applicable, we recommend that you deposit your laboratory protocols in protocols.io to enhance the reproducibility of your results. Protocols.io assigns your protocol its own identifier (DOI) so that it can be cited independently in the future. For instructions see: https://journals.plos.org/plosone/s/submission-guidelines#loc-laboratory-protocols. Additionally, PLOS ONE offers an option for publishing peer-reviewed Lab Protocol articles, which describe protocols hosted on protocols.io. Read more information on sharing protocols at https://plos.org/protocols?utm_medium=editorial-email&utm_source=authorletters&utm_campaign=protocols.

As the corresponding author, your ORCID iD is verified in the submission system and will appear in the published article. PLOS supports the use of ORCID, and we encourage all coauthors to register for an ORCID iD and use it as well. Please encourage your coauthors to verify their ORCID iD within the submission system before final acceptance, as unverified ORCID iDs will not appear in the published article. *Only*  the individual author can complete the verification step; PLOS staff *cannot*  verify ORCID iDs on behalf of authors.

We look forward to receiving your revised manuscript.

Kind regards,

Laetitia Willocquet, PhD

Academic Editor

PLOS One

Journal Requirements:

If the reviewer comments include a recommendation to cite specific previously published works, please review and evaluate these publications to determine whether they are relevant and should be cited. There is no requirement to cite these works unless the editor has indicated otherwise.

Please review your reference list to ensure that it is complete and correct. If you have cited papers that have been retracted, please include the rationale for doing so in the manuscript text, or remove these references and replace them with relevant current references. Any changes to the reference list should be mentioned in the rebuttal letter that accompanies your revised manuscript. If you need to cite a retracted article, indicate the article’s retracted status in the References list and also include a citation and full reference for the retraction notice.

[Note: HTML markup is below. Please do not edit.]

[NOTE: If reviewer comments were submitted as an attachment file, they will be attached to this email and accessible via the submission site. Please log into your account, locate the manuscript record, and check for the action link "View Attachments". If this link does not appear, there are no attachment files.]

To ensure your figures meet our technical requirements, please review our figure guidelines: https://journals.plos.org/plosone/s/figures

You may also use PLOS’s free figure tool, NAAS, to help you prepare publication quality figures: https://journals.plos.org/plosone/s/figures#loc-tools-for-figure-preparation.

NAAS will assess whether your figures meet our technical requirements by comparing each figure against our figure specifications.

Attachment Submitted filename: PONE-D-25-41622_R4-AE.docx

# Author comment: Author response to Decision Letter 5

**DOI:** 10.1371/journal.pone.0353375.r010

16 Jun 2026

Response to Reviewers

Dear Dr. Laetitia Willocquet,

We sincerely thank you for your careful evaluation of our revised manuscript and for providing these valuable comments. We have carefully considered each suggestion and have revised the manuscript accordingly. Our detailed responses are provided below.

Comment 1:

This appears in the text before Table S2; so it should be renamed as Table S2; then Table S2 page 26 should be renamed Table S3. Please also rename the corresponding file; and change in the text Fig. 4 to Fig. S1.

Response:

Thank you for pointing out these inconsistencies. We have revised all supplementary material numbering throughout the manuscript. The supplementary table cited before the original Table S2 has been renamed as Table S2, while the former Table S2 has been renumbered as Table S3. The corresponding supplementary files have also been renamed accordingly. In addition, the reference to Fig. 4 in the text has been corrected to Fig. S1 to ensure consistency between the manuscript and supplementary materials.

Comment 2:

Avoid the term "pressure" which is vague, and does not relate to any physical/measurable entity.

Response:

We appreciate this important clarification. The term "pressure" has been removed or replaced throughout the manuscript with more precise terminology. Specifically, expressions such as "inoculum pressure" and "disease pressure" have been revised to more measurable descriptions, including "pathogen multiplication," "disease intensity," "infection levels," or "inoculum availability," depending on the context. These changes improve the scientific precision of the manuscript.

Comment 3:

Overstatements should be avoided: they actually damage the work.

Response:

We fully agree with this observation. We carefully reviewed the entire manuscript and revised statements that could be interpreted as overly definitive or speculative. The language has been moderated to better reflect the observational nature and scope of our field study. Conclusions are now restricted to findings directly supported by the data, and speculative interpretations have been appropriately qualified.

Comment 4:

This needs to be removed, please refer to comments from the previous round of revisions.

Response:

Thank you for highlighting this issue. Following your recommendation and the comments provided during the previous revision round, the indicated statement has been completely removed from the revised manuscript.

Comment 5:

The sentence needs to be removed: here, the mean over all assessments was used as "severity", it therefore is logical that "severity" is close to AUDPC; the deleted statement would have been correct if "severity" would have corresponded to only one date of disease assessment.

Response:

We appreciate this clarification. We agree that the very high correlation observed between severity and AUDPC largely reflects the fact that severity was calculated as the mean across repeated assessments rather than representing a single disease assessment date. Therefore, the interpretation suggesting that single-point severity measurements adequately represented cumulative disease progression was inappropriate in this context. The sentence has been removed from the manuscript.

Comment 6:

A sentence may be added here: this result is counterintuitive, because STB epidemics, assessed as disease severity, are in general considered to develop at late stage of the crop growth, i.e., after flowering.

Response:

Thank you for this insightful suggestion. We have incorporated a sentence into the Discussion acknowledging that the observed association is somewhat counterintuitive because STB epidemics, when assessed through disease severity, are generally considered to intensify during the later stages of crop development, particularly after flowering. We further note that the observed relationships may reflect the specific disease dynamics and environmental conditions prevailing during the study period.

Comment 7:

PCA and MCA are statistical analyses which describe; they can help in developing hypotheses, but cannot allow "understanding" per se.

Response:

We agree with this important conceptual distinction. The wording throughout the manuscript has been revised to avoid implying causal inference or mechanistic understanding derived from PCA and MCA. Terms such as "understanding," "revealed mechanisms," or "explained biological relationships" have been replaced with more appropriate expressions, including "described patterns," "identified associations," "illustrated relationships," and "generated hypotheses." This revision ensures that the interpretation of multivariate analyses remains consistent with their descriptive nature.

We sincerely thank the Academic Editor for these thoughtful and constructive comments. Addressing these points has substantially improved the clarity, accuracy, and scientific rigor of the manuscript. We hope that the revised version adequately addresses all concerns and will be suitable for publication in PLOS ONE.

Kind regards,

On behalf of all authors

Habtewold Kifelew

Attachment Submitted filename: Response_to_Reviewers_auresp_5.docx

# Editor report: Decision Letter 5

**DOI:** 10.1371/journal.pone.0353375.r011

**Authors:** Laetitia Willocquet, Laetitia Willocquet, Laetitia Willocquet, Laetitia Willocquet, Laetitia Willocquet, Laetitia Willocquet

23 Jun 2026

Field Evaluation of Spring Wheat Genotypes Reveals Differential Resistance to Zymoseptoria tritici  in Ethiopia

PONE-D-25-41622R5

Dear Dr. Abebe,

We’re pleased to inform you that your manuscript has been judged scientifically suitable for publication and will be formally accepted for publication once it meets all outstanding technical requirements.

Within one week, you’ll receive an e-mail detailing the required amendments. When these have been addressed, you’ll receive a formal acceptance letter and your manuscript will be scheduled for publication.

An invoice will be generated when your article is formally accepted. Please note, if your institution has a publishing partnership with PLOS and your article meets the relevant criteria, all or part of your publication costs will be covered. Please make sure your user information is up-to-date by logging into Editorial Manager at Editorial Manager® and clicking the ‘Update My Information' link at the top of the page. For questions related to billing, please contact billing support.

If your institution or institutions have a press office, please notify them about your upcoming paper to help maximize its impact. If they’ll be preparing press materials, please inform our press team as soon as possible -- no later than 48 hours after receiving the formal acceptance. Your manuscript will remain under strict press embargo until 2 pm Eastern Time on the date of publication. For more information, please contact onepress@plos.org.

Kind regards,

Laetitia Willocquet, PhD

Academic Editor

PLOS One

Additional Editor Comments (optional):

The revisions have now addressed issues identified over the successive revision rounds.

# Editor report: Acceptance letter

**DOI:** 10.1371/journal.pone.0353375.r012

**Authors:** Laetitia Willocquet, Laetitia Willocquet, Laetitia Willocquet, Laetitia Willocquet, Laetitia Willocquet, Laetitia Willocquet

PONE-D-25-41622R5

PLOS One

Dear Dr. Abebe,

I'm pleased to inform you that your manuscript has been deemed suitable for publication in PLOS One. Congratulations! Your manuscript is now being handed over to our production team.

At this stage, our production department will prepare your paper for publication. This includes ensuring the following:

* All references, tables, and figures are properly cited

* All relevant supporting information is included in the manuscript submission,

* There are no issues that prevent the paper from being properly typeset

You will receive further instructions from the production team, including instructions on how to review your proof when it is ready. Please keep in mind that we are working through a large volume of accepted articles, so please give us a few days to review your paper and let you know the next and final steps.

Lastly, if your institution or institutions have a press office, please let them know about your upcoming paper now to help maximize its impact. If they'll be preparing press materials, please inform our press team within the next 48 hours. Your manuscript will remain under strict press embargo until 2 pm Eastern Time on the date of publication. For more information, please contact onepress@plos.org.

You will receive an invoice from PLOS for your publication fee after your manuscript has reached the completed accept phase. If you receive an email requesting payment before acceptance or for any other service, this may be a phishing scheme. Learn how to identify phishing emails and protect your accounts at https://explore.plos.org/phishing.

If we can help with anything else, please email us at customercare@plos.org.

Thank you for submitting your work to PLOS ONE and supporting open access.

Kind regards,

PLOS ONE Editorial Office Staff

on behalf of

Dr. Laetitia Willocquet

Academic Editor

PLOS One
